# Siderophore-Enabled Delivery of Carbapenem and Metallo-β-Lactamase Inhibitor: A Promising Strategy against Multidrug-Resistant *Pseudomonas aeruginosa*

**DOI:** 10.1021/jacs.6c08350

**Published:** 2026-09-27

**Authors:** Shahriar Safari, Ravil Petrov, Yulia P. Ustimenko, Katy Jeannot, Xavier Trivelli, Ernesto Anoz-Carbonell, Ruben C. Hartkoorn

**Affiliations:** † 56099 Univ. Lille, CNRS, Inserm, CHU Lille, Institut Pasteur Lille, U1019 - UMR 9017 - CIIL - Center for Infection and Immunity of Lille, F-59000 Lille, France; ‡ Bacteriology Unit, University Hospital of Besançon, F-25000 Besançon, France; § Associated French National Reference Center for Antibiotic Resistance in Pseudomonas Spp and Acinetobacter Spp, F-25000 Besançon, France; ∥ 578184 Univ. Lille, CNRS, Centrale Lille, UAR2638 - IMEC - Institut Michel-Eugène Chevreul, F-59000 Lille, France

## Abstract

Carbapenem-resistant *Pseudomonas aeruginosa* (CRPA), a World Health Organization high-priority pathogen, represents a major clinical threat due to limited treatment options. Its intrinsic resistance is driven in part by extremely low outer-membrane permeability, which restricts antibiotic uptake, while the increasing prevalence of metallo-β-lactamase (MBL)-producing strains, particularly NDM-expressing isolates, poses a critical therapeutic challenge, conferring resistance to both carbapenems and cefiderocol, with few effective inhibitors available. In this study, we evaluated the potential of a series of unique biomimetic siderophore conjugates in combating CRPA. As an initial approach, tris-catecholate siderophore–meropenem conjugates were synthesized and demonstrated to overcome nonenzymatic carbapenem resistance in *P. aeruginosa*, including porin loss and efflux upregulation, in an iron-dependent manner. We then targeted MBL-expressing CRPA by vectorizing a recently developed broad-spectrum MBL inhibitor, indole carboxylates (InC), which is largely ineffective against *P. aeruginosa*, likely due to poor permeability. These conjugates restored inhibitor activity and effectively reversed meropenem and cefiderocol resistance in MBL-producing clinical isolates, both *in vitro* and in a *Galleria mellonella* infection model. Together, these findings highlight the design and potential of siderophore conjugates as a versatile platform to revive the efficacy of life-saving antibiotics against multidrug-resistant Gram-negative pathogens.

## Graphical abstract

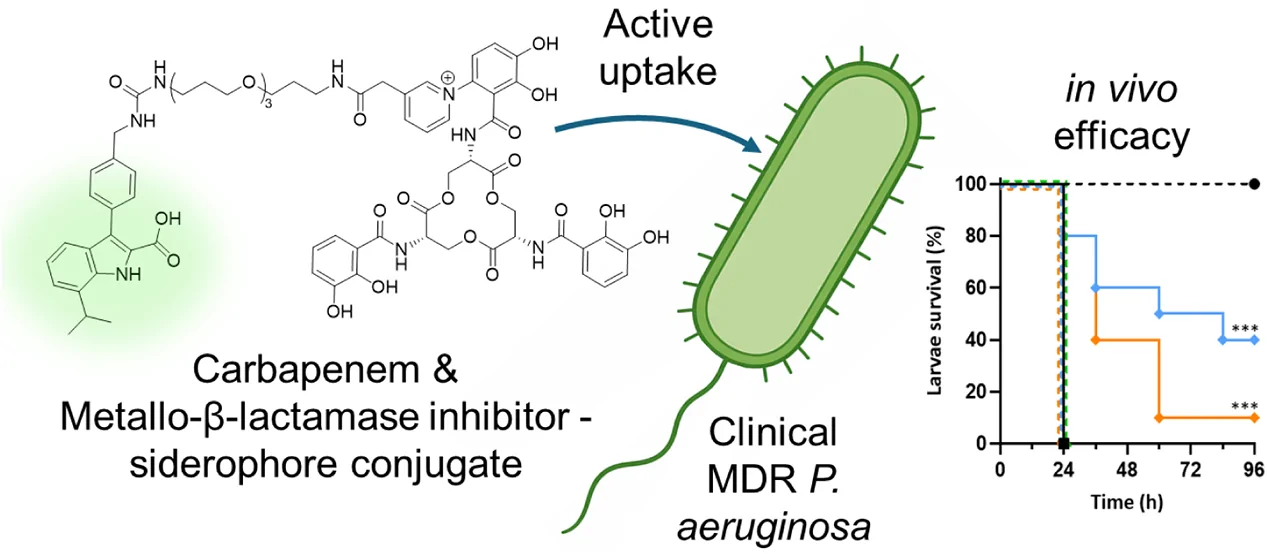

## Introduction

Antimicrobial resistance (AMR) poses a major global health threat, projected to cause millions of deaths and immense healthcare costs by 2050.^1,2^ AMR incurs approximately $900 billion in annual losses worldwide.^3^ Global efforts aim to reduce AMR-related mortality by 10% by 2030,^4^ and among these initiatives, finding solutions to fight infections by carbapenem-resistant *Pseudomonas aeruginosa* (CRPA) is a major priority. The WHO warns that current antibiotic pipelines are unlikely to meet clinical needs for *P. aeruginosa* over the next 5–7 years, underscoring the urgency for new therapeutic strategies.^5^

A major obstacle to developing novel antibiotics against CRPA is achieving potent molecules that can effectively penetrate bacteria. A distinctive feature of *P. aeruginosa* is its exceptionally impermeable outer membrane, even compared to other hard-to-penetrate Gram-negative bacteria. Indeed, studies have shown that in the case of some antibiotics, the outer membrane of *P. aeruginosa* can be up to 100 times less permeable than that of *Escherichia coli*.^6^ Beyond this baseline resistance barrier, multiple acquired mechanisms can also hinder antibiotic entry through porin alterations, reduce intracellular accumulation by upregulating efflux systems, and impair antibiotic activity via target modifications or the expression of antibiotic-degrading enzymes.^7^

With respect to carbapenem resistance in CRPA isolates, the most clinically challenging mechanism of resistance is mediated by the expression of carbapenemases. These include serine β-lactamases (SBLs), such as Class A KPC and GES, and Class D (OXA-type), as well as metallo-β-lactamases (MBLs) such as IMP, NDM, SPM, and VIM.^8^ In addition to carbapenemase production, the acquisition of mutations that alter OprD porin function greatly limit antibiotic access to the periplasm and represents another common mechanism of carbapenem resistance found in approximately 70% of CRPA isolates.^9^ By contrast, mutations in the carbapenem target penicillin-binding protein 3 (PBP3 encoded by *ftsI*) are infrequent^10^ and may even impose a fitness cost to the bacterium.^11^

As a means to combat carbapenem resistance by carbapenemases, a number of effective inhibitors of Class A and D SBL have been developed (such as avibactam, relebactam, vaborbactam), but these inhibitors lack activity on MBL.^12^ Encouragingly, the bicyclic boronate-based inhibitor, taniborbactam, currently in Phase III of clinical development, is a novel extended-spectrum β-lactamase inhibitor with activity on both SBL and many, though not all MBLs (lacks inhibition of IMP).^13^ Numerous other MBL inhibitors have also been described in recent years^14,15^ and may offer promising candidates for further clinical development. In particular, indole carboxylate (InC)-based MBL inhibitors (Figure 1) are potent reversible competitive inhibitors of NDM-1 with broad-spectrum activity against all clinically relevant MBLs^16^ and with demonstrated efficacy in murine models of infection with carbapenem-resistant *Enterobacterales.* However, likely as a result of poor permeability, these promising InC-based MBL inhibitors are less effective in *Acinetobacter* species and inactive against *P. aeruginosa*.^16^

**Figure 1:**
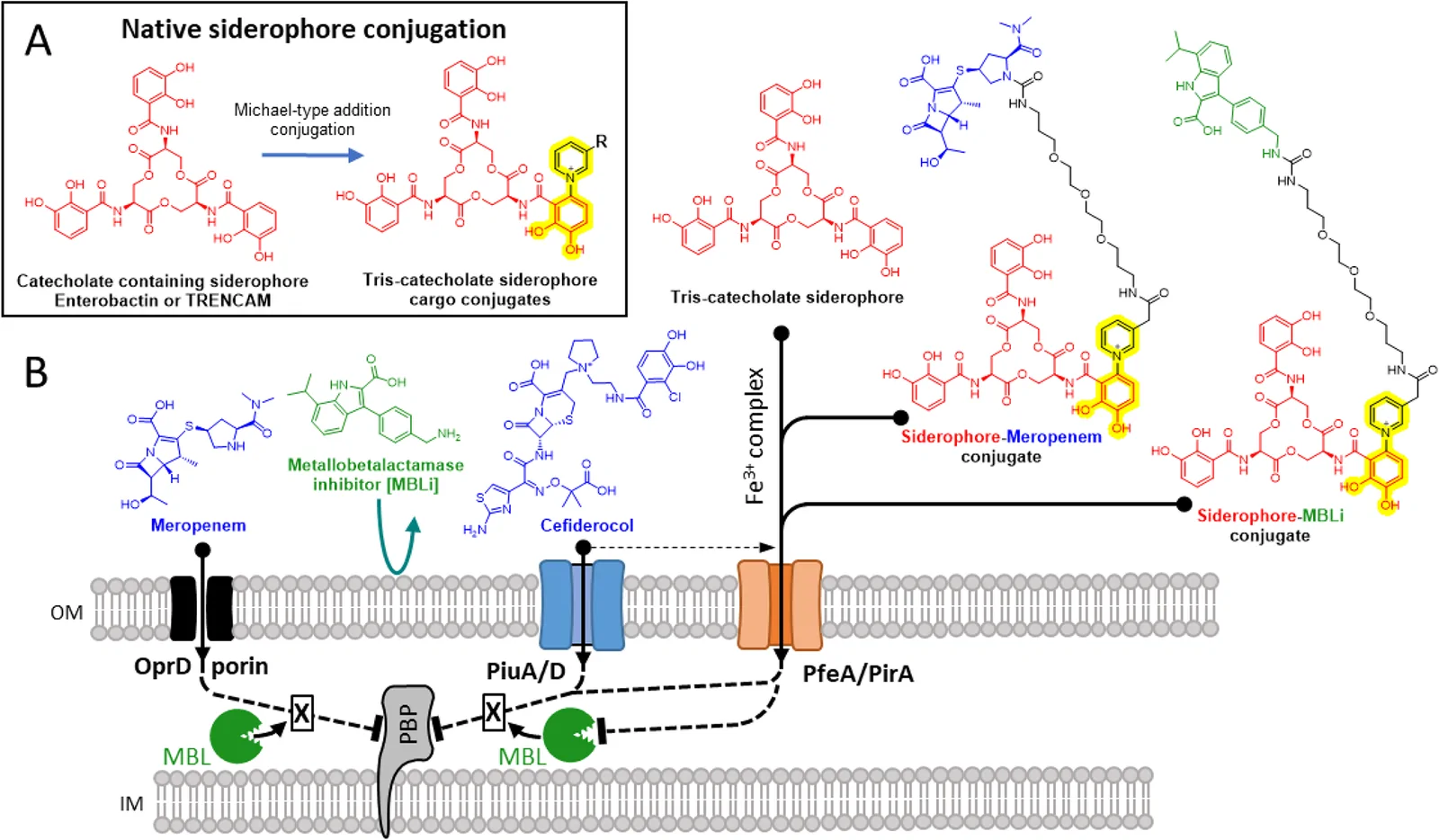
Siderophore conjugate synthesis and their proposed uptake into the periplasm of *P. aeruginosa* relevant to this work. (A) A schematic showing the general mechanism employed in this manuscript to generate siderophore-cargo conjugates using tris-catecholate siderophores. (B) Illustration depicting the route by which compounds relevant to this manuscript enter the periplasm of *P. aeruginosa* and inhibit their target. Note that TonB-dependent transporters (TBDT) likely internalize siderophores and siderophore conjugates as an Fe^3+^ complex (not shown). MBL: metallo-beta-lactamase, OM: outer membrane, IM: inner membrane, PBP: penicillin-binding protein.

To overcome the permeability barrier of Gram-negative bacteria, a number of “Trojan horse” strategies have been developed that conjugate antibiotics to carriers such as peptides, antibodies, nanoparticles, or siderophores to enhance intracellular delivery.^17^ Among these, siderophore-mediated vectorization is particularly attractive, inspired by natural sideromycins, including albomycin and salmycin, which exploit iron transport systems to gain entry into bacteria. These natural molecules have guided the rational design of synthetic siderophore–antibiotic conjugates to “vectorize” antibiotics for improved penetration.^18^ Prime examples include cefiderocol, a clinically approved cephalosporin that is able to bind iron and hijack multiple bacterial siderophore uptake systems, representing a key clinical success.^19^ The use of siderophore moieties as delivery vectors for antibiotics was pioneered by Miller and coworkers.^20,21^ In *P. aeruginosa*, this approach has focused on conjugating antibiotics to its endogenous siderophores pyoverdine and pyochelin,^22–27^ or to siderophores that the bacterium can scavenge, such as enterobactin and synthetic analogs and derivatives.^28–40^ These studies demonstrated that siderophore-based vectorization can facilitate the entry of diverse molecules into *P. aeruginosa*, representing a significant advance in designing active siderophore conjugates. However, among the conjugates evaluated, antibacterial activity was predominantly observed for β-lactam derivatives, such as ampicillin and amoxicillin. Owing to their β-lactam warhead, these compounds remain vulnerable to β-lactamase-mediated hydrolysis.^41,42^ To address this limitation, several studies have investigated the use of β-lactamase inhibitors in combination with β-lactam siderophore conjugates, demonstrating enhanced activity against Gram-negative bacteria, although the extent of this activity varied across species and was observed primarily in strains producing serine β-lactamases.^21,40,43–45^ Furthermore, the efficacy of such combinations can be attenuated in Gram-negative bacteria exhibiting reduced outer membrane permeability, which restricts the entry of β-lactamase inhibitors and limits their effectiveness in restoring the activity of β-lactam siderophore conjugates.^46^

Our recent work presented the discovery of a novel natural chlorodactyloferrin–pyridomycin siderophore–antibiotic conjugate from *Dactylosporangium fulvum*.^47^ Uniquely, it was demonstrated that the macromolecular conjugation of this siderophore–antibiotic could be mimicked through a chemical conjugation mechanism where, following oxidation of the siderophore catechol, the resultant o-quinones could undergo a Michael-type addition reaction on C-6 with 3-pyridyl nitrogen moieties of cargo molecules generating unique pyridinium–catechol conjugates (Figure 1). By changing the 3-pyridyl-containing cargo molecule, we showed that this reactivity enables the construction of novel chlorodactyloferrin–antibiotic and chlorodactyloferrin–fluorophore conjugates, that were effectively transported into bacterial cells as Trojan horse molecules.^47^ To date, the utility of this reaction has yet to be demonstrated for other catechol-containing siderophores, such as enterobactin.

In this work, we set out to determine whether siderophore-mediated vectorization could restore the activity of a potent indole carboxylate-based inhibitor against MBL-producing *P. aeruginosa*, particularly strains expressing clinically challenging NDM enzymes. To address this, we employed tris-catechol siderophore vectors, enterobactin and its synthetic analog TRENCAM,^48^ and conjugated cargos to these vectors using our recently developed, nature-inspired pyridine–catechol linkage, which enables attachment without requiring any modification of the siderophore scaffold. Establishing such a strategy could provide a means to preserve the efficacy of last-resort agents, including carbapenems like meropenem and a newer reserve antibiotic, cefiderocol,^49^ for the treatment of DTR (difficult-to-treat) *P. aeruginosa* infections.

## Results

### Investigating Silver-Salt Assisted Iodine-Mediated Pyridine–Catechol Conjugation

We previously reported the discovery of the biomimetic synthesis of catecholate-siderophore conjugates using established catechol oxidants such as Fe^3+^, silver oxide, and iodine, allowing for the potential conjugation of natural catechol-containing siderophores to pyridine-functionalized cargo molecules.^47^ This pyridine–catechol coupling was shown to be initiated by two-electron oxidation of the catechol, followed by a Michael-type addition of the nucleophilic pyridine nitrogen on carbon 6 of the quinone. When attempting to translate these conjugation reactions to form pyridine conjugates of the *tris*-catecholate siderophore enterobactin, the ferric iron-based approach was found to be largely ineffective, possibly due to the very negative redox potential of the tris-catecholate siderophore ferric iron complex.^50,51^ Encouragingly, however, early experiments indicated that iodine as a catechol oxidant could enable the formation of enterobactin conjugates of the same type. Despite this encouraging insight, we were wary that the use of iodine could lead to undesired side reactions (primarily β-lactam ring opening, as well as other nonspecific oxidation reactions), which could be problematic for making conjugates with β-lactam antibiotic cargo molecules (part of the aim of this manuscript). To address this, we looked for an alternative synthetic approach that would maintain the mild oxidant property of iodine while suppressing the side reactions potentially mediated by the nucleophilic nature of the iodide anion.^52^ An intriguing possibility was to investigate whether the *in situ* formation of pyridine iodonium salts, previously reported as mild oxidants,^53^ could promote the conjugation reaction. To avoid the need to isolate and purify the iodonium salts, an *in situ* strategy was adopted in which the addition of silver­(I) salts sequesters iodide as insoluble AgI, thereby shifting the equilibrium toward the formation of the iodide cation (I^+^) to form pyridine-iodonium salts. This approach also offered the potential advantage of suppressing undesirable side reactions associated with the iodide anion (I^–^).

To explore if pyridine-iodonium salts could mediate catechol-to-quinone oxidation and allow for the subsequent conjugation, we undertook an exploratory approach using simplified model substrates together with different combinations of iodine and silver salts. Specifically, a model 3-methylpyridine derivative (**MS1**) and a model catechol derivative (**MS2**) were synthesized as reaction substrates to enable good monitoring of the reaction by both UV absorption and mass spectrometry (UHPLC-UV-MS). Control experiments conducted in the absence of iodine (with or without the silver salts) did not allow for the formation of an MS1-MS2 conjugate (**MS3**) (Figure 2, entries 1–3), while the expected reactivity was observed in the presence of 1 eq. of iodine (entry 4). The addition of Ag(0) nanoparticles was found to have no impact on the iodine-mediated coupling (entry 5). The addition of 1–2 eq. of silver­(I) salts (AgBF4), before or after the addition of iodine, was, however, found to increase the yield by almost 2-fold (entries 6–10), with the best yields observed (51%) with 2 eq. of the silver salt. Purification of the conjugation product **MS3** obtained in entry 6 and NMR characterization confirmed **MS3** to have the expected catechol C6–pyridine nitrogen linkage (Figure 2C and D). This silver-aided reaction also promoted the conjugation of **MS1** with enterobactin [1] in both forms (entries 12 and 13 in SI). Together, these data demonstrate that the iodonium-pyridine salt can oxidize catechol and take part as a substrate in the Michael-type addition to form the pyridine–catechol conjugates.

**Figure 2:**
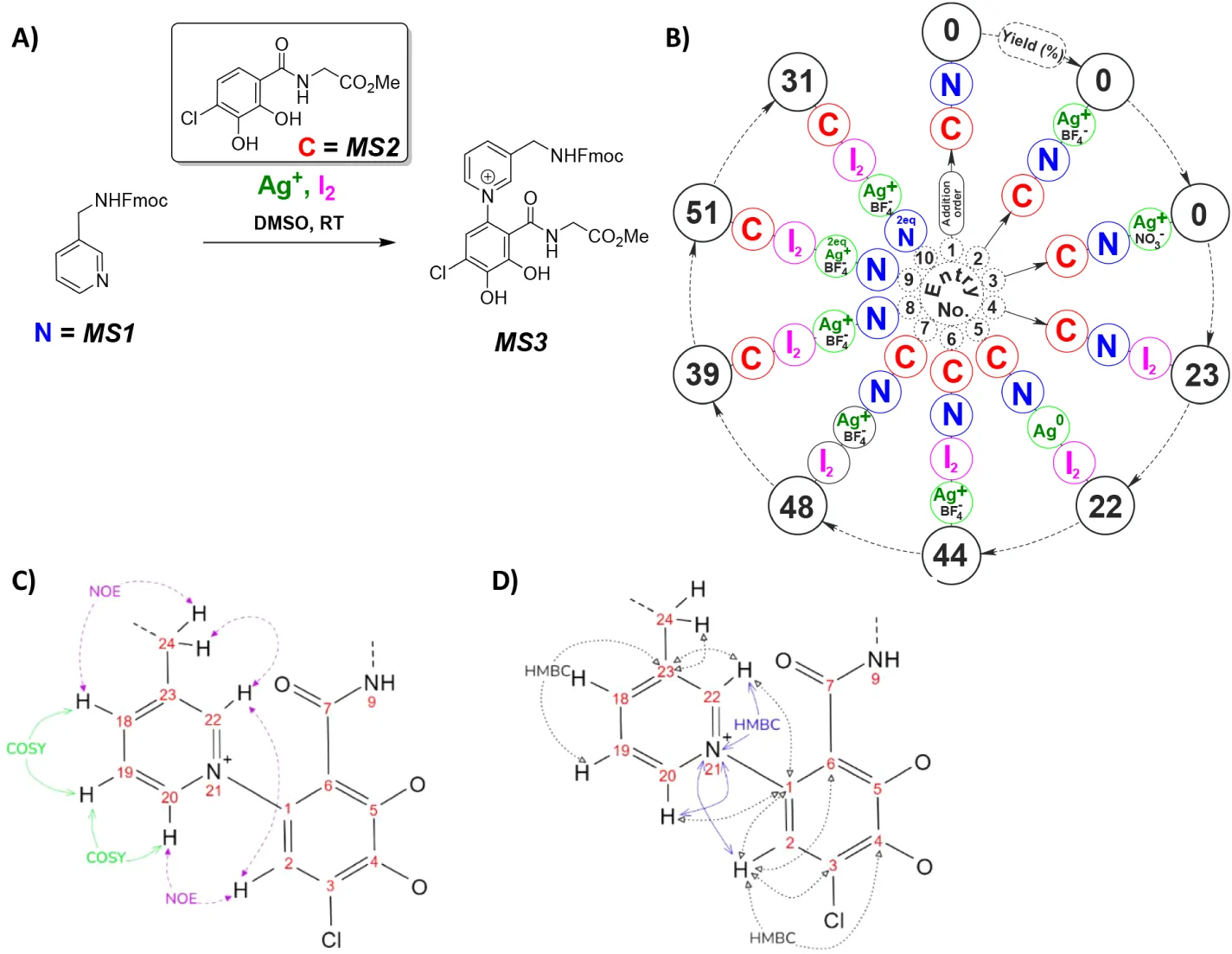
Overview of our preliminary studies focused on investigating the effect of Ag^+^ salts on oxidant activity and reaction yield. A) Model reactions between the pyridine-based substrate (***MS1***) and catechol-based substrate (***MS2***). B) Radial diagram illustrating the molar ratios and sequential addition of each component for the preparation of model substrate MS3: components are arranged radially according to the order of their introduction to the reaction mixture, with those closest to the center added first and those toward the periphery added last. Each segment reflects the relative molar ratio of each component, and the last outer circle indicates the final yield obtained for MS3 under the specified conditions as estimated from UV absorbance at 254 nm (LC–MS, for more detailed information for each entry, refer to the Supporting Information (SI). C) Characteristic ^1^H–^1^H (proton–proton) correlations (double-headed solid green arrows) based on 2D-COSY and 2D-NOESY data (double-headed dashed purple arrows). D) Characteristic ^1^H–^13^C (double-headed dotted black arrows) and ^1^H–^15^N (double-headed solid blue arrows) correlations based on 2D-HMBC data.

Next, we aimed to demonstrate that bis­(pyridine) iodonium salts can themselves play a dual role by acting as reagents and reactants at the same time by participating in the catechol oxidation and the Michael-type addition reaction (Figure 3). To achieve this, a [bis­(3-methylpyridine)­iodine­(I)]^+^ salt was prepared using AgBF_4_ according to the literature^54^ (Figure 3). Addition of this complex to either MS2 or the siderophore enterobactin resulted in the formation of the anticipated conjugation products, as confirmed by UHPLC-MS. In the case of enterobactin, the reaction afforded a mixture of mono-, bis-, and trisfunctionalized conjugates bearing 3-methylpyridine substituents (Figure S1). By adjusting the stoichiometric ratio between enterobactin and the iodonium salt, the product distribution could be modulated, enabling the preferential formation of the monofunctionalized conjugate. Of note, compared with the direct iodine-mediated oxidation protocol, the use of pyridinium-based iodonium salts as oxidants resulted in a lower proportion of bis- and trisfunctionalized conjugates. This behavior was attributed to their milder oxidative character, which provides improved control over the extent of functionalization and represents an additional advantage for the selective generation of the monofunctionalized conjugate. These experiments validated that the iodonium salts were indeed self-sufficient for both initiation and formation of the corresponding conjugates with catechol-containing compounds.

**Figure 3:**
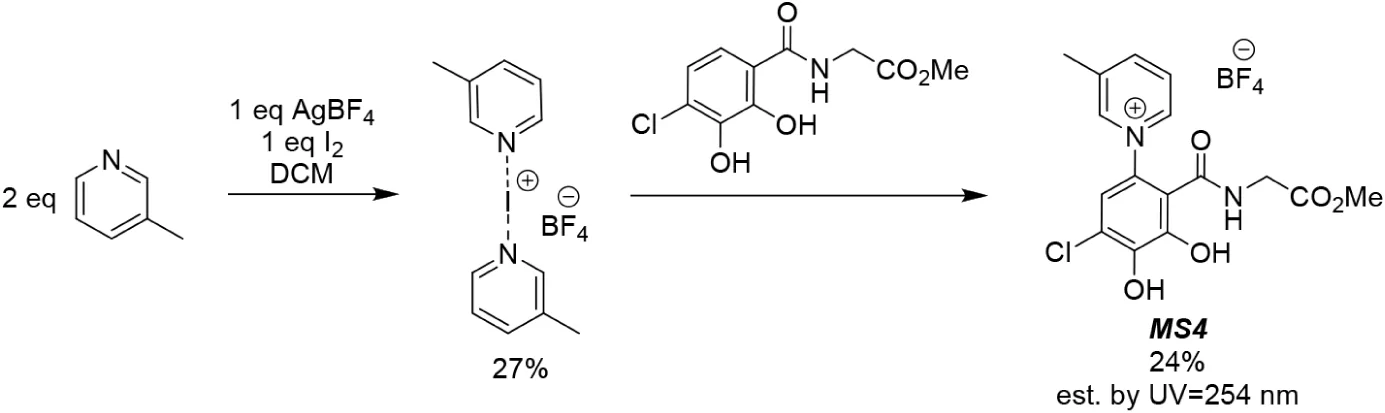
A representative modeling reaction showing that [bis­(pyridine)­iodine­(I)]^+^ -type complexes can play a dual role by acting as reagents and reactants at the same time (entry 11 in SI).

### Design and Synthesis of Enterobactin– and TRENCAM–Meropenem Conjugates

Now that we have developed a better understanding of the key parameters influencing conjugate formation with catecholate siderophores, we pursued the design and synthesis of such siderophore conjugates with cargo antibiotic molecules. The natural siderophore enterobactin [**1**] and its synthetic analog TRENCAM [**2**] (Figure 4) are both xenosiderophores that are actively taken up by *P. aeruginosa* under iron-limiting conditions.^55^ Furthermore, a recent study demonstrated the uptake of both enterobactin- and TRENCAM-based conjugates by *P. aeruginosa*.^56^ Both siderophores possess catechol groups, making them suitable candidates for conjugation with 3-pyridine-containing cargo molecules using the previously described Michael-type addition reactions.^47,57^ To explore this potential, both [**1**] and [**2**] were synthesized using previously described chemical synthesis procedures (described in the Supporting Information).

**Figure 4:**
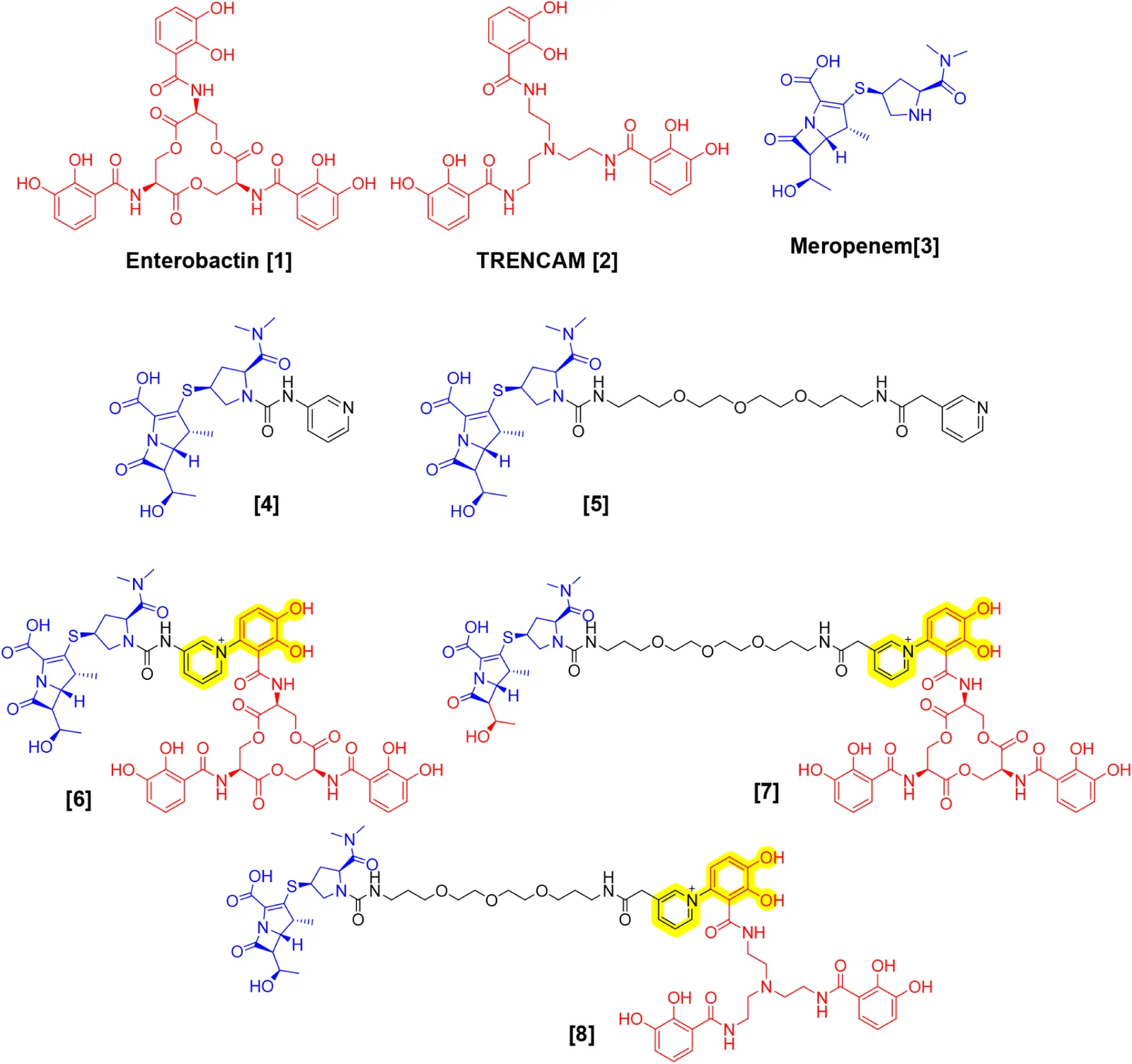
Chemical structures of tricatechol natural siderophore enterobactin [**1**], and synthetic siderophore TRENCAM [**2**], meropenem [**3**] and synthesized 3-pyridyl-substituted meropenem analogues [**4**] and [**5**], Ent-MEM [**6**] and Ent-PEG3-MEM [**7**] and the TRENCAM-PEG3-MEM [**8**] (see SI for details on chemical synthesis). Siderophores are drawn in red, antibiotics cargo in blue, linkers are in black and the site of catechol–pyridyl conjugation is highlighted in yellow.

As an initial proof of concept, it was decided to attach meropenem [**3**] as a cargo antibiotic to [**1**] and [**2**] using the silver-promoted iodine conjugation strategy. This choice exploits the periplasm-targeting properties of the siderophore vectors enterobactin and TRENCAM in *P. aeruginosa*.^56,58^ β-Lactam antibiotics were selected as a reasonable and pragmatic option in this context, as their antibacterial activity occurs in the periplasm.^59^ Among β-lactams, meropenem, a carbapenem, was chosen primarily for its superior stability against the chromosomally encoded AmpC β-lactamase,^60,61^ which limits the activity of previously reported siderophore−β-lactam conjugates such as aminopenicillins in *P. aeruginosa*.^43^ Additionally, meropenem is affected by both OprD porin loss and active efflux via MexAB-OprM,^62^ allowing assessment of whether the siderophore vectorization strategy can overcome multiple clinically relevant carbapenem resistance mechanisms. To enable conjugation to [**1**] and [**2**], a 3-pyridyl substituent needed to be introduced onto meropenem [**3**]. Structure–activity relationship studies (SARs) of carbapenems^63^ as well as previously reported siderophore conjugate^64^ indicated that meropenem’s secondary nitrogen was an optimal site for such a modification. With this rationale, two meropenem derivatives were prepared using solution-phase peptide coupling, as detailed in the Supporting Information (SI). For the first derivative, the 3-pyridyl substituent was directly introduced onto meropenem’s pyrrolidine nitrogen via a carbamate linkage, to give [**4**] (Figure 4). For the second derivative, the pyridyl group was appended through a PEG3 linker, analogous to the strategy previously reported by Nolan’s group,^64^ in which a stable PEG3 linker was used to provide a flexible and hydrophilic connection between the antibiotic cargo and the siderophore, while introducing sufficient distance to facilitate independent function of the two domains,^65^ yielding compound [**5**] (Figure 4).

To generate the desired siderophore–meropenem conjugates, meropenem derivatives [**4**] and [**5**] were coupled to siderophores [**1**] and [**2**] using silver-promoted iodine conjugation strategy (using silver hexanoate that was empirically found to prevent β-lactam ring opening) in DMSO at room temperature, following a procedure similar to the one previously reported^47^ (details of the synthesis in SI). These reactions afforded the pyridinium-functionalized enterobactin–meropenem conjugates (Ent-MEM [**6**], Ent-PEG3-MEM [**7**]) and TRENCAM–meropenem conjugate (TRENCAM-PEG3-MEM [**8**]) (Figure 4). The moderate yields obtained for these conjugates (15–33% for [**7**] and 12% for [**8**]) likely reflect the combination of several factors, including the formation of bis- and tris-functionalized conjugates, possible degradation of sensitive substrates such as β-lactam ring opening of [**5**], and/or competing nonspecific oxidative processes, side reactions such as oligomerization or polymerization of catechol-containing compounds. Optimization of the reaction conditions, including the choice of silver carboxylate and reagent stoichiometry, helped to minimize these competing processes; however, achieving high selectivity remains challenging due to the intrinsic reactivity of catechol oxidation and the sensitivity of the β-lactam moiety. Product conjugates were isolated by preparative reverse-phase HPLC, and their structures were confirmed by NMR spectroscopy (SI), which verified selective conjugation between the catechol in position C6 and the nitrogen atom of the pyridine ring, forming a positively charged pyridinium linkage. These results further validate our previously reported bioconjugation protocol in which the catechol moiety of the siderophore chlorodactyloferrin was exploited for conjugation. In line with the results obtained for the meropenem conjugates, yields after purification for [**10**] and [**11**] remained moderate (28% and 20%, respectively), mainly due to the formation of bis- and tris-functionalized conjugates.

### *In Vitro* Characterization of Antibacterial Activity of Siderophore–Meropenem Conjugates

To assess whether siderophore–meropenem conjugates retain target inhibition, the conjugates were first tested on purified *P. aeruginosa* penicillin-binding protein 3 (PBP3). This biochemical inhibition assay was achieved using a bocillin-FL-based fluorescence polarization (FP) assay^66,67^ and purified recombinant *P. aeruginosa* PBP3 that lacked the N-terminal hydrophobic transmembrane helix (34 amino acids) (Figure S2), as described previously.^68^ Using this assay, the meropenem conjugates [**7**] and [**8**] were found to have comparable PBP3 inhibition to meropenem [**3**], whereas the linker-free conjugate [**6**] was approximately 2–3-fold less potent (Table 1). These findings indicate that decoupling of meropenem from its siderophore conjugate is not required for effective inhibition of PBP3.

**Table 1:** Biochemical Inhibition of Recombinant *P. aeruginosa* PBP3 by MEM [**3**] and Siderophore–Meropenem Conjugates [**6**], [**7**], and [**8**]­^a^

Compound	Inhibition of Pa PBP3 IC_50_ (μM) (95% CI)
MEM [**3**]	1.36 (1.27 to 1.46)
Ent-MEM [**6**]	5.36 (5.12 to 5.62)
Ent-PEG3-MEM [**7**]	1.84 (1.76 to 1.91)
TRENCAM-PEG3-MEM [**8**]	1.79 (1.71 to 1.87)

a Assays were performed using a Bocillin-FL-based fluorescence polarization (FP) assay (curves in Figure S3) on 1 μM of purified Recombinant *P. aeruginosa* PBP3 that lacked the N-terminal hydrophobic transmembrane domain. Data are results from three independent assays.

To assess the antimicrobial activity of the siderophore–meropenem conjugates against *P. aeruginosa*, their minimum inhibitory concentrations (MICs) were determined using the broth microdilution assays. Assays were performed in a low-iron M9 medium^40^ (∼0.18 μM Fe^3+^ as measured by ICP-OES) or in the same medium supplemented with iron (10 μM FeCl_3_). Data showed that under iron-limited conditions, *P. aeruginosa* (PAO1) exhibited similar susceptibility to conjugates [**7**] and [**8**] as to MEM [**3**] itself, while the linker-free conjugate [**6**] was less active (Table 2). Importantly, upon iron supplementation, all three siderophore conjugates [**6**]–[**8**] lost antibacterial activity, in contrast to [**3**], which retained its efficacy, indicating an iron-dependent mechanism of action. In complementary experiments, the ability of siderophore–meropenem conjugates [**7**] and [**8**] to facilitate the bacterial uptake of ^57^Fe was evaluated by ICP-MS in *P. aeruginosa* FpvA FptA double knockout that is largely unable to take up the native siderophore pyoverdine and pyochelin. These ICP-MS data (Figure S4) confirmed that both conjugates promoted intracellular ^57^Fe accumulation. Together, these data support that these novel siderophore–meropenem conjugates can be internalized under iron-depleted conditions.

**Table 2:** Susceptibility of the Parental *P. aeruginosa* Strain (PAO1) and Indicated Isogenic Gene Deletion Strains to MEM [3], Ent-MEM [6], Ent-PEG3-MEM [7], and TRENCAM-PEG3-MEM [8] in Both Iron-Poor M9 Medium (M9) and in This Media Supplemented with 10 μM FeCl_3_ (M9 + Fe)^a^

		MIC (μM)							
		MEM [3]		Ent-MEM [6]		Ent-PEG_3_-MEM [7]		TRENCAM-PEG_3_-MEM [8]	
	Media**→**	M9	M9 + Fe	M9	M9 + Fe	M9	M9 + Fe	M9	M9 + Fe
*P. aeruginosa* strain	PAO1	0.5	0.5	16	>32	1	16	0.5	16
	Δ*pfeA*	0.5	0.5	>32	>32	>32	>32	16	>32
	Δ*pirA*	0.5	0.5	16	>32	2	>32	16	>32
	Δ*fpvA*	0.5	0.5	0.5	32	0.25	16	0.5	16
	Δ*fptA*	0.5	0.5	16	>32	4	32	0.5	32
	Δ*oprD*	16	8	16	>32	1	32	0.5	32
	Δ*mexR*	4	2	16	>32	1	32	0.5	32
	Δ*pfeA* Δ*pirA*	0.25	0.25	n.d.	n.d.	>32	>32	16	>32

a MIC values were determined from two independent experiments; the highest value is reported.

To identify the TonB-dependent outer-membrane transporter (TBDT) implicated in the uptake of the siderophore–meropenem conjugates, genes encoding TBDTs responsible for the uptake of endogenous siderophores, pyoverdine (*fpvA*) and pyochelin (*fptA*), as well as the uptake of xenosiderophores such as enterobactin (*pfeA* and *pirA*)^58^ were knocked out in PAO1. While the isogenic mutants were found to have an unaltered susceptibility to MEM [**3**] (Table 2), deletion of *pfeA* conferred resistance to conjugates [**6**]–[**8**], while deletion of *pirA* also affected the activity of the TRENCAM–meropenem conjugate [**8],** linking both TBDTs to the active internalization of the conjugates. The *pfeA* and *pirA* double deletion mutant was not found to be more resistant to TRENCAM–meropenem conjugate [**8**] compared to the single mutants (Table 2), which could suggest that at this concentration (MIC = 16 μM), activity may be because of alternative mechanism of low-efficiency entry. As a note, enterobactin [**1**] and TRENCAM [**2**] alone had no impact on bacterial growth of the wild-type or isogenic mutants up to 32 μM, suggesting that the siderophores themselves do not cause iron starvation in these M9 culture conditions. In addition, the data showed that deletion of *fptA* caused slight resistance to conjugate [**7**] (Table 2), which may implicate this TBDT in transport of [7] but may also be explained by indirect effects as a result of the transporter knockout. As an importer of the primary siderophore pyoverdine, deletion of *fpvA* resulted in sensitization of the bacteria to conjugates [**6**]–[**8**], which likely is because these bacteria can no longer take up pyoverdine, leading to increased iron stress and induction of auxiliary TBDTs to internalize conjugates [**6**]–[**8**]. In the case of [**6**], which was considerably less active than conjugates [**7**] and [**8**] against PAO1 (Table 2), despite its slightly lower affinity for PBP3 (Table 1), deletion of *fpvA* restored its activity to a level comparable to that observed for [**7**] and [**8**]. This observation suggests that [**6**] cannot efficiently compete with pyoverdine for iron acquisition, supporting the importance of the linker in preserving the iron-binding properties of the siderophore in its conjugated form and enabling subsequent internalization. Together, these data support that the described enterobactin and TRENCAM conjugates are taken up by TBDTs, particularly by PfeA.

As the loss of the OprD porin, or an increase in active efflux decreases susceptibility to meropenem,^62^ we next aimed to evaluate whether the siderophore–meropenem conjugates could overcome this common mechanism of resistance. To assess this, isogenic PAO1 deletion strains of *oprD* and the transcription repressor of the MexAB-OprM efflux pump, *mexR,* were constructed. As expected, both deletions reduced the susceptibility of PAO1 to meropenem. Encouragingly, neither of these mutations affected the antibiotic activity of siderophore–meropenem conjugates [**6**]–[**8**] (Table 2), confirming their porin-independent, TBDT-mediated uptake, and demonstrating that such conjugates can overcome meropenem resistance by porin loss. In addition, the siderophore conjugates appeared to no longer be good MexAB-OprM efflux substrates. Finally, the Ent-PEG3-MEM [**7**] was also shown to display *in vitro* efficacy on clinical CRPA MDR *P. aeruginosa* isolates in combination with metallo β-lactamase inhibitors, as is presented in sections below. These findings demonstrate how facilitating antibiotic uptake into bacteria can help overcome clinically important antibiotic resistance mediated by porin loss and active efflux.

### Siderophore Conjugates of Indole Carboxylate-Based Metallo β-Lactamases Inhibitors

While siderophore–meropenem conjugates effectively circumvented *P. aeruginosa* meropenem resistance mediated by porin loss and overexpression of efflux, evaluation of [**7**] against metallo-β-lactamase (NDM-1)-expressing clinical isolates of *P. aeruginosa* found them to be inactive (Table S3), likely because these conjugates are still degraded by these enzymes. With the objective of designing siderophore conjugates able to target such priority carbapenemase-producing *P. aeruginosa*, we turned our attention to a series of potent broad-spectrum metallo-β-lactamase inhibitors (MBLi), the indole-carboxylate-based MBL inhibitors (InCs) described as highly effective on MBL-producing Enterobacterales but not MBL-producing *P. aeruginosa*, likely due to their inability to effectively enter the periplasm.^16^

To design the indole carboxylate-based (InC) MBL inhibitors amenable to catechol-pyridyl conjugation, we employed a strategy based on the available structure–activity relationship data on InC-based MBL inhibitors^16^ and the insights gained from the siderophore–meropenem conjugates described above. By drawing parallels to the parent inhibitor scaffold, a triazole-substituted indole-2-carboxylate (compound 48 in^16^), we introduced a urea group in place of the triazole moiety, enabling its attachment via the PEG3-based linker (analogous to [**7**]) bearing a terminal 3-pyridyl group, thereby affording compound [**9**] (Figure 5, see details in SI). Compound [**9**] was subsequently conjugated to both enterobactin [**1**] and TRENCAM [**2**], yielding the siderophore–MBL inhibitor conjugates [**10**] and [**11**], respectively (Figure 5)

**Figure 5:**
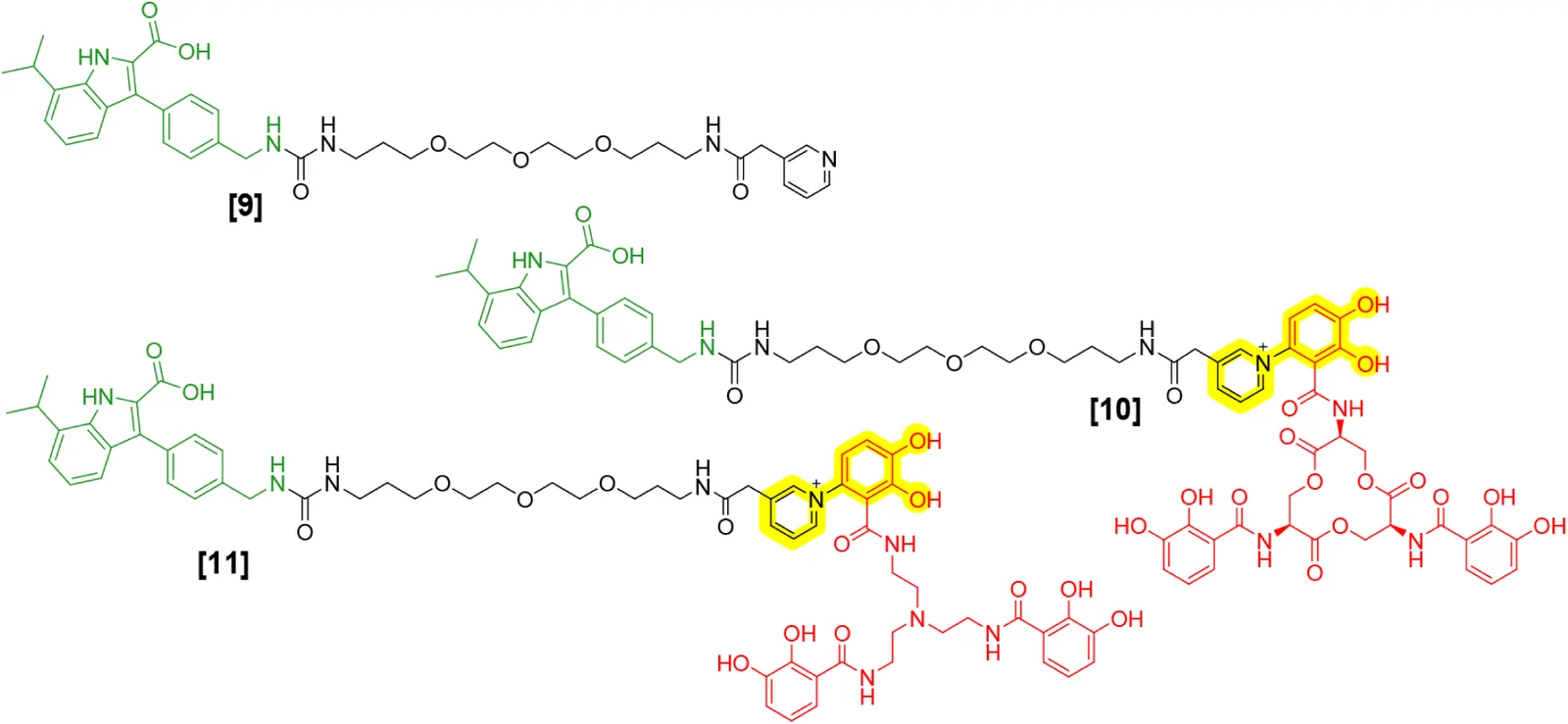
Chemical structures pyridine-PEG3-modified indole carboxylate-based MBL inhibitors [**9**], its enterobactin-PEG3-MBLi [**10**] and TRENCAM-PEG3-MBLi [**11**] derivatives. Siderophores are shown in red, MBLi cargo in green, linkers are in black, and the catechol–pyridyl conjugation sites are highlighted in yellow.

### *In Vitro* Characterization of Siderophore–MBL Inhibitor Conjugates

As with the siderophore–meropenem conjugates, we assess whether the generated siderophore–MBL inhibitor conjugates [**10**] and [**11**] retain on-target biochemical inhibition of the metallo-β-lactamase NDM-1 using a nitrocefin hydrolysis assay. Results revealed that all tested compounds inhibited NDM-1 in the nanomolar range (Table 3), with conjugates [**10**] and [**11**] showing a similar inhibitory activity to that of the parental inhibitor [**28**]. These results clearly indicate that the metallo-β-lactamase conjugates [**10**] and [**11**] are active by themselves and do not require any decoupling process to be active. In addition to this, when incubating *P. aeruginosa* PAO1 (growing in CAMHB or ID-CAMHB) with conjugates [**10**] or [**11**], analytical mass spectrometry (UHPLC-MS) in the bacterial pellet of PAO1 growing in ID-CAMHB found no evidence of compound degradation/metabolism products after 4 h incubation (Figure S6). Similarly, conjugates [**10**] or [**11**] were found to be largely stable in spent media, with more than 75% recovery after 24 h (Figure S7). Finally, as with siderophore–meropenem conjugates, ^57^Fe uptake assay using ICP-MS (as in ref. 40) with conjugates [**10**] and [**11**] showed that these conjugates promoted a similar intracellular ^57^Fe accumulation in the *P. aeruginosa* FpvA-FptA double-knockout strain (where pyoverdine uptake is largely blocked) (Figure S4). Altogether, this information points to the active uptake of conjugates [**10**] or [**11**] into *P. aeruginosa* in iron-depleted conditions and that the unmodified conjugates would be able to effectively inhibit NDM-1 without the need for decoupling.

**Table 3:** IC_50_ Values of MBL Inhibitor Conjugates against Recombinant NDM-1. Inhibitory Activity Was Evaluated by Monitoring Nitrocefin Hydrolysis in the Presence of Recombinant NDM-1^a^

Compound	Inhibition of NDM-1 IC_50_ (nM) (95% CI)
MBLi [**28**]	59 (43 to 82)
MBLi-PEG3-Pyr [**9**]	127 (98 to 171)
Ent-PEG3-MBLi [**10**]	53 (36 to 78)
TRENCAM-PEG3-MBLi [**11**]	87 (79 to 98)

a IC_50_ values are expressed in nM with 95% confidence intervals and were calculated from three independent experiments. Dose-response curves from which IC_50_ data were extracted are in Figure S5.

### TBDT-Dependent Siderophore–MBL Inhibitor Conjugate Uptake in *P. aeruginosa*

To assess TBDT uptake of the siderophore-MBL inhibitor conjugates into *P. aeruginosa*, we determined the accumulation of siderophore-MBLi conjugates [**10**] and [**11**] by analytical UHPLC-MS on PAO1 growing in high- and low-iron conditions, and in isogenic mutants lacking specific TBDTs identified to be important for siderophore-MEM conjugate uptake (PAO1, PAO1*ΔpfeA*, PAO1*ΔpirA* and PAO1*ΔpfeA ΔpirA*). Data (Figure 6) revealed that for both [**10**] and [**11**], accumulation into *P. aeruginosa* was found to be markedly increased in low-iron conditions, supporting iron-stress-dependent uptake expected of siderophore conjugates. Analysis of the TBDT isogenic mutants in low-iron medium confirmed that accumulation of both conjugates was greatly reduced in PAO1*ΔpfeA* compared to the parental strain, while deletion of pirA has a small/negligible impact on [**10**] and [**11**] uptake. In the double TBDT mutant (PAO1*ΔpfeA ΔpirA*) accumulation of both conjugates was even lower and resembled that of high-iron medium conditions. Together, these data support that both siderophore-MBLi conjugates [**10**] and [**11**] are preferentially taken up by *P. aeruginosa* in low-iron conditions, primarily through the TBDT PfeA with a minor residual contribution of PirA.

**Figure 6:**
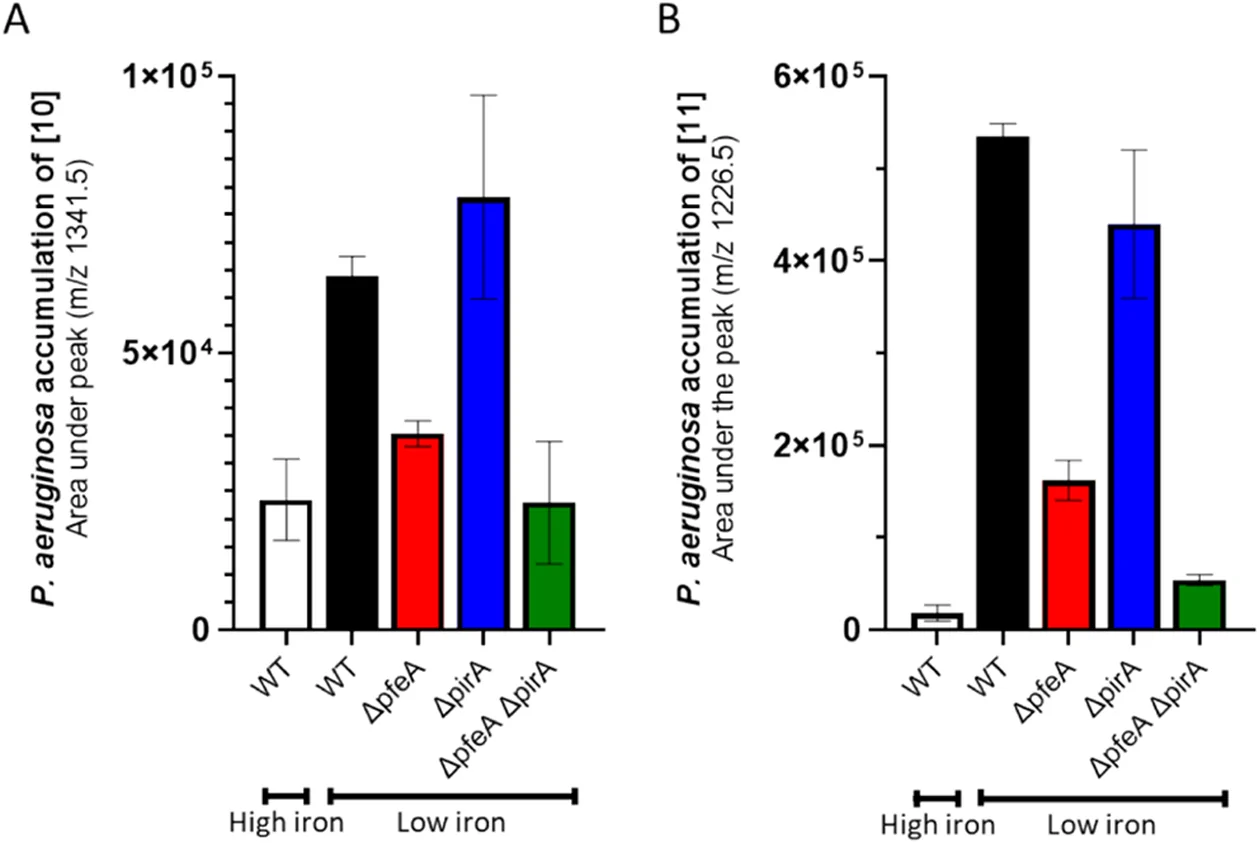
Accumulation of [**10**] and [**11**] in *P. aeruginosa* PAO1 and isogenic mutants. The accumulation of (A) enterobactin–MBLi conjugate [**10**] and (B) TRENCAM–MBLi conjugate [**11**] were measured by analytical UHLPC-MS in wild-type PAO1 (WT) or indicated isogenic mutants in both high-iron (CAMHB) and low-iron (ID-CAMHB) media. Bacterial cells were incubated with 100 μM of each conjugate for 1 h at 37 °C before analysis by UHPLC-MS using an Ultimate 3000 UHPLC system coupled to an LCQ Fleet Ion Trap Mass Spectrometer (Thermo Scientific). Compound quantification is based on the area under the extracted ion chromatogram (EIC) peak corresponding to the singly charged ions of [**10**] (*m*/*z* 1226.54) or [**11**] (*m*/*z* 1341.48). Similar data were observed when integrating the corresponding UV peak (305 nm). Results are presented as the mean ± SD of three independent biological replicates.

### Siderophore–MBL Conjugates to Overcome Antibiotic Resistance in Clinical MBL-Producing CRPA Isolates

To evaluate the activity of siderophore–MBLi conjugates [**10**] and [**11**], a set of MDR clinical *P. aeruginosa* isolates producing MBLs, NDM, VIM, and IMP were selected from the French National Reference Center for *P. aeruginosa* collection (detailed genotypes of strains can be found in Table S1). As a readout of carbapenemase inhibition, it was initially decided to investigate the ability of siderophore–MBLi conjugates to potentiate the *in vitro* activity of meropenem. In line with EUCAST guidelines for cefiderocol susceptibility testing of clinical *P. aeruginosa* strains (which has iron-dependent activity), these assays were performed in iron-depleted cation-adjusted Mueller–Hinton broth (ID-CAMHB).^69^ In this medium, enterobactin [**1**] and enterobactin–MBLi conjugates [**10**] alone did not show any impact on bacterial growth (tested up to 64 μM and 16 μM, respectively); however, both TRENCAM [**2**] and TRENCAM–MBLi conjugates [**11**] were found to inhibit *P. aeruginosa* growth at concentrations above 1 μM (this was not observed in low-iron M9 medium up to 32 μM). This observation was confirmed by following the growth of *P. aeruginosa* in ID-CAMHB in the presence of a concentration range of enterobactin [**1**] and TRENCAM [**2**] (Figure S8). This TRENCAM [**2**]-associated activity on *P. aeruginosa* is likely due to the inability of the bacterium to release iron from TRENCAM [**2**], and that pyoverdine is unable to effectively compete with this siderophore in such low-iron conditions. Together, this finding limited the analysis of TRENCAM–MBLi [**11**] to concentrations below 1 μM. In additional control experiments, it was also confirmed that the nonconjugated pyridine-modified MBL inhibitor [**9**] itself was unable to boost meropenem activity against NDM-1-producing clinical isolates of *P. aeruginosa* with a wild-type OprD porin (isolates Pa 2311260 and Pa 2412170, Table S2), in line with data reported on other indole-carboxylate-based MBL inhibitors.^16^

Checkerboard assays on the MBL-expressing clinical strains of MDR *P. aeruginosa* in ID-CAMHB found that enterobactin–MBLi conjugate [**10**] was a potent potentiator of meropenem activity in bacteria expressing a metallo-β-lactamase (NDM-1, VIM, or IMP), and where OprD was not mutated (allowing meropenem to enter the periplasm) (Table 4). While the potency of [**10**] was strain dependent, likely due to differences in intrinsic baseline resistance to meropenem, [**10**] was generally able to enhance susceptibility to meropenem to below its clinical resistance breakpoint (>21 μM or >8 mg/L, EUCAST 2026 v.16.0) at concentrations around 1 μM (Table 4). It was also noted that [**10**] was able to boost meropenem in the VIM-expressing clinical isolates (strain Pa 2411595 and Pa 2412054), but not in a VIM-expressing clinical isolate with an additional mutation in the TBDT, PfeA (strain Pa 2512749), supporting the role of PfeA as a TBDT that mediated the uptake of this conjugate (Table 4).^16^ Finally, in clinical strains carrying mutations in *oprD*, [**10**]-meropenem combinations were ineffective, as meropenem itself was likely unable to penetrate the bacteria (Table 4). To confirm the role of *oprD* mutations in meropenem resistance, the enterobactin–MBL conjugate [**10**] was tested in combination with the enterobactin–meropenem conjugate [**7**] on three *oprD*-deficient NDM-expressing clinical strains where meropenem was not boosted, and found to restore the antibiotic activity (Table 4). Finally, [**10**] was found to also potentiate the activity of meropenem in a clinical MDR strain of *A. baumannii* expressing NDM-1 (Table 4). Notably, *A. baumannii* is able to exploit enterobactin as a xenosiderophore, despite lacking its own biosynthetic pathway. This capacity relies on recognition and uptake of ferric enterobactin via the TBDT FepA homologue^70,71^ and whole-genome analysis of the NDM-1-producing *A. baumannii* isolate (CIP7010) confirmed it to carry *fepA*. Altogether, these findings suggest that such vectorized inhibitors can also be useful for other MDR pathogens.

**Table 4:** *In Vitro* Activity of MEM [**3**]/Ent-PEG3-MEM [**7**] in Combination with Ent-PEG3-MBLi [**10**] or TRENCAM-PEG3-MBLi [**11**] against a Panel of MBL-Producing Clinical Isolates of *P. aeruginosa* and an NDM-1-Producing Clinical Isolate of *A. baumannii* *(Ab)* ^a^

						MIC (μM) of MEM [3] or Ent-PEG3-MEM [7] in ID-CAMHB			
Strain		MBLs	other β-lactamase	OprD porin	TBDT mutations	MEM [3]	MEM [3] + Ent-PEG3-MBLi [10]	MEM [3] + Tre-PEG3-MBLi [11]	Ent-PEG3-MEM [7] + Ent-PEG3-MBLi [10]
P. aeruginosa	2311260	NDM-1		wt			0.5	32	n.d.
	2512892	NDM-1		wt		>64	4	>64	n.d.
	2412091	NDM-1		wt		>64	8	>64	n.d.
	2512537	NDM-1		wt		>64	8	64	n.d.
	2412170	NDM-1		wt		>64	8	64	n.d.
	2513161	NDM-1		wt		>64	32	>64	n.d.
	2411694	NDM-1		mut^b^		>64	64	>64	1
	2411726	NDM-1		mut^c^		>64	64	>64	2
	2311286	NDM-1	LCR-1	mut^d^	*piuA* ^c^	>64	32	>64	1
	2412054	VIM-2		wt		>64	16	64	n.d.
	2411595	VIM-2	PER-1 OXA-4	wt	*piuA* ^c^	>64	16	>64	n.d.
	2512749	VIM-4		wt	*pfeA* ^e^ *foxA* ^f^	>64	64	>64	n.d.
	196883	IMP-1		wt	*fiuA* ^g^	>64	4	32	n.d.
	2412280	IMP-19		wt	*piuA* ^c^	>64	8	32	n.d.
***Ab***	CIP7010	NDM-1				>64	2	32	n.d.

a The values shown represent the lowest MIC of MEM [3] or Ent-PEG3-MEM [7] achieved within the tested MBL inhibitor concentration ranges (0.03 to 16 μM for Ent-PEG3-MBLi [10] and 0.03 to 1 μM for TRENCAM-PEG3-MBLi [11]). Complete checkerboard datasets for each inhibitor concentration are provided in the Supplementary Information (Tables S3 and S4). The MEM resistant breakpoint is >21 μM (>8 mg/L) according to EUCAST 2026 v.16.0. Data correspond to the higher value from two independent experimental replicates.

b Premature stop codon (Q402X).

c Truncated gene.

d Missense substitution (M372 V).

e Missense substitution (V703I).

f Missense substitution (P171L).

g Missense substitution (T152P), n.d., not determined.

In parallel with [**10**], the potency of the TRENCAM–MBL inhibitor conjugate [**11**] was evaluated on the panel of clinical *Pseudomonas* strains. As TRENCAM showed intrinsic antibiotic activity on *P. aeruginosa* in ID-CAMHB, this evaluation was limited to concentrations below ≤1 μM (subantibiotic). Checkerboard combination studies at these concentrations showed that [**11**] could slightly improve the MIC of MEM [**3**] from >64 μM to 32 μM in some MBL-expressing clinical strains of MDR *P. aeruginosa* (expressing NDM-1, VIM, or IMP with a functional OprD porin), although to a lesser extent than observed with similar concentrations of [**10**] (Table 4).

As the expression of MBLs (especially NDM-1) in clinical strains of *P. aeruginosa* is frequently associated with mutations in *oprD*,^72,73^ this largely limited the association of siderophore–MBL inhibitor conjugates with meropenem. To overcome the impact of oprD mutations, it was decided to next investigate the activity of siderophore–MBL inhibitor conjugates [**10**] and [**11**] on cefiderocol activity. While lacking a native siderophore backbone, cefiderocol instead carries a monocatechol group that enables uptake via TBDTs (PiuA/D, PirA, and PiuC),^74^ and is thus not impacted by OprD mutations. On the other hand, expression of NDM-1, but not other MBLs, is a major mechanism of cefiderocol resistance.^75^

Checkerboard assay results (Table 5) showed that both the enterobactin- and TRENCAM-MBL inhibitor conjugates [**10**] and [**11**] were able to reverse cefiderocol resistance in NDM-1-expressing clinical strains of *P. aeruginosa* irrespective of the OprD genotype, in stark contrast to meropenem. Although the strains displayed some differences, [**10**] and [**11**] generally appeared to be very potent, with nanomolar concentrations being sufficient to substantially reduce the MIC of cefiderocol across the tested isolates. While both siderophore conjugates were able to revert cefiderocol activity in these NDM strains, the activity of the enterobactin conjugate appeared superior and was able to decrease the MIC to a lower level for the majority of strains. At higher concentrations of [**10**], the siderophore conjugates were found to antagonize the activity of cefiderocol (Table S5), likely as a result of competition for the low levels of iron in the media. Finally, it was noted that the enterobactin- and TRENCAM-MBL inhibitor conjugates had a comparable activity when combined with cefiderocol (a similar activity was also observed earlier with enterobactin and TRENCAM meropenem conjugates), while this was not the case in combination with meropenem [3]. Currently, we do not have a clear explanation for this discrepancy, though it may be related to *in vitro* culture conditions, as TRENCAM-PEG3-MBLi [11] did appear efficacious in combination with meropenem in *in vivo* infection studies presented hereafter.

**Table 5:** Potentiation of CFD by Ent-PEG3-MBLi [**10**] or TRENCAM-PEG3-MBLi [**11**] against NDM-1 Expressing Clinical Isolates of *P. aeruginosa* ^a^

Strain		MBLs	other β-lactamase	OprD porin	TBDT mutations	MIC (μM) of CFD in ID-CAMHB		
						CFD	CFD + Ent-PEG3-MBLi [10]	CFD + Tre-PEG3-MBLi [11]
*P. aeruginosa*	2311260	NDM-1		wt		8	2	4
	2512892	NDM-1		wt		0.5	<0.03	0.06
	2412091	NDM-1		wt		2	<0.03	0.25
	2512537	NDM-1		wt		1	0.06	0.12
	2412170	NDM-1		wt		1	<0.03	0.06
	2513161	NDM-1		wt		1	0.06	0.12
	2411694	NDM-1		mut^b^		8	0.25	0.5
	2411726	NDM-1		mut^c^		4	0.12	0.25
	2311286	NDM-1	LCR-1	mut^d^	*piuA* ^c^	8	4	2

a Values represent the lowest MIC of CFD observed within the tested concentrations of the MBL inhibitors (0.03 to 1 μM). Complete checkerboard results can be found in the Supplementary Information (Table S5). The CFD clinical breakpoint is >2.7 μM or >2 mg/L according to EUCAST 2026 v.16.0. Data represent the higher value from two independent experiments .

b Premature stop codon (Q402X).

c Truncated gene.

d Missense substitution (M372V).

### Siderophore–MBL Inhibitor Conjugates Demonstrate *In Vivo* Efficacy in a *Galleria mellonella* Infection Model

While the *in vitro* experiments yielded encouraging results, it remained unclear how these results generated in low-iron media would translate to *in vivo* activity, where the degree of iron stress may be different. To address this, it was decided to explore siderophore-conjugate efficacy in *Galleria mellonella* larvae infected with *P. aeruginosa*. Infections of *G. mellonella* have been established as an attractive alternative infection model for several clinically important bacterial pathogens, including members of the ESKAPE group,^76–81^ and as a useful platform for evaluating novel antimicrobial agents. Critically, it has also been shown that *G. mellonella* imposes iron-limiting conditions on the infection, making it an ideal model for studying siderophore conjugates.^82^

Prior to infection studies, it was confirmed that the siderophores and siderophore conjugates displayed no *in vitro* cytotoxicity at 100 μM against HepG2 cells (Table S6). Next, a number of NDM-producing *P. aeruginosa* isolates were evaluated for their ability to infect and colonize *G. mellonella* (test for isolate virulence), identifying three NDM-producing isolates (Pa 2311260, Pa 2512892, and Pa 2412091) whose infection caused larval death within 24 h (Table S7). To evaluate the efficacy of the siderophore-MBL inhibitor conjugates in this model, larvae were infected with 20 CFU of *P. aeruginosa*, followed by the injection 2 h postinfection of meropenem or cefiderocol alone or in combination with [**10**] or [**11**], with subsequent monitoring of larval survival. Treatment of larvae with 10.7 mg kg^–1^ of [**10**] or 9.8 mg kg^–1^ [**11**], which corresponds to a predicted larval body concentration of 8 μM, did not have a significant impact on the survival of *P. aeruginosa*-infected larvae. Similarly, administration of 20 mg kg^–1^ of meropenem or 20 mg kg^–1^ of cefiderocol alone (predicted larval body concentrations of 52 μM and 26 μM, respectively) did not significantly improve infected larvae survival (Figure 7). In contrast, coadministration meropenem or cefiderocol with [**10**] resulted in a significant marked improvement in larval survival for infections by all three NDM-1-producing *P. aeruginosa* isolates tested (Figure 7). Similarly, coadministration of meropenem or cefiderocol with [**11**] also significantly protected the larvae from *P. aeruginosa* infections, though it was less pronounced than observed with [**10**] (Figure 7). Overall, the extent and order of meropenem or cefiderocol efficacy potentiation by [**10**] and [**11**] seen in *G. mellonella* infection model was remarkably consistent with the *in vitro* activity results in ID-CAMHB. Collectively, these *in vivo* results support the potential of the MBL inhibitor conjugates under pathophysiological iron conditions and against clinically relevant MBL-producing *P. aeruginosa* isolates.

**Figure 7:**
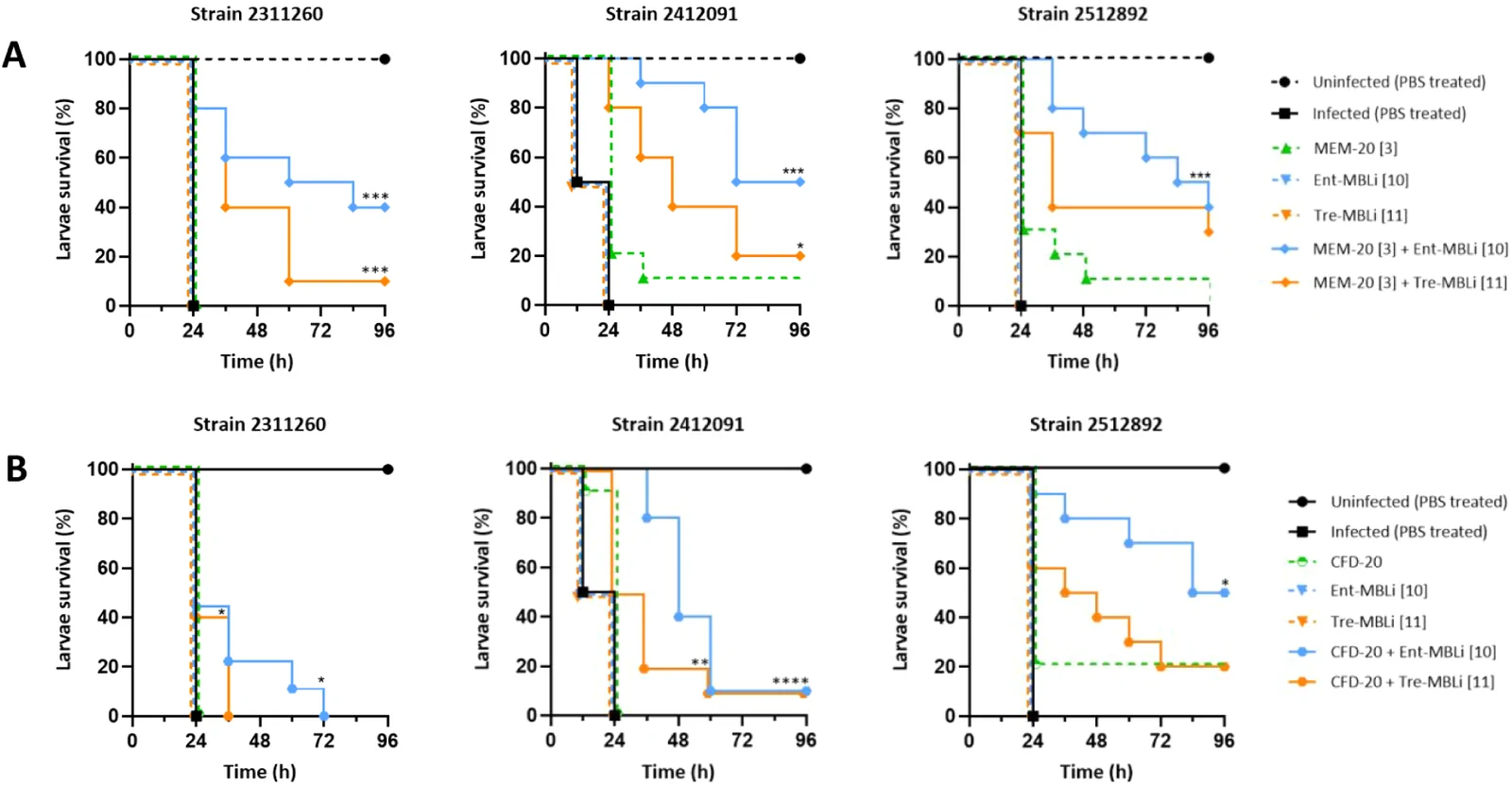
*In vivo* efficacy of MBL inhibitor conjugates in restoring the activity of MEM and CFD in the *G. mellonella* infection model. Groups of ten larvae were infected with 10 μL of bacterial suspension of three NDM-1-producing strains in PBS (2 × 10^3^ CFU/mL). Two hours postinfection, larvae received a single 10 μL dose of (**A**) 20 mg/kg MEM or (**B**) 20 mg/kg CFD alone or in combination with inhibitor conjugates (10.7 and 9.8 mg/kg for [**10**] and [**11**], respectively). Larval survival was monitored every 12 h for up to 96 h. Asterisks indicate groups with significantly enhanced survival compared with the group treated with antibiotic alone, as determined by the Gehan–Breslow–Wilcoxon test (*p < 0.05, **p < 0.01, ***p < 0.001, ****p < 0.0001). Detailed *p* values are provided in the Supporting Information (Table S8).

## Discussion

*P. aeruginosa* is a major global pathogen, and carbapenem-resistant *P. aeruginosa* has been classified by the World Health Organization as a high-priority pathogen.^5^ While nonenzymatic mechanisms of carbapenem resistance are common in *P. aeruginosa*, the emergence of carbapenemase-producing strains has resulted in more severe resistance profiles, with patients infected by these strains showing higher 30-day mortality than those with noncarbapenemase-producing CRPA.^9^

Research over the last decades has clearly demonstrated that siderophores can enhance the penetration of certain conjugated antibiotic payloads, which, when applied to *P. aeruginosa* have mostly targeted periplasmic proteins.^21,65,83^ Siderophores used for antibiotic delivery must both outcompete native siderophores for iron binding and exhibit high affinity for outer membrane transporters to ensure uptake.^84^ Enterobactin and TRENCAM are particularly attractive siderophores, as their exceptionally high affinities for iron (*Ka* ≈ 10^49^ M^–1^ and *Ka* ≈ 10^43^ M^–1^, respectively^85^), can compete with that of the native siderophore pyoverdine (*Ka* ≈ 10^31^ M^–1^)^85^ in *P. aeruginosa*. It has indeed previously been shown that enterobactin uptake by *P. aeruginosa* is remarkably efficient, transporting up to 2,000 iron atoms per bacterium per minute under iron-limiting conditions *via* the PfeA TBDT transporter.^84^ While tris-catecholate siderophores have favorable properties as vectors for antibiotic conjugates, it should be noted that thus far no true siderophore-antibiotic conjugate has been developed as a drug. Such conjugates will likely need further optimization to improve drug-like properties, with previous work suggesting liabilities such as enzymatic cleavage of enterobactin ester bonds (not an issue for TRENCAM),^40^ oxidation or methylation of the catechol moieties themselves, and potential sequestration by lipocalin-2 (highlighted in^18^). Nonetheless, native tris-catecholate siderophores remain a pertinent vector for proof-of-concept efficacy studies, with resulting bioactive scaffolds ready for further medicinal chemistry optimization.

With respect to enterobactin and TRENCAM, successful conjugates have thus far been generated by mimicking the glycosylation site of salmochelin, where cargo is attached on the C5 catechol position.^31^ In this work, we demonstrate that payload coupling to the C6 catechol position of the siderophore using a recently reported Michael-type addition, in which the cargo 3-pyridyl group reacts with the catechol moiety of the siderophore,^47^ can similarly lead to bioactive conjugates that are actively taken up by *P. aeruginosa* through the PfeA TBDT system. Although this strategy has thus far only been employed in bioconjugation with chlorodactyloferrin,^47^ we now report its broader applicability to other catechol-containing siderophores such as enterobactin and TRENCAM. An interesting advantage of this strategy is that it can be performed on the native, unmodified siderophores, even though yields are not always optimal and can be complicated for siderophores with multiple catechols. Mechanistic studies performed here demonstrate that this iodine-mediated catechol–pyridine conjugation reaction can alternatively also proceed through a silver­(I) salt-promoted formation of the iodonium-pyridine salt that can itself oxidize the catechol as well as take part as a substrate in the Michael-type addition reaction. Taken together, these findings highlight the uniqueness of this bioconjugation approach, which, despite its facility and operational simplicity, has not yet been actively employed for assembling this class of molecules.

In addition to the siderophore vector, the antimicrobial warhead to be attached must also be carefully considered, particularly if the objective is to have antimicrobial activity on a priority bacterial pathogen with diverse existing mechanisms of antibiotic resistance. Important considerations for this warhead include that 1) outer-membrane penetration represents a primary barrier to efficacy, 2) it is not too big as certain limitations related to cargo size exist,^31^ 3) the warhead activity is not impacted by existing mechanisms of antibiotic resistance in the target bacterium (such as target mutations or drug-inactivating enzymes), 4) whether the warhead can act as a siderophore conjugate or requires decoupling. In this study, in line with the periplasmic destination of tris-catecholate siderophores such as enterobactin in *P. aeruginosa*,^58^ which do not cross the inner membrane into the cytosol, the conjugated cargo must be active in the periplasm.^18^ Based on this rationale, we selected meropenem as the cargo antibiotic, a last-resort antipseudomonal carbapenem,^49^ whose PBP3 target is situated in the periplasm. Using this approach, we showed that by redirecting meropenem uptake into the periplasm *via* active transport through the PfeA transporter, siderophore–meropenem conjugates ([**6**], **[7**], and [**8**]) were able to overcome clinically relevant meropenem resistance in *P. aeruginosa* mediated by the loss of the OprD porin or by efflux.^62^ While these results with siderophore–meropenem conjugates are noteworthy, the selection of the meropenem warhead did not fulfill all the criteria for a warhead targeting priority pathogens such as CRPA, as it was still subject to degradation by carbapenemases (it was inactive in MBL-expressing CRPA).

Carbapenem resistance mediated by metallo-β-lactamases (MBLs) remains a major challenge that leads to the inactivation of carbapenems regardless of their route of entry. To date, it remains challenging to develop MBL inhibitors that are effective in *P. aeruginosa*. Bicyclic boronates, such as taniborbactam and xeruborbactam, have expanded coverage against some MBLs; however, taniborbactam is inactive against IMP-type MBLs, and xeruborbactam, while broader in spectrum, fails to restore susceptibility in *P. aeruginosa* due to efflux-mediated resistance.^86^ Recently described indole carboxylate-based MBL inhibitors represent a promising new class of potent inhibitors active against *Enterobacterales*, but inactive in *Pseudomonas*, likely due to poor outer membrane penetration,^16^ making them an ideal candidate for attachment to siderophores. The extensive published structure–activity relationship data^16^ around the indole carboxylate-based MBL inhibitors facilitated the design of pyridyl-containing analogs used for coupling to enterobactin and TRENCAM, and based on the findings from enterobactin–meropenem conjugates, the indole carboxylate scaffold was linked to the siderophore *via* a PEG3 linker. Encouragingly, these tris-catecholate siderophore–MBL inhibitor conjugates showed remarkably potent ability to revert resistance to meropenem and cefiderocol in a panel of MBL (NDM, VIM, and IMP)-expressing CPRA, validating that access to the periplasm was a fundamental barrier to this inhibitor series, and highlighting how introducing active uptake of the molecule can restore activity. These data also highlighted that in the context of meropenem, for which there can be multiple mechanisms of resistance in CRPA isolates, inhibition of NDM-1 with siderophore-MBL inhibitor conjugates cannot overcome additional resistance by loss of porins that prevent meropenem entry. Encouragingly, however, in the context of cefiderocol that enters the periplasm by active uptake, porin mutations do not alter the efficacy of siderophore-indole carboxylate MBL inhibitor conjugates, though rare mutations in cefiderocol uptake machinery do. Overall, this work highlights how siderophore conjugates can be successfully designed and synthesized to target the priority threat of CRPA and help overcome resistance to this last-resort antibiotic.

Finally, while many siderophore conjugates have been shown to act as Trojan horse molecules *in vitro*,^18^ it was not certain that this activity would be maintained *in vivo*. In particular, the extent to which the iron stress experienced *in vitro* in low-iron media recapitulates the infection setting is uncertain, as iron *in vivo* is present but sequestered by host iron-binding proteins.^58^ Indeed, even between different low-iron media (such as low-iron M9 and ID-CAMHB) the activity of cefiderocol varied, while we also observed that TRENCAM itself prevented *P. aeruginosa* growth in ID-CAMHB, but to a lesser extent in low-iron M9. Considering this important variable, it was reassuring to confirm the activity of enterobactin– and TRENCAM–MBL inhibitor conjugates [**10**] and [**11**] in *G. mellonella* infected with NDM-1 carbapenemase-expressing clinical isolates of *P. aeruginosa*. Indeed, when coadministered with either meropenem or cefiderocol, the potency of [**10**] and [**11**] was comparable to that observed *in vitro* in ID-CAMHB. In addition, it was notable that TRENCAM and TRENCAM-MBLi conjugate [**11**] alone showed no activity against *P. aeruginosa* in infected *G. mellonella*, while this was seen in artificial low-iron media used in the *in vitro* studies, and this may be explained by the greater abundance of “bound” iron in the infection model, which would prevent TRENCAM-mediated iron starvation. Together, these results validate that the *in vitro* efficacy of siderophore conjugates can be reproduced *in vivo*, providing an important validation step for the potential of Trojan horse siderophore conjugates.

Together, the strategy presented provides an important advance in the design of siderophore conjugates for targeting multidrug-resistant *P. aeruginosa* infections. We show that siderophore-mediated vectorization of the indole carboxylate derivative restored the activity of this highly potent inhibitor against metallo-β-lactamase-producing *P. aeruginosa*, for which there are otherwise few therapeutic options.^49^ Overall, these findings highlight the potential of siderophore-mediated transport as a versatile strategy for efficient delivery of therapeutic payloads, such as MBL inhibitors, into Gram-negative pathogens, thereby reversing resistance to last-line antibiotics such as carbapenems and cefiderocol.

## Materials and Methods

### Synthetic Chemistry

The chemical synthesis of compounds [**1**]–[**11**] and their structural validation of the compounds can be found in the SI Synthetic and Analytical Chemistry.

### Bacterial Strains and Culture Media

*P. aeruginosa* strain PAO1 (ATCC 15692, LMG 12228) was obtained from the Belgian Coordinated Collections of Microorganisms (BCCM). All clinical strains of *P. aeruginosa* and *A. baumannii* were provided by the French National Reference Centre for *P. aeruginosa* collection.

For routine culture, bacteria were grown in cation-adjusted Mueller–Hinton broth (CAMHB) or on CAMH-Agar. For culture in low-iron media, two media were routinely used, specifically low-iron M9 medium^40^ and iron-depleted cation-adjusted Mueller–Hinton broth (ID-CAMHB). The low-iron M9 medium contained 6.8 g L^–1^ Na_2_HPO_4_, 3 g L^–1^ KH_2_PO_4_, 0.5 g L^–1^ NaCl, 1 g L^–1^ NH_4_Cl, 0.4% (w/v) glucose, 2 mM MgSO_4_, 0.1 mM CaCl_2_, and 0.2% (w/v) casein amino acids, with a residual iron concentration of approximately 0.18 μM, determined by inductively coupled plasma optical emission spectroscopy (ICP-OES, LASIRE CNRS UMR 8516). Iron-depleted cation-adjusted Mueller–Hinton broth (CAMHB) was prepared by treating standard CAMHB (BD, 212322) with Chelex 100 resin (Bio-Rad, biotechnology grade) for 6 h at room temperature, followed by supplementation with calcium, magnesium, and zinc at final concentrations of 22.5, 11.25, and 0.75 mg L^–1^, respectively, according to EUCAST guidelines.^69^ To minimize iron contamination and maintain consistent iron-restricted conditions, all reagents used for medium preparation were of trace-metal-basis grade and purchased from Sigma-Aldrich (France): CaCl_2_ (ref 499609), ZnSO_4_·7H_2_O (ref 935662), MgSO_4_ (ref 203726), NH_4_Cl (ref 326372), Na_2_HPO_4_·7H_2_O (ref 431478), KH_2_PO_4_ (ref 229806), and NaCl (ref 204439).

### Generation of PAO1 Gene Knockout Strains

Isogenic *P. aeruginosa* PAO1 knockout strains of *oprD, mexR, pfeA, pirA, fpvA,* and *fptA* were generated using the double crossover method using the pFOG-based suicide vector.^87^ Briefly, for each target gene, around 700 bp regions upstream and downstream were amplified form PAO1 gDNA, using the primers in Table S9 and assembled with linearized pFOG (*Bam*HI and *Eco*RI digest) using the NEBuilder HiFi DNA Assembly Kit (NEB). This resulted in the formation of pFOG-*oprD-*flanks, pFOG-*mexR-*flanks, pFOG-*pfeA-*flanks, pFOG-*pirA-*flanks, pFOG-*fpvA-*flanks and pFOG-*fptA-*flanks. Two microliters of the assembled constructs were then transformed into chemically competent *E. coli* TransforMax EC100D pir+ (Bioresearch Technologies), serving as the donor strain of the pFOG-based vectors.

To introduce the pFOG-based plasmids into *P. aeruginosa* PAO1, a triparental conjugation strategy was used. Briefly, an overnight culture of the donor strain (*E. coli* EC100 pir+ with pFOG-based vectors) and Ec1046 helper strain (*E. coli* HB101 with plasmid pRK2013, kanR)^88^ were spotted (25 μL each) and coincubated on LB agar at 37 °C for 2 h. At the same time, the recipient strain (PAO1) was incubated separately at 42 °C, after which 25 μL was added to the donor-helper mixture and incubated (6 h at 37 °C). Mixed spots were then resuspended in fresh LB medium and plated on Pseudomonas Isolation Agar containing 25 mg/L irgasan (triclosan) and 250 mg/L gentamicin to select for the first crossover PAO1 transformants.

To select for double-crossover gene deletion mutants, single-crossover transformants were grown in the absence of antibiotics, followed by plating on LB-no salt agar supplemented with 20% sucrose and 0.5 μg mL^–1^ anhydrotetracycline (incubated in the dark, 28 °C, 24 h, to counter-select SacB and to induce I-SceI).^87^ Individual colonies were screened for the desired gene deletion using PCR with primers located outside the targeted region (Table S9). For double mutants, this gene inactivation procedure was performed twice, once of each gene.

### Expression and Purification of Recombinant Truncated Pa-PBP3

A recombinant C-terminal His-tagged truncated variant of the *ftsI* gene (PBP3) lacking the first 34 amino acids was expressed in and purified from *E. coli*, similar to a previous report.^68^ Briefly, the truncated *ftsI* was amplified from PAO1 genomic DNA (using primers 41 and 42, Table S9) and cloned into the *NcoI* and *XhoI* sites of pET28a, giving pET28–34PBP. pET28–34PBP was transformed into chemically competent *Escherichia coli* NiCo21­(DE3) (New England Biolabs) selected on kanamycin. A kanamycin-resistant colony (transformant) was expanded in LB medium (1 L, at 37 °C, shaking) to OD_600_ = 0.3, and protein expression was induced by addition of 1 mM IPTG (4 h, 37 °C, shaking). Cells were then harvested (8000*g*, 20 min, 4 °C), and expression of the recombinant protein (predicted molecular weight 60128.66 Da) was verified by SDS–PAGE analysis. For protein purification, the bacterial pellet was resuspended in 20 mM phosphate buffer, pH 7.0, 500 mM NaCl, and lysed by two passes through a French press (one shot cell disruptor, I&L Biosystems) at 1500 psi. Lysates were centrifuged (48000*g*, 30 min, 4 °C), followed by purification on an NGC chromatography system (Bio-Rad, Quest 100 Plus) using a HisTrap HP 5 mL column (Cytiva) and a mobile phase gradient of 25–500 mM imidazole in 20 mM phosphate buffer, pH 7.0, 500 mM NaCl. Fractions containing the isolated target protein (by SDS–PAGE) were pooled and concentrated using a Vivaspin Turbo 15 concentrator (30 kDa MWCO, Sartorius), and buffer exchanged with a Zeba spin desalting column (7 kDa MWCO, 10 mL; Thermo Fisher Scientific) to remove imidazole.

### Bocillin-FL Fluorescence Polarization (FP) Assay to Measure Pa-PBP3 Inhibition

The inhibition of purified recombinant His-PBP3 was determined using a previously described bocillin-FL fluorescence polarization assay,^66,67^ with slight modifications. Briefly, 50 μL of purified His-PBP3 (at a final concentration of 1 μM) in potassium phosphate buffer (50 mM, pH 7.4) was mixed with serially diluted meropenem (0–100 μM, DMSO content maintained at 4% for all samples). Samples were then preincubated (10 min, 30 °C, 300 rpm), after which bocillin-FL (1 μM) was added to all samples (10 min, 30 °C, 300 rpm) to bind remaining free His-PBP3. Fluorescence polarization was then recorded on a PerkinElmer EnVision microplate reader (excitation/emission 480/535 nm) using black 384-well microplates (Greiner). Fluorescence data were background corrected and expressed as a percentage activity compared to the no-inhibitor control, allowing for the calculation of inhibitor IC_50_.

### NDM-1 Enzyme Inhibition Assay

The inhibitory activity of MBL inhibitor conjugates against recombinant NDM-1 was determined using nitrocefin (EUROMEDEX) as a chromogenic substrate, as previously described.^89^ Recombinant NDM-1 (Gly29–Arg270) was purchased from RayBiotech (USA). Assays were performed in a total reaction volume of 100 μL in 50 mM HEPES buffer (pH 7.2) supplemented with 1 μM ZnSO_4_ and 0.01% Triton X-100, using flat-bottom 96-well microplates (TC Plate, SARSTEDT). Briefly, recombinant NDM-1 was used at a final concentration of 6 nM and incubated with 18 concentrations of each inhibitor (0–10 μM; 3-fold serial dilutions). The final DMSO concentration in all reactions was maintained at 1% (v/v). Enzyme and inhibitors were incubated for 15 min at 25 °C before initiating the reaction by the addition of nitrocefin to a final concentration of 10 μM. Nitrocefin hydrolysis was monitored by measuring absorbance at 492 nm for 45 kinetic cycles using an Infinite M Plex microplate reader (Tecan). Initial reaction velocities were calculated from the linear portion of progress curves. IC_50_ values were determined from three independent experiments by fitting normalized initial velocity data to a four-parameter logistic model using GraphPad Prism version 10.4.1

### ^57^Fe Uptake Assay

The ^57^Fe uptake assay was performed using an Agilent 7900 ICP-MS (platform of the Research Federation Michel-Eugène Chevreul hosted by the LASIRE laboratory), as previously described.^40,90^ ^57^Fe_2_O_3_ (Trace Sciences International Corp.) was first dissolved in concentrated HCl (12 M) and subsequently diluted with water for analysis (Acros Organics) to a final stock concentration of 1 mM. Bacteria from an overnight culture were inoculated into low-iron M9 medium and grown at 37 °C until reaching an OD600 of 0.4–0.6. The cultures were then centrifuged (4,000 rpm, 10 min, 4 °C), and the cell pellets were resuspended in one-fourth of the original culture volume of M9 medium to obtain an OD600 greater than 2. Siderophores and siderophore conjugates were preloaded with 0.9 equiv. of ^57^Fe. Ten microliters of each prepared solution was dispensed into wells of a 96-well microplate (TC Plate, SARSTEDT), followed by the addition of 90 μL of the concentrated bacterial suspension to achieve a final compound concentration of 10 μM. The plate was sealed and incubated at 30 °C for 1 h with shaking. Following incubation, bacterial cells were collected by centrifugation (4,000 rpm, 10 min, 4 °C) and washed three times. The first two washes were performed with M9 medium, whereas the final wash was carried out using M9 medium supplemented with 2% (w/v) EDTA to chelate and remove extracellular ^57^Fe that had not been internalized by the bacteria. The bacterial pellets were digested in concentrated nitric acid for 30 min at room temperature and subsequently diluted with water to a final nitric acid concentration of 2%. The ^57^Fe content was quantified by ICP-MS and normalized to the magnesium content (measured simultaneously by ICP-MS) to account for differences in bacterial biomass and determine relative ^57^Fe uptake.

### Antibacterial Assays

The antimicrobial activity of compounds of interest was determined on *P. aeruginosa* cultured in specific low-iron media such as low-iron M9 medium or iron-depleted cation-adjusted Mueller–Hinton broth (ID-CAMHB), or in these media supplemented with 10 μM FeCl_3_ (as indicated in results). Overall, the antimicrobial activity was determined using a standard microdilution assay following EUCAST guidelines. For compound distribution (including checkerboard combinations), antibiotic stocks were prepared in dimethyl sulfoxide (DMSO) and dispensed as 2-fold serial dilutions into 384-well microplates (Greiner, 781182) using a D300e digital dispenser (Tecan). *P. aeruginosa* strains of interest were grown into exponential phase in specified media, after which they were diluted into the same specified media (to OD_600_ = 0.001) and 50 μL added to the microtiter wells. Plates were sealed and incubated (37 °C for at least 20 h), after which bacterial growth was assessed visually and antibiotic activity was reported following the EUCAST criteria.^91^

### HepG2 Cytotoxicity Assay

HepG2 cytotoxicity was determined by the ARIADNE-ADME platform (French National Infrastructure ChemBioFrance). Briefly, HepG2 cells were seeded in a 384-well plate (3000 cells per well in 40 μL DMEM containing 10% fetal calf serum). Following 24 h for cell adhesion, a concentration range (0, 12.5, 25, 50, and 100 μM) of compounds of interest (dissolved in DMSO) was added to the wells, as well as Hoechst 33342 (20 ng/mL) and propidium iodide (1 μg/mL). The final volume per well was 70 μL (0.4% DMSO). Carfilzomib (499, 249,142, 61, and 30.6 nM) was used as a positive control cytotoxic compound in this assay. At 24 , 48, and 72 h after compound addition, live imaging was performed using the automated confocal microscope In Cell Analyzer 6000 (GE Healthcare). The cytotoxicity was defined as the percentage of cells labeled by propidium iodide in the total cell population (Hoechst-labeled cells) using Columbus software (PerkinElmer). Compounds were tested twice in technical triplicates.

### Determination [10] and [11] Stability in “Spent Culture” Filtrate and in Bacteria

“Spent” medium was prepared from the culture filtrate of an overnight culture of *P. aeruginosa* PAO1 growing in iron-depleted cation-adjusted Mueller–Hinton broth (conditions similar to those used for MIC assays), by centrifuging the culture and filtering the culture supernatant (0.22 μm filter). 100 μM of conjugate [10] or [11] was spiked into this spent medium and incubated at 37 °C. To measure the conjugate stability, samples (100 μL) were taken at different time points (0, 4 and 24 h), mixed with an equal volume of methanol, centrifuged, and the supernatant analyzed by analytical UHPLC-MS. The amount of conjugate in the samples was measured from the area under the peak of the extracted single-ion chromatogram of the single charge [10] (mass call: 1226.54) or [11] (mass call: 1341.48), and expressed as a percentage of that measured at time 0.

To determine compound stability in bacteria, *P. aeruginosa* PAO1 cells were grown to exponential phase in iron-depleted cation-adjusted Mueller–Hinton broth under the same conditions used for MIC assays. Cells were collected and resuspended in fresh iron-depleted cation-adjusted Mueller–Hinton broth to a final volume of 400 μL and adjusted to a final bacterial density of 10^9^ cfu/mL. Either conjugate [10] or [11] was then added to a final concentration of 100 μM, and samples were incubated at 37 °C for 4 h. Following incubation, bacterial cells were collected by centrifugation and resuspended in water:DMSO (1:1, v/v). The presence of intact conjugates and/or potential degradation products was assessed by analytical UHPLC-MS.

### Accumulation of [10] and [11] in *P. aeruginosa* PAO1

*P. aeruginosa* PAO1 wildtype and the knockout mutants Δ*pfeA*, Δ*pirA*, and Δ*pfeA*Δ*pyrA* were grown to exponential phase in CAMHB (high-iron media condition) or ID-CAMHB (low-iron medium condition). Cells were collected and resuspended in fresh iron-depleted cation-adjusted Mueller–Hinton broth to a final volume of 400 μL and adjusted to a final OD_600 nm_ of 1.0 (approximately, 10^9^ cfu/mL). Conjugate [10] or [11] was then added to a final concentration of 100 μM, and samples were incubated at 37 °C for 1 h. Following incubation, bacterial cells were collected by centrifugation and resuspended in water:DMSO (1:1, v/v). Conjugate accumulation was analyzed by analytical UHPLC-MS. Chromatographic peaks were monitored by UV absorbance at 305 nm, corresponding to the absorption maximum of both conjugates, and the area under the UV peak (AUC) was determined. In parallel, extracted ion chromatograms (EICs) corresponding to the singly charged ions of [10] (*m*/*z* 1226.54) and [11] (*m*/*z* 1341.48) were integrated, and the corresponding AUC values were obtained.

### Evaluation of *In Vivo* Compound Efficacy in *P. aeruginosa*-Infected *Galleria Mellonella*

The *in vivo* efficacy of meropenem and cefiderocol, administered either alone or in combination with siderophore–MBLi conjugates, was assessed using a previously described *G. mellonella* infection model^81^ with minor modifications. Final instar stage *G. mellonella* larvae (purchased from Valomolia, Bischheim, France) weighing approximately 350 mg were maintained at room temperature (in the dark) until use. Individuals were then selected for the experiments. The bacterial infection inoculum was prepared by culturing *P. aeruginosa* strains to mid-logarithmic phase in cation-adjusted Mueller–Hinton broth (CAMHB), followed by two washes in sterile phosphate-buffered saline (PBS), and a final resuspension in PBS to 2 × 10^3^ CFU mL^–1^. Groups of ten larvae were then injected with 20 CFU per larvae in the left proleg using a Hamilton microsyringe fitted with a 30-gauge needle and maintained at 37 °C (in the dark). All drug stocks were prepared in 30% DMSO, ensuring that the final injection volume (10 μL) resulted in a DMSO concentration below 1%. Two hours postinfection, a single dose (10 μL) of either meropenem (20 mg kg^–1^), cefiderocol (20 mg kg^–1^), alone or in combination with MBLi conjugates (final larval concentration of 8 μmol kg^–1^ (∼8 μM)), was injected into the right proleg of larvae. Larvae were then maintained at 37 °C (in the dark), and larval survival was monitored every 12 h. Larvae were considered dead when no response was observed upon touch and were removed at each time point. All experiments included several control groups, consisting of larvae injected with sterile PBS and infected larvae treated with PBS. Survival curves were analyzed using the Kaplan–Meier method, and statistical comparisons between groups were performed using the log-rank test and Gehan-Breslow-Wilcoxon test, with *p* ≤ 0.05 considered statistically significant (GraphPad Prism 10.4.2).

## Supplementary Material





## Notes

Additional experimental data, chemical synthesis procedures, related analytical data including NMR spectra of all novel compounds (PDF)
Figure S1. Coupling of Enterobactin [1] with 3-methylpyridine using direct iodine-mediated oxidation (A) and iodonium salt-mediated oxidation (B) strategies. Figure S2. Pa-PBP3 expression and purification. Figure S3. Dose response curves of meropenem conjugates with Pa-PBP3 (PDF)
