# Supplementary material for: Siderophore-Enabled Delivery of Carbapenem and Metallo-β-Lactamase Inhibitor: A Promising Strategy against Multidrug-Resistant Pseudomonas aeruginosa
Source: J Am Chem Soc. 2026 Sep 27;148(39):41959–77. doi: 10.1021/jacs.6c08350 (PMC13651510; doi:10.1021/jacs.6c08350)

Supplementary Information

Synthetic and Analytical Chemistry

Siderophore-Enabled Delivery of Carbapenem and Metallo-β-Lactamase Inhibitor: A Promising Strategy Against Multidrug-Resistant *Pseudomonas aeruginosa*

AUTHOR NAMES: Shahriar SAFARI[1§], Ravil PETROV[1§], Yulia P. USTIMENKO[1], Katy JEANNOT[2], Xavier TRIVELLI[3], Ernesto ANOZ-CARBONELL[1], Ruben C. HARTKOORN[1]*

[1] Univ. Lille, CNRS, Inserm, CHU Lille, Institut Pasteur Lille, U1019 - UMR 9017 - CIIL - Center for Infection and Immunity of Lille, F-59000 Lille, France.

[2] Bacteriology Unit, University Hospital of Besançon, F-25000 Besançon, France; Associated French National Reference Center for Antibiotic Resistance in *Pseudomonas* spp and *Acinetobacter* spp, F-25000 Besançon, France.

[3] Univ. Lille, CNRS, Centrale Lille, UAR2638 - IMEC - Institut Michel-Eugène Chevreul, F-59000 Lille, France.

[§] *S.S.* and *R.P.* contributed equally to this work

[*] ruben.hartkoorn@inserm.fr

Contents

Synthetic and Analytical Chemistry S5

Materials S5
Analytical methods and purification S5
NMR spectrometry S5

Synthetic pathways S6

Preparation of Substrates for the Model Reaction S6
Comp. *MS1* S6
Comp. *MS2* S6
Comp. *MS3* S6
Comp. *MS5 (entry 13 for LC-MS characterization)* S6
General Procedure for Model Reactions S6
Bis(3-methylpyridine)I^+ BF_4^- S6

Synthesis of Enterobactin [1] S7

Comp. 12 .... S7
Comp. 13 .... S7
Comp. 14 .... S7
Comp. 15 .... S7
Comp. 16 .... S8
Comp. 17: Triserine trilactone trihydrochloride S8
Comp. 18 .... S8
Comp. 1: Enterobactin S9
Synthesis of TRENCAM S9
Comp. 19 .... S9
Comp. 20 .... S9
Comp. 2: TRENCAM S10
Synthesis of Meropenem-functionalized linkers 4 and 5 .... S10
Comp. 21 .... S10
Comp. 22 (HCl salt) S11
Comp. 23 (free base) S11
Comp. 4 .... S11
Comp. 5 .... S12
Synthesis of Meropenem-siderophore conjugates 7 and 8 .... S13
Silver 2-ethylhexanoate (AgEHA) S13
Comp. 6 .... S13
Comp. 7 .... S13
Comp. 7b S14
Comp. 8 .... S14
Synthesis of comp. 9 (PEG-linked indole–pyridyl analogue) S14
Comp. 24, ethyl 7-isopropyl-*1H*-indole-2-carboxylate S14
Comp. 25, ethyl 3-iodo-7-isopropyl-*1H*-indole-2-carboxylate S15
Comp. 26 .... S15
Comp. 27 .... S15
Comp. 28 .... S16
Comp. 9 .... S16
Synthesis of MBL-siderophore conjugates 10 and 11 .... S17

Synthetic procedures for MBL-siderophore conjugates 10 and 11 .... S17

Comp. 11 .... S17
Comp. 10 .... S17
References S18

Low resolution UHPLC-MS and HRMS data S20

Comp *MS1* S20
Comp *MS1* with 1 eq I_2 added S21
Comp *MS1* in the presence of 1 eq I_2 and 2 eq STF (reaction mixture) S22
Bis(3-methylpyridine)I^+ BF_4^- S23
Analytical Results for the Model Reaction S24
Entry 1 (control experiment, a 1:1 eq mixture of *MS1* and *MS2*, no oxidant) S24
Entry 4 (1 eq I_2 added to 1:1 eq mixture of *MS1* and *MS2*) S25
Entry 6 (1 eq I_2 was added to 1:1 eq mixture of *MS1* and *MS2,* followed by 1 eq STF) S26
Entry 7 (1 eq STF was added to 1:1 eq mixture of *MS1* and *MS2,* followed by 1eq I_2) S27
Entry 8 (1 eq I_2 was added to 1:1 eq mixture of STF and *MS1,* followed by 1 eq *MS2*) S28
Entry 9 (1 eq I_2 was added to a mixture of 2 eq STF and 1 eq *MS1,* followed by 1 eq *MS2*) S29
Entry 10 (1 eq I_2 was added to a mixture of 1 eq STF and 2 eq *MS1,* followed by 1 eq *MS2*) S30
Entry 11 (1 eq of bis(3-methylpyridine) iodonium was added to 1 eq *MS2*) S31
Entry 12 (3 eq of bis(3-methylpyridine) iodonium was added to 1 eq Enterobactin) S32
Entry 13 (2 eq STF was added to 1:1 eq mixture of *MS1* and *Enterobactin,* followed by 1eq I_2) S34
Comp. 1 (Enterobactin) S35
Comp. 2 (TRENCAM) S36
Comp. 4 .... S37
Comp. 5 .... S38
Comp. 6 .... S39
Comp. 7 .... S41
Comp. 7, HRMS (ESI): *m/z* $[M]^+$ calcd for $C_{65}H_{78}N_9O_{25}S^+$ 1416.4830, found 1416.4878 .... S42
Comp. 7b S43
Comp. 8 .... S44
Comp. 8, HRMS (ESI): *m/z* $[M]^+$ calcd for $C_{62}H_{81}N_{10}O_{19}S^+$ calculated 1301.5395, found 1301.5378 .... S45
Comp. 9 .... S46
Comp. 10 .... S47
Comp. 11, HRMS (ESI): *m/z* $[M]^+$ calcd for $C_{67}H_{73}N_8O_{22}^+$ 1341.4839, found 1341.4832 .... S48
Comp. 10 .... S49
Comp. 10, HRMS (ESI): *m/z* $[M]^+$ calcd for $C_{64}H_{76}N_9O_{16}^+$ 1226.5405, found 1226.5405 .... S50
Comp. 21 .... S51
Comp. 22 .... S52
Comp. 25 .... S53
Comp. 26 .... S54
Comp. 27 .... S55
Comp 28. .... S56

NMR data S57

MS1, ^{1}H NMR in DMSO-d_6, Bruker-300 .... S57
MS1, ^{13}C NMR of DMSO-d_6, Bruker-300 .... S57
Comp. *MS1* (^{1}H/^{13}C/^{15}N chemical shifts assignments) S58
Effect of iodine and iodine/STF mixture on the ^{1}H NMR spectrum of comp MS1 .... S59
Comp. *MS3*, ^{1}H NMR in DMSO-d_6 .... S60
Comp. *MS3*, ^{13}C NMR in DMSO-d_6 .... S60
Comp. *MS3* (^{1}H/^{13}C/^{15}N chemical shifts assignments) S61
Comp. *MS5* (^{1}H/^{13}C/^{15}N chemical shifts assignments) S63

Comp. [bis(3-methylpyridine)I]$^+$BF$_4^-$, ^{1}H NMR in CDCl$_3$ S65
Comp. bis(3-methylpyridine)I$^+$BF$_4^-$, ^{13}C NMR in CDCl$_3$ S65
Comp. 1, ^{1}H NMR in DMSO-d_6.......... S66
Comp. 1, ^{13}C NMR in DMSO-d_6.......... S66
Comp. 2, ^{1}H NMR in DMSO-d_6, Bruker-300.......... S67
Comp. 2, ^{13}C NMR in DMSO-d$_6$, Bruker-300.......... S68
Comp. 4, ^{1}H NMR in DMSO-d_6, Bruker-300 .... S69
Comp. 4, ^{13}C NMR in DMSO-d_6, Bruker-300 .... S70
Comp. 5 ^{1}H NMR in DMSO-d_6, Bruker-300 .... S71
Comp. 5, ^{13}C NMR in DMSO-d_6, Bruker-300 .... S72
Comp. 6, ^{1}H/^{13}C/^{15}N chemical shifts in DMSO-d_6 .... S73
Comp. 7, ^{1}H/^{13}C/^{15}N chemical shifts in DMSO-d_6 .... S75
Comp. 8, ^{1}H/^{13}C/^{15}N chemical shifts in DMSO-d_6 .... S77
Comp. 9, ^{1}H NMR in DMSO-d_6, Bruker-300 .... S79
Comp. 9, ^{13}C NMR in DMSO-d_6, Bruker-300 .... S79
Comp. 11, ^{1}H/^{13}C/^{15}N chemical shifts in DMSO-d_6 .... S80
Comp. 10, ^{1}H/^{13}C/^{15}N chemical shifts in DMSO-d_6 .... S82
Comp. 16, ^{1}H NMR in DMSO-d_6, Bruker-300.......... S85
Comp. 16, ^{13}C NMR in DMSO-d_6, Bruker-300.......... S86
Comp. 18, ^{1}H NMR in CDCl$_3$, Bruker-300.......... S87
Comp. 18, ^{13}C NMR in CDCl$_3$, Bruker-300.......... S88
Comp. 20, ^{1}H NMR in CDCl$_3$, Bruker-300.......... S89
Comp. 20, ^{13}C NMR in CDCl$_3$, Bruker-300.......... S90
Comp. 21, ^{1}H NMR in DMSO-d_6, Bruker-300 .... S91
Comp. 21, ^{1}H NMR in CDCl$_3$, Bruker-300 .... S91
Comp. 21, ^{13}C NMR in CDCl$_3$, Bruker-300 .... S92
Comp. 22, ^{1}H NMR in CDCl$_3$, Bruker-300 .... S92
Comp. 22, ^{13}C NMR of in CDCl$_3$, Bruker-300.......... S93
Comp. 25, ^{1}H NMR in DMSO-d_6, Bruker-300 .... S94
Comp. 25, ^{13}C NMR in DMSO-d_6, Bruker-300 .... S94
Comp. 27, ^{1}H NMR in DMSO-d_6, Bruker-300 .... S95
Comp. 27, ^{13}C NMR in DMSO-d_6, Bruker-300 .... S95
Comp. 28, ^{1}H NMR in DMSO-d_6, Bruker-300 .... S96
Comp. 28, ^{13}C NMR in DMSO-d_6, Bruker-300 .... S96

Synthetic and Analytical Chemistry

Materials

All solvents and reagents were purchased from commercial suppliers, including Sigma-Aldrich, TCI (Belgium), Thermo Scientific Chemicals, ABCR (Germany), and BLD Pharm, and were used as received. Chloroform ($CHCl_3$) and dichloromethane (DCM) were stored over freshly activated 4 Å molecular sieves. Dibutyltin (IV) oxide (CAS 818-08-6, purity 98%), 2,3-dihydroxybenzoic acid (CAS 303-38-8, 99%), benzyl bromide (CAS: 100-39-0, 98%), *N*-hydroxysuccinimide (NHS, CAS: 6066-82-6, 98%), EDAC (1-(3-dimethylaminopropyl)-3-ethylcarbodiimide hydrochloride, 98%, CAS 25952-53-8), TFA (trifluoroacetic Acid, CAS 76-05-1, 99.5%), DSC (*N,N*′-disuccinimidyl carbonate, CAS 74124-79-1, 98%), DIPEA (*N,N*-diisopropylethylamine, CAS 7087-68-5, 99%), silver nitrate (CAS 7761-88-8, >99%), glacial acetic acid (CAS: 64-19-7, 99%), potassium trimethylsilanolate ($KOSiMe_3$, CAS 10519-96-7, 2 M solution in THF), and silver tetrafluoroborate (STF, CAS 14104-20-2, 99%) were obtained from Thermo Scientific Chemicals. (S)-Methyl 3-hydroxy-2-(tritylamino)propanoate (Trt-*L*-Ser-OMe, CAS 4465-44-5, 95%), tris(2-aminoethyl)amine (CAS 4097-89-6, 98%), *N,N'*-dicyclohexylmethanediimine (DCC, CAS 538-75-0, 99%), NH_2-PEG_3-C_2-Boc (CAS 252881-74-6, 97%), Meropenem trihydrate (CAS 96036-03-2, 97%), 2-isopropylaniline (*o*-aminocumene, CAS 643-28-7, 95%), ethyl-2-methylacetoacetate (CAS 609-14-3, 95%), *N*-iodosuccinimide (NIS, CAS 516-12-1, 98%), [1,1'-bis(diphenylphosphino)ferrocene]dichloropalladium(II) (Pd(dppf)Cl_2, CAS 72287-26-4, 98%), 4-(*tert*-butoxycarbonylaminomethyl)phenylboronic acid pinacol ester (CAS 330794-35-9, 96%) were obtained from BLD Pharma. 1-Hydroxybenzotriazole hydrate (HOBt, 99% by dry wt, water >20%, CAS 123333-53-9) and 2-(pyridin-3-yl)acetic acid (CAS 501-81-5, 95%) were obtained from ABCR GmbH. Potassium 2-ethylhexanoate (KEHA, CAS 3164-85-0, >95.0%) and 1,1′-carbonyldiimidazole (CDI, CAS 530-62-1, >97.0%) was purchased from TCI Europe.

Analytical methods and purification

The progress of reactions was routinely monitored by thin layer chromatography (TLC) using MilliporeSigma commercial glass plates coated with silica gel 60 F254. Visualization was achieved by monitoring fluorescence under UV light at 254 and 365 nm. Flash column chromatography was performed using prepacked silica gel cartridges (SiO_2, 30-50 µm average diameter) and Interchim PuriFlash XS 420 system. UV detection at 250 nm and 280 nm was used to direct collections of the relevant fractions.

Analytical spectrometry analysis (UHPLC-MS) were performed on an Ultimate 3000 UHPLC system, coupled with an LCQ Fleet Ion Trap Mass Spectrometer (Thermo Scientific). Chromatographic separation was achieved using an Acquity UPLC Peptide BEH C_{18} Column (300Å, 1.7 µm, 2.1 mm x 100 mm). Mobile phase system was composed of solvent A (H_2O, 0.1% formic acid) and B (acetonitrile, 0.1% formic acid), typically run through a linear gradient from 0% to 100% of solvent B in 12 min. Elution of compounds was monitored by UV absorbance at 215 nm and 254 nm, and by mass spectrometry electrospray ionization. Chromatographic peaks were processed using the ICIS automatic peak detection algorithm in Thermo Scientific FreeStyle™ (version 1.8 SP2 QF1) with default integration parameters.

Where indicated, compounds were purified using a BUCHI C-830 automated flash chromatography system using either a semipreparative XBridge™ Peptide BEH C_{18} column (10 x 250 mm, 130 Å, 5 micron) or preparative XBridge™ Peptide BEH C_{18} OBDTM column (19 x 150 mm, 130 Å, 5 micron) from Waters. Unless specified, compounds were eluted using the same mobile phase system as described above for UHPLC-MS. The corresponding fractions were assayed by analytical UHPLC-MS to determine the molecular weights and purities, and fractions with the desired characteristics were pooled together. Acetonitrile was evaporated and the aqueous solution was lyophilized to yield the purified products. The purity of all compounds prepared for biological testing, as determined by UHPLC, was greater than 95%. Yields refer to chromatographically pure compounds as determined by TLC (single spot) or HPLC.

High resolution mass spectrometry of purified compounds was performed by the ARIADNE-ADME platform (Institut Pasteur de Lille, France), using a quadrupole time-of-flight (TOF) LCT Premier XE mass spectrometry machine (Waters).

NMR spectrometry

NMR spectra were recorded on Bruker Avance III300 spectrometer equipped with a 5 mm BBO (X-^1H) probe or a Bruker Avance IIIHD 600 equipped with a 5 mm cryogenic QCI (^1H/^2H/^{13}C/^{15}N/^{19}F) probe. The ^1H and ^{13}C spectra were referenced to the signals of residual organic solvents as internal references, to the DSS trimethylsilyl signal

(0.00 ppm) for aqueous solutions and to the residual TFA signal for ^{19}F spectra. Indirect referencing was used for ^{15}N. Chemical shifts (δ) are in parts per million (ppm) downfield from tetramethylsilane (TMS). NMR coupling constants (J) are reported in Hertz (Hz), and splitting patterns are indicated as follows: s (singlet), br (broad singlet), d (doublet), dd (doublet of doublet), m (multiplet).

Synthetic pathways

Preparation of Substrates for the Model Reaction

Comp. *MS1*

The title compound was synthesized following the reported method, and the respective spectroscopic data (NMR and MS) agreed with those reported in the literature.[1]

Comp. *MS2*

The synthesis and analytical characterization of the title compound was presented in our previous paper.[2]

Comp. *MS3*

Comp ***MS2*** (20 mg, 0.077 mmol, 1.00 equiv) and ***MS1*** (27 mg, 0.082 mmol, 1.06 equiv) were suspended in DMSO (600 µL) in a 2 mL Eppendorf plastic test tube. A solution of silver tetrafluoroborate (16 mg, 0.082 mmol, 1.06 equiv) in DMSO (300 µL) was added dropwise to the mixture, followed by gentle shaking to ensure proper mixing. Subsequently, a solution of iodine (20 mg, 0.079 mmol, 1.03 equiv) in DMSO (200 µL) was added under vortex mixing. The reaction mixture was then centrifuged to remove the precipitate. The supernatant was diluted with DMSO to a total volume of 3 mL and purified by reverse-phase HPLC (C^{18}) using H_2O/MeCN as eluents to afford the target compound as a pale-yellow solid. Yield: 24 mg (42%).

^1H NMR (300 MHz, DMSO-d_6): δ 9.31 (s, 1H), 8.97 – 8.87 (m, 2H), 8.47 (d, J = 8.1 Hz, 1H), 8.15 (dd, J = 8.1, 6.0 Hz, 1H), 8.03 (t, J = 6.0 Hz, 1H), 7.90 (d, J = 7.5 Hz, 2H), 7.69 (d, J = 7.4 Hz, 2H), 7.42 (t, J = 7.4 Hz, 3H), 7.37 – 7.28 (m, 3H), 4.43 (t, J = 6.5 Hz, 5H), 4.25 (t, J = 6.5 Hz, 1H), 3.87 (d, J = 5.6 Hz, 2H), 3.54 (s, 3H).

^{13}C NMR (300 MHz, DMSO-d_6) δ 171.69, 171.38, 170.20, 163.42, 156.94, 146.05, 145.25, 145.10, 144.86, 144.24, 141.27, 140.36, 132.86, 128.12, 127.54, 125.54, 120.64, 66.08, 52.22, 47.22, 41.30, 41.25.

Comp. *MS5 (entry 13 for LC-MS characterization)*

Enterobactin (44 mg, 0.066 mmol) and comp **MS1** (21.6 mg, 0.066 mmol) were reacted in the presence of STF (25.5 mg, 0.132 mmol, 2 eq) and iodine (16.7 mg, 0.066 mmol, 1 eq) in DMSO using the same procedure described above for comp **MS3** to give the title product as a white solid after purification by reverse phase HPLC with water/acetonitrile (0.1% TFA) gradient. Yield: 26 mg (39%).

General Procedure for Model Reactions

Stock solutions of compounds ***MS1***, ***MS2***, STF, and iodine were prepared in DMSO (0.5 mL each, 25 mM). Metallic silver nanoparticles (Ag NPs) were synthesized according to a literature protocol.[3] The obtained nanoparticles were pelleted by centrifugation, and the supernatant was discarded. The solid was weighed and resuspended in DMSO to obtain a suspension of nominal concentration 25 mM. For each analysis, 10 µL aliquots of the respective stock solutions were combined in the specified order (see entry numbers in **Scheme 1** in the main text). The resulting mixtures were centrifuged to remove the precipitate, after which 10 µL of each supernatant was further diluted with DMSO (200 µL) and analyzed by LC–MS.

Bis(3-methylpyridine)I^+ BF_4^-

The title compound was prepared according to a literature procedure for structurally related molecules in comparable yield to aid in LC–MS data interpretation.[4] While its UV signal was not detectable under the LC–MS conditions employed, a direct MS infusion provided its characteristic mass spectral signature for comparison.

^1H NMR (300 MHz, $CDCl_3$): δ 8.58 (br. s, 1H), 8.53 (d, J = 5.0 Hz, 1H), 7.74 (d, J = 7.9 Hz, 1H), 7.41 (dd, J = 7.8, 5.4 Hz, 1H), 2.38 (s, 3H).

^{13}C jmod NMR (75 MHz, $CDCl_3$): δ 151.22 (CH), 148.16 (CH), 140.72 (CH), 136.14 (CH), 125.21 (CH), 77.16 (C_q), 18.51 (CH_3).

Synthesis of Enterobactin [1]

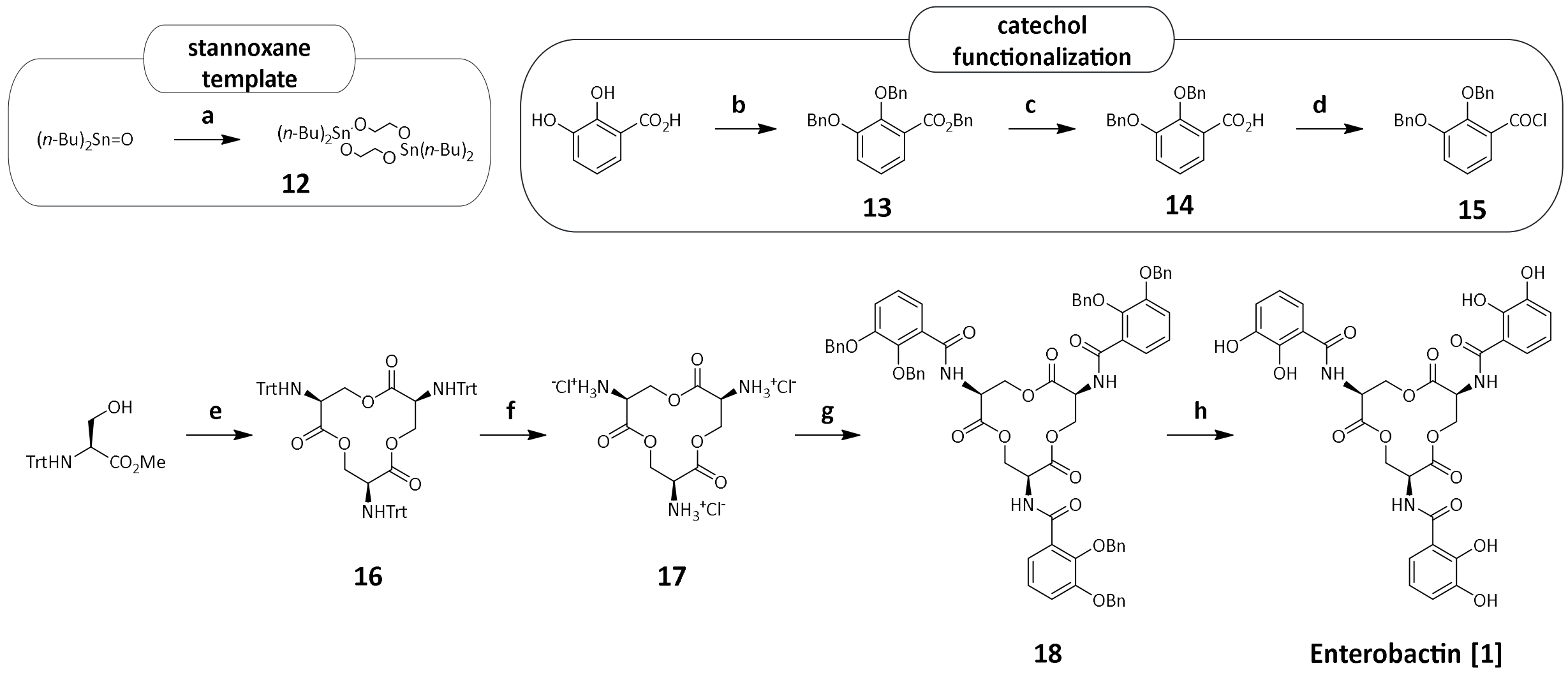

Scheme 1. Synthesis of Enterobactin following representative literature methods,[5–8] reagents and conditions: a) 1 eq. ethylene glycol, benzene, reflux overnight (99%); b) 5.6 eq. BnBr, 16.7 eq. K_2CO_3, acetone, reflux, 12h, 98%; c) 3 eq. KOH, EtOH, reflux, H^+, 87%; d) $SOCl_2$, benzene, reflux; e) 0.05-0.15 eq. template 12, toluene (or xylene), reflux, 48-72 h (44-95%); f) 8 eq. TFA, DCM, 0 °C to RT, 1 h / 6 eq HCl in EtOH, 99%; g) 4 eq. comp. 15, 10 eq. DIPEA, DCM, 0 °C to RT, 12 h, 95%, or comp. 14, 21 eq. DIPEA, 4.5 eq. PyBOP, 0.8 eq. DMAP, DCM, 0 °C to RT, 12 h, 81%; h) H_2, 10% Pd/C, EtOH/EtOAc, 12 h, 92%.

Comp. 12

The template was prepared essentially according to the published literature procedure [6] without modification. In brief, dibutyltin (IV) oxide (50 g, 0.2 mol, 1 eq.), ethylene glycol (12.67 g, 0.2 mol, 1.02 eq.) were refluxed in benzene (0.5 L) overnight in a flask fitted with a condenser and a Dean-Stark trap. The reaction solution was allowed to reach room temperature and then cooled using an ice-water bath. The resulting solid was collected by filtration, washed with small portions of benzene, and dried in vacuo. Yield: 58.04 g (99%).

Comp. 13

The synthesis of **13** was carried out according to the published literature with minor modifications.[9] A solution of 2,3-dihydroxybenzoic acid (2 g, 12.98 mmol, 1 eq.) in acetone (100 mL) was treated with K_2CO_3 (30 g, 217.07 mmol, 16.7 eq.) and benzyl bromide (12.442 g, 72.74 mmol, 5.6 eq.). The reaction mixture was heated overnight at reflux under nitrogen and then was allowed to cool to room temperature. The resultant precipitate was filtered off and washed with acetone. The filtrates were combined, the organic volatiles were removed in high vacuum. The crude product was further purified by flash chromatography (hexane/EtOAc, gradient, 0-20% EtOAc) affording the benzyl 2,3-bis(benzyloxy)benzoate. Yield: 5.4 g (98 %).

Comp. 14

Powdered potassium hydroxide (2.141 g, 38.16 mmol, 3 eq.) was added in small portions to ice-cold solution of **13** (5.4 g, 12.72 mmol) in ethanol (200 mL). The reaction mixture was then heated at reflux until TLC indicated the starting material was consumed. The mixture was allowed to cool to ambient temperature, and the organic solvent was removed by rotary evaporation. The residue was suspended in water (100 mL), and the aqueous layer was first extracted with diethyl ether (3 × 50 mL), and the organic extracts were discarded. The aqueous layer was then acidified with 2M HCl (25 mL), and the resultant bulky precipitate was extracted with EtOAc (2 × 100 mL). The combined organic layers were combined, dried over Mg_2SO_4, filtered and the solvent was removed *in vacuo* to provide the title product as a white solid which was used in the next step without purification. Yield: 3.7 g (87%).

Comp. 15

Comp. **15** was prepared by following a similar literature protocol.[10] Thionyl chloride (1 mL, 13.8 mmol, 34.8 eq.) was added to a solution of acid **14** (530 mg, 1.6 mmol, 4 eq.) in anhydrous benzene (20 mL), and the mixture was

heated at reflux for 30 min. The volatiles were removed under reduced pressure, and the residue was dried under high vacuum to give **15** as a waxy solid which was taken to the next step without further purification.

Comp. 16

The trityl trilactone was obtained following the literature protocol with minor modifications.[6]

A) Using toluene as a solvent. Trt-*L*-Ser-OMe (5 g, 13.834 mmol, 1 eq.) and stannoxane **12** (1.2 g, 2.048 mmol, 0.15 eq.) were placed in a 0.5 L flask equipped with a condenser and a Dean-Stark trap containing 4 Å molecular sieves. The reaction mixture was refluxed under nitrogen in toluene for 72 h. After cooling, the solvent was removed under reduced pressure. The obtained residue was dissolved in dichloromethane, applied to a silica gel plug (dry loading), and purified by flash column chromatography using gradient elution from dichloromethane/cyclohexane (2:1, v/v) to 1% MeOH in DCM. Evaporation of the solvent induced self-crystallization of the product as a white solid (mp > 240°C with decomp.). Yield: 2 g (44%). ^{1}H and ^{13}C NMR data were in full agreement with those previously reported.[5]

^{1}H NMR (300 MHz, $CDCl_3$): δ 7.43 (dd, *J* = 8.0, 1.4 Hz, 18H), 7.33 – 7.20 (m, 27H), 4.04 (t, *J* = 11.1 Hz, 3H), 3.53 – 3.37 (m, 6H), 2.62 (d, *J* = 10.2 Hz, 3H).

^{13}C NMR (75 MHz, $CDCl_3$): δ 172.50, 145.42, 128.78, 128.14, 126.75, 77.16, 71.46, 66.77, 54.56.

B) Using xylene as a solvent. Trt-*L*-Ser-OMe (10 g, 27.667 mmol) and stannoxane **12** (820 mg, 1.399 mmol, 0.05 eq.) were placed in a 1 L flask equipped with a condenser and a Dean-Stark trap containing 4 Å molecular sieves. The reaction mixture was refluxed under nitrogen in toluene for 48 h. After cooling, the solvent was removed under reduced pressure and the target product was purified as described above. Yield: 8.7 g (95%). Analytical data are consistent with those reported above.

Comp. 17: Triserine trilactone trihydrochloride

Under a nitrogen atmosphere, TFA (5 mL, 65 mmol, 32 eq.) was added dropwise via a syringe to an ice-cooled solution of tris(*N*-trityl-*L*-serine) trilactone **16** (2 g, 2.024 mmol) in dichloromethane (10 mL). The reaction mixture was stirred for 1 h, after which the organic solvents were removed under reduced pressure. The residue was co-evaporated with ethanol (3 × 25 mL) to ensure complete removal of TFA. Separately, in a 50 mL flask, acetyl chloride (1.6 mL, 22.422 mmol) was added dropwise to 25 mL of ice-cooled anhydrous ethanol according to the literature protocol.[5] An aliquot of this solution (14 mL) was added to the residue obtained by TFA treatment and removal, and the mixture was stirred for additional 30 min. The solvent was then removed by rotary evaporation. Diethyl ether (25 mL) was added to the solid, which was then quickly collected by filtration, washed with small portions of diethyl ether, and dried under high vacuum to afford the title product as a white solid. Yield: 746 mg (99%).

Comp. 18

A) Acyl chloride method. The freshly prepared benzoyl chloride **15** (1.6 mmol) was dissolved in anhydrous dichloromethane (10 mL) and added dropwise over 10 min *via* syringe to an ice-cooled, stirred solution of lactone **17** (147 mg, 0.4 mmol) and DIPEA (691 µL, 4 mmol, 10 eq.) in dichloromethane (25 mL). The mixture was allowed to stir overnight while warming to room temperature. The organic solvents were evaporated *in vacuo*, and the residue was extracted into EtOAc (100 mL). The organic layer was washed successively with 5% aqueous citric acid, saturated aqueous sodium bicarbonate, brine, dried over $MgSO_4$, filtered, and concentrated under reduced pressure. The crude product was purified by flash chromatography on silica gel (DCM/EtOAc, gradient 5–30% EtOAc) to give the title compound as a white solid. Yield: 456 mg (95%). The ^{1}H NMR data agreed with those reported in literature.[6]

^{1}H NMR (300 MHz, $CDCl_3$): δ 8.46 (d, *J* = 7.3 Hz, 3H), 7.71 – 7.62 (m, 3H), 7.50 – 7.04 (m, 36H), 5.20 – 5.00 (m, 12H), 4.90 (tt, *J* = 11.1, 5.5 Hz, 3H), 4.16 (dd, *J* = 10.8, 4.6 Hz, 3H), 4.00 (dt, *J* = 22.9, 11.5 Hz, 3H).

^{13}C jmod NMR (75 MHz, $CDCl_3$): δ 169.21 (C=O), 165.06 (C=O), 151.79 (C_q), 147.14 (C_q), 136.42 (C_q), 136.17 (C_q), 129.05 (CH), 128.77 (CH), 128.61 (CH), 128.35 (CH), 127.78 (CH), 126.54 (C_q), 124.43 (CH), 123.36 (CH), 117.77 (CH), 76.46 (CH_2), 71.41 (CH_2), 64.30 (CH_2), 51.60 (CH).

B) Phosphonium-based method. Under a nitrogen atmosphere, DIPEA (2.2 g, 17.2 mmol, 21.3 eq.) was added dropwise to a stirred, ice-cooled suspension of triseryl salt **17** (300 mg, 0.8 mmol) in anhydrous dichloromethane (20 mL). Acid **14** (1.2 g, 3.6 mmol, 4.5 eq.) and DMAP (80 mg, 0.7 mmol, 0.8 eq.) were then added in one portion, followed by PyBOP (1.9 g, 3.6 mmol, 4.5 eq.). The reaction mixture was stirred overnight while warming to room temperature. The reaction mixture was then subjected to the same workup and purification sequence as described above, yielding the title compound as a white solid; analytical data matched those described above. Yield: 790 mg (81%).

C) Carbodiimide method. Under a nitrogen atmosphere, DIPEA (2.63 mL, 15.1 mmol, 10.1 eq.) was added dropwise to a stirred, ice-cooled suspension of triseryl salt **17** (555 mg, 1.5 mmol) in anhydrous dichloromethane (20 mL). Acid **14** (2.01 g, 6.0 mmol, 4 eq.), HOBt (960 mg, 7.1 mmol, 4.8 eq.) and DMAP (20 mg, 0.16 mmol, 0.1 eq.) were then added in one portion, followed by dropwise addition of DIC (1.41 mL, 9 mmol, 6 eq.). The reaction mixture was stirred overnight while warming to room temperature. The workup procedure and purification were carried out as described above to afford the title compound as a white solid. All measured physicochemical and analytical data matched those reported for the product described above. Yield: 1.21 g (67%).

Comp. 1: Enterobactin

The protected enterobactin **18** (1.2 g, 0.99 mmol) was hydrogenated over 10% palladium on carbon (*ca.* 0.7 g) in a mixture of EtOH/EtOAc (1:1, v/v) for 12 h using *in situ* generated hydrogen (zinc powder/2M aqueous hydrochloric acid) in a Coware® apparatus (1.0-5.0 mmol size).[11] After completion, the catalyst was removed by centrifugation, the filtrate was evaporated, and the obtained residue was dried in high vacuum to yield comp. **1** as white foam (612 mg, 92%). Formation of the title product was confirmed by LC-MS and NMR, and the data were consistent with literature values.

^{1}H NMR (300 MHz, DMSO-d_6): δ 9.45 (s, 2H), 9.14 (d, *J* = 6.8 Hz, 3H), 7.36 (d, *J* = 7.6 Hz, 3H), 6.98 (d, *J* = 7.3 Hz, 3H), 6.73 (t, *J* = 7.9 Hz, 3H), 5.01 – 4.82 (m, 3H), 4.65 (t, *J* = 9.9 Hz, 3H), 4.41 (dd, *J* = 10.8, 4.3 Hz, 3H).

^{13}C NMR (75 MHz, DMSO-d_6): δ 169.48, 168.98, 148.64, 146.21, 119.31, 118.44, 118.37, 115.34, 63.50, 51.35.

Synthesis of TRENCAM

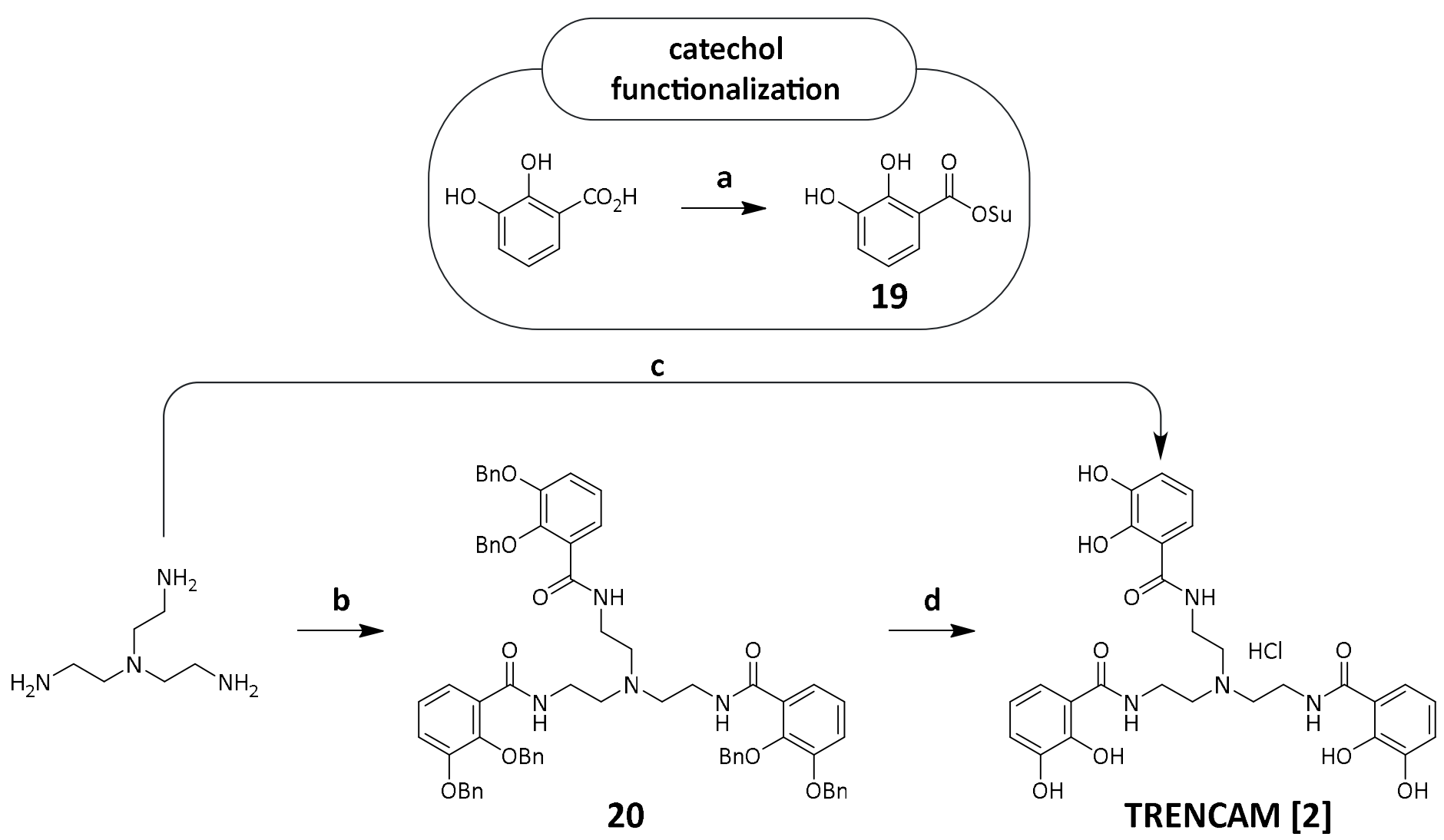

Scheme 2. Synthesis of TRENCAM according to adopted literature procedures,[12,13] reagents and conditions: a) 1 eq. NHS, 1.2 eq. DCC, THF, 16 h, 28%; b) 4 eq. comp. **14**, 4 eq. HOBt, 6 eq. DIPEA, 0.4 eq. DMAP, 6 eq. EDAC, DCM, 0 °C to RT, 12 hr, 82%; c) 4 eq. DIPEA, 3.2 eq. comp **19**, DMF, 12 h, 74%; d) H_2, 10% Pd/C, EtOH/EtOAc, 16h, 92%.

Comp. 19

Using commercially available 2,3-dihydroxybenzoic acid (4 g, 25.95 mmol, 1.00 eq.), *N*-hydroxysuccinimide (3 g, 26.07 mmol, 1 eq.), DCC (6.42 g, 31.12 mmol, 1.2 eq.) and THF as a solvent, the synthesis of the title compound was carried out according to the published protocol.[14] Yield: 1.82 g (28%).

Comp. 20

Under nitrogen, a solution of tris(2-aminoethyl)amine (289 mg, 1.98 mmol), acid **14** (2.644 g, 7.91 mmol, 4 eq.), and HOBt (1.068 g, 7.9 mmol, 4 eq.) in 30 mL anhydrous dichloromethane was cooled with an ice-water bath. While stirring, DIPEA (1.532 g, 11.85 mmol, 6 eq.) and DMAP (96 mg, 0.77 mmol, 0.4 eq.) were added followed by EDAC

(2.273 g, 11.86 mmol, 6 eq.). The reaction mixture was stirred overnight while warming to room temperature. The volatiles were removed by rotary evaporation, and the obtained residue was extracted into EtOAc (100 mL). The organic layer was washed with sodium bicarbonate, brine, dried over anhydrous $MgSO_4$, and evaporated under reduced pressure. The obtained crude viscous oil was purified by flash chromatography using gradient elution staring from 30% EtOAc in DCM to 10% MeOH in DCM to obtain the title product as a white gum after drying in high vacuum. Yield: 1.78 g (82%).

^{1}H NMR (300 MHz, $CDCl_3$): δ 7.80 (t, *J* = 5.3 Hz, 3H), 7.66 – 7.57 (m, 3H), 7.46 – 7.31 (m, 15H), 7.31 – 7.19 (m, 17H), 7.09 – 7.05 (m, 4H), 5.08 (s, 6H), 5.02 (s, 6H), 3.15 (dd, *J* = 12.4, 6.3 Hz, 6H), 2.31 (t, *J* = 6.7 Hz, 6H).

^{13}C jmod NMR (75 MHz, $CDCl_3$): δ 165.33 (C=O), 151.78 (C_q), 146.82 (C_q), 136.64 (C_q), 136.58 (C_q), 128.73 (CH), 128.69 (CH), 128.27 (CH), 127.92 (C_q), 127.68 (CH), 124.38 (CH), 123.17 (CH), 116.85 (CH), 76.34 (CH_2), 71.27 (CH_2), 52.61 (CH_2), 37.38 (CH_2).

Comp. 2: TRENCAM

A) Under nitrogen atmosphere, DIPEA (517 mg, 4 mmol, 4 eq.) was added to ice-cold solution of tris(2-aminoethyl)amine (146 mg, 1 mmol, 1 eq.) in anhydrous DMF. Comp. **19** (804 mg, 3.2 mmol, 3.2 eq.) was added in one portion and stirring was continued overnight. The reaction mixture was concentrated under reduced pressure. The residue was then dissolved with 5 molar equivalents of hydrochloric acid in DI water/acetonitrile (4:1, v/v), filtered and purified by reverse-phase chromatography with water and acetonitrile (with 0.1% TFA) as eluents to give the title product as a white solid after lyophilization. Yield: 435 mg (74%).

^{1}H NMR (300 MHz, DMSO-d_6): δ 12.16 (s, 3H), 9.96 (br. s, 1H), 9.34 (s, 2H), 8.99 (t, *J* = 6.0 Hz, 3H), 7.25 (dd, *J* = 8.1, 1.3 Hz, 3H), 6.96 (dd, *J* = 7.8, 1.3 Hz, 3H), 6.71 (t, *J* = 7.9 Hz, 3H), 3.71 (br. m, 6H), 3.48 (br. s, 6H).

^{13}C jmod NMR (75 MHz, DMSO) δ 170.10 (C=O), 149.16 (C_q), 146.26 (C_q), 131.46 (C_q), 119.06 (CH), 118.21 (CH), 117.66 (CH), 115.29 (C_q), 51.41 (CH_2), 34.10 (CH_2).

B) Comp. **20** (2.86 g, 2.61 mmol) was hydrogenated over 10% palladium on carbon in a mixture of EtOH/EtOAc for 16 h using *in situ* generated hydrogen in a Coware® apparatus (1.0-5.0 mmol size). After completion, the catalyst was removed by filtration through Celite, and the filtrate was evaporated under vacuum. The obtained residue was suspended in water, acidified with 1 M HCl (5.4 mL) and lyophilized. Yield: 1.112 g (72%) of the title comp. **2** as a white solid. The spectroscopic data were identical with those of the product described above.

Synthesis of Meropenem-functionalized linkers 4 and 5

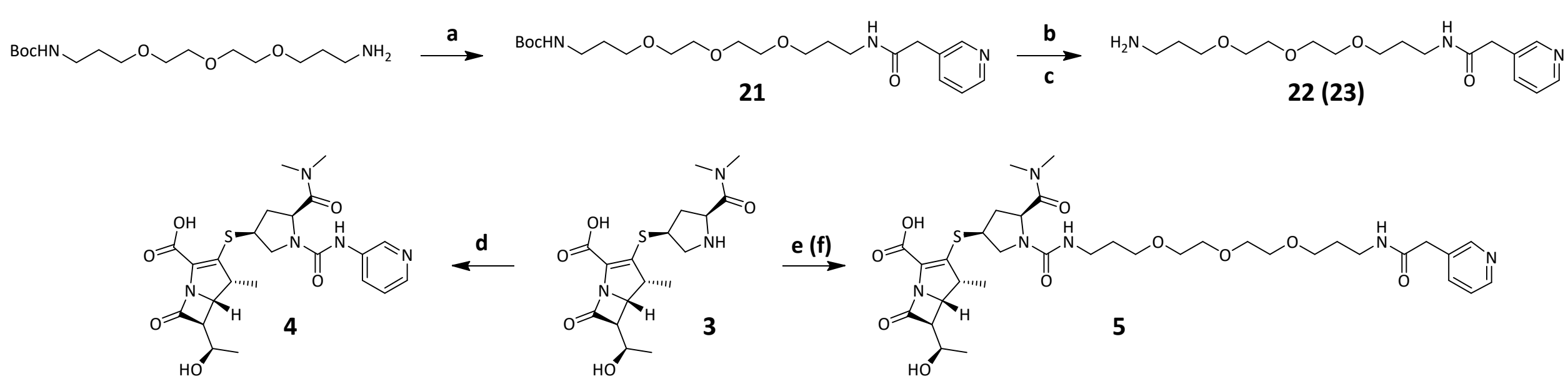

Scheme 3. Synthesis of the pyridine- and meropenem-functionalized ligands 4 and 5, reagents and conditions: a) 6 eq DIPEA, 1.5 eq 2-(pyridin-3-yl)acetic acid, 1.8 eq HOBt, 0.1 eq DMAP, 1.5 eq EDAC, DMF, 0 °C to rt, 94 %; b) 19 eq TFA, DCM, 0 °C to rt, 1 hr / 6 eq HCl in EtOH, 85% (over two steps) for 22 (HCl salt); c) 18.8 eq $NaHCO_3$, quant for 23 (free base); d) 2 eq 3-aminopyridine, 2.3 eq CDI, 1 eq. comp. 3, 4.8 eq Et_3N, 1 eq potassium 2-ethylhexanoate (KEHA), DMSO, rt, 44%; e) 1 eq comp. 22, 9 eq DIPEA, 1.2 eq DSC, $CHCl_3$, 0 °C, 1h / 1.4 eq. comp. 3, 1.4 eq KEHA, $CHCl_3$/DMSO, 0 °C to rt, 1h, 53%; f) 1 eq comp. 23, 1 eq DSC, $CHCl_3$, 0 °C, 1h / 1 eq. comp. 3, 1 eq KEHA, $CHCl_3$/DMSO, 0 °C to rt, 1h, 35%.

Comp. 21

The title compound was prepared by adopting a similar literature protocol.[15] Under a nitrogen atmosphere, DIPEA (4.2 mL, 24.0 mmol, 6 eq) was added dropwise to an ice-cooled solution of NH_2-PEG_3-C_2-Boc (1.282 g, 4 mmol), 2-(pyridin-3-yl)acetic acid (1.041 g, 6 mmol, 1.5 eq), HOBt (973 mg, 7.2 mmol, 1.8 eq), and DMAP (49 mg, 0.4 mmol, 0.1 eq) in anhydrous DMF (25 mL). After that, EDAC (1.15 g, 6 mmol,

1.5 eq) was added to the reaction mixture in one portion, and the reaction mixture was stirred overnight while warming to room temperature. The volatiles were removed using rotary evaporation, and the obtained residue was extracted into EtOAc. The organic layer was washed with aqueous sodium bicarbonate, brine, dried over anhydrous $MgSO_4$, and evaporated under reduced pressure to give the title product as a yellowish oil in quantitative yield. The material was judged to be of sufficient purity by proton NMR and was used directly in the next step without purification. The reaction was repeated several times on a twofold larger scale to afford comp. **21** in yields of ca. 3.2 g (94%).

^{1}H NMR (300 MHz, DMSO-d_6): δ 8.48 – 8.38 (m, 1H), 8.08 (t, *J* = 5.3 Hz, 1H), 7.65 (dt, *J* = 7.8, 1.9 Hz, 1H), 7.32 (ddd, *J* = 7.8, 4.8, 0.6 Hz, 1H), 6.74 (t, *J* = 5.2 Hz, 1H), 3.53 – 3.41 (m, 10H), 3.37 (td, *J* = 6.3, 3.3 Hz, 4H), 3.33 (s, 1H), 3.09 (dd, *J* = 12.6, 6.8 Hz, 2H), 3.02 – 2.85 (m, 2H), 1.68 – 1.53 (m, 4H), 1.37 (s, 9H).

^{1}H NMR (300 MHz, $CDCl_3$): δ 8.54 – 8.49 (m, 2H), 7.70 (dt, *J* = 7.8, 1.9 Hz, 1H), 7.29 – 7.23 (m, 1H), 6.60 (br. s, 1H), 5.05 (br. s, 1H), 3.66 – 3.48 (m, 14H), 3.37 (dd, *J* = 12.2, 5.8 Hz, 2H), 3.26 – 3.14 (m, 2H), 1.81 – 1.68 (m, 4H), 1.44 (s, 9H).

^{13}C NMR (75 MHz, $CDCl_3$): δ 169.89, 156.19, 150.33, 148.47, 136.92, 131.37, 123.62, 77.16, 70.58, 70.35, 70.27, 70.11, 69.54, 40.73, 38.59, 38.50, 29.79, 28.75, 28.55.

Comp. 22 (HCl salt)

Under a nitrogen atmosphere, TFA (6.7 mL, 87.7 mmol, 19.26 eq) was added dropwise via a syringe to an ice-cooled solution of Pyr-NH-PEG$_3$-C$_2$-Boc (2 g, 4.6 mmol) in dichloromethane (20 mL). The reaction mixture was stirred for 1 h, after which the organic solvents were removed under reduced pressure. The residue was co-evaporated with ethanol (3 × 25 mL) to ensure complete removal of TFA. Separately, in a 50 mL flask, acetyl chloride (1.6 mL, 22.422 mmol) was added dropwise to 25 mL of ice-cooled anhydrous ethanol according to the literature protocol.[5] The obtained ethanolic HCl was added to the residue obtained by TFA treatment, and the mixture was stirred for additional 30 min. The solvent was then removed by rotary evaporation, and the residue additionally co-evaporated with ethanol (3 × 25 mL) and dried in high vacuum to give the title product as a pale-yellow viscous oil. Yield: 1.593 g (85%).

Comp. 23 (free base)

Comp. **22** (1.3 g, 3.2 mmol) was dissolved in deionized water (20 mL) and neutralized by the gradual addition of powdered sodium bicarbonate (5 g, 59.5 mmol, 18.8 eq). The mixture was lyophilized, and acetonitrile extraction (3×35 mL) followed by centrifugation was employed to remove insoluble salts. The combined organic layers were evaporated and dried under high vacuum to afford the free amine as a colorless viscous oil in quantitative yield. The product was stored at –20 °C.

^{1}H NMR (300 MHz, $CDCl_3$): δ 8.51 (d, *J* = 1.9 Hz, 1H), 8.49 (dd, *J* = 4.8, 1.6 Hz, 1H), 7.69 (dt, *J* = 7.8, 1.8 Hz, 1H), 7.26 (ddd, *J* = 7.8, 4.8, 0.6 Hz, 1H), 7.20 (br. s, 1H), 3.72 – 3.44 (m, 14H), 3.41 – 3.29 (m, 2H), 1.83 – 1.66 (m, 4H).

^{13}C NMR (75 MHz, $CDCl_3$): δ 169.97, 150.19, 148.18, 136.78, 131.44, 123.46, 77.16, 70.37, 69.99, 69.92, 69.75, 69.54, 40.46, 39.61, 37.92, 32.58, 28.70.

Comp. 4

With stirring, a solution of 3-aminopyridine (113 mg, 1.2 mmol, 2 eq.) in dry DMSO (5mL) was treated with CDI (227 mg, 1.4 mmol, 2.3 eq.) under nitrogen in a cooled water bath (≈ 20 °C). The mixture was stirred for 2h min at this temperature, after which **3** (262 mg, 0.6 mmol, 1 eq.) was added in a single portion, followed by the dropwise addition of triethylamine (292 mg, 2.89 mmol, 4.8 eq.) and KEHA (109 mg, 0.6 mmol, 1 eq.). The reaction was then allowed to warm to room temperature and stirred for 1 h. The mixture was filtered, carefully diluted with ice-cold water-acetonitrile (4:1, v/v) to a total volume of 10 mL, and

purified in two injections by reverse-phase HPLC (C_{18} column) using acid-free H_2O/MeCN as eluents. The target compound was obtained as a white solid after lyophilization. Yield: 132 mg (44%).

^{1}H NMR (300 MHz, DMSO-d_6): δ 8.65 (d, J = 2.5 Hz, 1H), 8.54 (s, 1H), 8.13 (d, J = 4.6 Hz, 1H), 7.88 (d, J = 8.5 Hz, 1H), 7.25 (dd, J = 8.3, 4.7 Hz, 1H), 4.85 (t, J = 8.0 Hz, 1H), 4.29 – 4.10 (m, 2H), 4.01 – 3.90 (m, 1H), 3.81 (s, 1H), 3.68 – 3.52 (m, 2H), 3.32 – 3.20 (m, 3H), 3.06 (s, 3H), 2.81 (s, 3H), 1.21 – 1.09 (m, 6H).

^{13}C NMR (75 MHz, DMSO-d_6): δ 174.04 (C=O), 171.08 (C=O), 161.98 (C=O), 152.89 (C=O), 146.32 (C_q), 142.65 (CH), 140.88 (CH), 136.97 (C_q), 126.23 (C_q), 125.86 (CH), 123.21 (CH), 64.18 (CH), 59.66 (CH), 55.97 (CH), 55.24 (CH), 54.98 (CH_2), 42.54 (CH), 39.52 (CH), 36.45 (CH_3), 35.36 (CH_3), 34.41 (CH_2), 21.76 (CH_3), 16.96 (CH_3).

Comp. 5

b) By the reaction with the hydrochloride salt **22**. Under a nitrogen atmosphere, comp. **22** (175 mg, 0.43 mmol) was suspended in 20 mL of anhydrous chloroform, and the solution was cooled down with an ice-water bath. DIPEA (494 mg, 3.82 mmol, 9 eq) was added dropwise to the stirred solution, followed by DSC (130 mg, 0.51 mmol, 1.2 eq) in one portion. The reaction mixture was allowed to react for 1 hr at 0°C. After that, powdered Meropenem (260 mg, 0.59 mmol, 1.40 eq) was added in one portion followed by dropwise addition of KEHA (108 mg, 0.59 mmol, 1.4 eq) in 0.8 mL of anhydrous DMSO. The reaction mixture was stirred for 1 hr while warming to room temperature, and concentrated *in vacuo*. The obtained concentrated DMSO solution was further stirred for 0.5 hr, dissolved in 10 mL of ice-cold solution of DI water and acetonitrile (4:1, v/v). The resultant solution was filtered and the title product was purified by reverse-phase HPLC (C_{18}) using acid-free H_2O/MeCN as eluents, affording the target compound as a white solid after lyophilization. Yield: 168 mg (53%). Formation of the title product was confirmed by LC-MS and NMR spectra. LC-MS (ESI$^+$): [M+H]$^+$ m/z calcd. for $C_{35}H_{52}N_6O_{10}S$, 749.35; found, 749.33.

^{1}H NMR (300 MHz, DMSO-d_6): δ 8.44 (d, J = 1.7 Hz, 1H), 8.42 (dd, J = 4.8, 1.5 Hz, 1H), 8.31 (t, J = 5.1 Hz, 1H), 7.67 (dt, J = 8.0, 1.8 Hz, 1H), 7.31 (dd, J = 8.0, 4.8 Hz, 1H), 6.20 (t, J = 5.2 Hz, 1H), 5.00 (br. s, 1H), 4.67 (t, J = 7.9 Hz, 1H), 3.97 (dd, J = 9.2, 2.2 Hz, 1H), 3.93 – 3.78 (m, 2H), 3.62 – 3.36 (m, 20H), 3.19 – 2.95 (m, 10H), 2.79 (s, 3H), 1.62 (s, 4H), 1.15 (d, J = 6.2 Hz, 3H), 1.05 (d, J = 7.0 Hz, 3H).

^{13}C NMR (75 MHz, DMSO-d_6): δ 172.69, 171.97, 169.47, 163.73, 155.63, 149.99, 147.54, 136.52, 132.29, 123.31, 69.75, 69.52, 68.39, 67.99, 64.68, 58.73, 55.65, 55.39, 54.29, 41.53, 39.95, 39.28, 37.32, 36.44, 35.97, 35.33, 35.12, 30.04, 29.24, 21.87, 16.61.

A) By the reaction with the free base **23**. Di(*N*-succinimidyl)carbonate (71 mg, 0.27 mmol) was added in one portion to a solution of linker **23** (94 mg, 0.27 mmol) in $CHCl_3$ (10 mL) at 0°C and stirred for 1 hr. Comp. **3** (118 mg, 0.27 mmol) was then added in one portion, followed by the dropwise addition of a solution of KEHA (49 mg, 0.27 mmol) in anhydrous DMSO (2 mL). The reaction mixture was then allowed to warm to room temperature with stirring continued for 1 hour. The solvent was then concentrated under reduced pressure, and the resulting residue was dissolved in 10 mL of ice-cold solution of DI water and acetonitrile (4:1, v/v). The title product was purified by reverse-phase HPLC (C_{18}) using acid-free H_2O/MeCN as eluents. The compound was collected at 22% acetonitrile (0.1% TFA in water) to give a white solid after lyophilization. Yield: 70 mg (35%). The analytical data of the obtained product were identical to those reported above.

Synthesis of Meropenem-siderophore conjugates 7 and 8

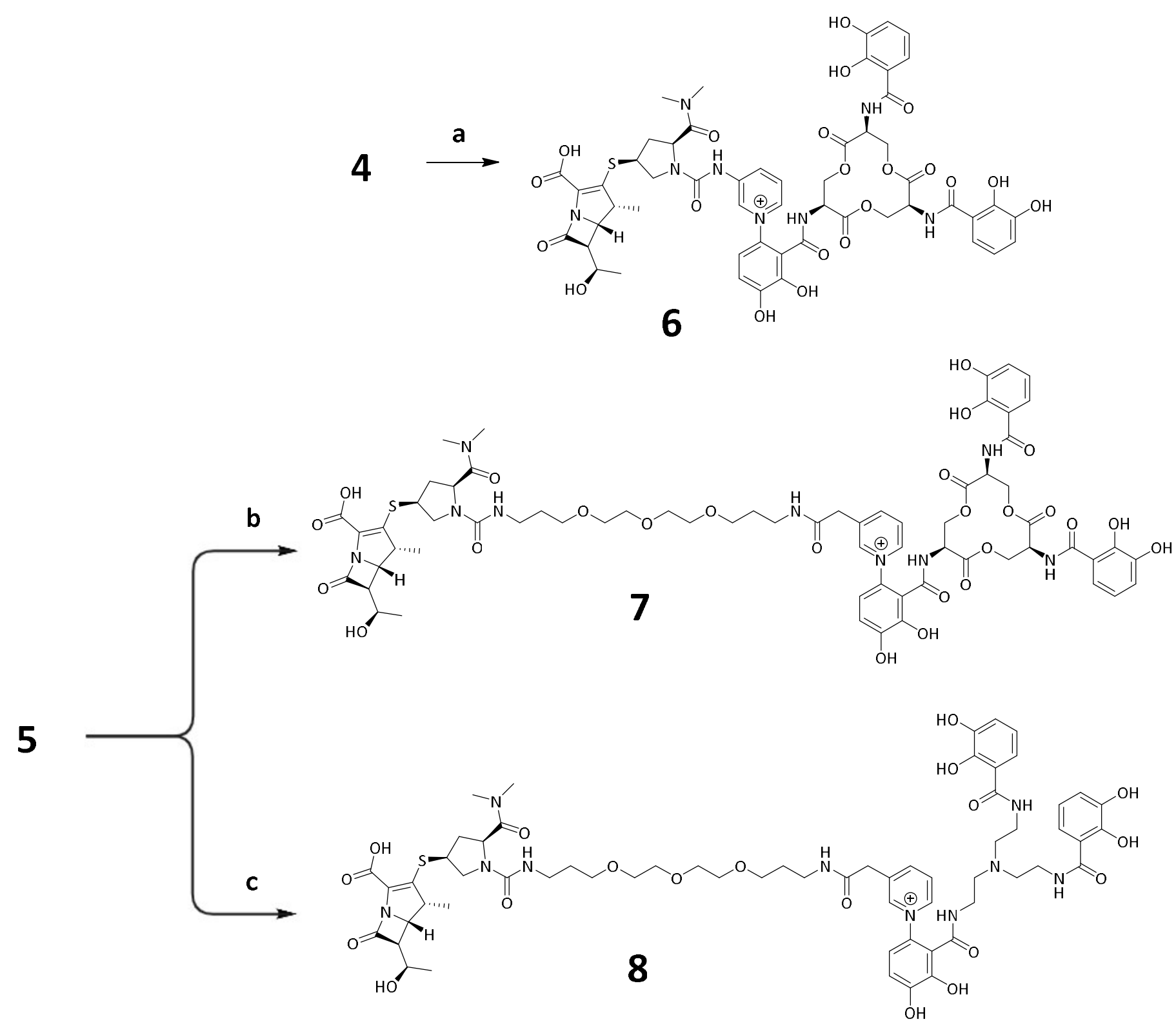

Scheme 4. Synthesis of Meropenem-conjugated siderophores, reagents and conditions: a) 1 eq Ent, 2.4 eq AgEHA, 1 eq comp. 4, 1 eq I_2, DMSO, rt, 1 hr, 8%; b) 1 eq Ent, 2 eq silver 2-ethylhexanoate (AgEHA*), 1 eq comp. 5, 1 eq I_2, DMSO, rt, 1 hr, 15%; c) 1.6 eq comp. 2, 2 eq AgEHA, 1 eq I_2, DMSO, rt, 21%. *Substitution of AgEHA by STF led to β-lactam ring opening during the coupling reaction, affording product 7b.

Silver 2-ethylhexanoate (AgEHA)

Silver 2-ethylhexanoate was obtained following the general procedure of Hsu and co-workers[16] by mixing KEHA (208 mg, 1.141 mmol, 1 eq.) and silver nitrate (194 mg, 1.142 mmol, 1 eq.) in H_2O (30 mL). The precipitate was collected by filtration, washed with distilled water and methanol, and then dried in high vacuum. The product was obtained as a white sticky solid after drying in high vacuum. Yield: 254 mg (87%).

Comp. 6

Using Enterobactin (**1**) (16.4 mg, 0.024 mmol, 1 eq.), comp. **4** (12 mg, 0.024 mmol, 1 eq.), AgEHA (14 mg, 0.056 mmol, 2.4 eq.), iodine (6 mg, 0.024 mmol, 1 eq.), the title comp. was prepared as a white solid by following the same procedure described for **7** (see the protocol below). Yield: 2.3 mg (8%). HRMS (ESI): *m/z* $[M]^+$ calcd for $C_{53}H_{55}N_8O_{21}S^+$ 1171.3197, found 1171.3205.

Comp. 7

Comp. **1** (31.5 mg, 0.047 mmol) and comp. **5** (35 mg, 0.047 mmol) were suspended in DMSO (0.6 mL) in a 2 mL Eppendorf plastic test tube. A solution of AgEHA (24 mg, 0.094 mmol) in DMSO (0.3 mL) was added dropwise to the mixture, followed by gentle shaking to ensure proper mixing. The resulting solution was kept at room temperature for 5 min with additional shaking (2-3 times). With vigorous stirring applied, a solution of iodine (12.1 mg, 0.047 mmol) in DMSO (0.2 mL) was added dropwise, and the reaction mixture

was left at room temperature for 1 h with stirring. The reaction mixture was then centrifuged, the supernatant was diluted to a total volume of 5 mL using water-acetonitrile (4:1, v/v) and purified by reverse-phase HPLC (C_{18}) using acid-free H_2O/MeCN as eluents to afford the target compound as a white solid. Yield: 10 mg (15%). HRMS (ESI): *m/z* $[M]^+$ calcd for $C_{65}H_{78}N_9O_{25}S^+$ 1416.4830; found 1416.4878.

Comp. 7b

Comp. **1** (20 mg, 0.03 mmol) and comp. **5** (22.3 mg, 0.03 mmol, 1 eq.) were reacted in the presence of STF (13.1 mg, 0.07 mmol, 2.2 eq.) and iodine (7.5 mg, 0.030 mmol, 1 eq.) in DMSO using the same procedure described above for comp. **7** to give the title product as a white solid after purification by reverse phase HPLC with water/acetonitrile (0.1% TFA) gradient. Yield: 14.27 mg (33%). The compound was identified as a hydrolyzed β-lactam product by low-resolution LC-MS and was therefore excluded from further analytical and biological studies.

Comp. 8

A) 1.6:1 ratio of comp. **2**/comp. **5**. Using comp. **2** (85 mg, 0.14 mmol, 1.6 eq.), comp. **5** (67 mg, 0.09 mmol, 1 eq.), AgEHA (45 mg, 0.18 mmol, 2 eq.), and iodine (23 mg, 0.09 mmol, 1 eq.), the title compound was prepared as a white solid by following the same procedure described for **7**. Yield: 26 mg (21%). HRMS (ESI): *m/z* $[M]^+$ calcd for $C_{62}H_{81}N_{10}O_{19}S^+$ calculated 1301.5395, found 1301.5378.

B) 1:1 ratio of comp. **2**/**5**. Using comp. **2** (20 mg, 0.03 mmol, 1 eq.), comp. **5** (25 mg, 0.03 mmol, 1 eq.), AgEHA (17 mg, 0.07 mmol, 2 eq.), and iodine (8.6 mg, 0.03 mmol, 1.00 eq.), the title comp. was prepared as a white solid by following the same procedure described for **7**. Yield: 5.7 mg (12%). The isolated compound exhibited analytical data identical to those of the product described above.

Synthesis of comp. 9 (PEG-linked indole–pyridyl analogue)

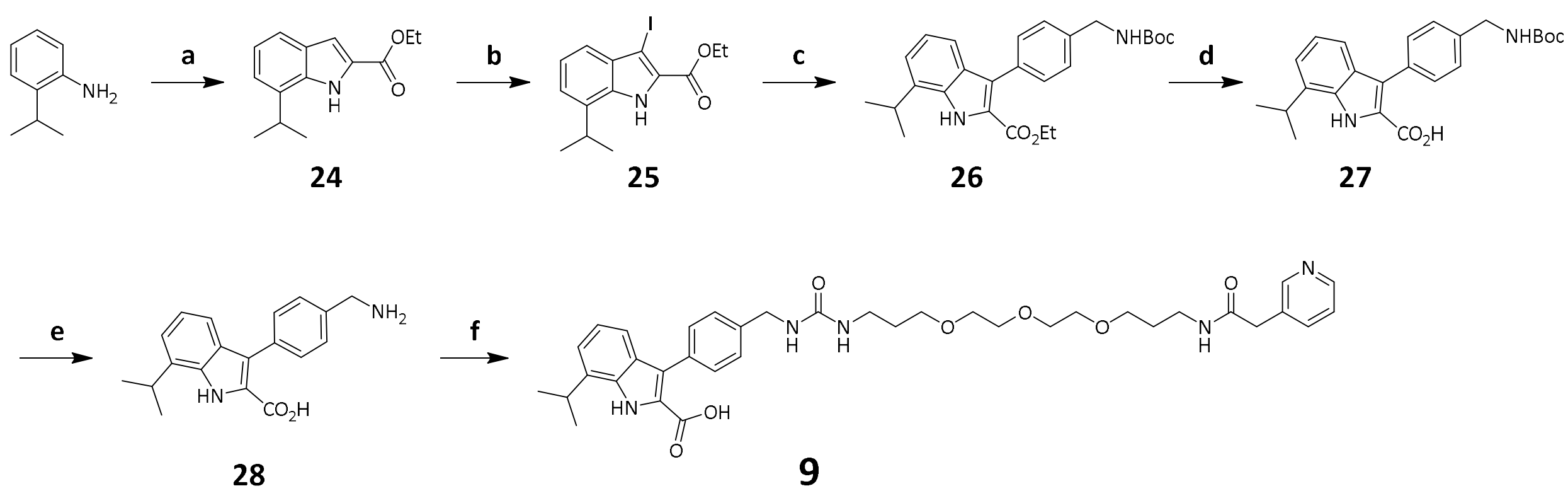

Scheme 5. Synthesis of 9, reagents and conditions: a) 1 eq $NaNO_2$, 4 eq. conc. HCl, water, 0 °C, 10 min; 1.1 eq. ethyl-2-methylacetoacetate, 3.5 eq KOH, EtOH, rt, 1 h; 1.1 eq p-TsOH, toluene, reflux, overnight, 15%; b) 2 eq NIS, DCM, rt, 78%; c) 1.3 eq pinacol ester of 4-(tert-butoxycarbonylaminomethyl)phenylboronic acid, 5.8 eq K_2CO_3, 0.06 eq $Pd(dppf)Cl_2$, water/acetonitrile, 80 °C, 1.5 hr, quant.; d) 7.5 eq KOH, EtOH, reflux, 1 hr / H^+ (aq), 93%; e) TFA, DCM, 0 °C to RT, 1 h / 2.5 eq HCl in EtOH, 60%; f) 1.4 eq comp. 22, 17 eq DIPEA, 1.3 eq DSC, 1 eq KEHA, 1.1 eq DBU, $CHCl_3$/DMSO, 0 °C to RT, 2hr, 91%.

Comp. 24, ethyl 7-isopropyl-*1H*-indole-2-carboxylate

The title compound was prepared as a colorless oil following a literature procedure[17] using *o*-aminocumene (4.2 g, 31.1 mmol), hydrochloric acid (42 mL, conc. 11%, 126.7 mmol, 4 eq), sodium nitrite (2.07 g, 30 mmol, 1 eq, as a 1M aqueous soln), ethyl-2-methylacetoacetate (4.4 g, 30.5 mmol, 1 eq),

potassium hydroxide (6.17 g, 110 mmol, 3.5 eq, as a 10 M aqueous soln), and toluenesulfonic acid (5.71 g, 33.16 mmol, 1.1 eq). Yield: 1.1 g (15%). A threefold scale-up of this reaction with *o*-aminocumene (12.61 g, 93.2 mmol) afforded the target compound **24** (3.26 g) in comparable yield.

Comp. 25, ethyl 3-iodo-7-isopropyl-*1H*-indole-2-carboxylate

The title compound was prepared following the literature procedure[17] with little modification. Under nitrogen, NIS (1.6 g, 7.1 mmol, 2 eq) was added in small portions to an ice-cold solution of Comp. **24** (822 mg, 3.55 mmol) in DCM (20 mL), and the reaction mixture was stirred overnight while warming to room temperature. The reaction mixture was evaporated *in vacuo*, the residue was taken into EtOAc (100 mL). The organic layer was washed with 0.5 M aqueous sodium thiosulfate (2×30 mL), $NaHCO_3$ (30 mL), 0.3 M citric acid (2×30 mL), brine, and dried over $MgSO_4$. The organic extract was filtered and concentrated *in vacuo*. The crude product was purified by silica gel column chromatography using petroleum ether/EtOAc (9:1, v/v). Yield as a yellowish crystalline solid: 989 mg (78 %). The physical and spectroscopic properties of the isolated compound were in full agreement with the reported values. The reaction was repeated several times on a comparable scale (1.1 g of comp. **24)**, and omission of sodium thiosulfate during the workup resulted in a decreased isolated yield (450 mg, 27%).

^{1}H NMR (300 MHz, $CDCl_3$): δ 9.14 (s, 1H), 7.42 (br. d, *J* = 7.5 Hz, 1H), 7.28 – 7.17 (m, 2H), 4.48 (q, *J* = 7.1 Hz, 2H), 3.39 – 3.16 (m, *J* = 6.9 Hz, 1H), 1.63 (s, 1H), 1.48 (t, *J* = 7.1 Hz, 3H), 1.38 (d, *J* = 6.9 Hz, 6H).

^{13}C NMR (75 MHz, $CDCl_3$): δ 161.29, 134.73, 132.38, 131.66, 127.09, 122.35, 122.16, 121.31, 77.58, 77.16, 76.74, 66.75, 61.67, 28.96, 22.87, 14.51.

Comp. 26

Under nitrogen, comp. **25** (1.744 g, 4.88 mmol) was dissolved in acetonitrile (30 mL) and treated with the 4-boc-phenylboronic acid ester (2.148 g, 6.45 mmol, 1.3 eq), potassium carbonate (3.944 g, 28.54 mmol, 5.8 eq), and water (1.6 mL). The reaction mixture was purged with argon for 10 min, and Pd(dppf)Cl_2 (212 mg, 0.29 mmol, 0.06 eq) was added in one portion. Under nitrogen, the reaction mixture was brought to 80 °C (bath temperature) and heated at this temperature for 1.5 h. Quantitative product formation was confirmed by TLC, showing a new spot with bright blue fluorescence in Pet/EtOAc (9:1, v/v) TLC solvent system. The reaction was cooled, filtered, and the acetonitrile layer was separated from the water phase, and then evaporated under reduced pressure. The residue was extracted with EtOAc (100 mL), washed with brine, dried over $MgSO_4$, and concentrated *in vacuo.* The crude product was carried forward to the next step without additional purification in the form of a brown tar. The respective LC-MS analysis confirmed the title product formation detected as a dimer for the major ion with concomitant in-source single *tert*-butyl cleavage. LC-MS (ESI): $[2M+H - CO_2C_4H_9]^+$ m/z calcd. for $C_{26}H_{32}N_2O_4$, 773.99; found, 773.25.

Comp. 27

To a stirred solution of comp. **26** (est. ~4.88 mmol) in ethanol (50 mL) was added KOH (1.22 g, 21.7 mmol, 4.5 eq). The reaction mixture was heated at reflux under nitrogen for 1 h, during which the progress was monitored by TLC by the disappearance of the starting material and appearance of a new spot at the baseline. The reaction solution was then cooled to ambient temperature, and the solvent was removed under reduced pressure. The residue was dissolved in deionized water (50 mL), and an aqueous solution of ammonium chloride (1.164 g, 21.76 mmol, 4.5 eq) was added. After brief stirring, the mixture was concentrated *in vacuo*. The aqueous phase was further acidified with citric acid (8.36 g, 43.51 mmol, 8.9 eq), saturated with NaCl, and extracted with EtOAc (2 × 100 mL). The combined organic extracts were dried over anhydrous $MgSO_4$, filtered, and concentrated *in vacuo*. The crude material was purified by flash chromatography (hexane/EtOAc, gradient 20–70% EtOAc) to afford the target product as a white solid

(1.92 g, 96%). The respective LC-MS analysis for the major ion indicated dimer formation with concomitant in-source single *tert*-butyl cleavage. LC-MS (ESI): $[2M+H - CO_2C_4H_9]^+$ m/z calcd. for $C_{24}H_{28}N_2O_4$, 717.3574; found, 717.42.

^{1}H NMR (300 MHz, DMSO-d_6): δ 11.44 (s, 1H), 7.48 – 7.36 (m, 3H), 7.32 – 7.21 (m, 3H), 7.16 (d, *J* = 7.0 Hz, 1H), 7.03 (t, *J* = 7.6 Hz, 1H), 4.20 (d, *J* = 6.1 Hz, 2H), 3.74 (dt, *J* = 13.6, 6.9 Hz, 1H), 1.42 (s, 9H), 1.28 (d, *J* = 6.8 Hz, 6H).

^{13}C jmod NMR (75 MHz, DMSO-d_6): δ 163.07 (C=O), 155.89 (C=O), 138.45 (C_q), 134.33 (C_q), 133.18 (C_q), 132.53 (C_q), 130.31 (CH), 127.27 (C_q), 126.28 (CH), 124.09 (C_q), 122.27 (C_q), 120.65 (CH), 120.18 (CH), 117.84 (CH), 77.77 (C_q), 43.22 (CH_2), 28.29 (CH_3), 26.91 (CH), 23.12 (CH_3).

Comp. 28

Under a nitrogen atmosphere, TFA (10 mL, 130.68 mmol, 28 eq) was added dropwise *via* syringe to an ice-cooled solution of comp. **27** (1.92 g, 4.7 mmol) in dichloromethane (20 mL). The reaction mixture was stirred for 1 h, after which the solvent was removed under reduced pressure. The residue was repeatedly co-evaporated with ethanol (3 × 25 mL) to ensure complete removal of residual TFA. Separately, concentrated HCl (36%, 1.2 mL, 11.8 mmol, 2.5 eq) was added dropwise to a mixture of ethanol (50 mL) and deionized water (2 mL) to generate ethanolic HCl. This solution was then added to the residue obtained from the TFA treatment, and the mixture was stirred for 30 min. The resulting solution was concentrated *in vacuo* and purified by reverse-phase chromatography using water/acetonitrile (0.1% TFA) as eluents. Relevant fractions, as identified by LC-MS, were combined, diluted with 10% acetic acid, and lyophilized to afford the product as a white solid (976 mg, 60%). LC-MS (ESI): $[2M+H]^+$ m/z calcd. for $C_{19}H_{20}N_2O_2$, 617.31; found, 617.17.

^{1}H NMR (300 MHz, DMSO-d_6): δ 10.92 (s, 1H), 7.61 (d, *J* = 7.7 Hz, 2H), 7.52 (d, *J* = 7.5 Hz, 2H), 7.29 (d, *J* = 7.6 Hz, 1H), 7.03 (d, *J* = 6.9 Hz, 1H), 6.96 (t, *J* = 7.5 Hz, 1H), 4.03 (s, 2H), 3.80 – 3.61 (m, 1H), 1.27 (d, *J* = 6.7 Hz, 6H).

^{13}C jmod NMR (75 MHz, DMSO-d_6): δ 165.97 (C=O), 135.75 (C_q), 132.87 (C_q), 132.46 (C_q), 132.09 (C_q), 132.03 (C_q), 130.46 (CH), 127.93 (CH), 127.47(C_q), 119.82 (CH), 118.16 (CH), 116.88 (CH), 116.28 (C_q), 42.27 (CH_2), 27.17 (CH_3), 23.14 (CH_3).

Comp. 9

Under a nitrogen atmosphere, comp. **22** (1.05 g, 2.55 mmol, 1.4 eq) was suspended in 25 mL of anhydrous chloroform, and the solution was cooled down with an ice-water bath. DIPEA (4.12 g, 31.9 mmol, 17 eq) was added dropwise to the stirred solution, followed by DSC (606 mg, 2.37 mmol, 1.3 eq) in one portion. The reaction mixture was allowed to react for 1 hr at 0°C. After that, indole **28** (638 mg, 1.85 mmol) was then added in one portion, followed by the dropwise addition of KEHA (350 mg, 1.92 mmol, 1 eq) in anhydrous DMSO (2 mL) and DBU (317 mg, 2.08 mmol, 1.1 eq). The reaction mixture was stirred for 1 hr while warming to room temperature, and concentrated *in vacuo*. The obtained concentrated DMSO solution was further stirred for 0.5 hr, dissolved in an ice-cold mixture of 1M HCl (10 mL) and acetonitrile (4:1, v/v). The resultant solution was filtered, and the title product was purified by reverse-phase HPLC (C_{18}) using H_2O/MeCN (0.1% TFA) as eluents, affording the target compound as a caramel-like substance after lyophilization. Yield: 1.133 g (91%). LC-MS (ESI): $[M+H]^+$ *m/z* calcd. for $C_{37}H_{47}N_5O_7$, 674.35, found 674.58.

^{1}H NMR (300 MHz, DMSO-d_6): δ 11.48 (s, 1H), 8.77 – 8.68 (m, 2H), 8.28 – 8.17 (m, 2H), 7.83 (dd, *J* = 7.9, 5.5 Hz, 1H), 7.41 (d, *J* = 8.1 Hz, 2H), 7.30 (d, *J* = 8.2 Hz, 2H), 7.25 (d, *J* = 7.9 Hz, 1H), 7.17 (d, *J* = 6.9 Hz, 1H), 7.07 – 7.00 (m, 1H), 6.41 (br. s, 1H), 5.95 (br. s, 2H), 4.27 (s, 2H), 3.82 – 3.69 (m, 1H), 3.66 (s, 2H), 3.56 – 3.44 (m, 8H), 3.41 (q, *J* = 6.4 Hz, 4H), 3.17 – 3.03 (m, 4H), 1.71 – 1.56 (m, 4H), 1.28 (d, *J* = 6.8 Hz, 6H).

^{13}C jmod NMR (75 MHz, DMSO-d_6): δ 168.40 (C=O), 163.01 (C=O), 158.19 (C=O), 144.29 (CH), 144.04 (CH), 142.20 (CH), 139.28 (C_q), 135.15 (C_q), 134.42 (C_q), 133.24 (C_q), 132.40 (C_q), 130.31 (CH), 127.29 (C_q), 126.44 (CH), 125.68 (CH), 123.85 (C_q), 122.50 (C_q), 120.71 (CH), 120.26 (CH), 117.87 (CH), 69.78 (CH_2), 69.57 (CH_2), 69.53 (CH_2), 68.21 (CH_2), 67.96 (CH_2), 42.82 (CH_2), 38.46 (CH_2), 36.73 (CH_2), 36.15 (CH_2), 30.25 (CH_2), 29.21 (CH_2), 26.92 (CH), 23.12 (CH_3).

Synthesis of MBL-siderophore conjugates 10 and 11

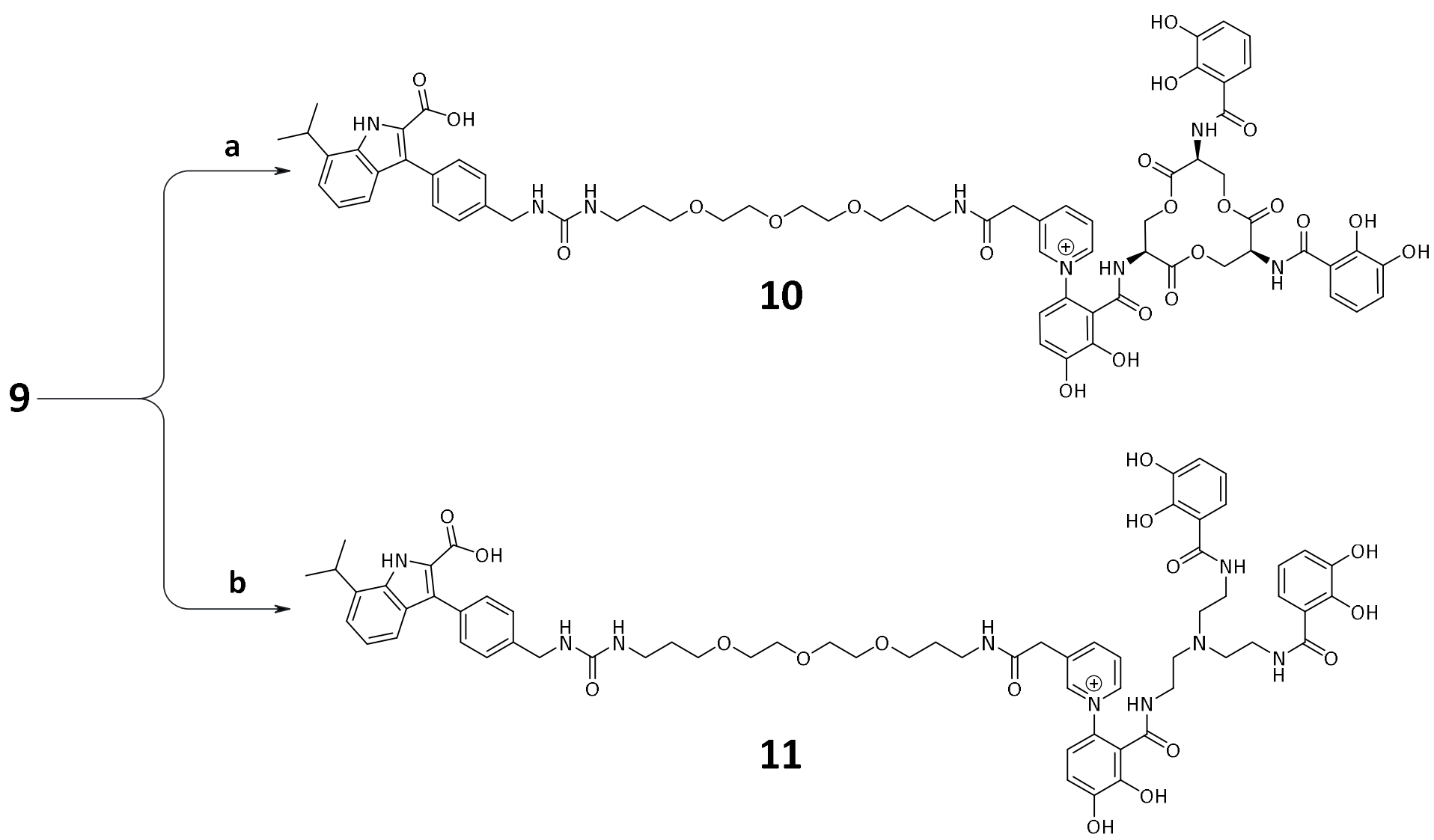

Scheme 6. Synthesis of MBL conjugates, reagents and conditions: a) 1 eq comp. 9, 1 eq Ent (1), 2.1 eq STF, 1.1 eq I_2, DMSO, rt, 1h, 21%; b) 1.3 eq comp. 9, 1 eq TRENCAM (2), 2.7 eq STF, 1.2 eq I_2, DMSO, rt, 1h, 28%.

Synthetic procedures for MBL-siderophore conjugates 10 and 11

Comp. 11

Comp. **1** (28 mg, 0.042 mmol, 1 eq.) and comp. **9** (28 mg, 0.042 mmol, 1 eq.) were suspended in DMSO (0.6 mL) in a 2 mL Eppendorf plastic test tube. A suspension of STF (17 mg, 0.087 mmol, 2.1 eq.) in DMSO (0.3 mL) was added in one portion to the mixture, followed by gentle shaking to ensure proper mixing. The resulting solution was kept at room temperature for 5 min with additional shaking (2-3 times). With vigorous stirring applied, a solution of iodine (12 mg, 0.047 mmol, 1.1 eq.) in DMSO (0.3 mL) was added dropwise, and the reaction mixture was left at room temperature for 1h with stirring. The reaction mixture was then centrifuged to remove the precipitate. The supernatant was diluted to a total volume of 5 mL using 1M HCl and acetonitrile (4:1, v/v) and purified by reverse-phase HPLC (C_{18}) using H_2O/MeCN with 0.1% TFA as eluents to afford the target compound as a white solid. Yield: 12 mg (21%). The molecular ion was observed in agreement with the calculated exact mass (HRMS (ESI): *m/z* [M]$^+$ calcd for $C_{67}H_{73}N_8O_{22}^+$ 1341.4839, found 1341.4832).

Comp. 10

Using TRENCAM×HCl (160 mg, 0.27 mmol, 1 eq.), comp. **9** (258 mg, 0.36 mmol, 1.3 eq.), STF (141 mg, 0.73 mmol, 2.7 eq.), and iodine (80 mg, 0.32 mmol, 1.2 eq.) in DMSO at room temperature, the title compound was prepared by following the same procedure described for **11**, off-white solid. Yield: 98 mg (28%). HRMS (ESI): *m/z* [M]$^+$ calcd for $C_{64}H_{76}N_9O_{16}^+$ 1226.5405, found 1226.5405.

References

1. Bildziukevich, U. *et al.* Amides derived from heteroaromatic amines and selected steryl hemiesters. *Steroids* **78**, 1347–1352 (2013).

2. Caradec, T. *et al.* A Novel Natural Siderophore Antibiotic Conjugate Reveals a Chemical Approach to Macromolecule Coupling. *ACS Cent. Sci.* **9**, 2138–2149 (2023).

3. B. Aziz, S. *et al.* Fabrication of Interconnected Plasmonic Spherical Silver Nanoparticles with Enhanced Localized Surface Plasmon Resonance (LSPR) Peaks Using Quince Leaf Extract Solution. *Nanomaterials* **9**, (2019).

4. von der Heiden, D., Rissanen, K. & Erdélyi, M. Asymmetric [N–I–N]+ halonium complexes in solution? *Chemical Communications* **56**, 14431–14434 (2020).

5. Marinez, E. R., Salmassian, E. K., Lau, T. T. & Gutierrez, C. G. Enterobactin and Enantioenterobactin. *J. Org. Chem.* **61**, 3548–3550 (1996).

6. Meyer, M. *et al.* High-Yield Synthesis of the Enterobactin Trilactone and Evaluation of Derivative Siderophore Analogs1. *J. Am. Chem. Soc.* **119**, 10093–10103 (1997).

7. Johnstone, T. C. & Nolan, E. M. Determination of the Molecular Structures of Ferric Enterobactin and Ferric Enantioenterobactin Using Racemic Crystallography. *J. Am. Chem. Soc.* **139**, 15245–15250 (2017).

8. Lee, A. A. *et al.* Facile and Versatile Chemoenzymatic Synthesis of Enterobactin Analogues and Applications in Bacterial Detection. *Angew. Chem. Int. Ed.* **55**, 12338–12342 (2016).

9. Baramov, T. *et al.* Synthesis and Structural Characterization of Hexacoordinate Silicon, Germanium, and Titanium Complexes of the E. coli Siderophore Enterobactin. *Chemistry – A European Journal* **19**, 10536–10542 (2013).

10. Pintér, Á. & Haberhauer, G. Synthesis and Investigation of a Chiral Enterobactin Analogue Based on a Macrocyclic Peptide Scaffold. *Chemistry – A European Journal* **14**, 11061–11068 (2008).

11. Demaerel, J., Veryser, C. & Borggraeve, W. Ex situ gas generation for lab scale organic synthesis. *React. Chem. Eng.* **5**, (2020).

12. Dertz, E. A., Xu, J. & Raymond, K. N. Tren-Based Analogues of Bacillibactin: Structure and Stability1. *Inorg. Chem.* **45**, 5465–5478 (2006).

13. Motz, R. N. *et al.* Conjugation to Native and Nonnative Triscatecholate Siderophores Enhances Delivery and Antibacterial Activity of a β-Lactam to Gram-Negative Bacterial Pathogens. *J. Am. Chem. Soc.* **146**, 7708–7722 (2024).

14. Soulere, L., Viode, C., Perie, J. & Hoffmann, P. Selective inhibition of Fe- versus Cu/Zn-superoxide dismutases by 2,3-dihydroxybenzoic acid derivatives. *Chem. Pharm. Bull. (Tokyo).* **50**, 578–582 (2002).

15. Zheng, T., Bullock, J. L. & Nolan, E. M. Siderophore-Mediated Cargo Delivery to the Cytoplasm of Escherichia coli and Pseudomonas aeruginosa: Syntheses of

Monofunctionalized Enterobactin Scaffolds and Evaluation of Enterobactin–Cargo Conjugate Uptake. *J. Am. Chem. Soc.* **134**, 18388–18400 (2012).

16. Hsu, S. L.-C., Chen, Y.-T., Chen, M.-L. & Chen, I.-G. Low Sintering Temperature Nano-Silver Pastes with High Bonding Strength by Adding Silver 2-Ethylhexanoate. *Materials* **14**, (2021).

17. Gao, M.-L. *et al.* Synthesis and Evaluation of Carbapenem/Metallo-β-Lactamase Inhibitor Conjugates. *ChemMedChem* **19**, e202400302 (2024).

Low resolution UHPLC-MS and HRMS data

Comp *MS1*

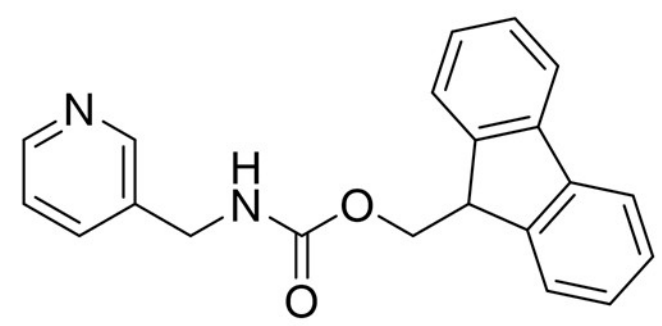

Chemical Formula: $C_{21}H_{18}N_2O_2$
Exact Mass: 330.1368

LR HPLC-MS chromatogram of comp *MS1*, UV 254 nm, C_{18}, 0–100% H_2O/MeCN+0.1% FA (linear gradient, 0-12 min), +ESI (pur. %)

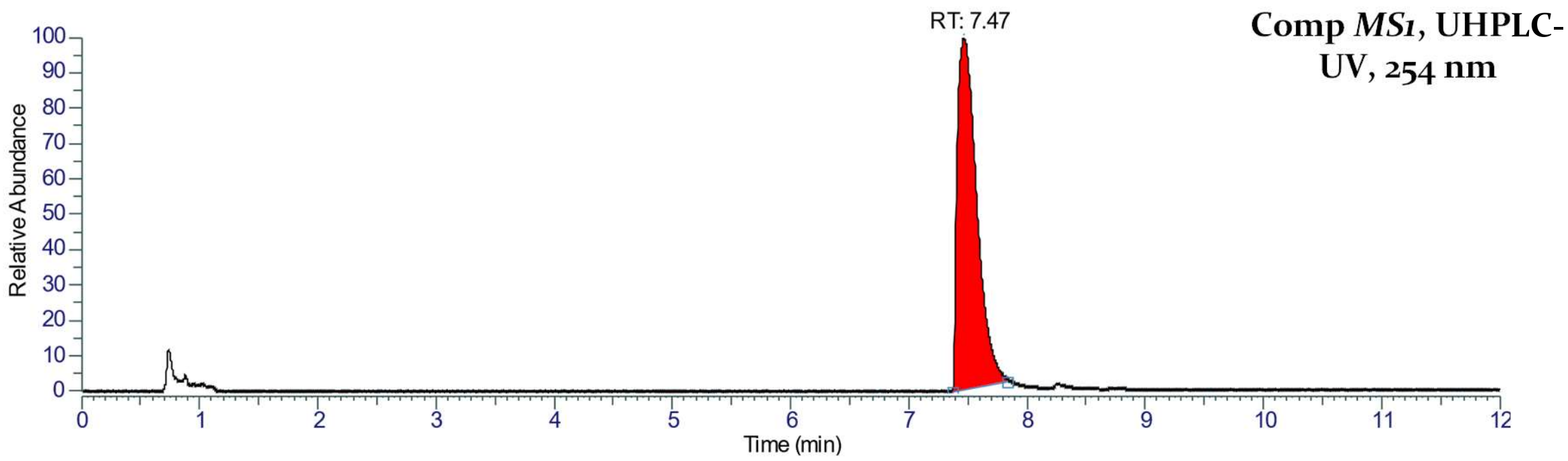

LR HPLC-MS BPC chromatogram of comp *MS1*, +ESI, m/z 200–2000

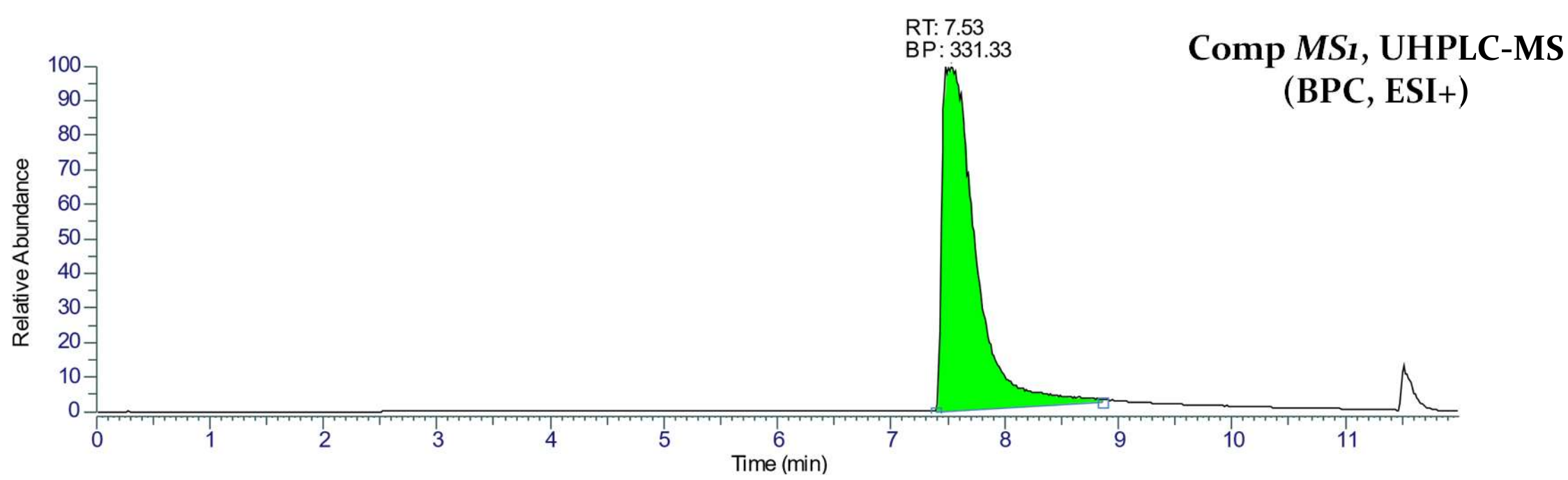

LR HPLC-MS BP spectrum of comp *MS1*, +ESI, m/z 200–2000

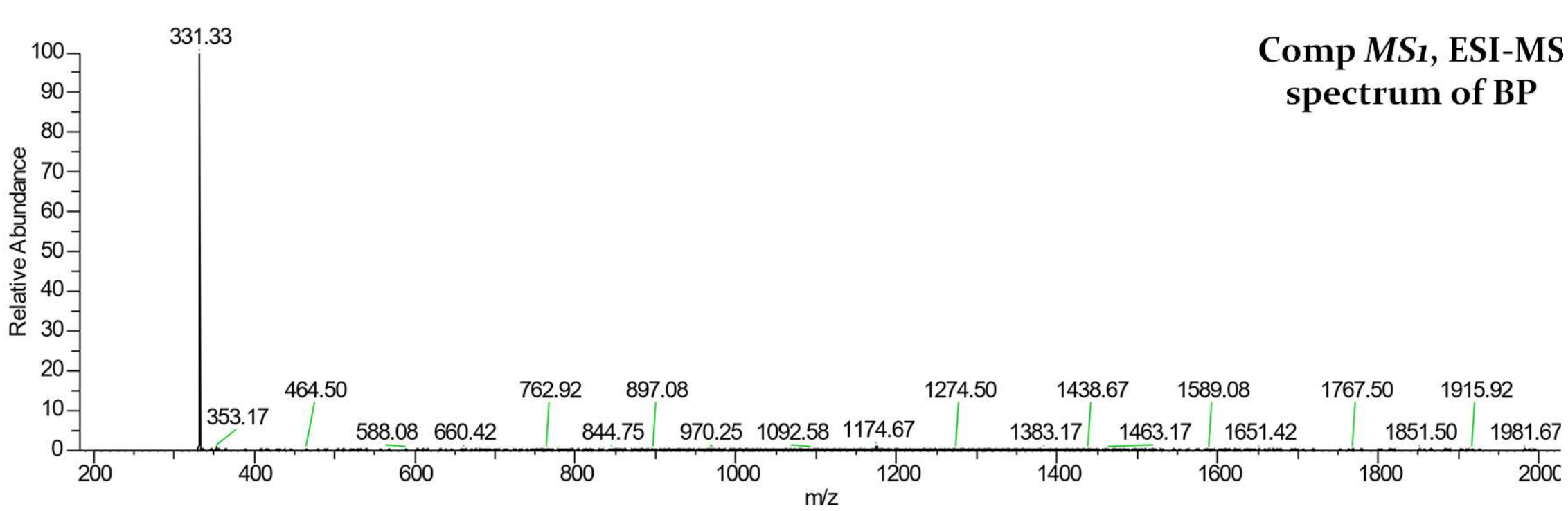

Comp *MS1* with 1 eq I_2 added

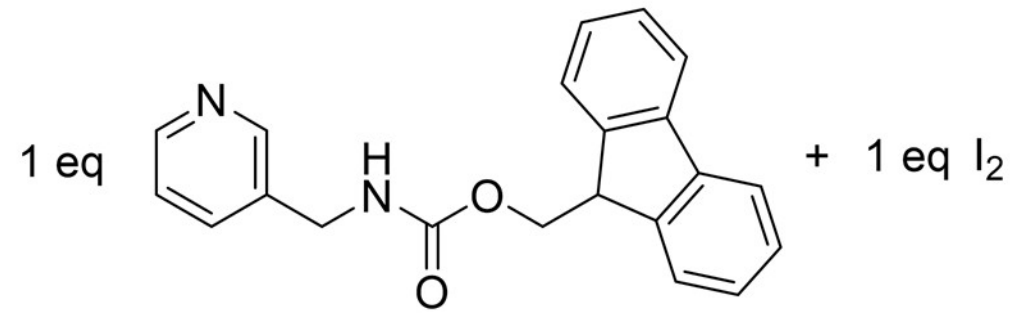

LR HPLC-MS chromatogram of comp , UV 254 nm, C_{18}, 0–100% H_2O/MeCN+0.1% FA (linear gradient, 0-12 min), ESI^+ (pur. %)

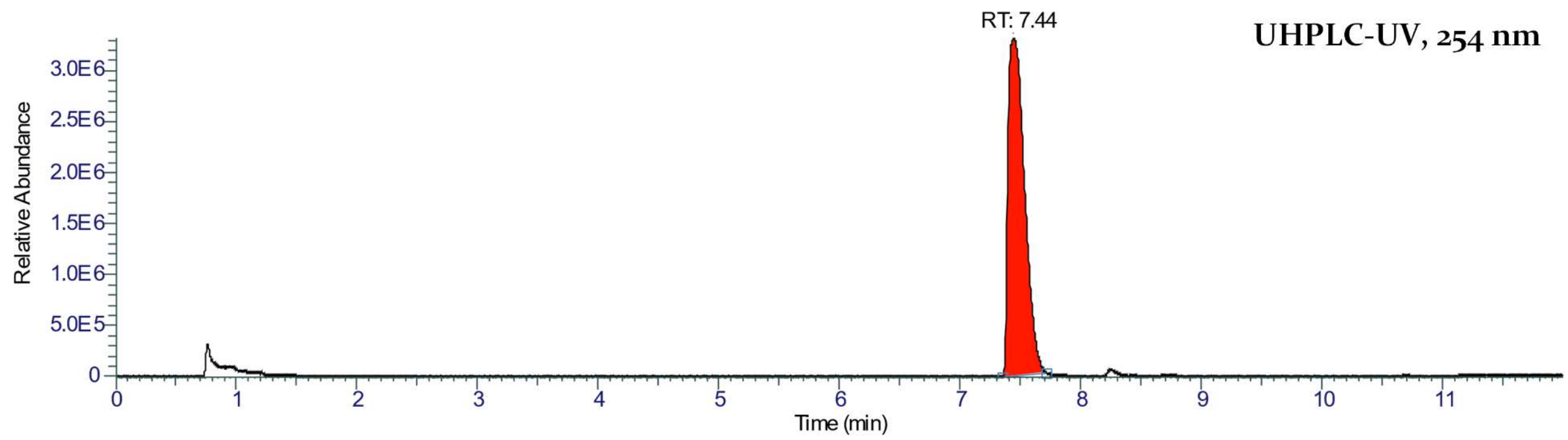

LR HPLC-MS BPC chromatogram, ESI^+, m/z 200–2000

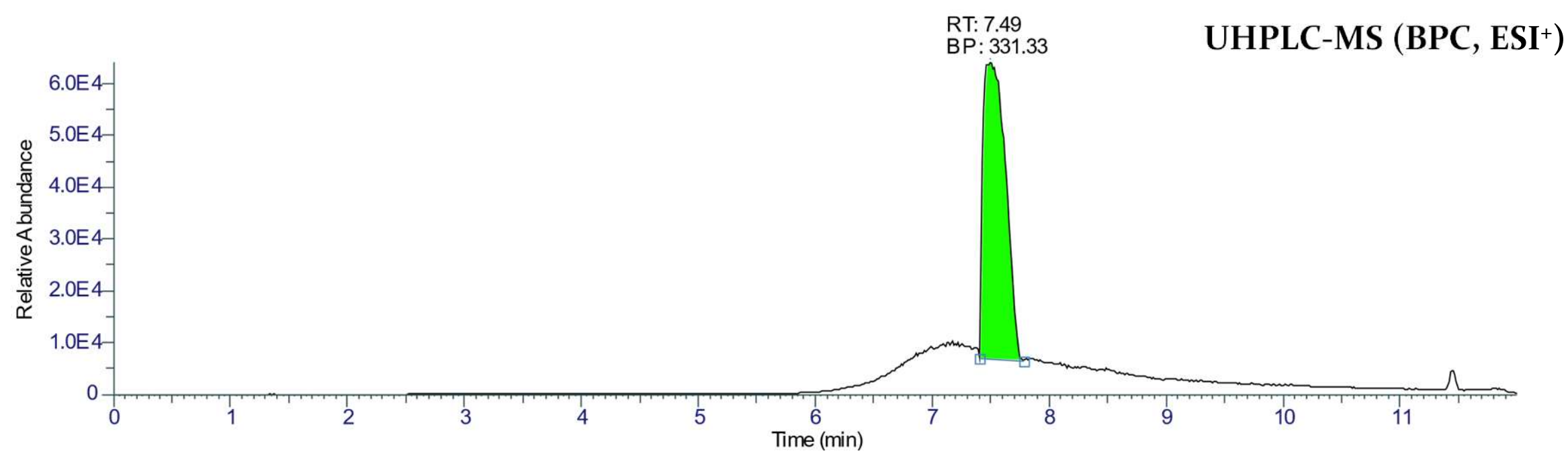

LR HPLC-MS BP spectrum of the base peak at t = 7.49 min, ESI^+, m/z 200–2000

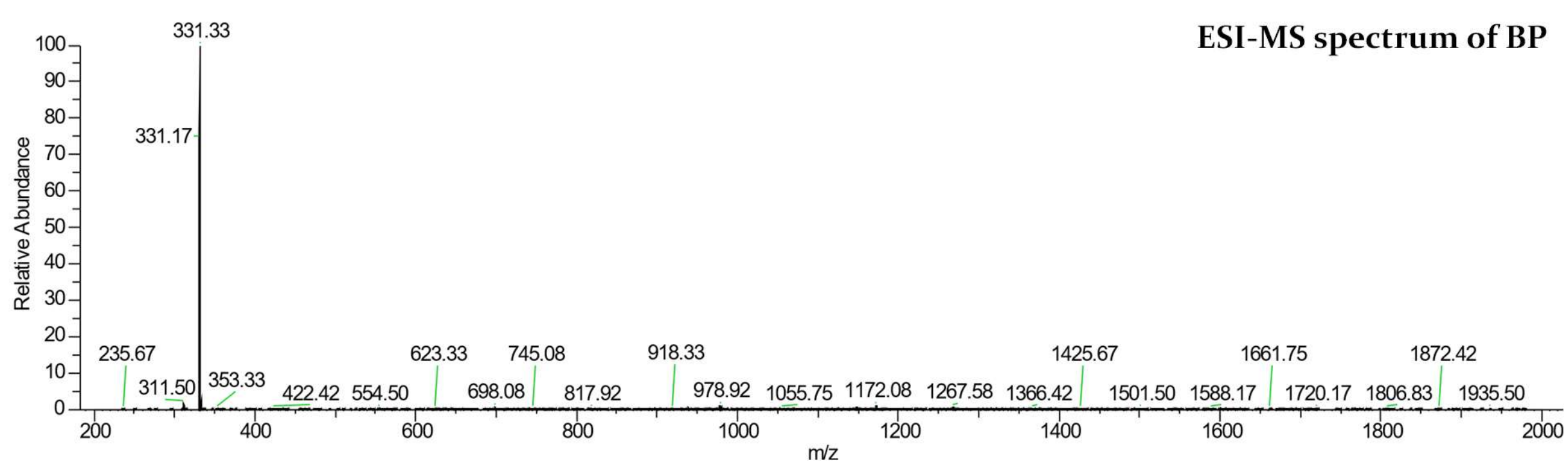

Comp *MS1* in the presence of 1 eq I_2 and 2 eq STF (reaction mixture)

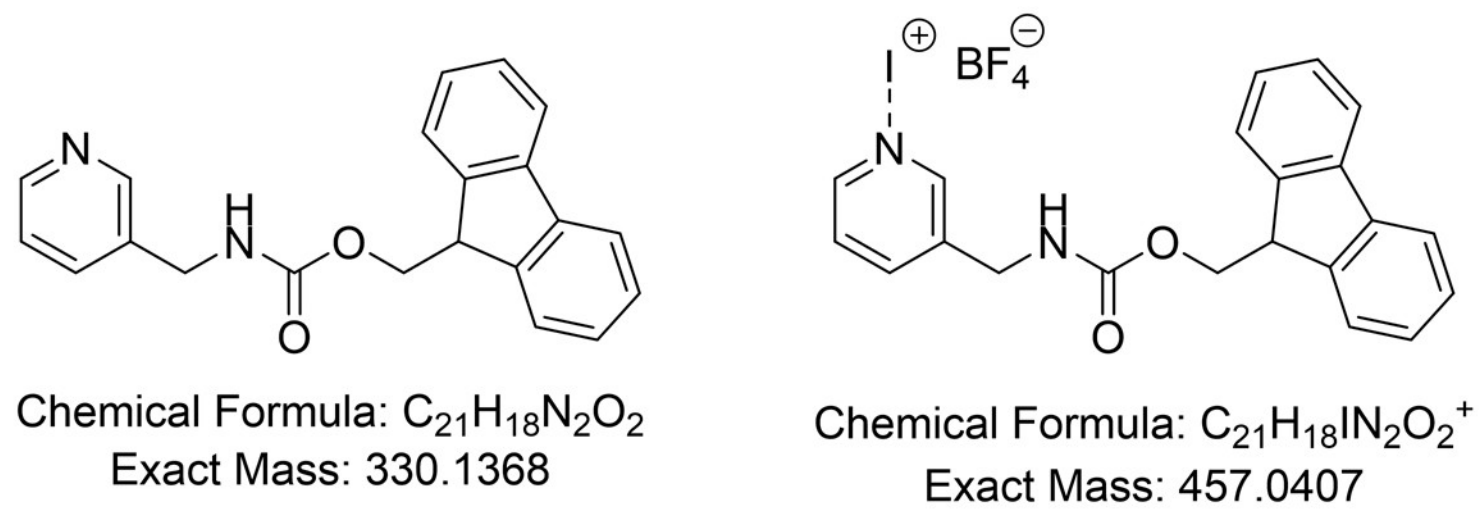

LR HPLC-MS chromatogram, UV 254 nm, C_{18}, 0–100% H_2O/MeCN+0.1% FA (linear gradient, 0-12 min), ESI^+ (13:1 ratio of MS1 to $[MS1\text{-}I]^+$ at 254 nm)

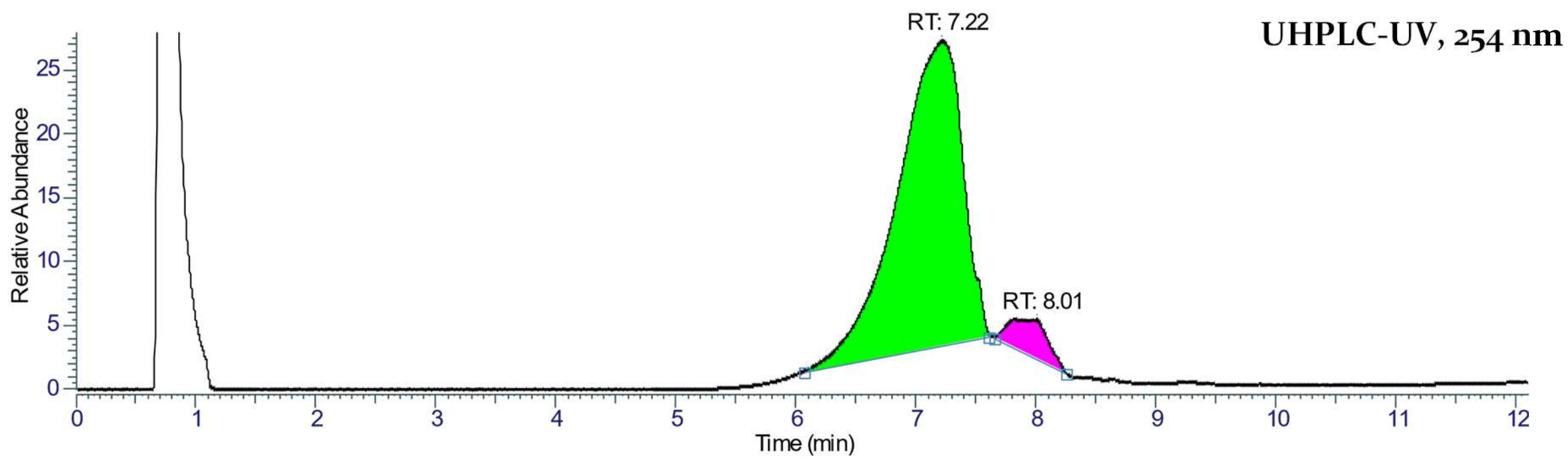

LR HPLC-MS BPC chromatogram, ESI^+, m/z 200–2000 (1.6:1 ratio of MS1 to $[MS1\text{-}I]^+$ at 254 nm)

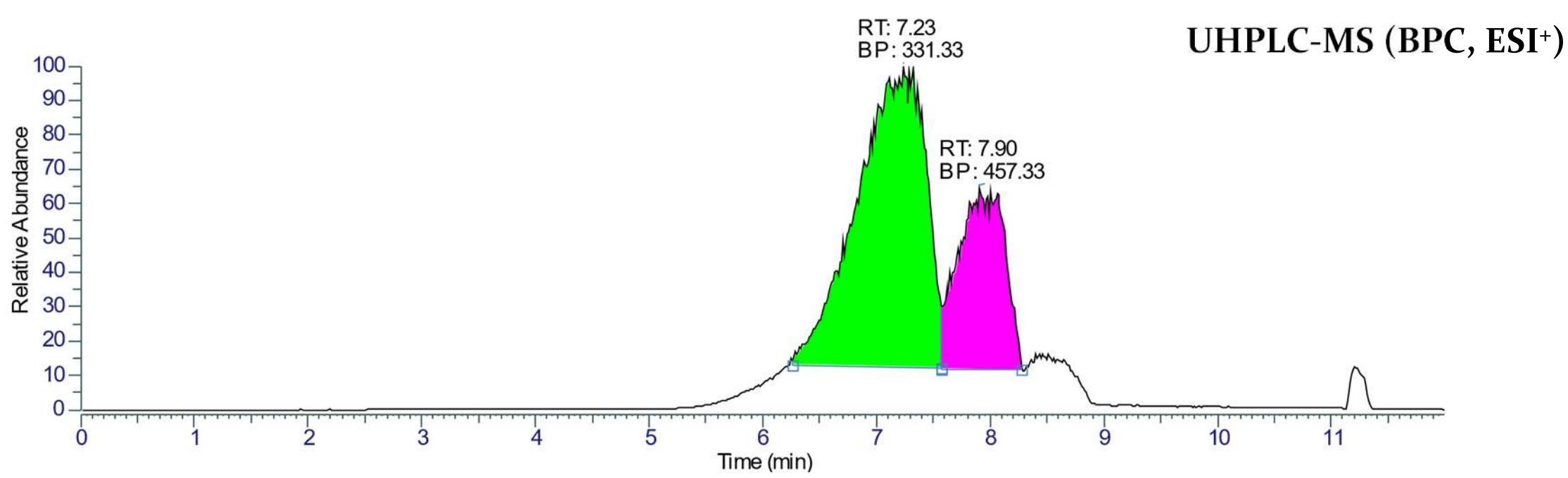

LR HPLC-MS BP spectrum, ESI^+, m/z 200–2000

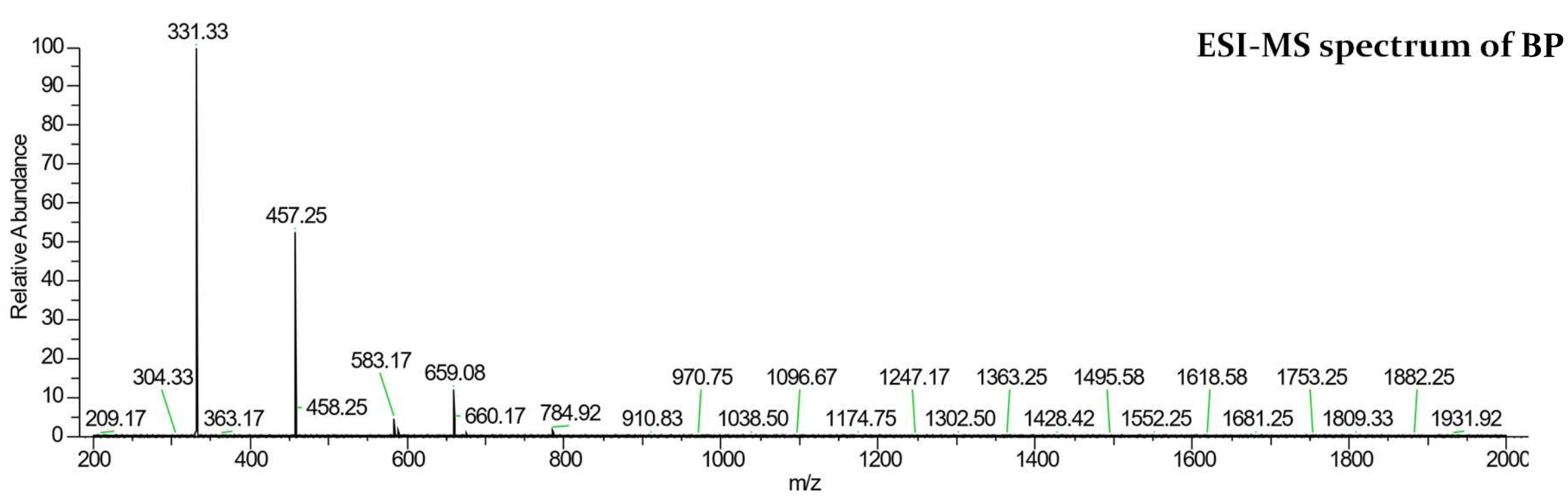

Bis(3-methylpyridine)I^+ BF_4^-

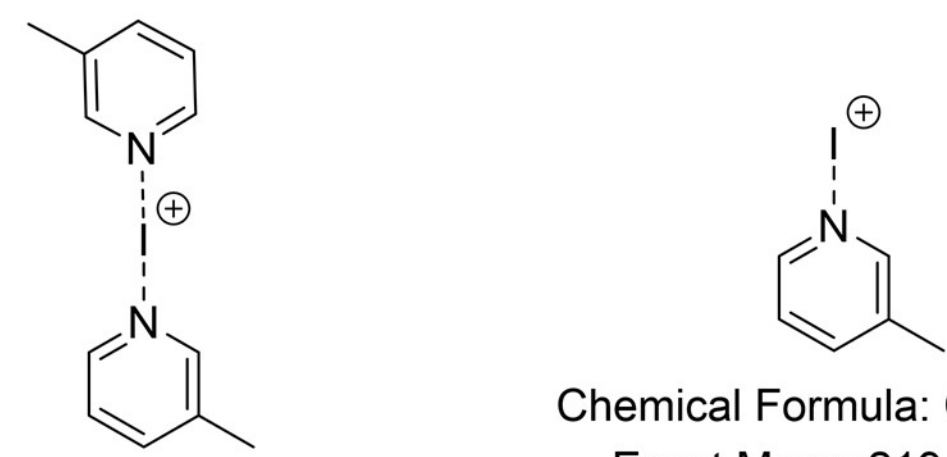

Chemical Formula: $C_{12}H_{14}IN_2^+$
Exact Mass: 313.0196

Chemical Formula: $C_6H_7IN^+$
Exact Mass: 219.9618

LR HPLC-MS BP spectrum of bis(3-methylpyridine)$I^+BF_4^-$, ESI+, m/z 200–2000

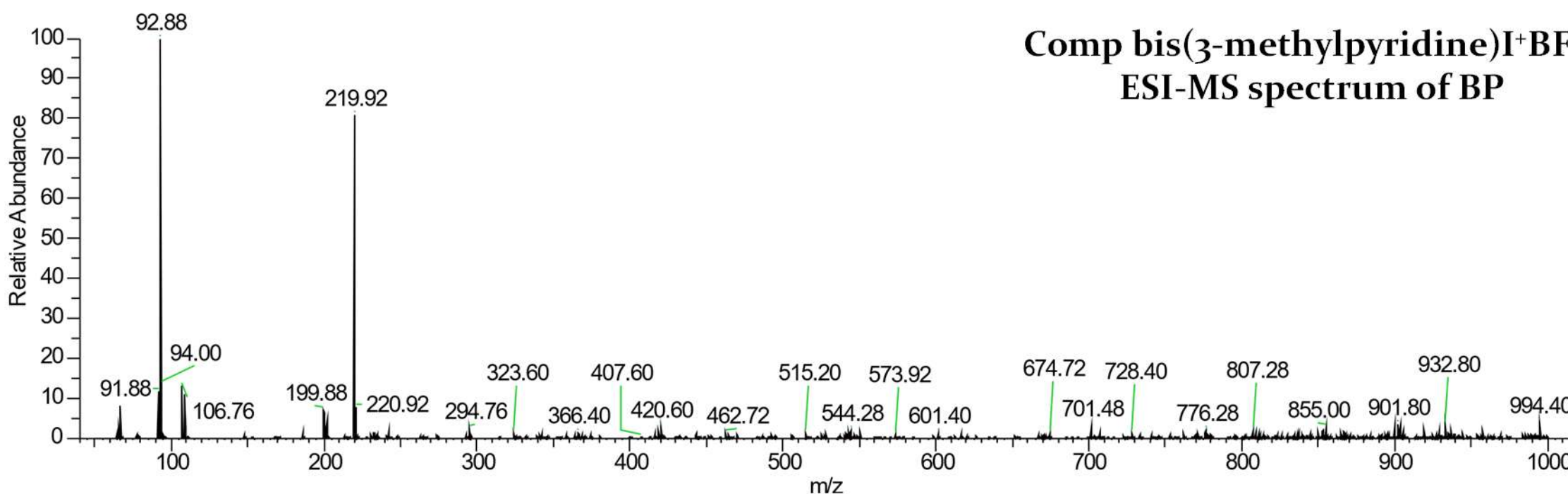

Analytical Results for the Model Reaction

Entry 1 (control experiment, a 1:1 eq mixture of *MS1* and *MS2*, no oxidant)

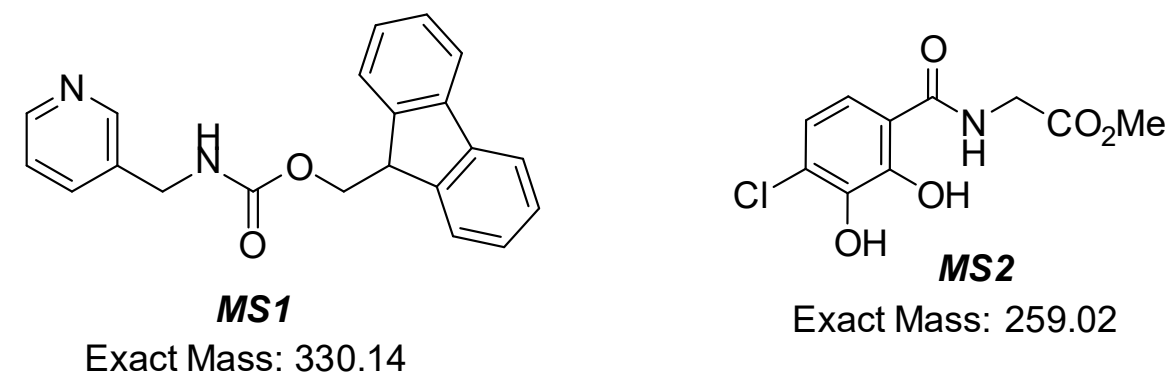

LR HPLC-MS chromatogram, UV 254 nm, C_{18}, 0–100% H_2O/MeCN+0.1% FA (linear gradient, 0-12 min), ESI$^+$

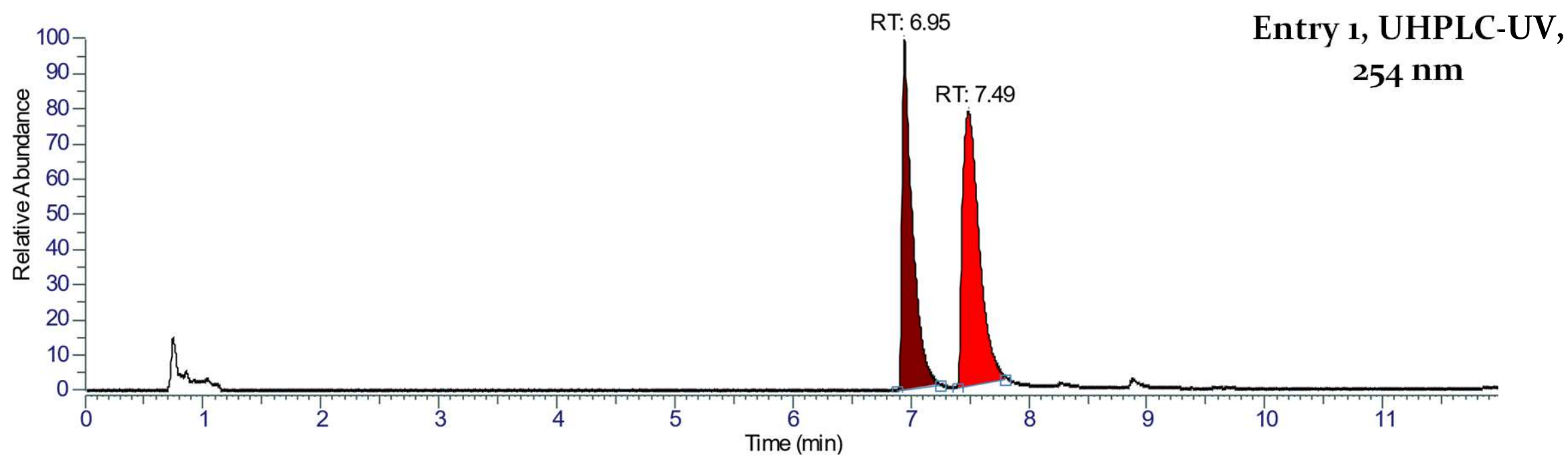

LR HPLC-MS BPC chromatogram, ESI$^+$, m/z 200–2000

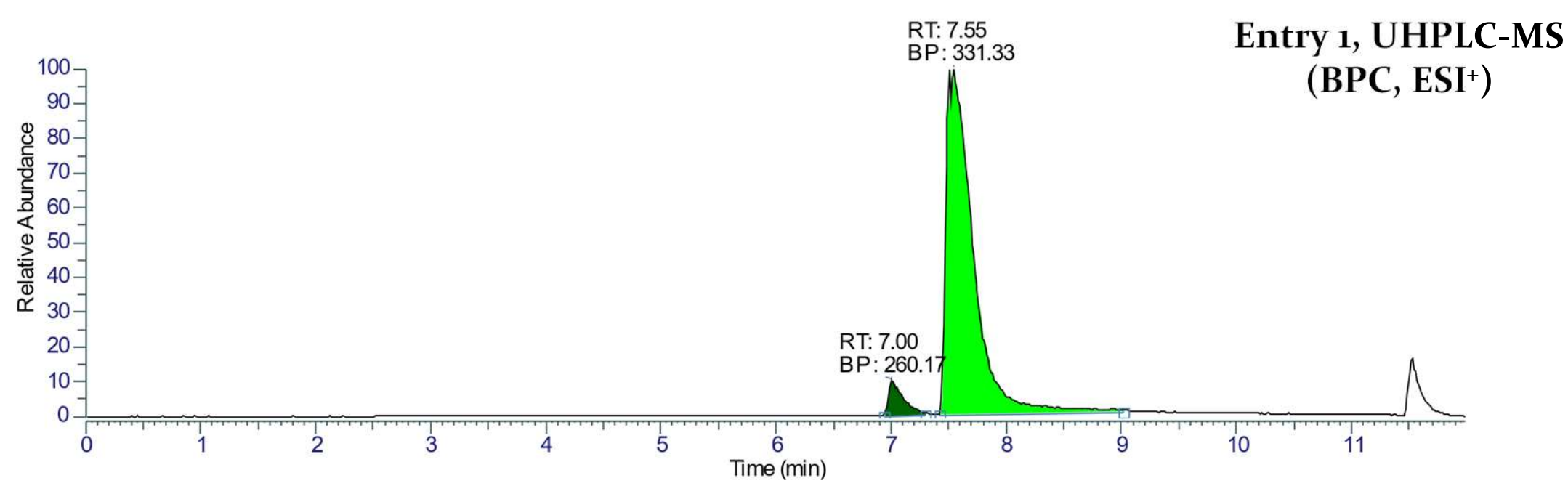

LR HPLC-MS BP spectrum, ESI$^+$, m/z 200–2000

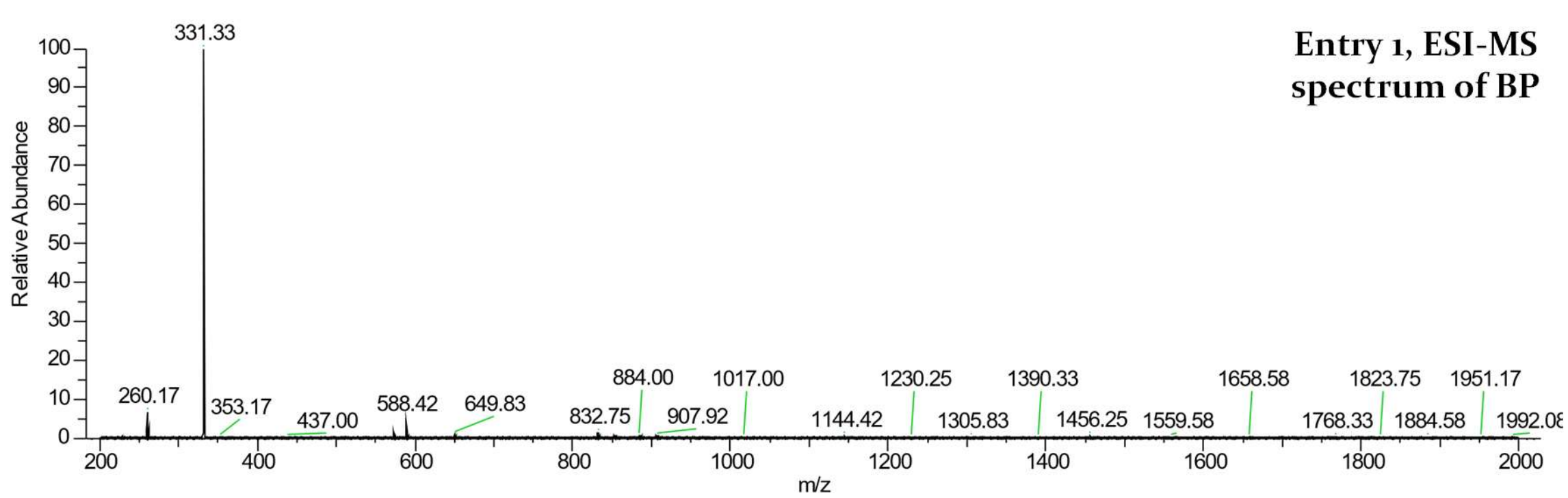

Entry 4 (1 eq I_2 added to 1:1 eq mixture of *MS1* and *MS2*)

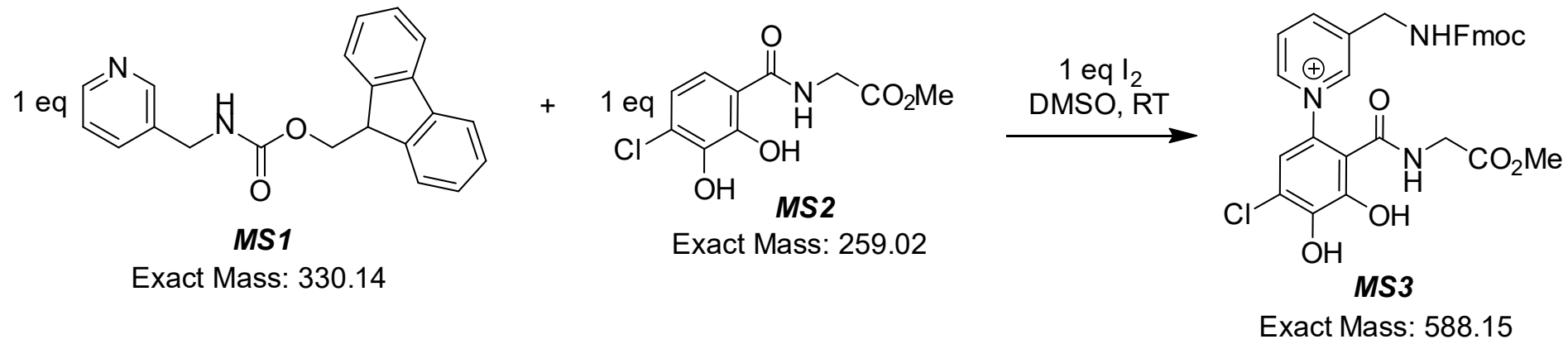

LR HPLC-MS chromatogram, UV 254 nm, C_{18}, 0–100% H_2O/MeCN+0.1% FA (linear gradient, 0-12 min), ESI⁺

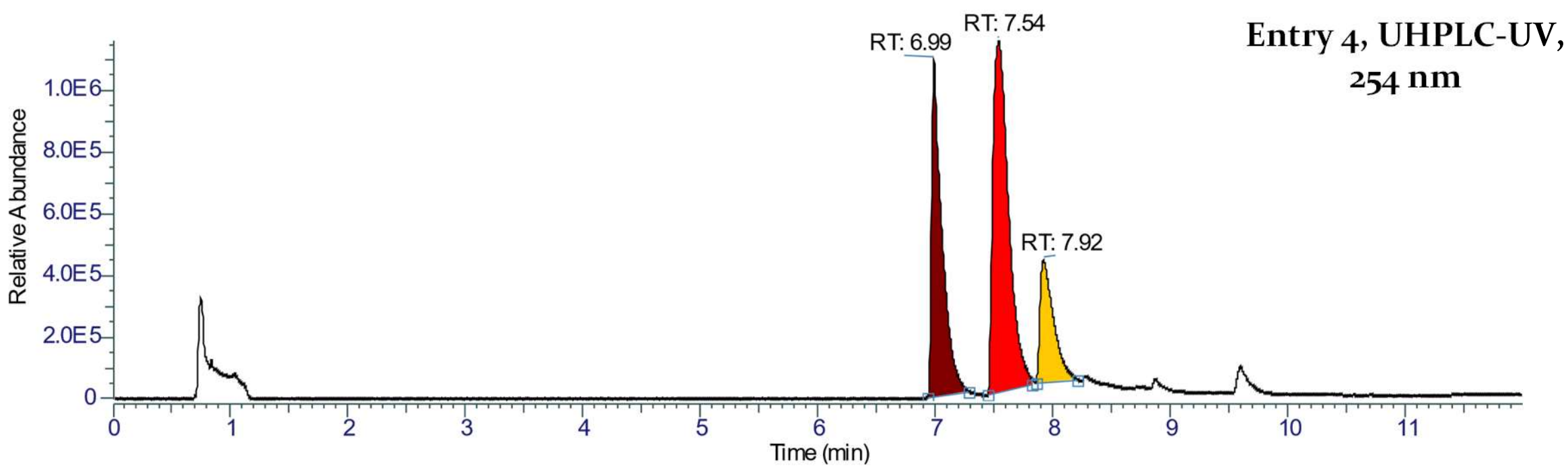

PEAK LIST											
Index	RT (Min)	Start RT	End RT	Peak Area	Peak Height	Baseline Width	SignalToNoise	TraceId	% Area	% Height	Integration Method
1	6.96	6.90	7.26	13584329.73	1897045.82	0.35	3337.33	Trace1	36.65	42.29	Automated - ICIS
2	7.54	7.45	7.81	15477573.20	1614232.64	0.36	2839.80	Trace1	41.76	35.99	Automated - ICIS
3	7.93	7.86	8.22	8003535.64	974535.93	0.35	1714.43	Trace1	21.59	21.72	Automated - ICIS

LR HPLC-MS BPC chromatogram of entry 4, ESI⁺, m/z 200–2000

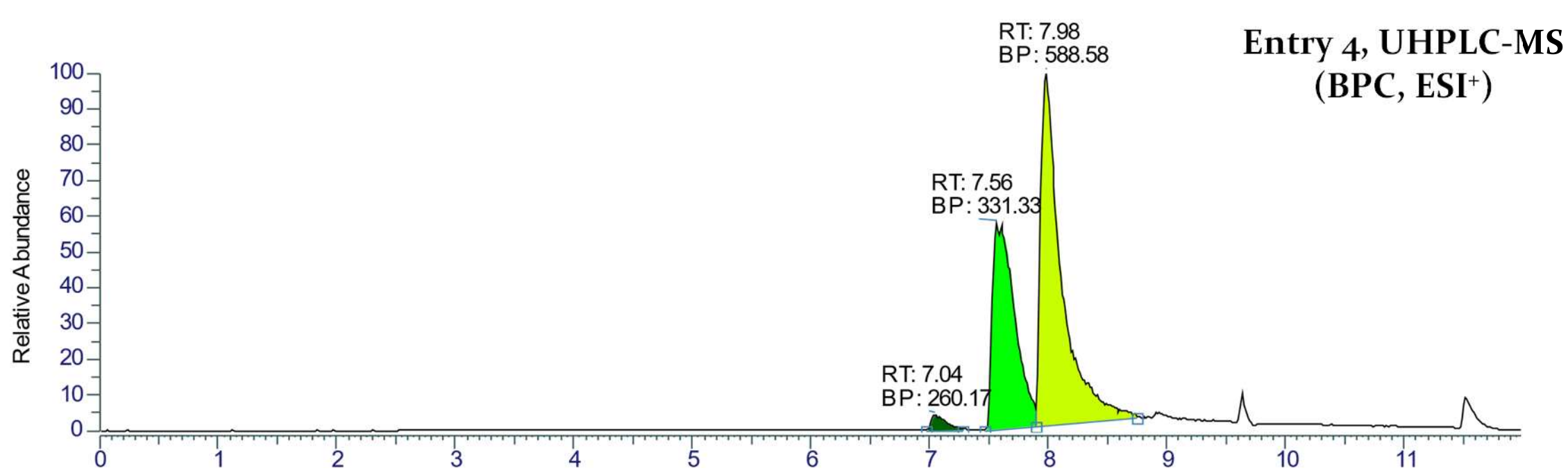

LR HPLC-MS BP spectrum, ESI⁺, m/z 200–2000 (BP at *t*=7.98 min)

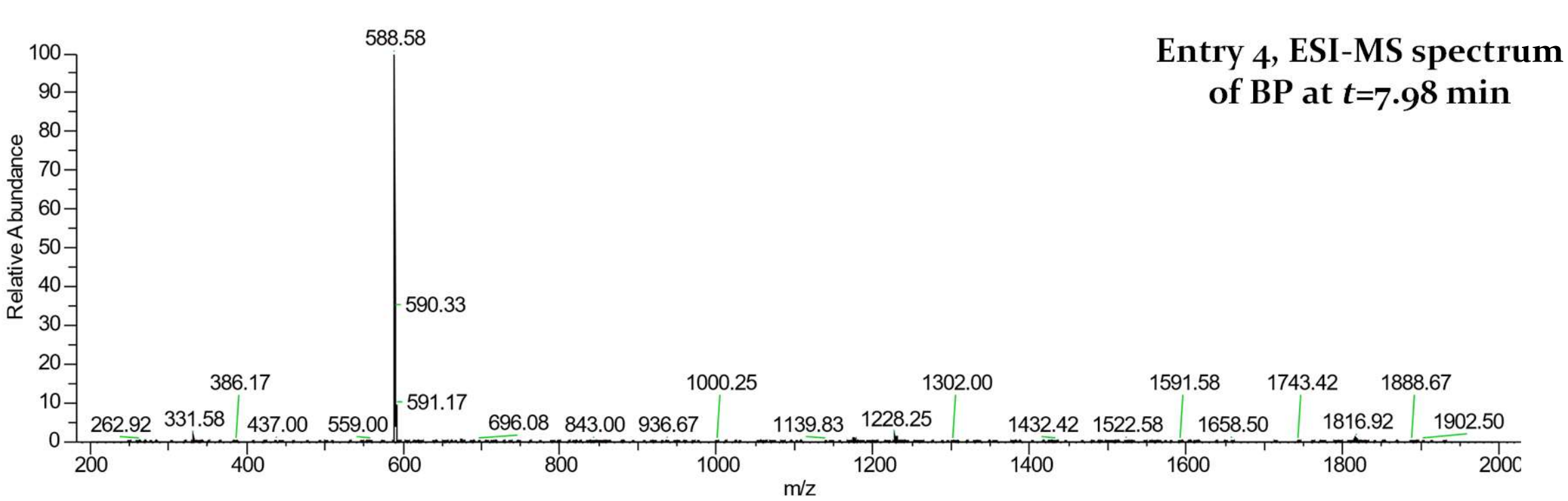

Entry 6 (1 eq I_2 was added to 1:1 eq mixture of *MS1* and *MS2,* followed by 1 eq STF)

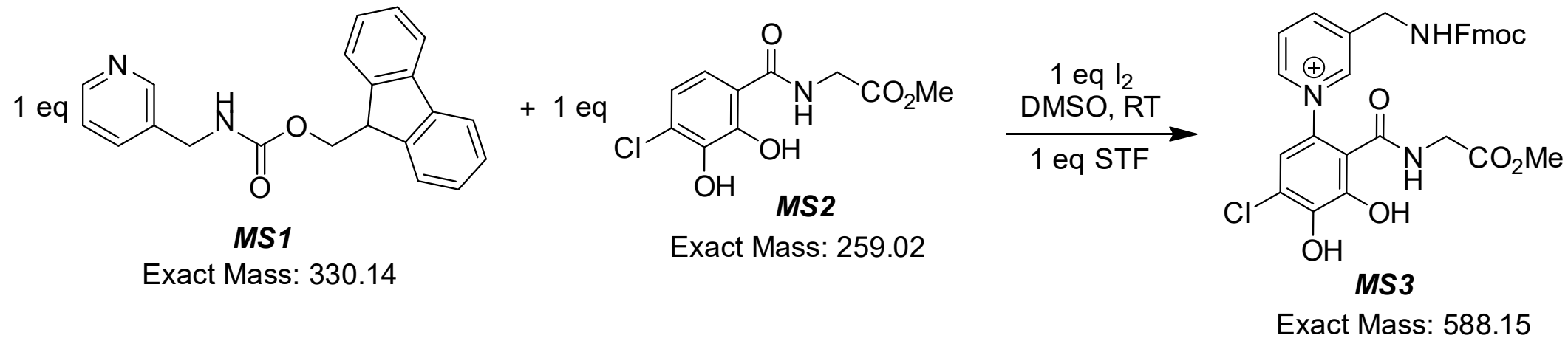

LR HPLC-MS chromatogram, UV 254 nm, C_{18}, 0–100% H_2O/MeCN+0.1% FA (linear gradient, 0-12 min), ESI^+

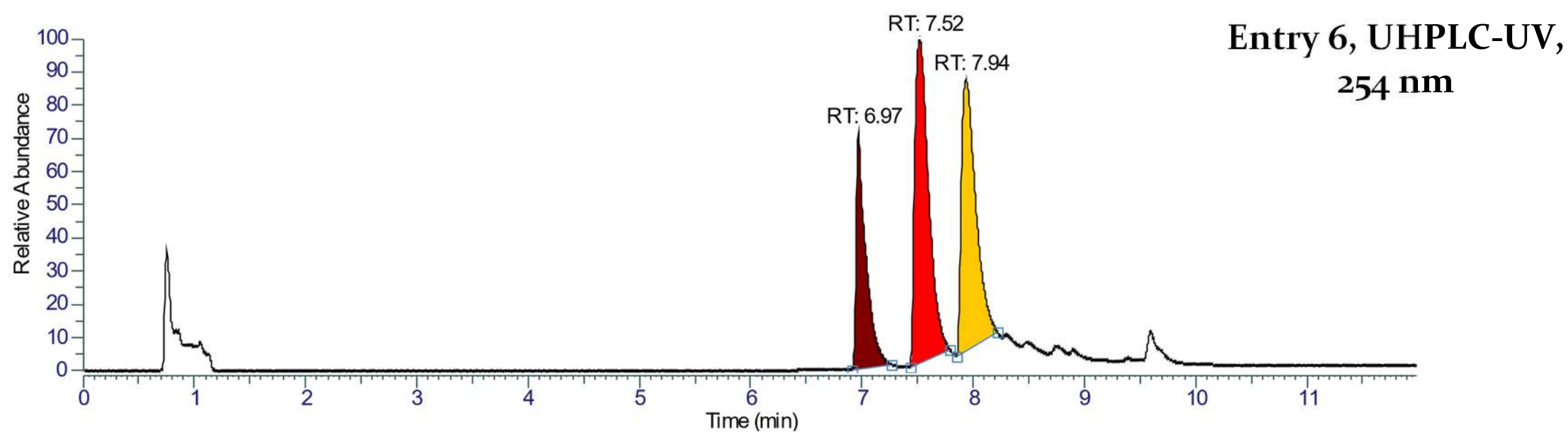

PEAK LIST											
Index	RT (Min)	Start RT	End RT	Peak Area	Peak Height	Baseline Width	SignalToNoise	TraceId	% Area	% Height	Integration Method
1	6.97	6.91	7.26	7782740.13	1241763.00	0.35	1917.46	Trace1	20.87	28.66	Automated - ICIS
2	7.52	7.43	7.79	13124295.12	1426492.19	0.36	2202.71	Trace1	35.19	32.93	Automated - ICIS
3	7.94	7.85	8.22	16392586.35	1664290.16	0.36	2569.91	Trace1	43.95	38.41	Automated - ICIS

LR HPLC-MS BPC chromatogram of entry 6, ESI^+, m/z 200–2000

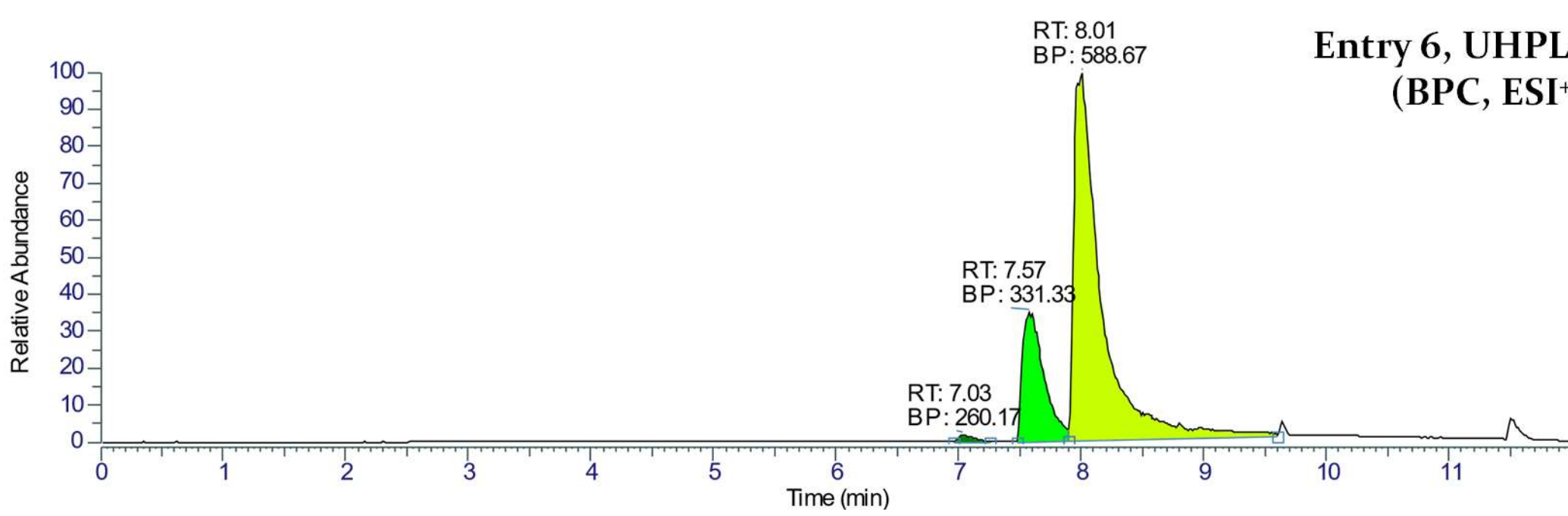

LR HPLC-MS BP spectrum, ESI^+, m/z 200–2000 (BP at *t*=8.01 min)

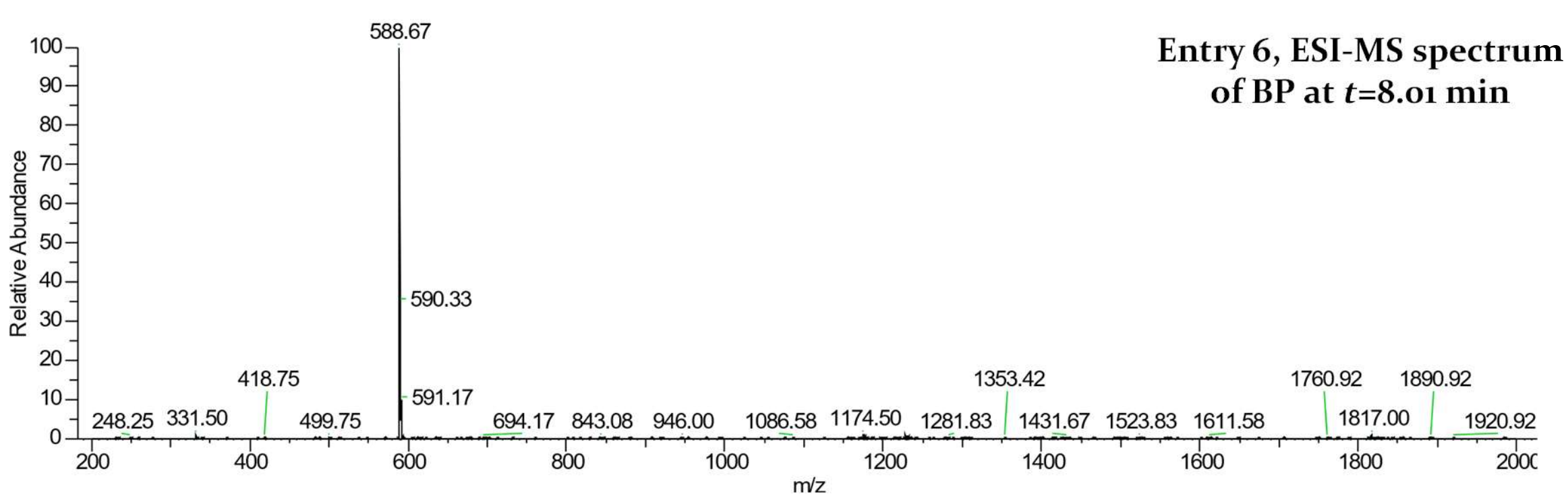

Entry 7 (1 eq STF was added to 1:1 eq mixture of *MS1* and *MS2,* followed by 1eq I_2)

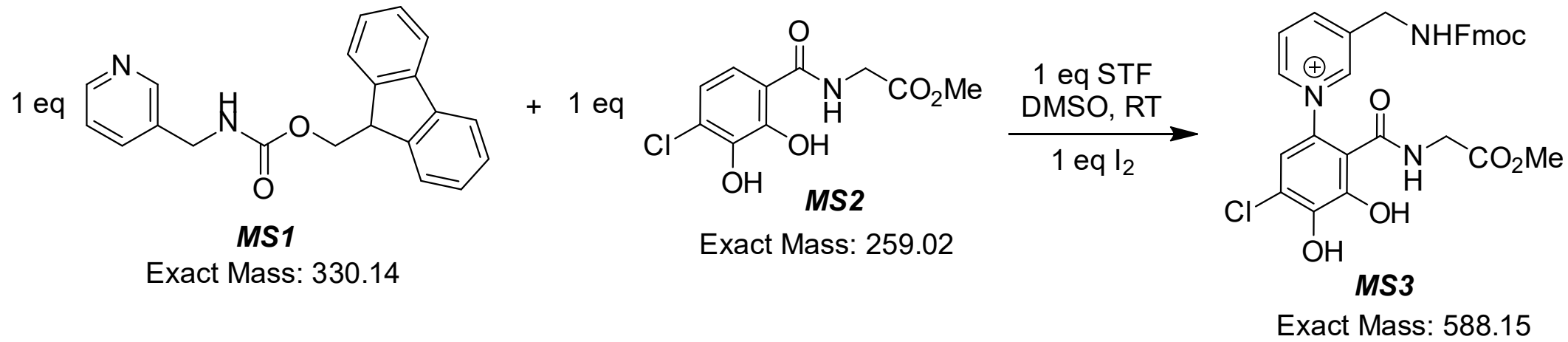

LR HPLC-MS chromatogram, UV 254 nm, C_{18}, 0–100% H_2O/MeCN+0.1% FA (linear gradient, 0-12 min), ESI+

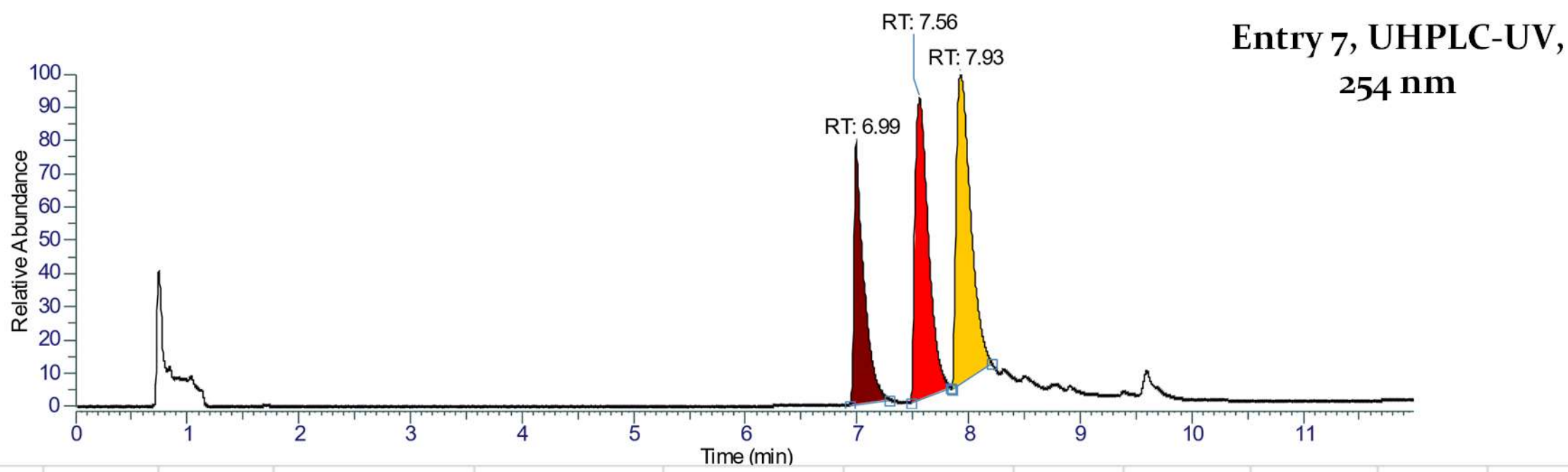

PEAK LIST											
Index	RT (Min)	Start RT	End RT	Peak Area	Peak Height	Baseline Width	SignalToNoise	TraceId	% Area	% Height	Integration Method
1	7.00	6.94	7.29	8037336.64	1272750.99	0.35	1793.06	Trace1	21.88	29.26	Automated - ICIS
2	7.56	7.48	7.84	11137057.85	1268042.52	0.35	1786.43	Trace1	30.32	29.15	Automated - ICIS
3	7.93	7.85	8.21	17556240.50	1809264.65	0.36	2548.90	Trace1	47.80	41.59	Automated - ICIS

LR HPLC-MS BPC chromatogram of entry 7, ESI+, m/z 200–2000

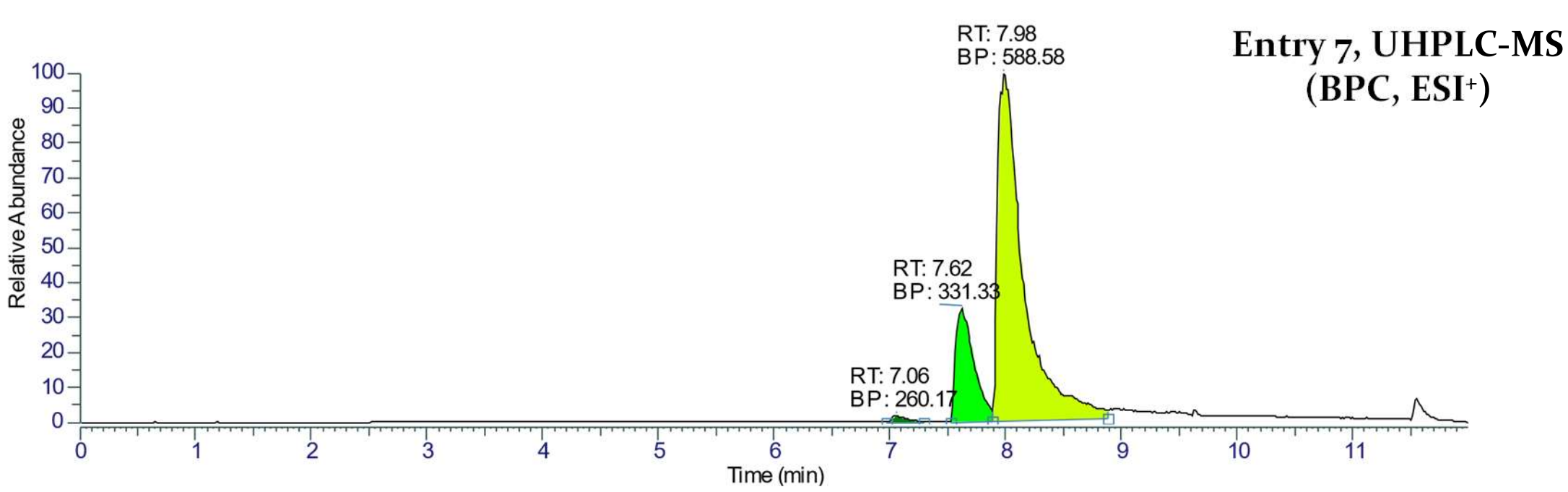

LR HPLC-MS BP spectrum, ESI+, m/z 200–2000 (BP at *t*=7.98 min)

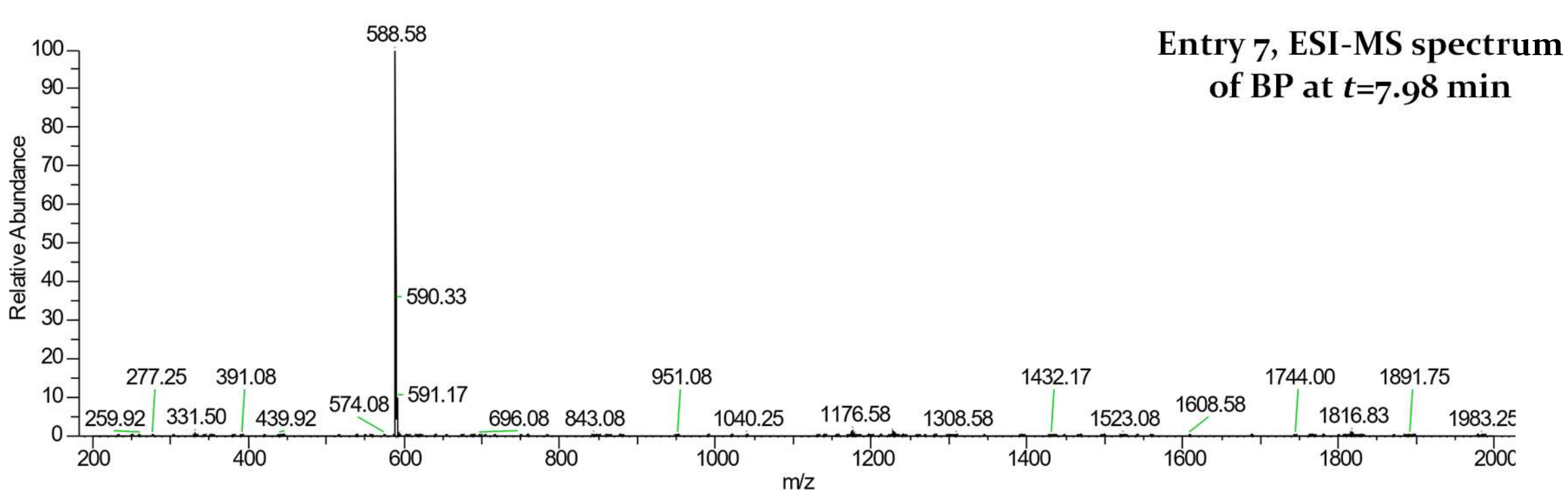

Entry 8 (1 eq I_2 was added to 1:1 eq mixture of STF and *MS1,* followed by 1 eq *MS2*)

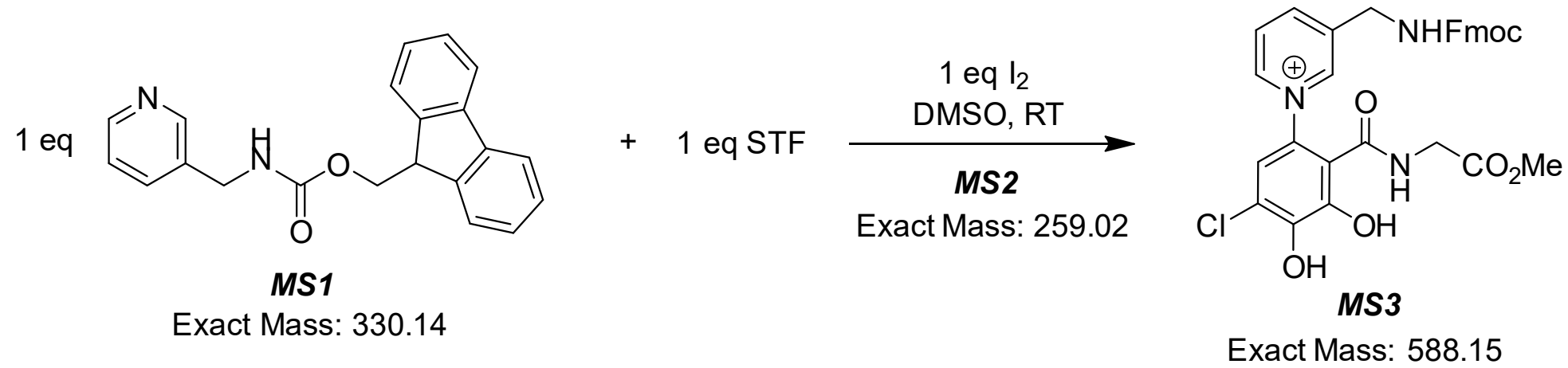

LR HPLC-MS chromatogram, UV 254 nm, C_{18}, 0–100% H_2O/MeCN+0.1% FA (linear gradient, 0-12 min), ESI⁺

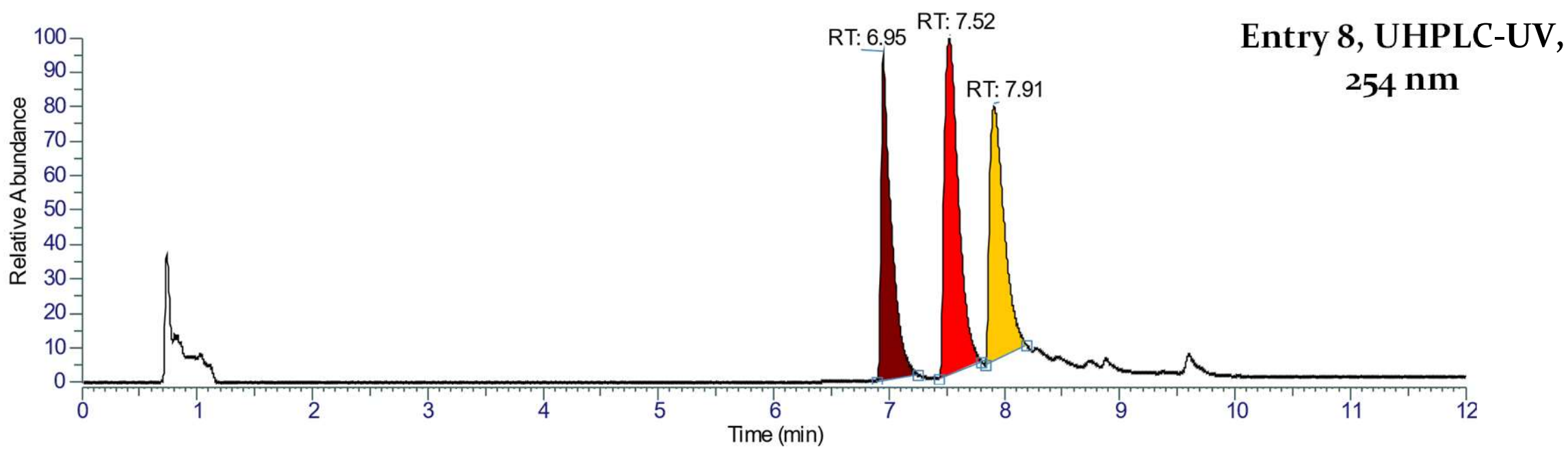

PEAK LIST											
Index	RT (Min)	Start RT	End RT	Peak Area	Peak Height	Baseline V	SignalToNo	TraceId	% Area	% Height	Integration Method
1	6.95	6.90	7.25	10473310.41	1629346.71	0.35	2568.01	Trace1	27.00	34.78	Automated - ICIS
2	7.53	7.44	7.80	13154475.99	1445545.26	0.36	2278.32	Trace1	33.92	30.86	Automated - ICIS
3	7.91	7.83	8.19	15158589.46	1609495.19	0.36	2536.73	Trace1	39.08	34.36	Automated - ICIS

LR HPLC-MS BPC chromatogram of entry 8, ESI⁺, m/z 200–2000

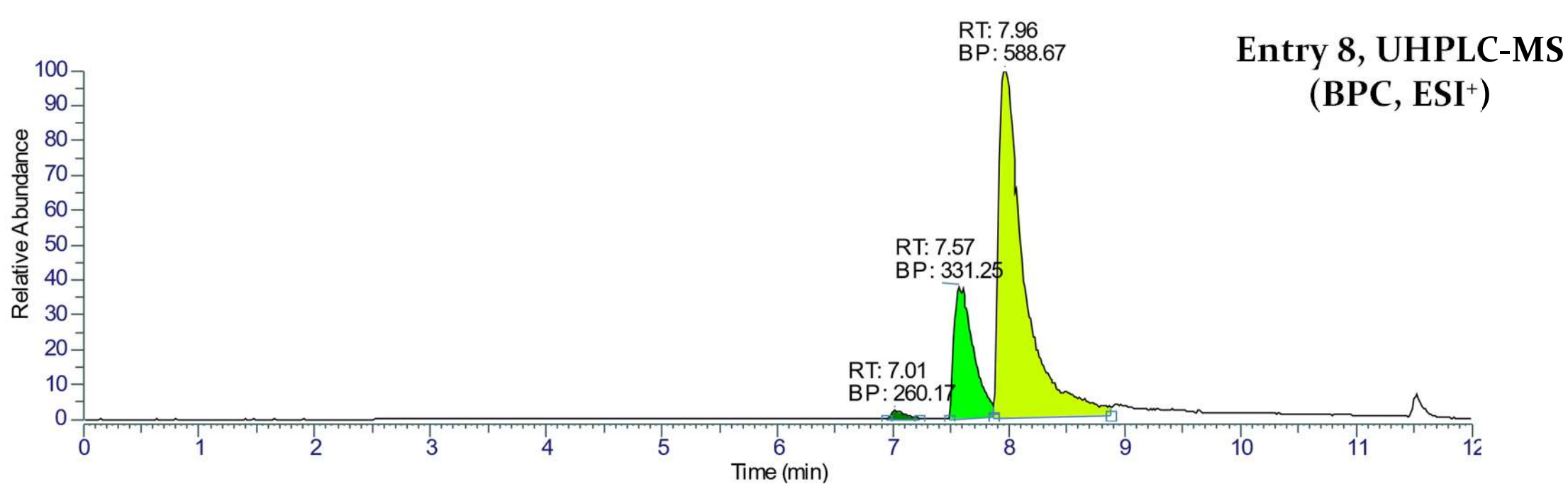

LR HPLC-MS BP spectrum, ESI⁺, m/z 200–2000 (BP at *t*=7.96 min)

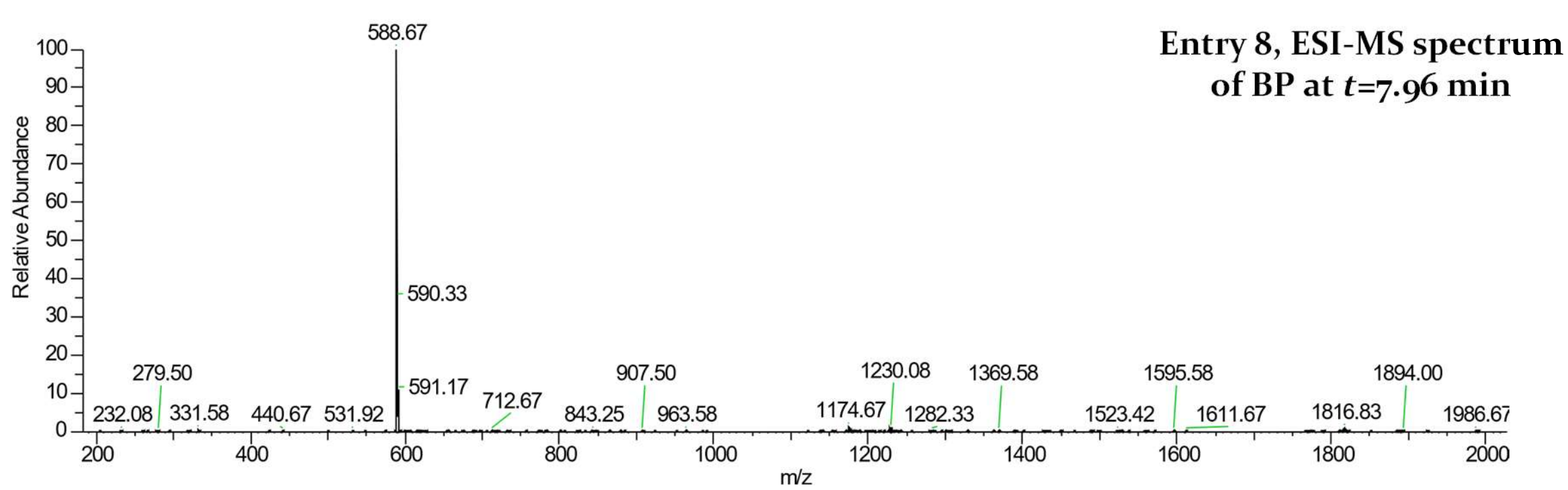

Entry 9 (1 eq I_2 was added to a mixture of 2 eq STF and 1 eq *MS1,* followed by 1 eq *MS2*)

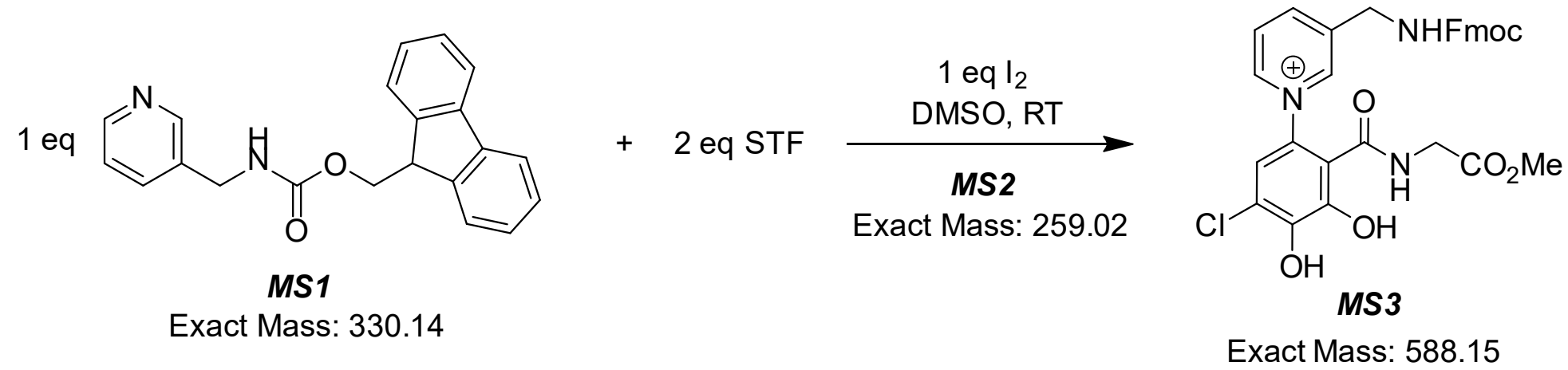

LR HPLC-MS chromatogram, UV 254 nm, C_{18}, 0–100% H_2O/MeCN+0.1% FA (linear gradient, 0-12 min), ESI⁺

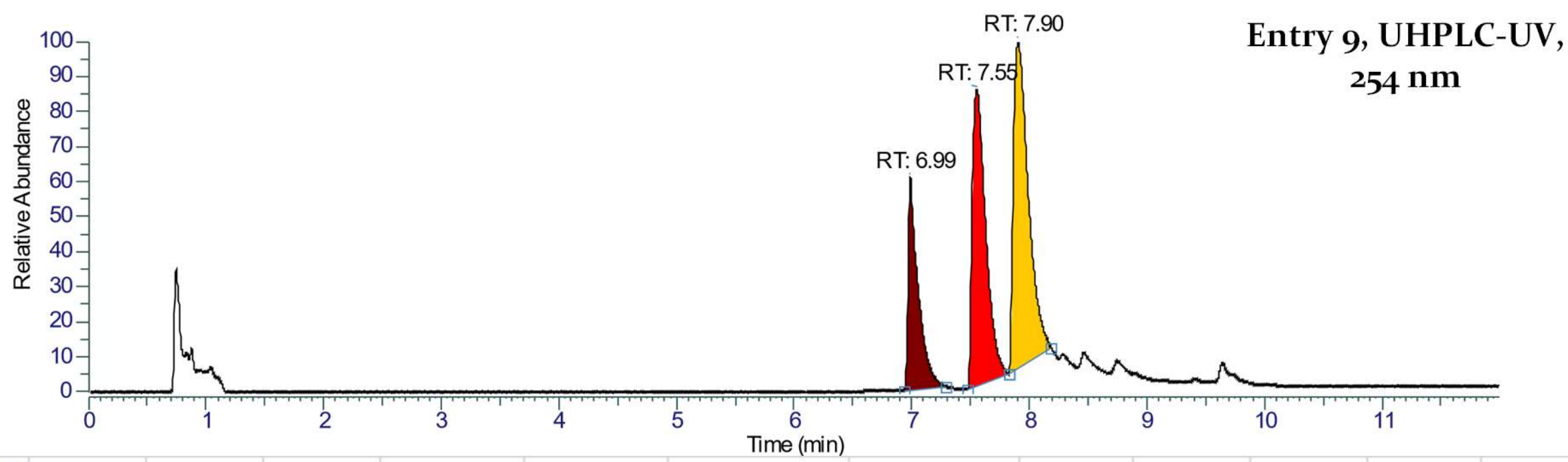

PEAK LIST											
Index	RT (Min)	Start RT	End RT	Peak Area	Peak Height	Baseline Width	SignalToNoise	TraceId	% Area	% Height	Integration Method
1.00	6.99	6.94	7.28	6721002.17	1099876.21	0.35	1726.15	Trace1	18.98	26.32	Automated - ICIS
2.00	7.56	7.48	7.82	10796168.04	1285944.01	0.34	2018.17	Trace1	30.48	30.77	Automated - ICIS
3.00	7.90	7.83	8.19	17901173.47	1793412.63	0.36	2814.59	Trace1	50.54	42.91	Automated - ICIS

LR HPLC-MS BPC chromatogram of entry 9, ESI⁺, m/z 200–2000

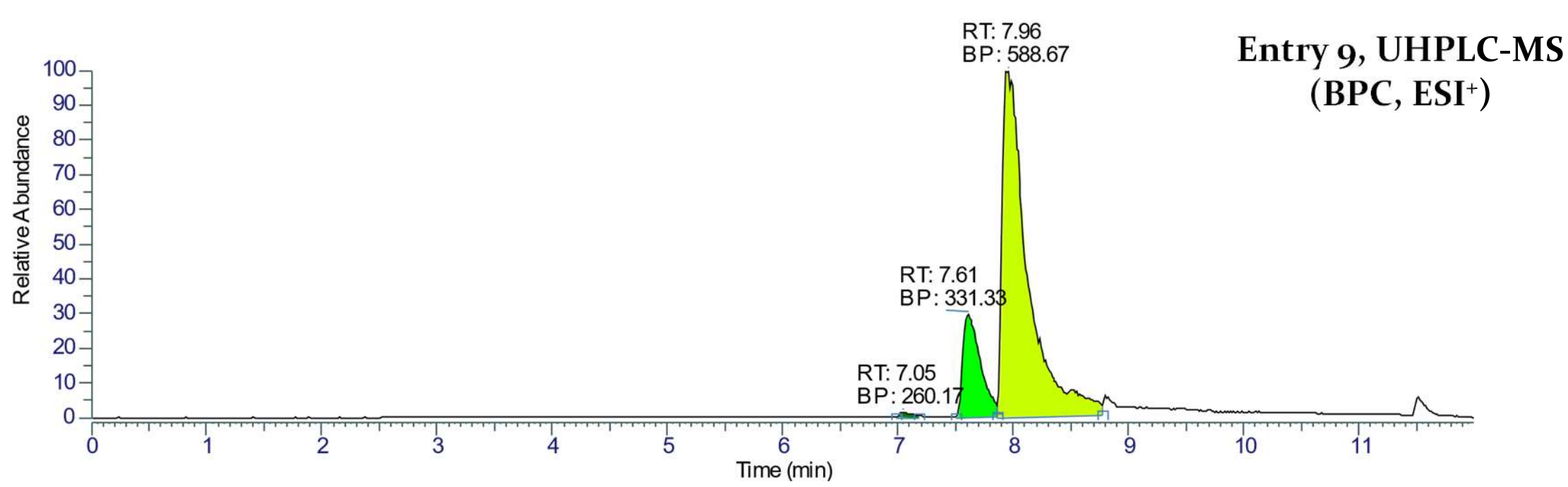

LR HPLC-MS BP spectrum, ESI⁺, m/z 200–2000 (BP at *t*=7.96 min)

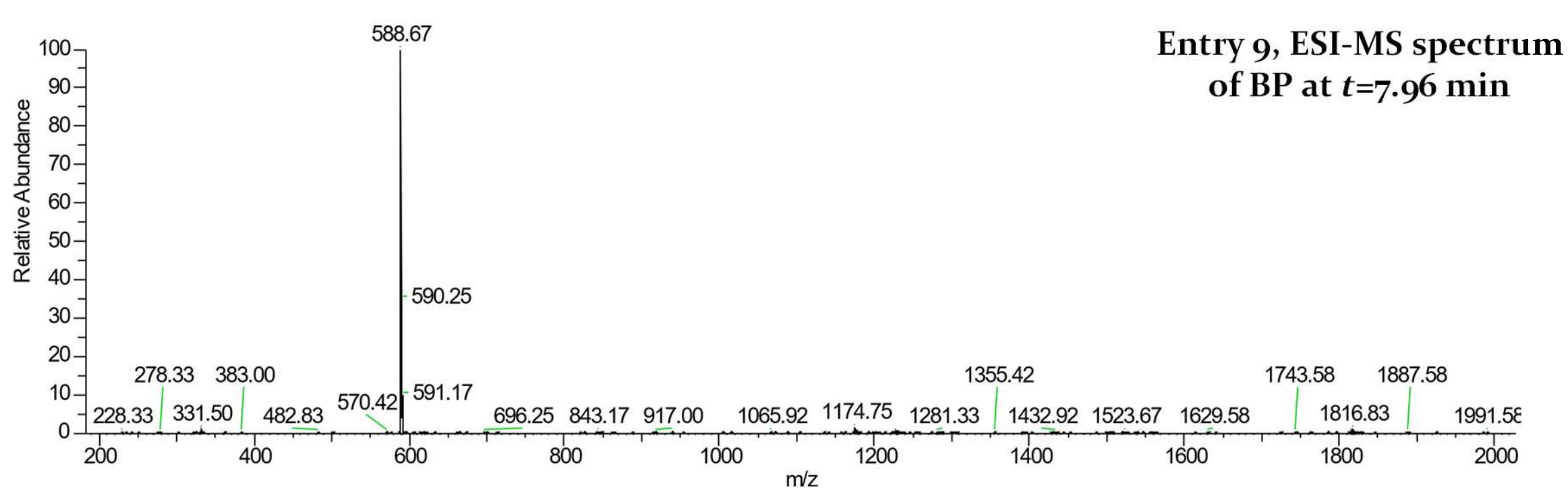

Entry 10 (1 eq I_2 was added to a mixture of 1 eq STF and 2 eq *MS1,* followed by 1 eq *MS2*)

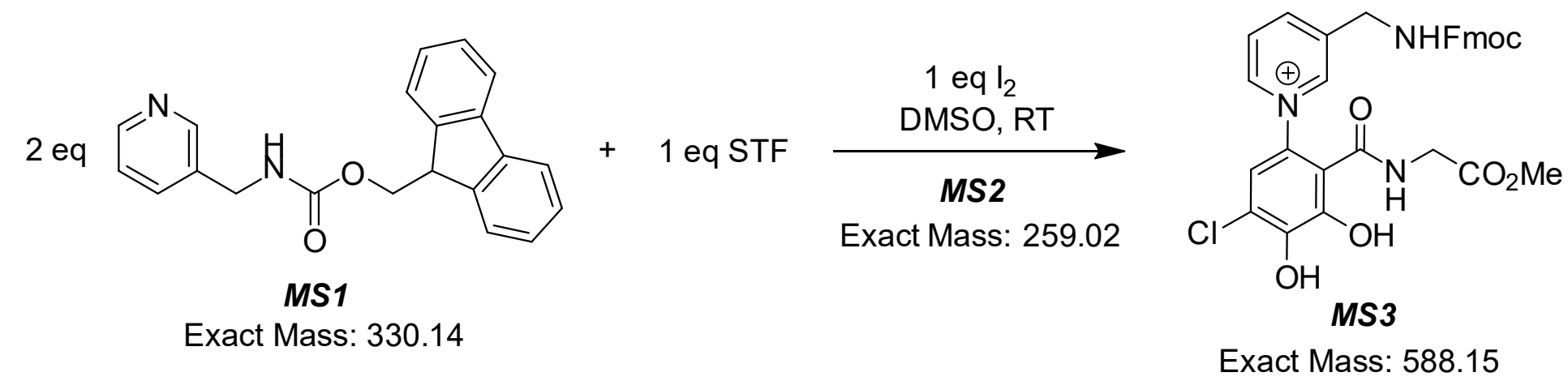

LR HPLC-MS chromatogram, UV 254 nm, C_{18}, 0–100% H_2O/MeCN+0.1% FA (linear gradient, 0-12 min), ESI$^+$

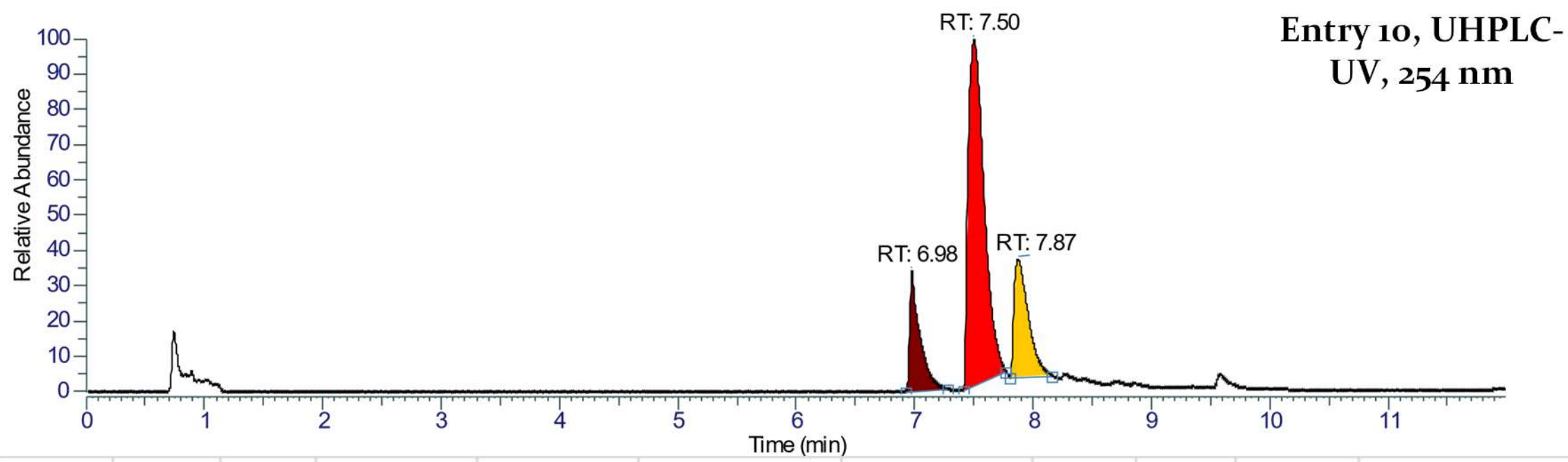

PEAK LIST											
Index	RT (Min)	Start RT	End RT	Peak Area	Peak Height	Baseline Width	SignalToNoise	TraceId	% Area	% Height	Integration Method
1	6.98	6.93	7.28	9057689.32	1456759.12	0.35	1988.25	Trace1	17.98	26.70	Automated - ICIS
2	7.50	7.41	7.77	25890483.76	2256585.81	0.36	3079.90	Trace1	51.41	41.35	Automated - ICIS
3	7.88	7.81	8.16	15416165.42	1743630.20	0.35	2379.79	Trace1	30.61	31.95	Automated - ICIS

LR HPLC-MS BPC chromatogram of entry 10, ESI$^+$, m/z 200–2000

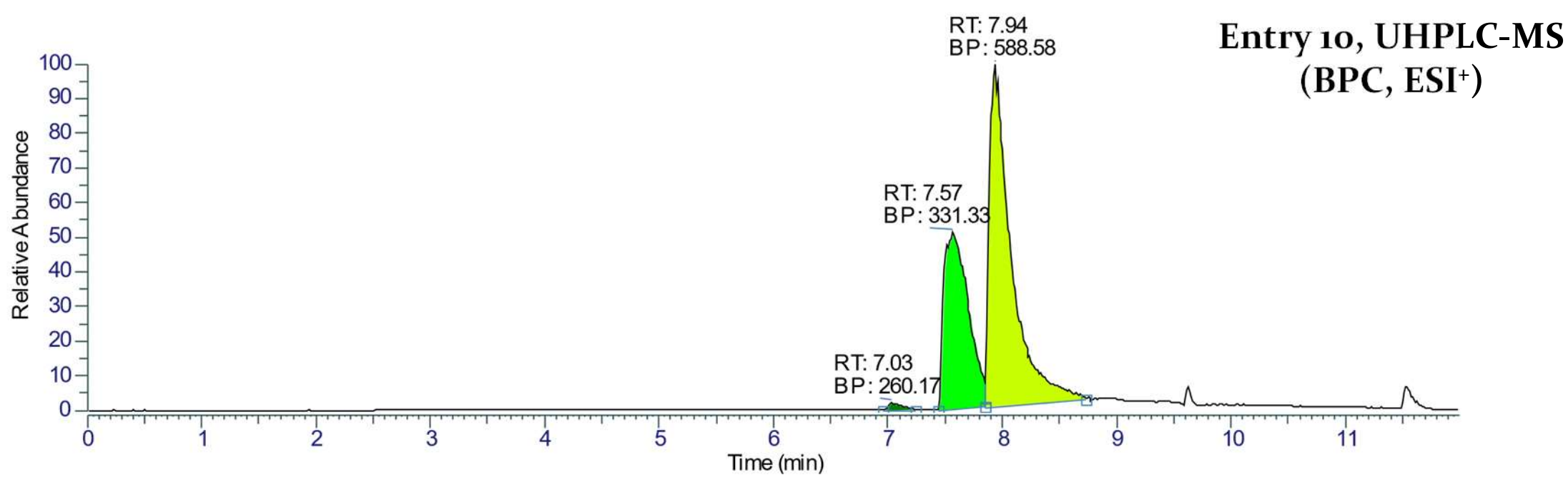

LR HPLC-MS BP spectrum, ESI$^+$, m/z 200–2000 (BP at t=7.94 min)

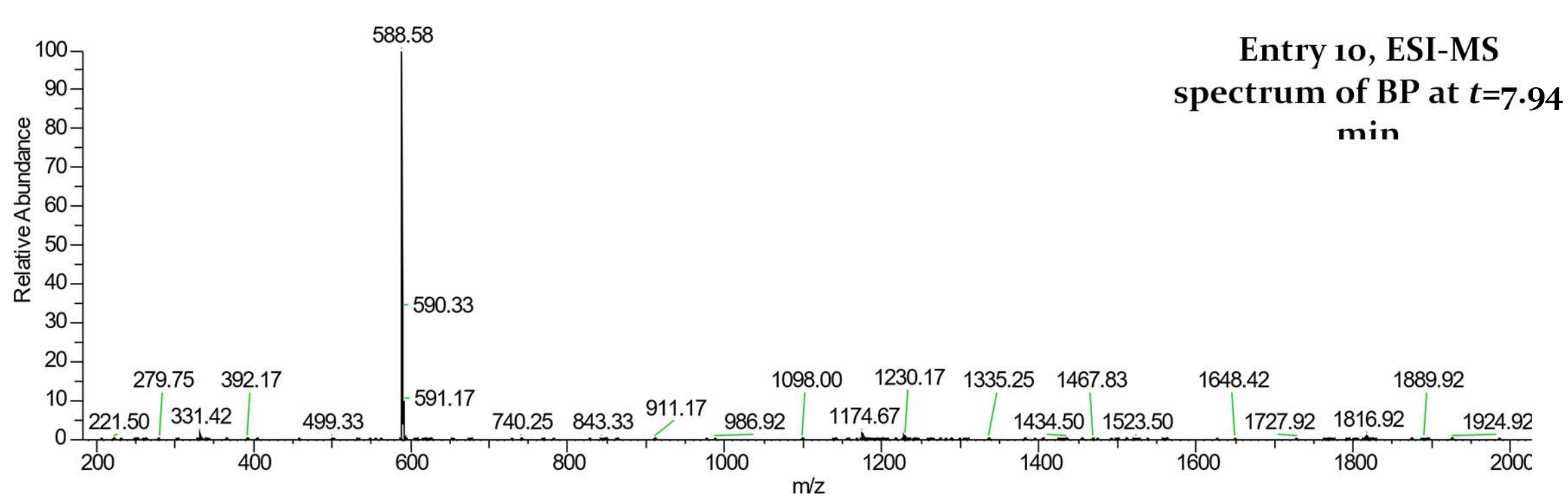

Entry 11 (1 eq of bis(3-methylpyridine) iodonium was added to 1 eq *MS2*)

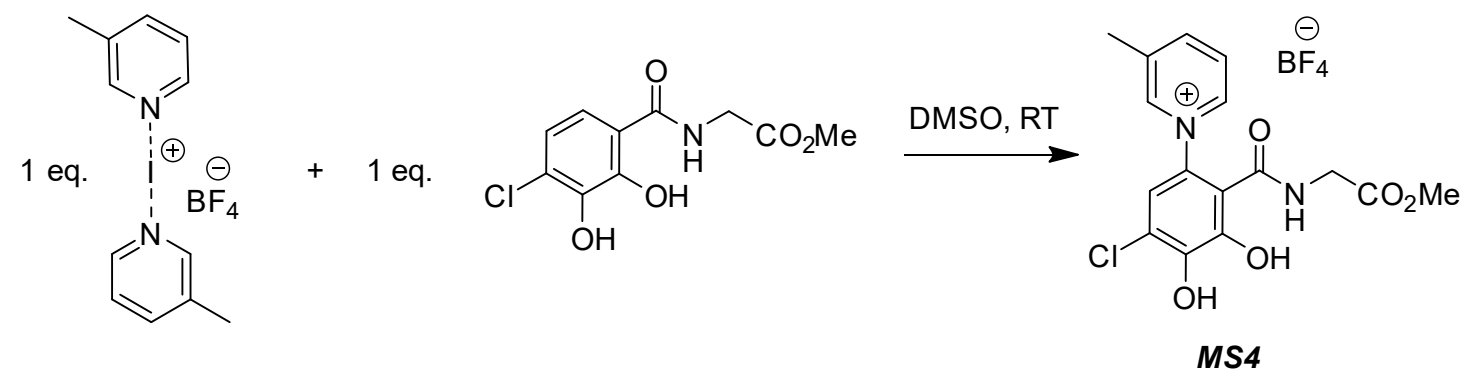

LR HPLC-MS chromatogram, UV 254 nm, C_{18}, 0–100% H_2O/MeCN+0.1% FA (linear gradient, 0-12 min), ESI$^+$

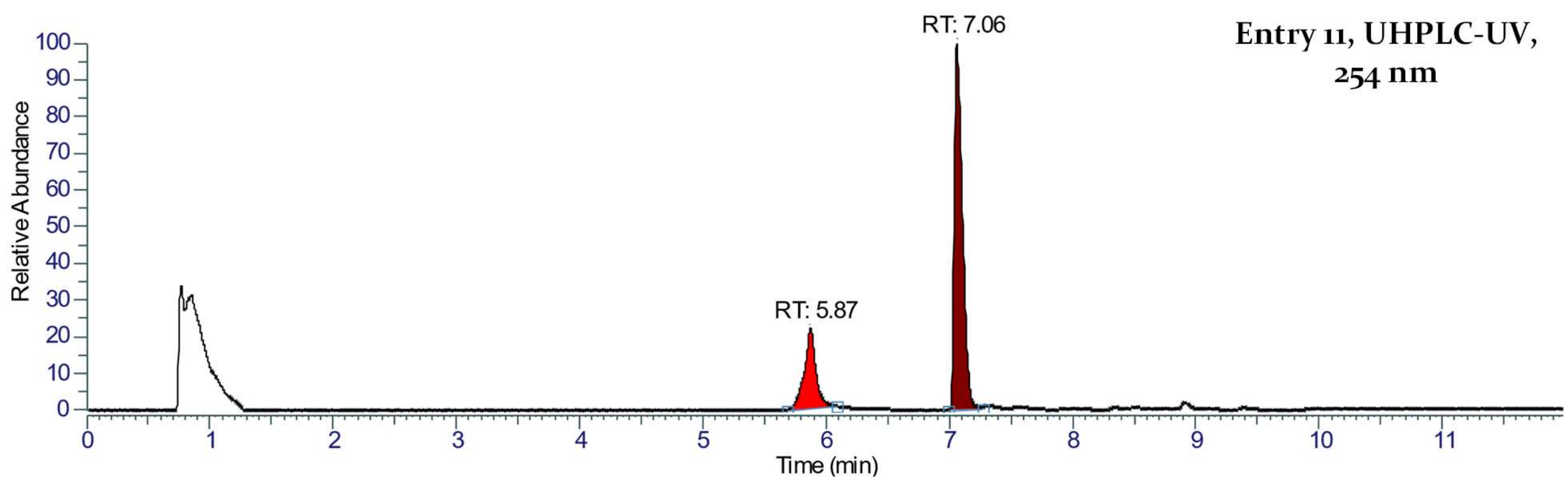

Index	RT (Min)	Start RT	End RT	Peak Area	Peak Height	Baseline Width	SignalToNoise	% Area	% Height	Integration
1	5.873333	5.683333	6.09	3834399.06	579847.92	0.41	3587.28	23.69	17.96	Automated - ICIS
2	7.06	6.996667	7.27	12348404.63	2648215.74	0.27	16383.42	76.31	82.04	Automated - ICIS

LR HPLC-MS BPC chromatogram of entry 11, ESI$^+$, m/z 200–2000

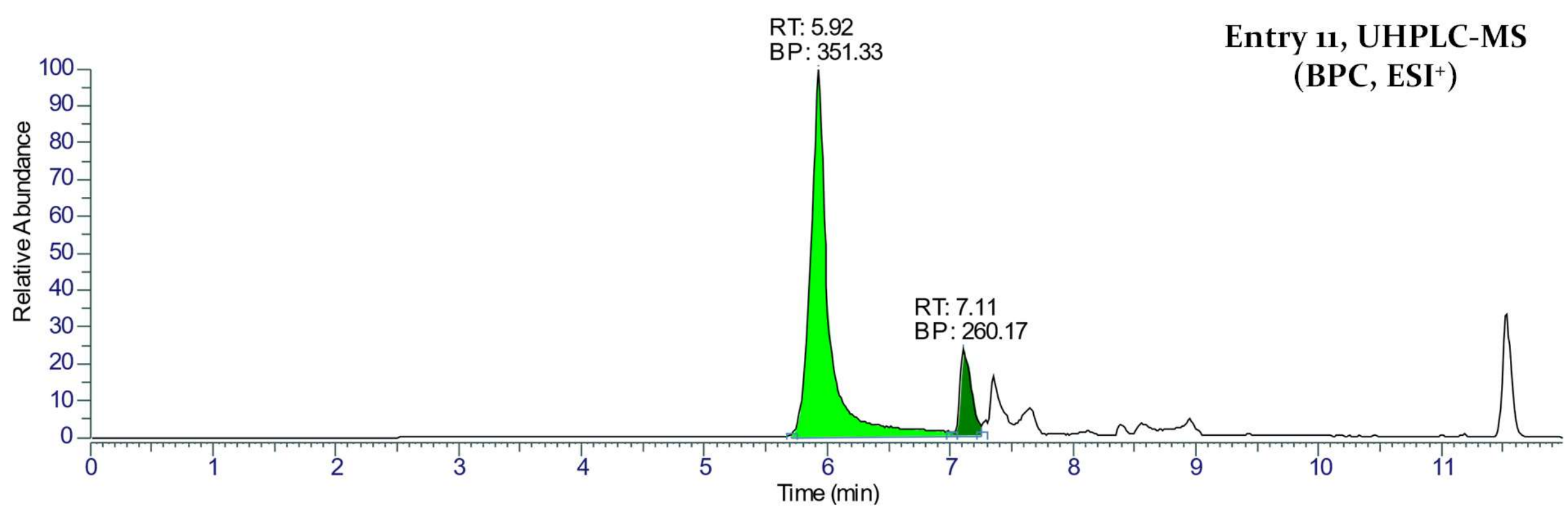

LR HPLC-MS BP spectrum, ESI$^+$, m/z 200–2000 (BP at *t* = 5.92 min)

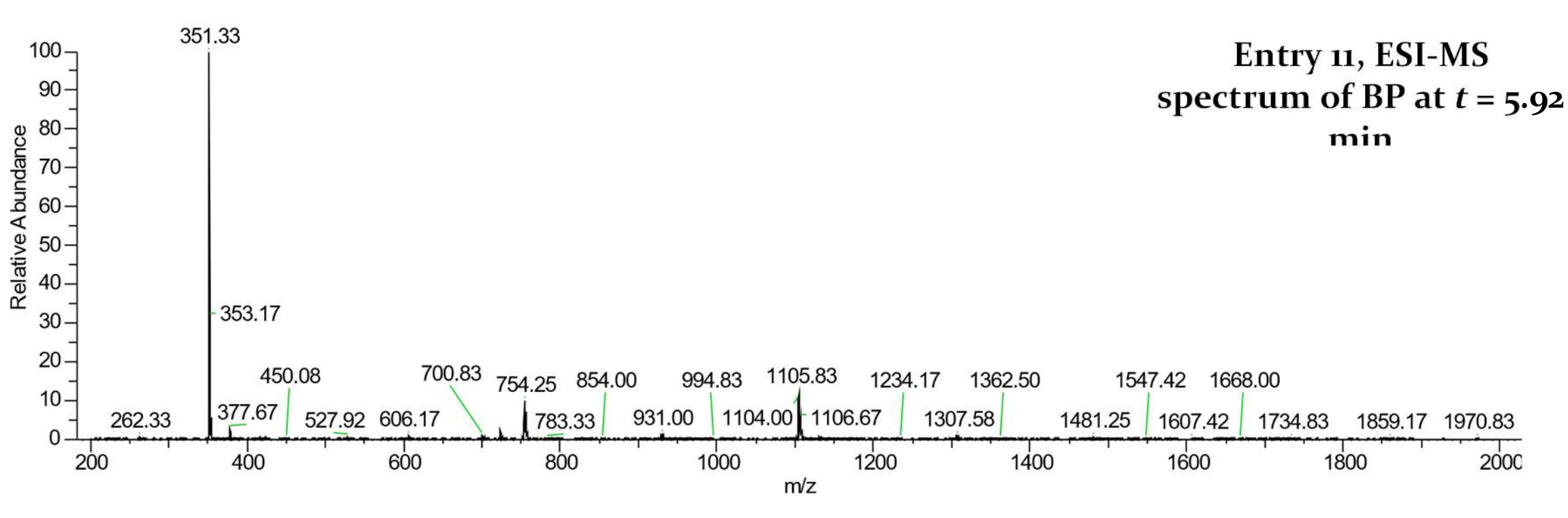

Entry 12 (3 eq of bis(3-methylpyridine) iodonium was added to 1 eq Enterobactin)

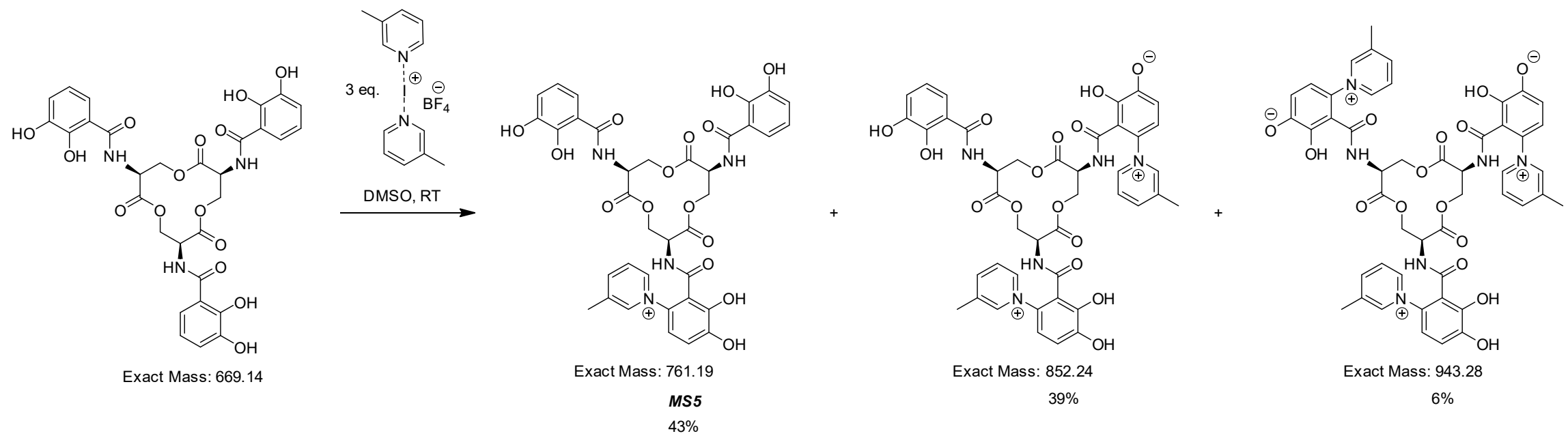

LR HPLC-MS chromatogram, UV 254 nm, C_{18}, 0–100% H_2O/MeCN+0.1% FA (linear gradient, 0-12 min), ESI$^+$

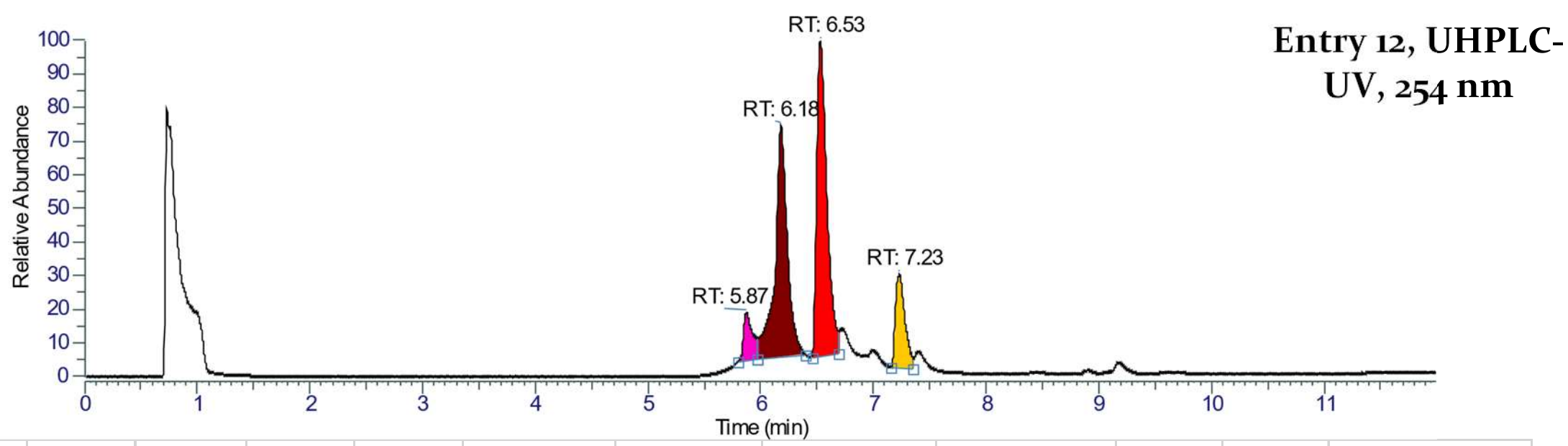

Index	RT (Min)	RT (Sec)	Start RT	End RT	Peak Area	Peak Height	Baseline Width	SignalToNoise	% Area	% Height	Integration Method
1	5.87	352.40	5.80	5.98	836178.82	145357.73	0.17	695.61	5.97	6.92	Automated - ICIS
2	6.18	370.80	5.98	6.39	5450279.47	706844.02	0.42	3382.59	38.92	33.67	Automated - ICIS
3	6.53	391.80	6.45	6.70	6032384.71	963023.38	0.24	4608.54	43.08	45.88	Automated - ICIS
4	7.23	433.80	7.16	7.36	1684770.03	283963.81	0.20	1358.91	12.03	13.53	Automated - ICIS

LR HPLC-MS BPC chromatogram of entry 12, ESI$^+$, m/z 200–2000

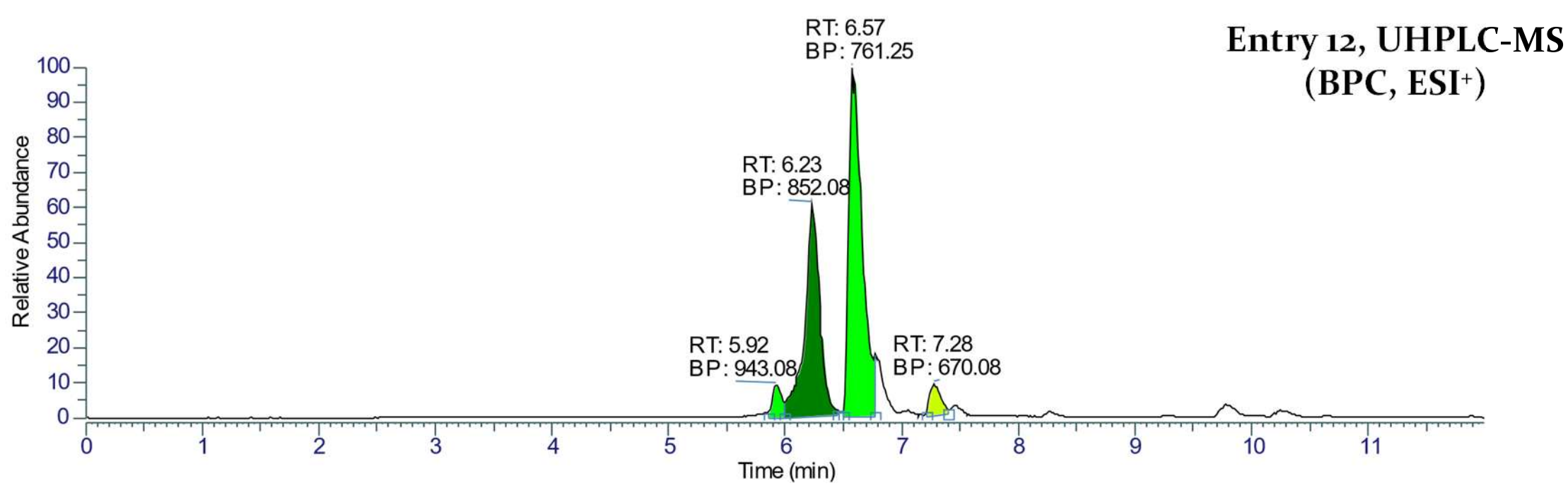

LR HPLC-MS BP spectrum, ESI$^+$, m/z 200–2000 (BP at t = 6.57 min)

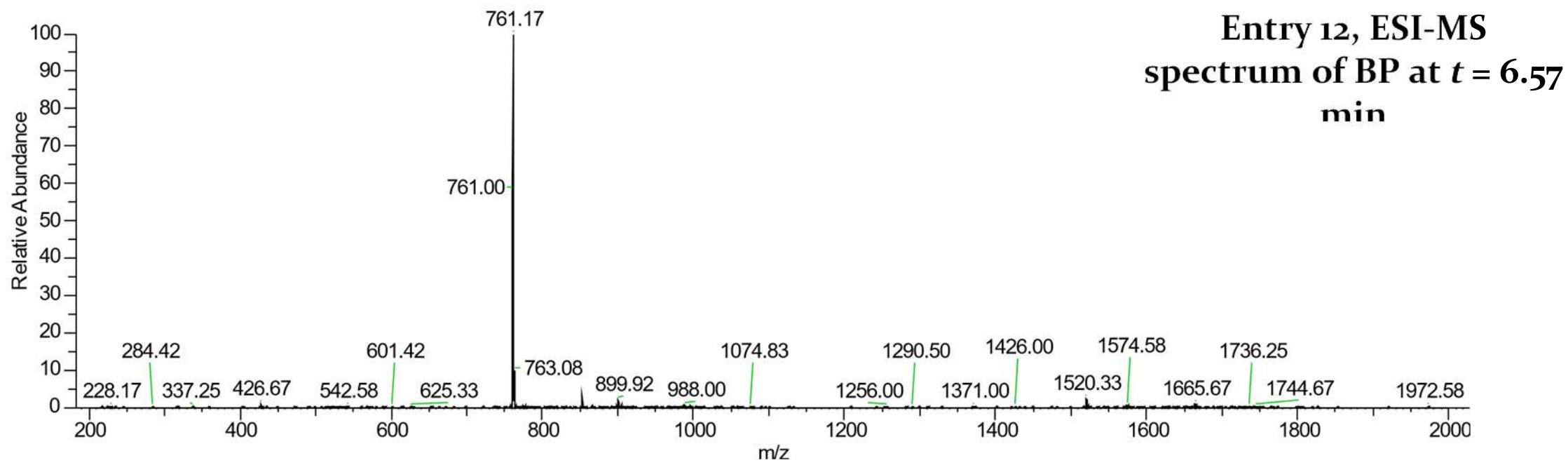
Entry 12, ESI-MS spectrum of BP at t = 6.57 min
Relative Abundance
m/z
761.17
761.00
763.08
284.42
601.42
1074.83
1290.50
1426.00
1574.58
1736.25
228.17
337.25
426.67
542.58
625.33
899.92
988.00
1256.00
1371.00
1520.33
1665.67
1744.67
1972.58

Entry 13 (2 eq STF was added to 1:1 eq mixture of *MS1* and *Enterobactin,* followed by 1eq I_2)

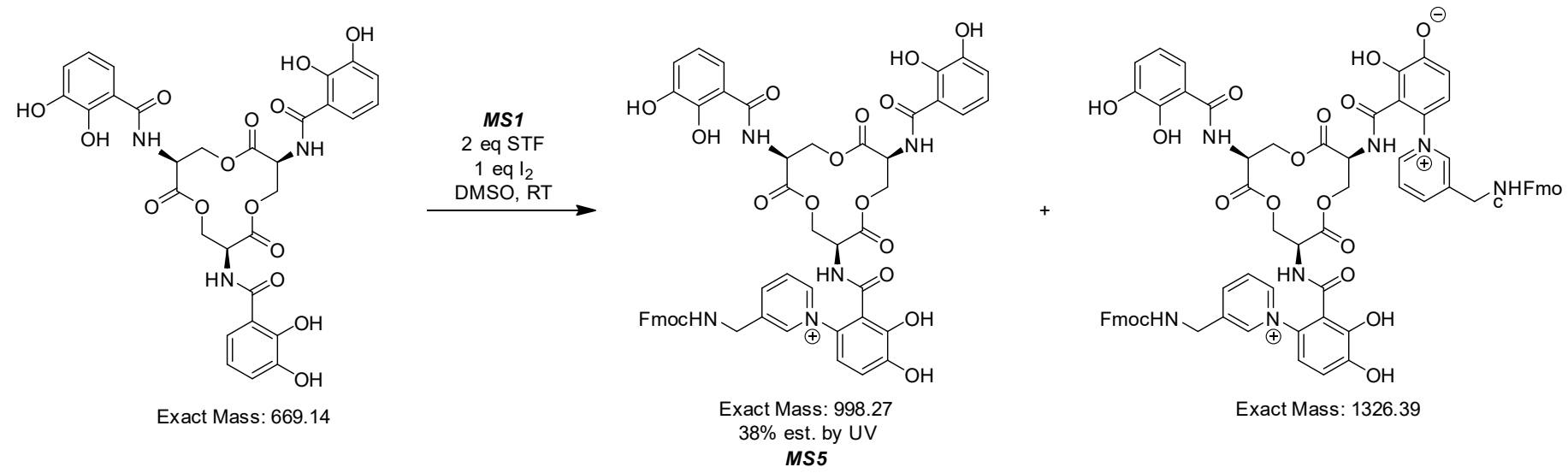

LR HPLC-MS chromatogram, UV 254 nm, C_{18}, 0–100% H_2O/MeCN+0.1% FA (linear gradient, 0-12 min), ESI⁺

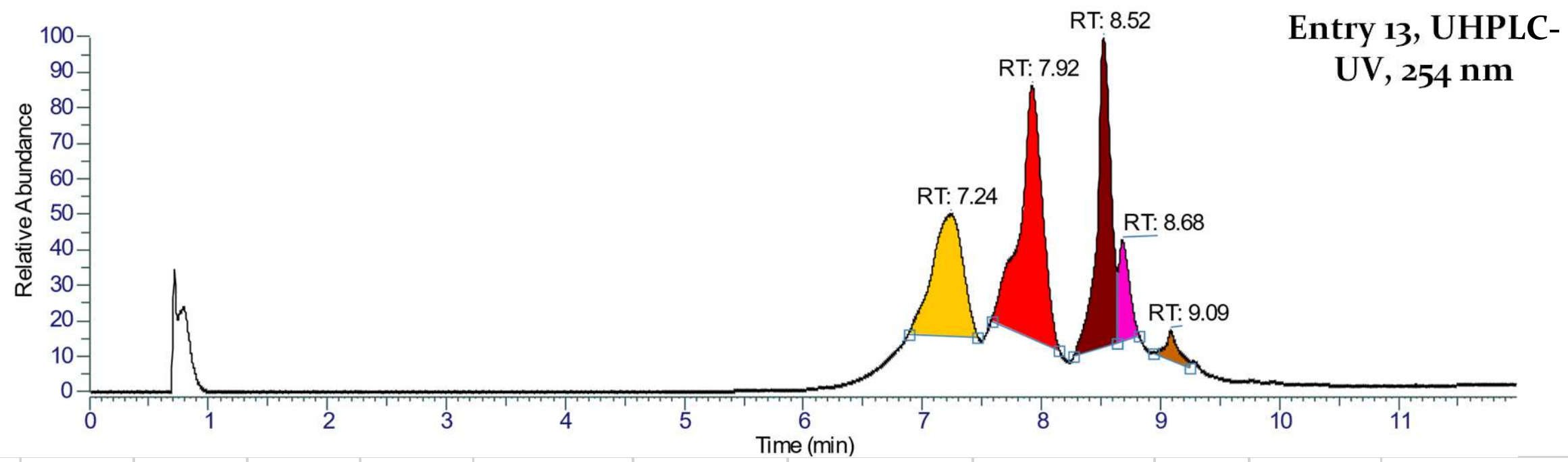

Index	RT (Min)	RT (Sec)	Start RT	End RT	Peak Area	Peak Height	Baseline Width	SignalToNoise	% Area	% Height	Integration Method
1	7.24	434.40	6.89	7.47	4416333.07	247323.36	0.58	2115.38	24.44	14.94	Automated - ICIS
2	7.92	475.40	7.58	8.15	6855497.34	514708.24	0.57	4402.35	37.93	31.09	Automated - ICIS
3	8.52	511.40	8.27	8.64	5106769.98	630659.64	0.37	5394.10	28.26	38.09	Automated - ICIS
4	8.68	520.80	8.64	8.82	1249572.29	204466.83	0.18	1748.83	6.91	12.35	Automated - ICIS
5	9.09	545.20	8.94	9.25	445419.95	58440.72	0.31	499.85	2.46	3.53	Automated - ICIS

LR HPLC-MS BPC chromatogram of entry 13, ESI⁺, m/z 200–2000

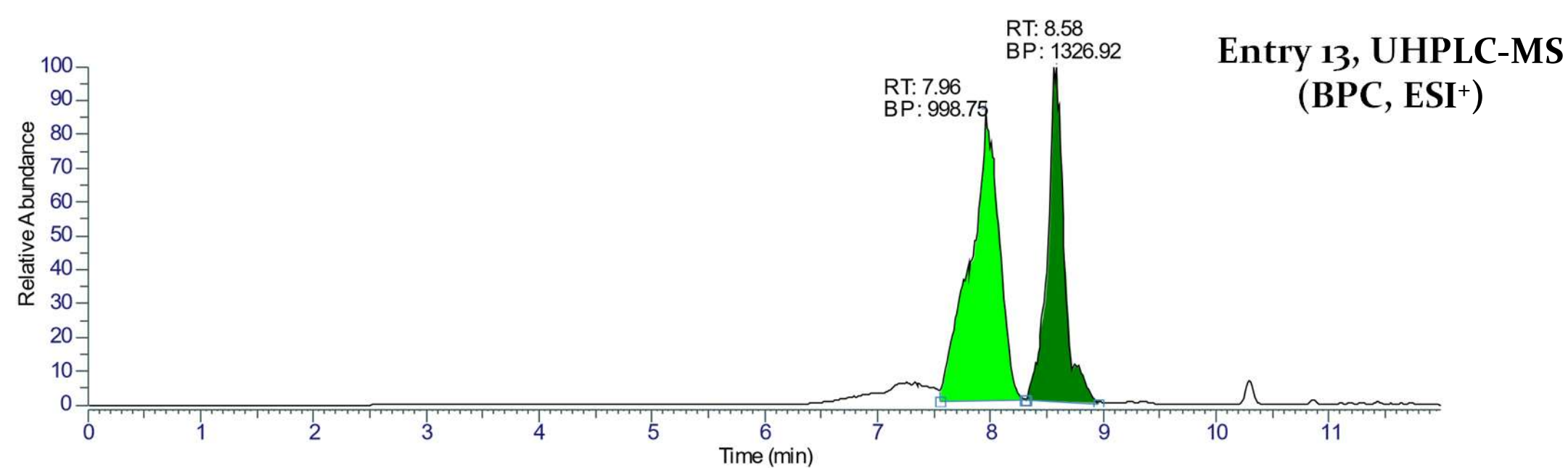

LR HPLC-MS BP spectrum, ESI⁺, m/z 200–2000 (BP at *t* = 7.96 min)

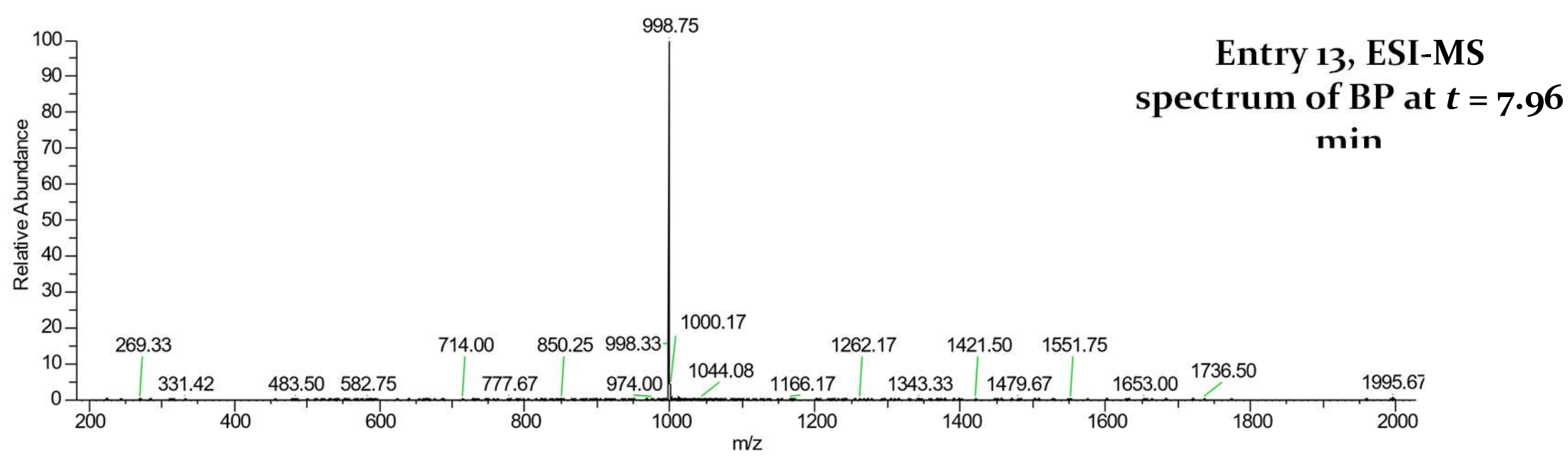

Comp. 1 (Enterobactin)

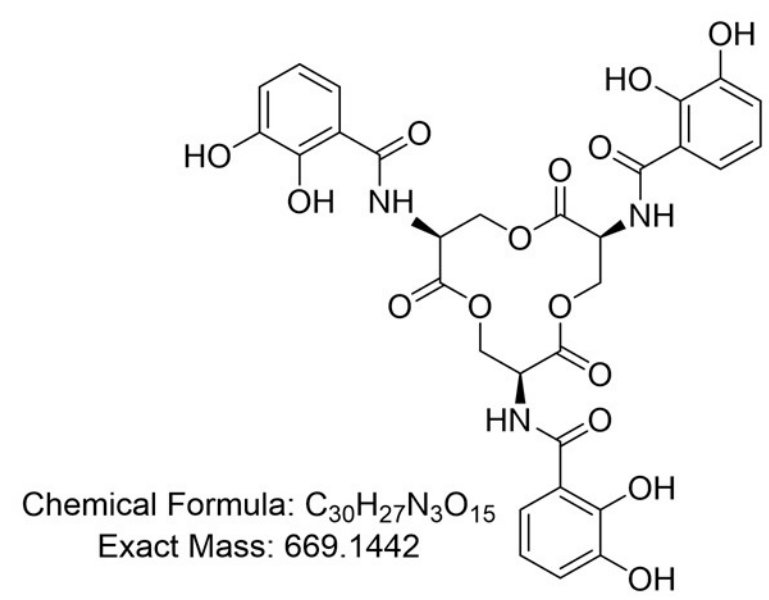

LR HPLC-MS chromatogram of comp. 1, UV 254 nm, C_{18}, 0–100% H_2O/MeCN+0.1% FA (linear gradient, 0-12 min), +ESI (pur. 99%)

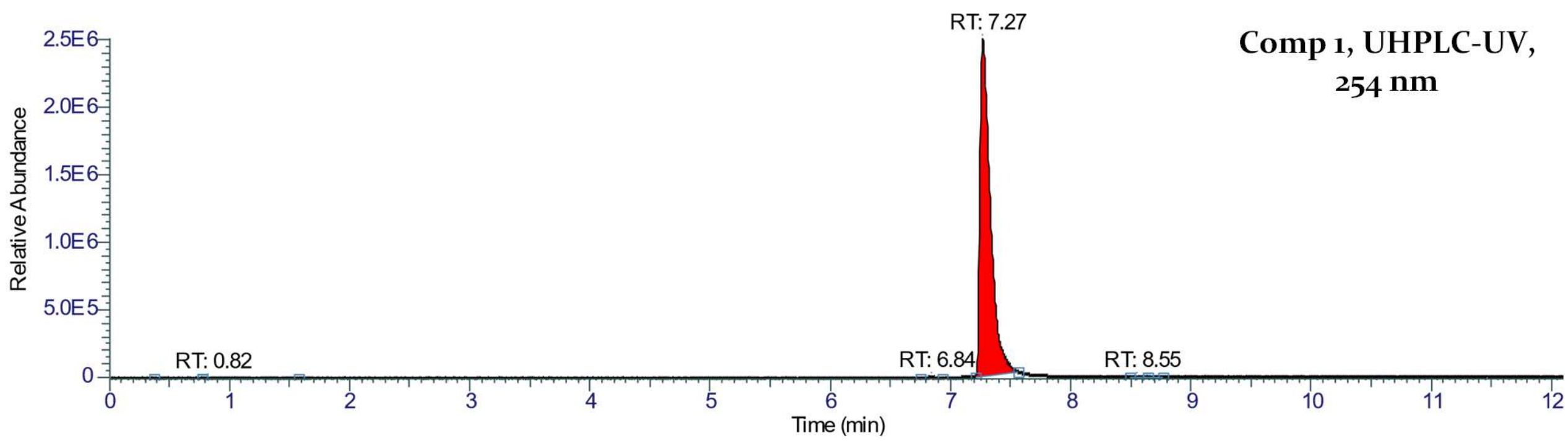

LR HPLC-MS BPC chromatogram of comp. 1, +ESI, m/z 200–2000

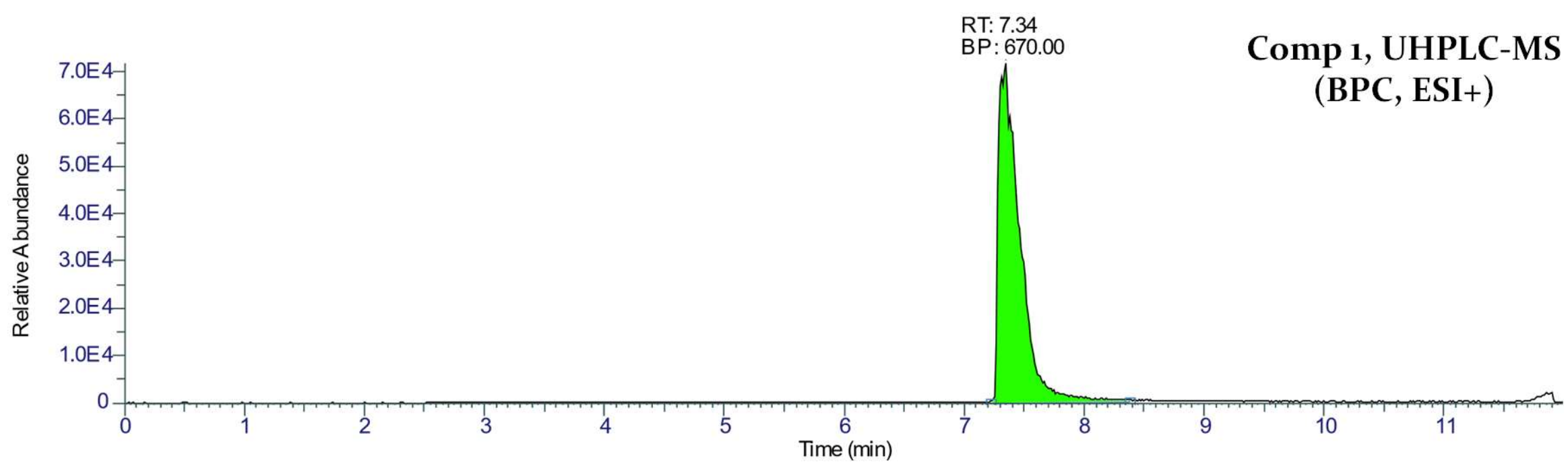

LR HPLC-MS BPC spectrum of comp. 1, +ESI, m/z 200–2000

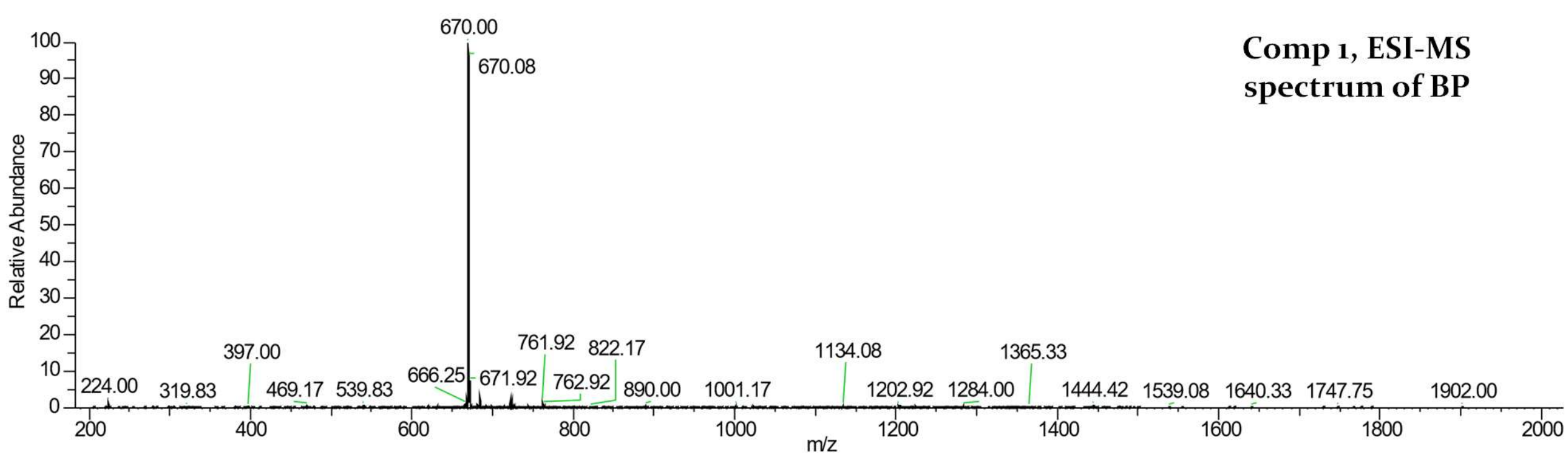

Comp. 2 (TRENCAM)

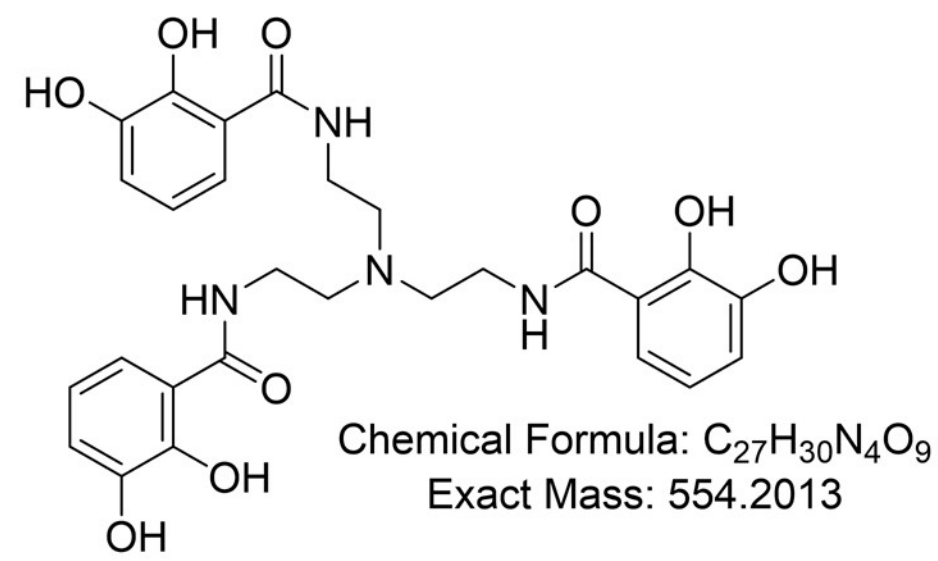

LR HPLC-MS chromatogram of comp. 2, UV 254 nm, C_{18}, 0–100% H_2O/MeCN+0.1% FA (linear gradient, 0-12 min), +ESI (pur. 100%)

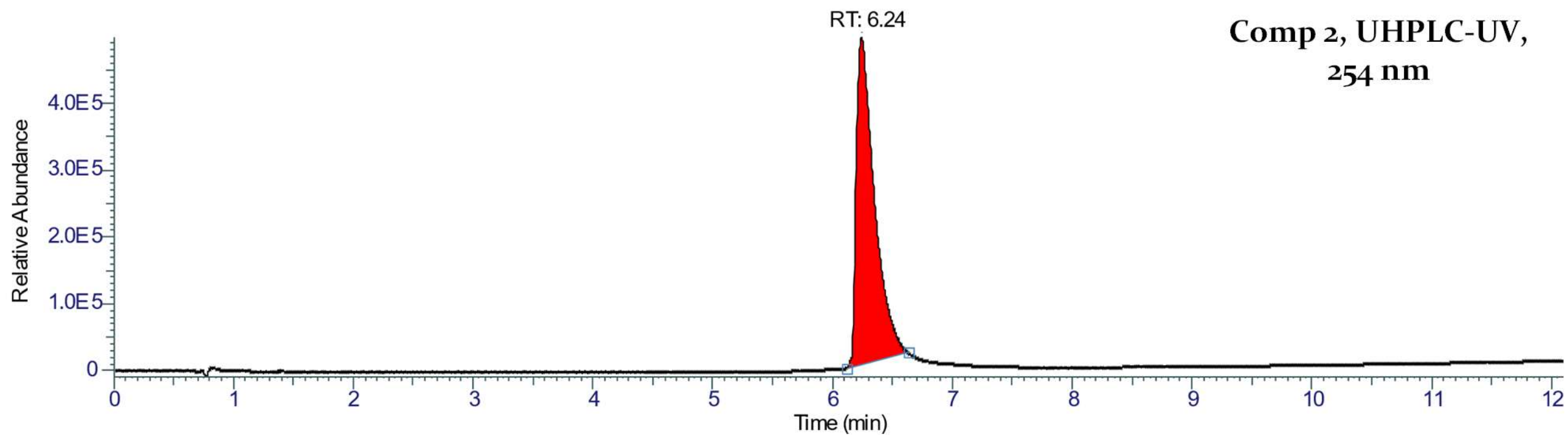

LR HPLC-MS BPC chromatogram of comp. 2, +ESI, m/z 200–2000

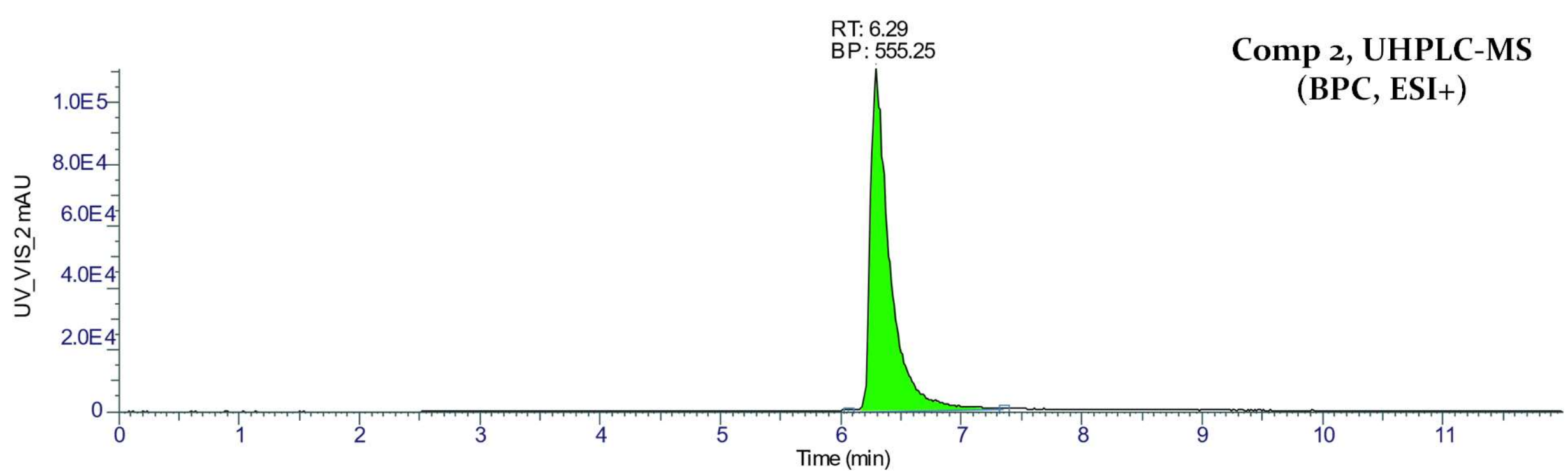

LR HPLC-MS BP spectrum of comp. 2, +ESI, m/z 200–2000

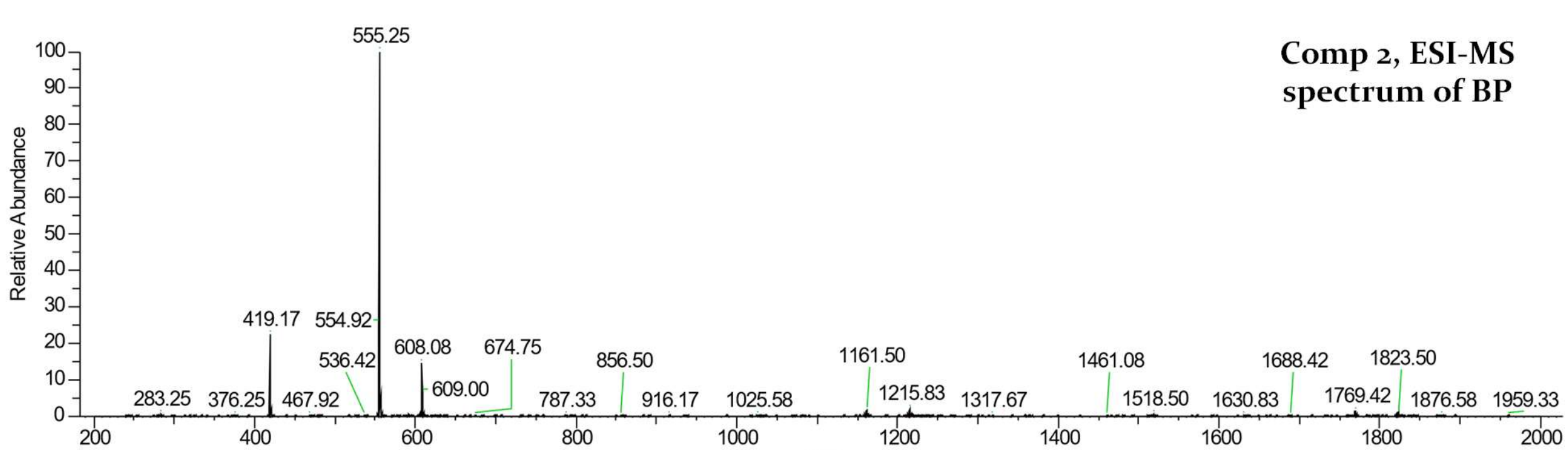

Comp. 4

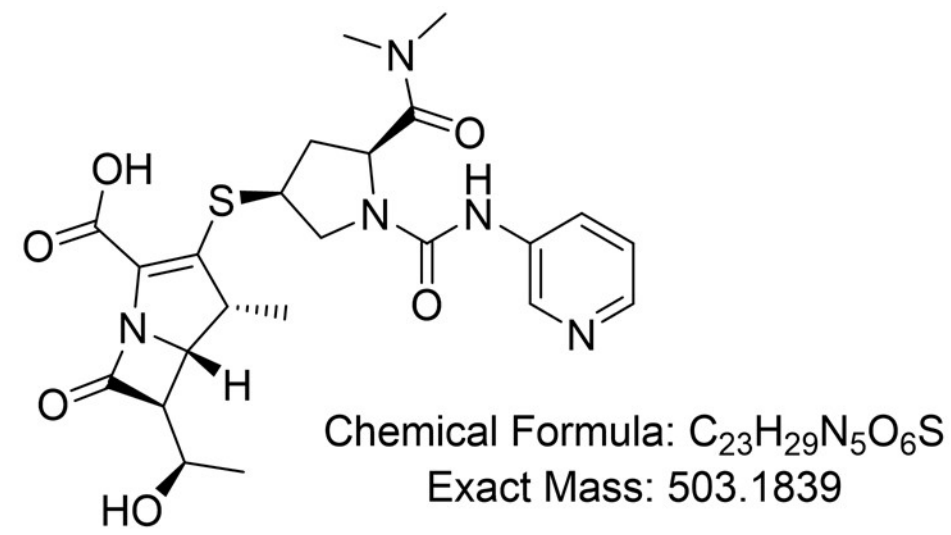

LR HPLC-MS chromatogram of comp. 4, UV 254 nm, C_{18}, 0–100% H_2O/MeCN+0.1% FA (linear gradient, 0-12 min), +ESI (pur. 90%)

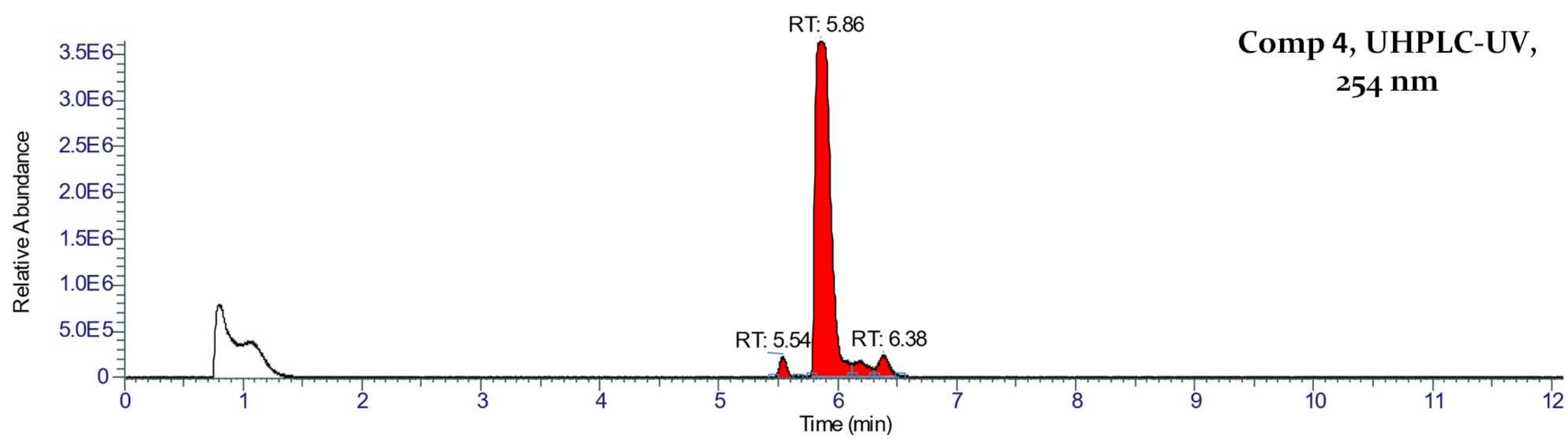

LR HPLC-MS BPC chromatogram of comp. 4, +ESI, m/z 200–2000

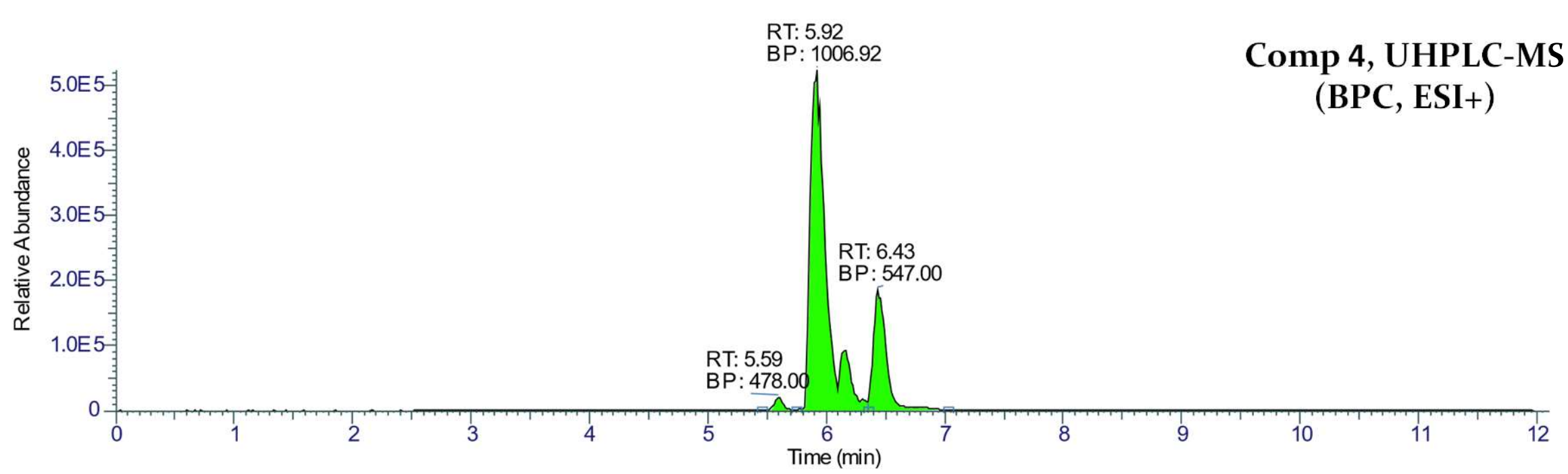

LR HPLC-MS BP spectrum of comp. 4, +ESI, m/z 200–2000

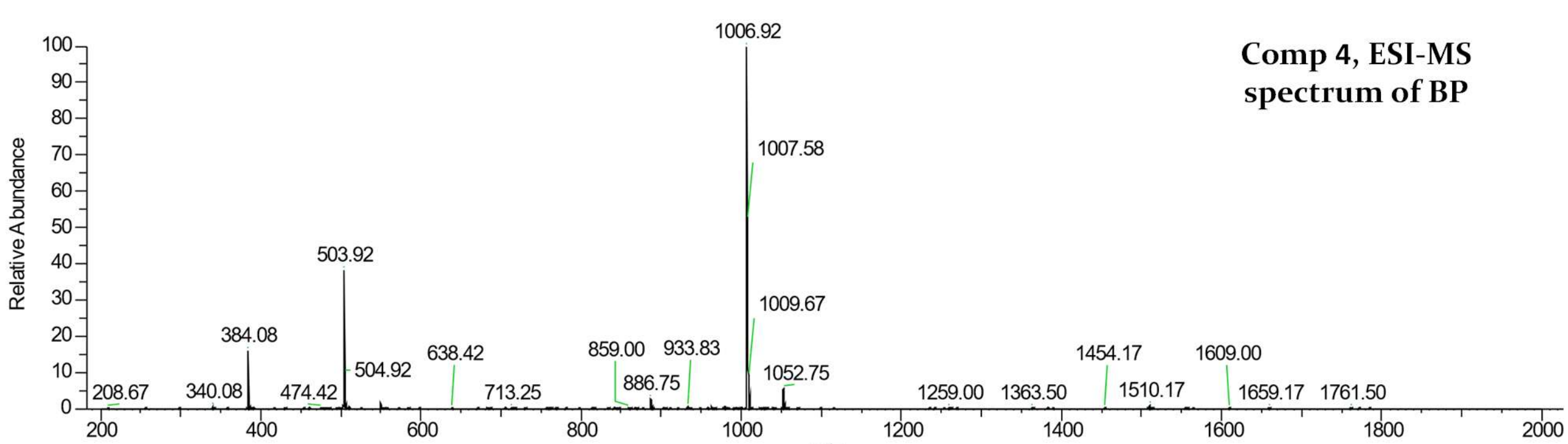

Comp. 5

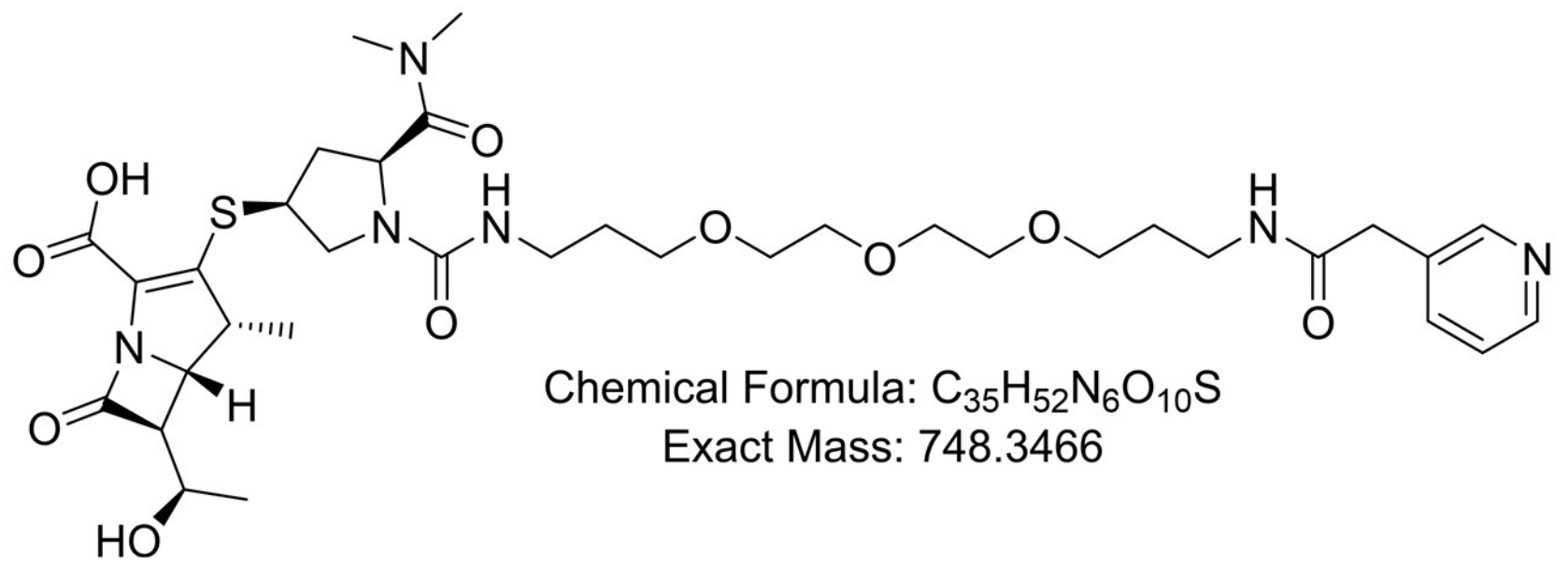

LR HPLC-MS chromatogram of comp. 5, UV 254 nm, C_{18}, 0–100% H_2O/MeCN+0.1% FA (linear gradient, 0-12 min), +ESI (pur. 98%)

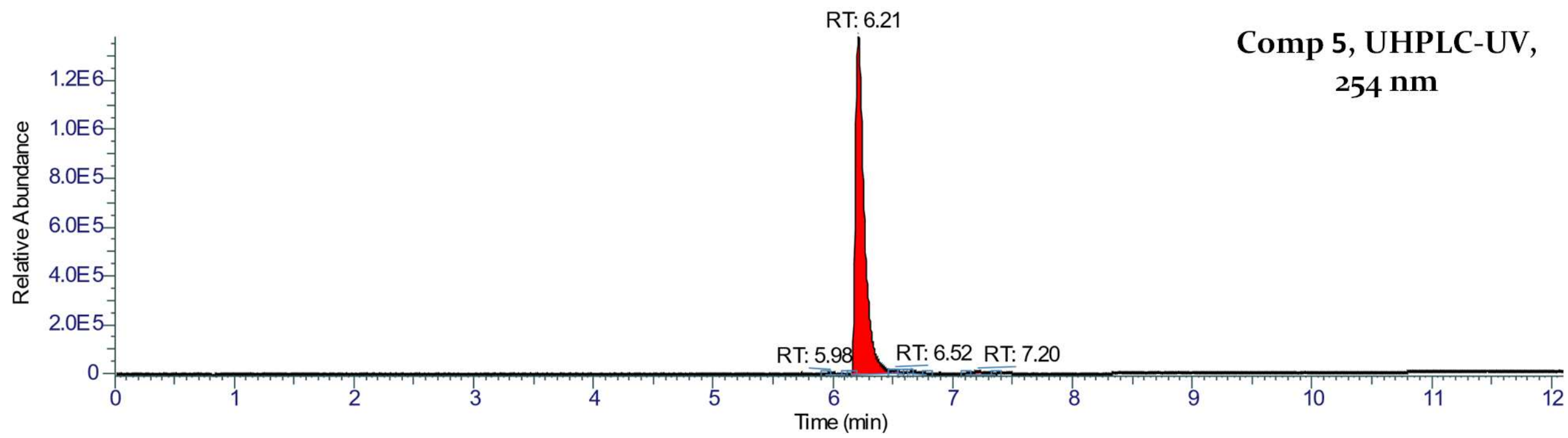

LR HPLC-MS BPC chromatogram of comp. 5, +ESI, m/z 200–2000

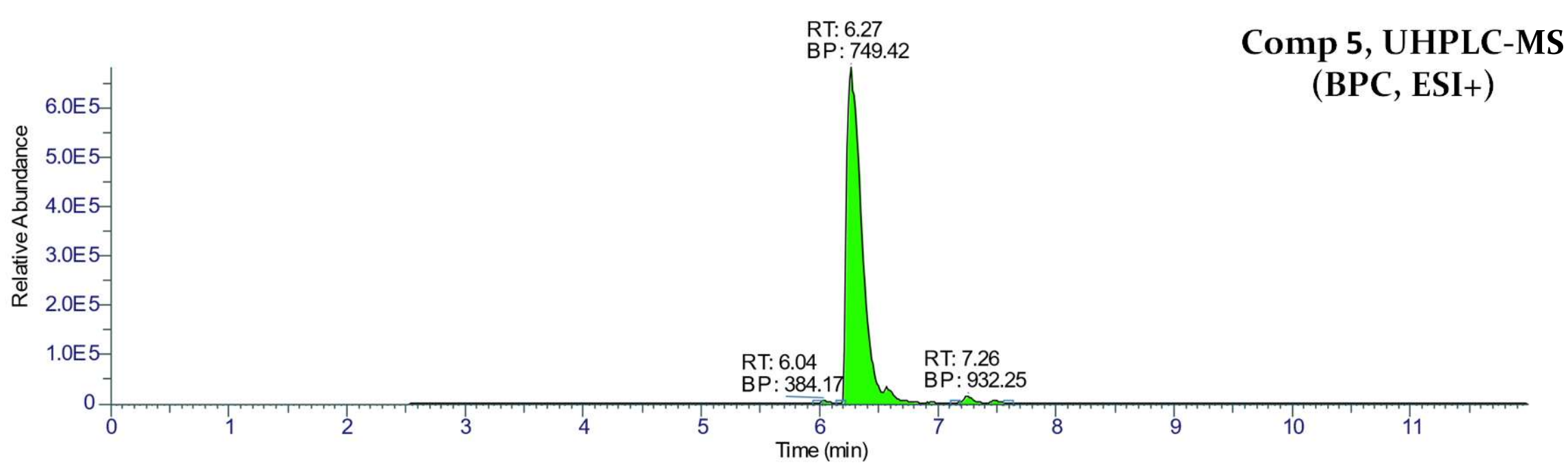

LR HPLC-MS BP spectrum of comp. 5, +ESI, m/z 200–2000

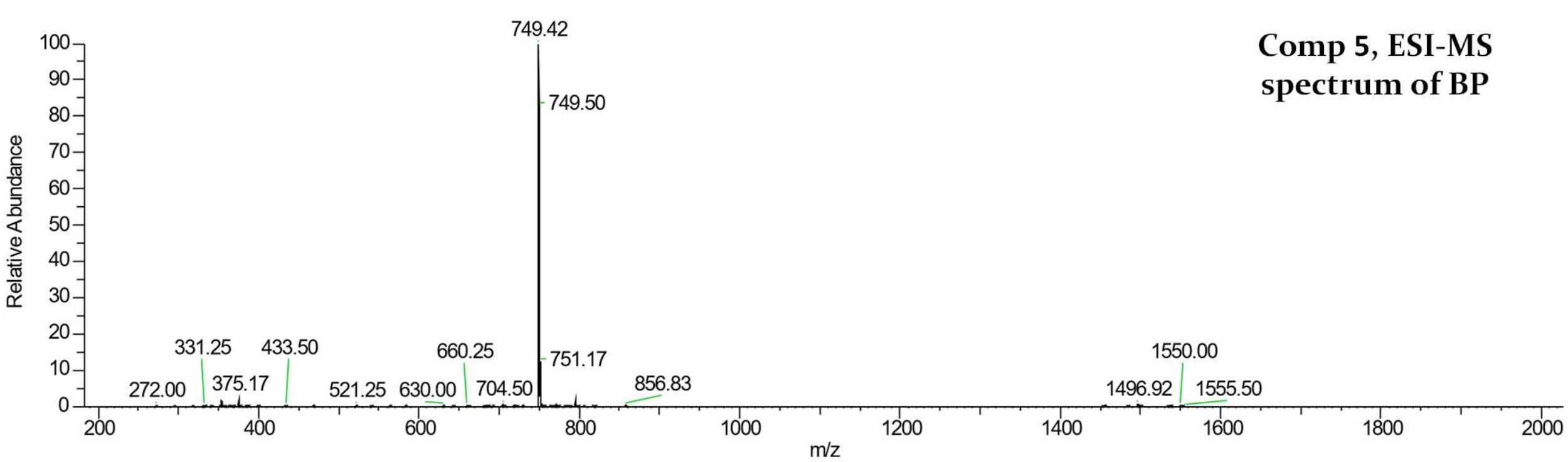

Comp. 6

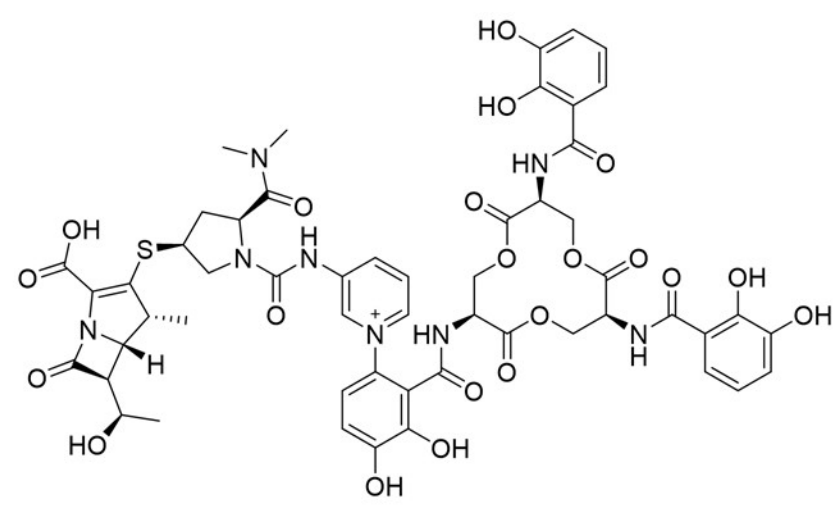

LR HPLC-MS chromatogram of comp. 6, UV 254 nm, C_{18}, 0–100% H_2O/MeCN+0.1% FA (linear gradient, 0-12 min), +ESI (pur. 96%)

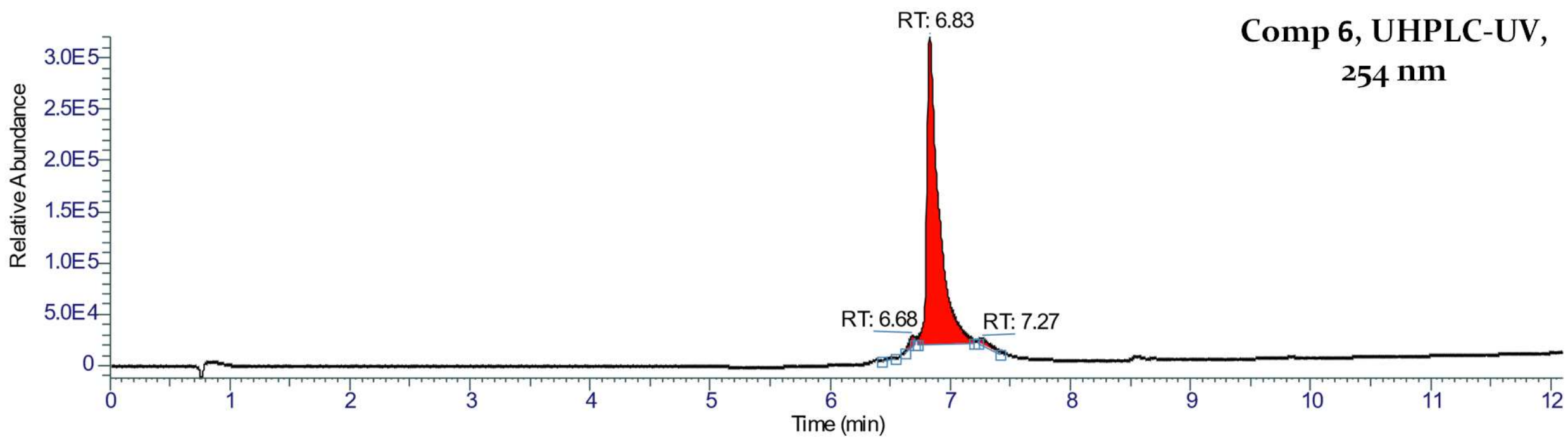

LR HPLC-MS BPC chromatogram of comp. 6, +ESI, m/z 200–2000

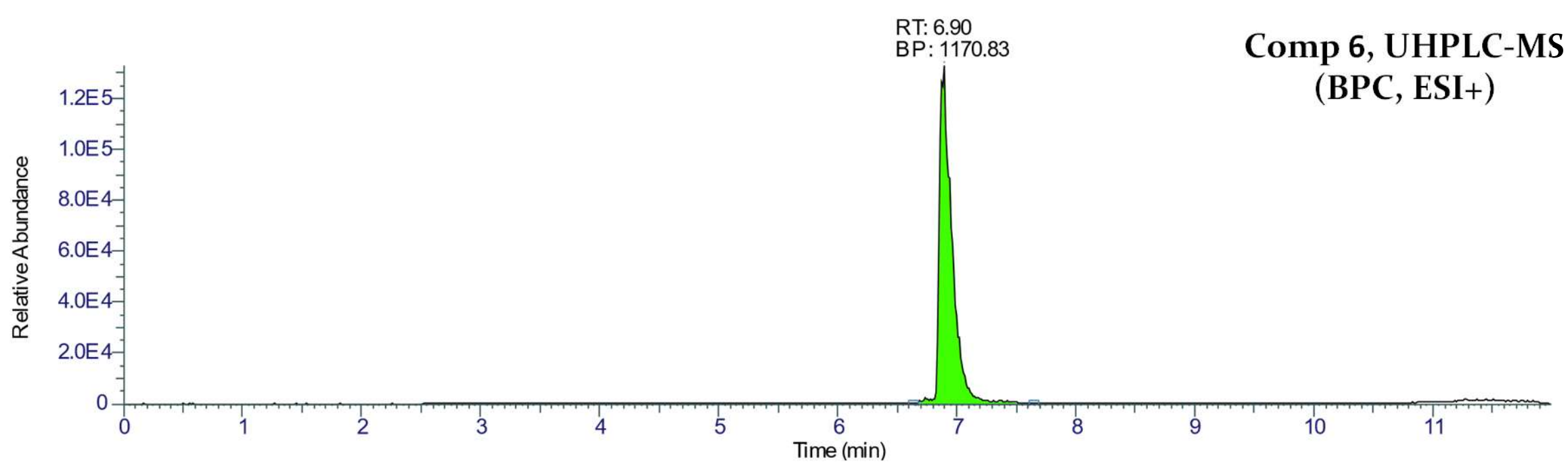

LR HPLC-MS BP spectrum of comp. 6, +ESI, m/z 200–2000

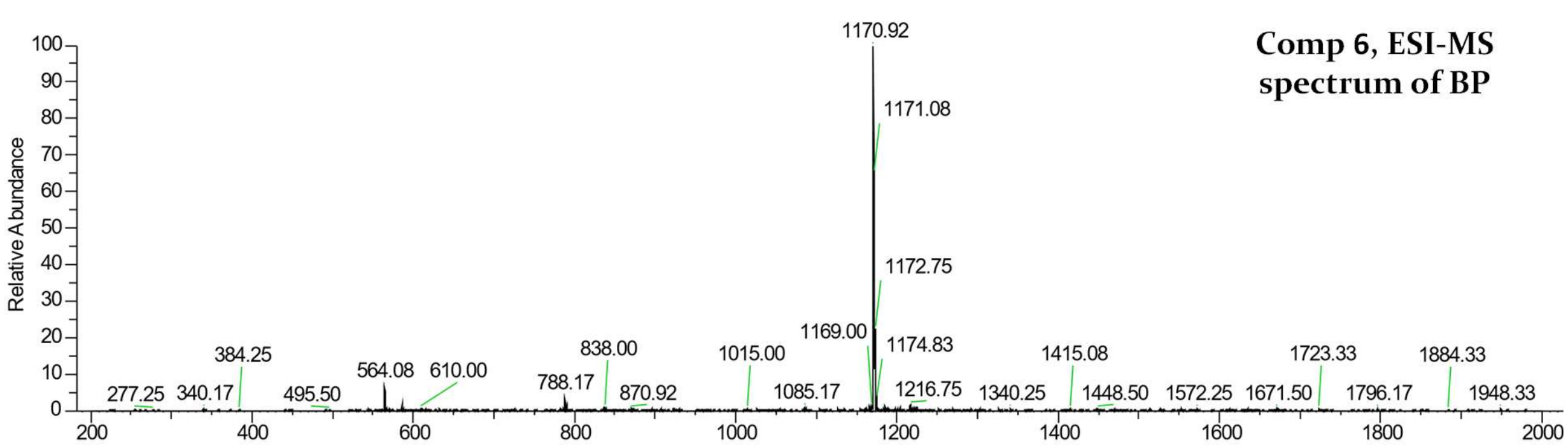

Comp. 6, HRMS (ESI): m/z $[M]^+$ calcd for C53H55N8O21S+ 1171.3197, found 1171.3205

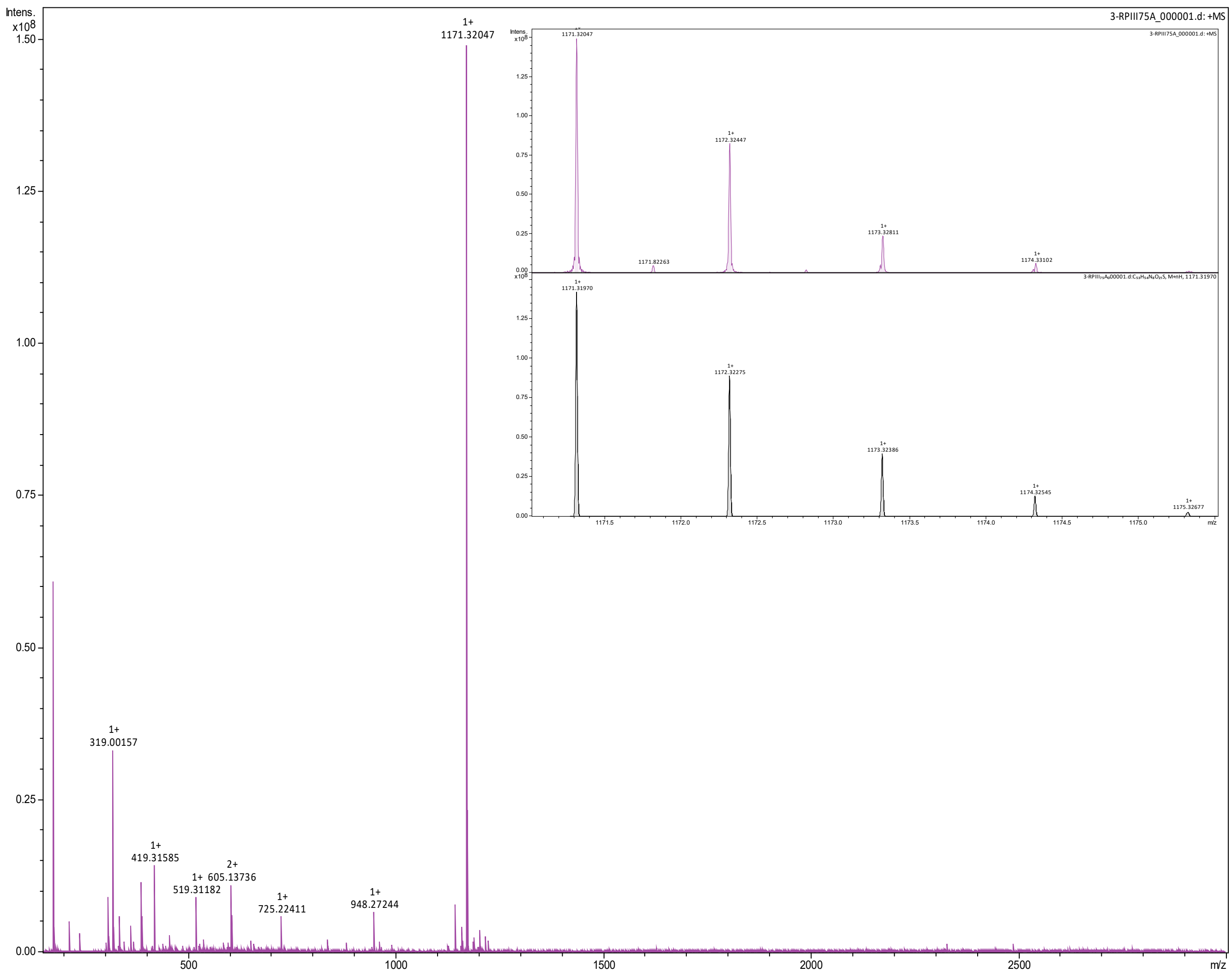
3-RPIII75A_000001.d: +MS
Intens.
x10^8
1+
1171.32047
1+
319.00157
1+
419.31585
1+ 519.31182
2+ 605.13736
1+
725.22411
1+
948.27244
m/z
1+
1172.32447
1+
1173.32811
1+
1174.33102
1171.82263
3-RPIII₇₅A₀00001.d:C₅₃H₅₄N₈O₂₁S, M+nH, 1171.31970
1+
1171.31970
1+
1172.32275
1+
1173.32386
1+
1174.32545
1+
1175.32677

Comp. 7

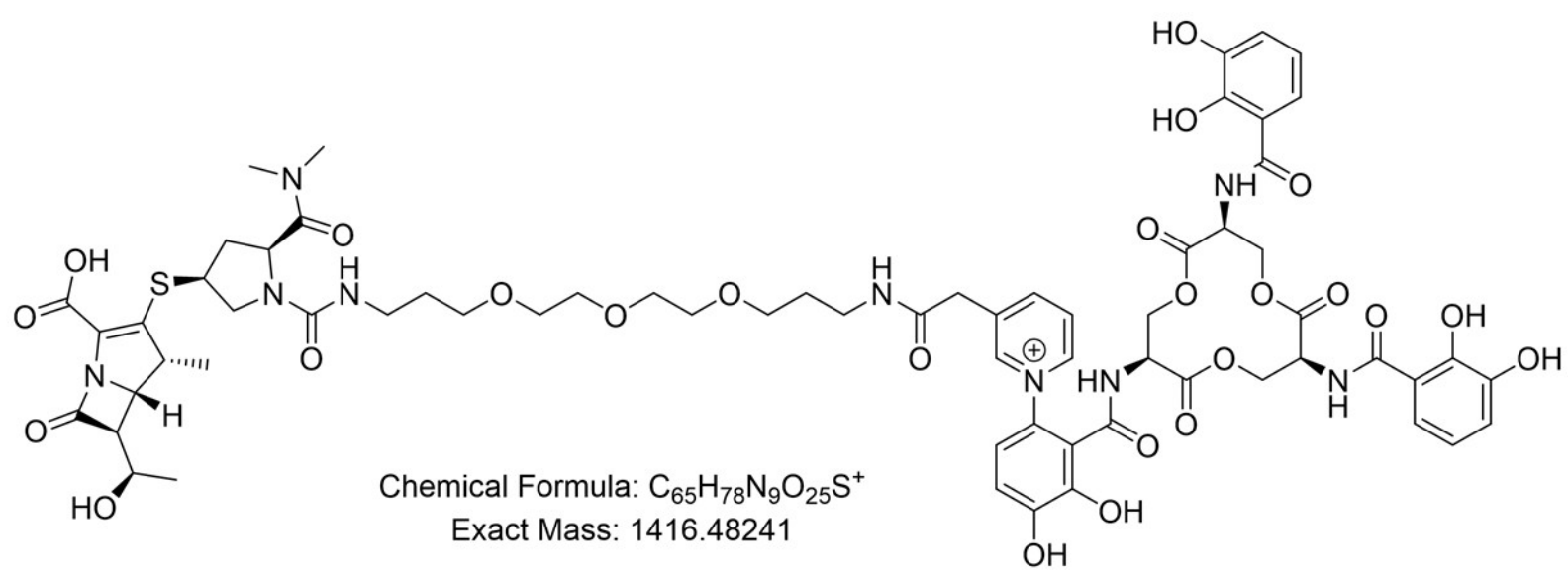

LR HPLC-MS chromatogram of comp. 7, UV 254 nm, C_{18}, 0–100% H_2O/MeCN+0.1% FA (linear gradient, 0-12 min), +ESI (pur. 95%)

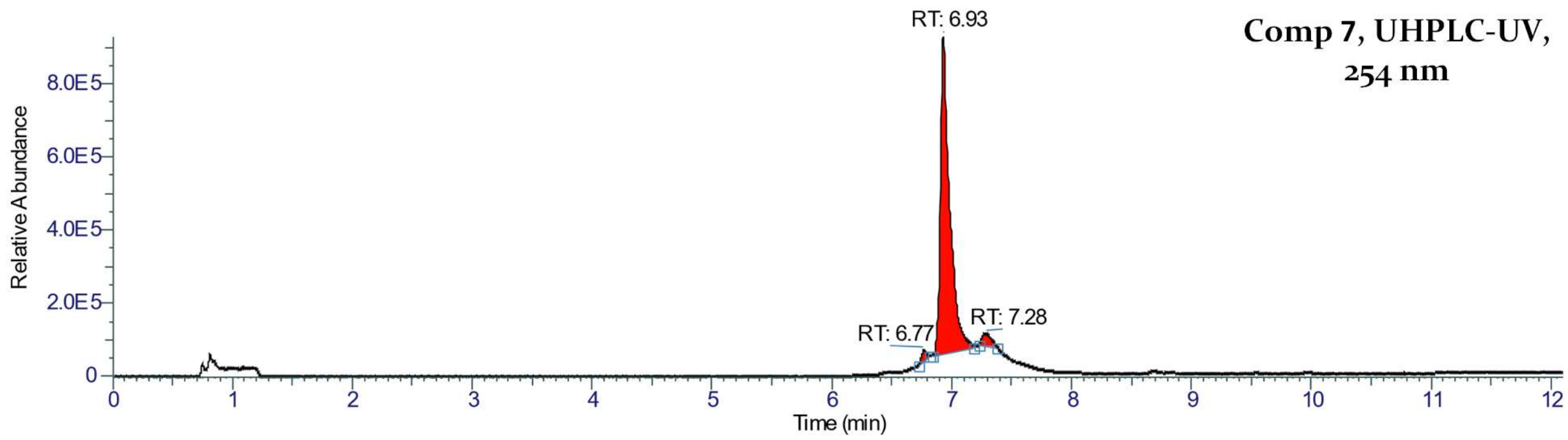

LR HPLC-MS BPC chromatogram of comp. 7, +ESI, m/z 200–2000

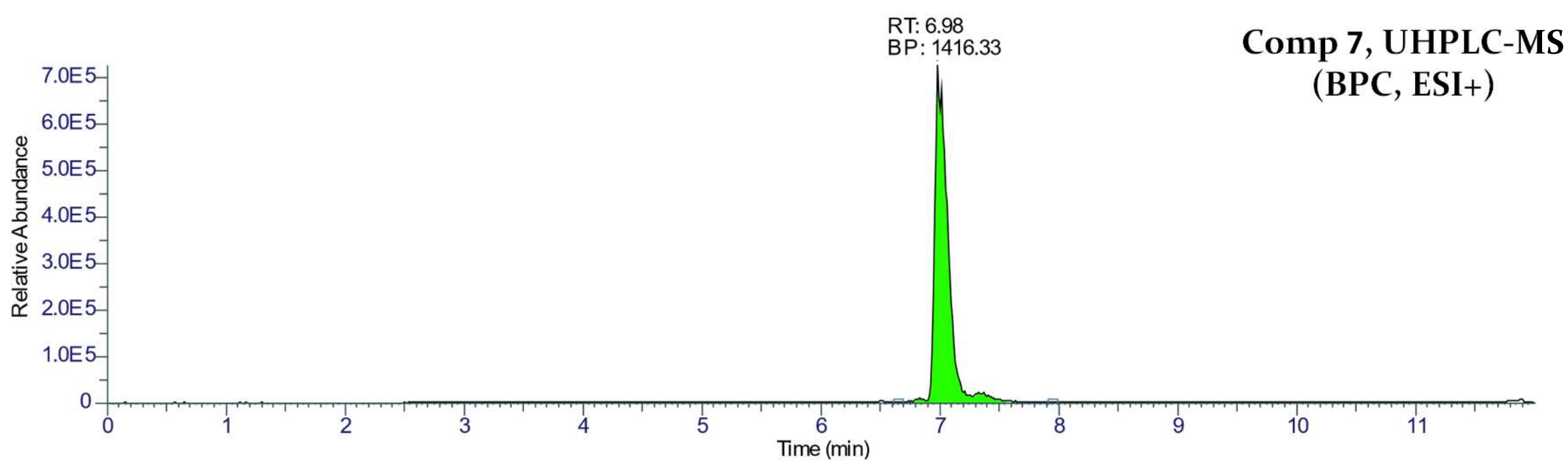

LR HPLC-MS BP spectrum of comp. 7, +ESI, m/z 200–2000

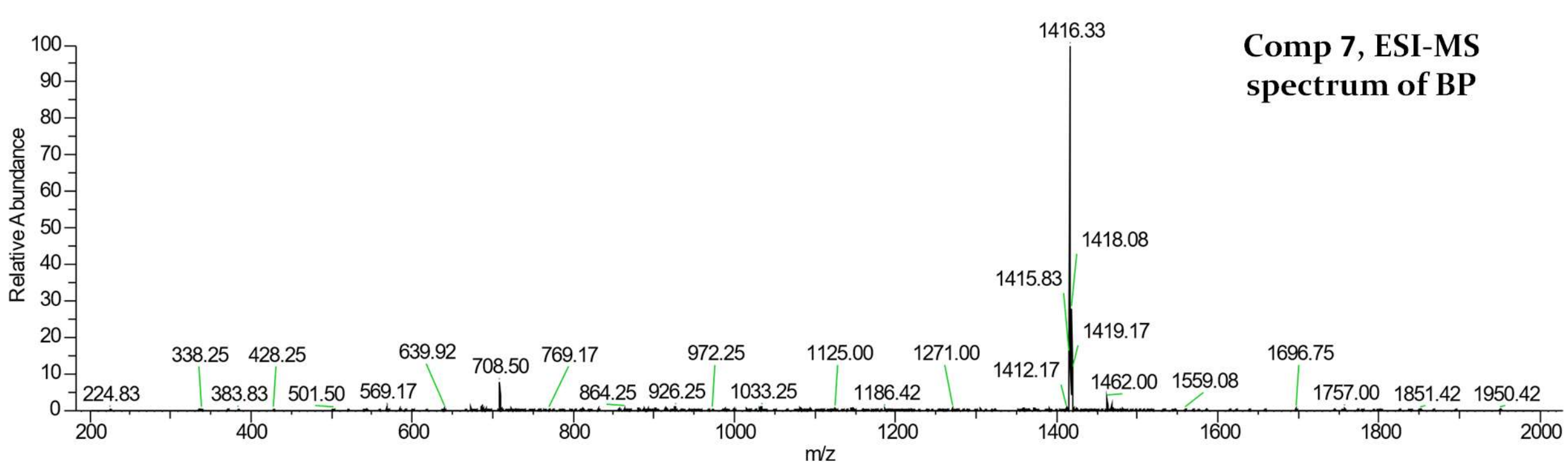

Comp. 7, HRMS (ESI): *m/z* [M]$^+$ calcd for $C_{65}H_{78}N_9O_{25}S^+$ 1416.4830, found 1416.4878

Elemental Composition Report

Page 1

Single Mass Analysis

Tolerance = 50.0 mDa / DBE: min = -1.5, max = 50.0
Element prediction: Off
Number of isotope peaks used for i-FIT = 3

Monoisotopic Mass, Even Electron Ions
317 formula(e) evaluated with 16 results within limits (up to 1 best isotopic matches for each mass)
Elements Used:
C: 50-70 H: 50-80 N: 3-10 O: 5-27 S: 0-1

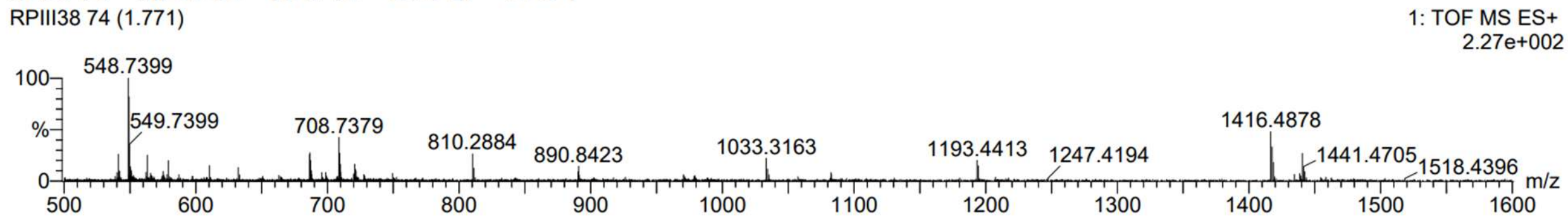

Minimum:				-1.5		
Maximum:		50.0	10.0	50.0		
Mass	Calc. Mass	mDa	PPM	DBE	i-FIT	Formula
1416.4878	1416.4830	4.8	3.4	31.5	0.4	C65 H78 N9 O25 S

Comp. 7b

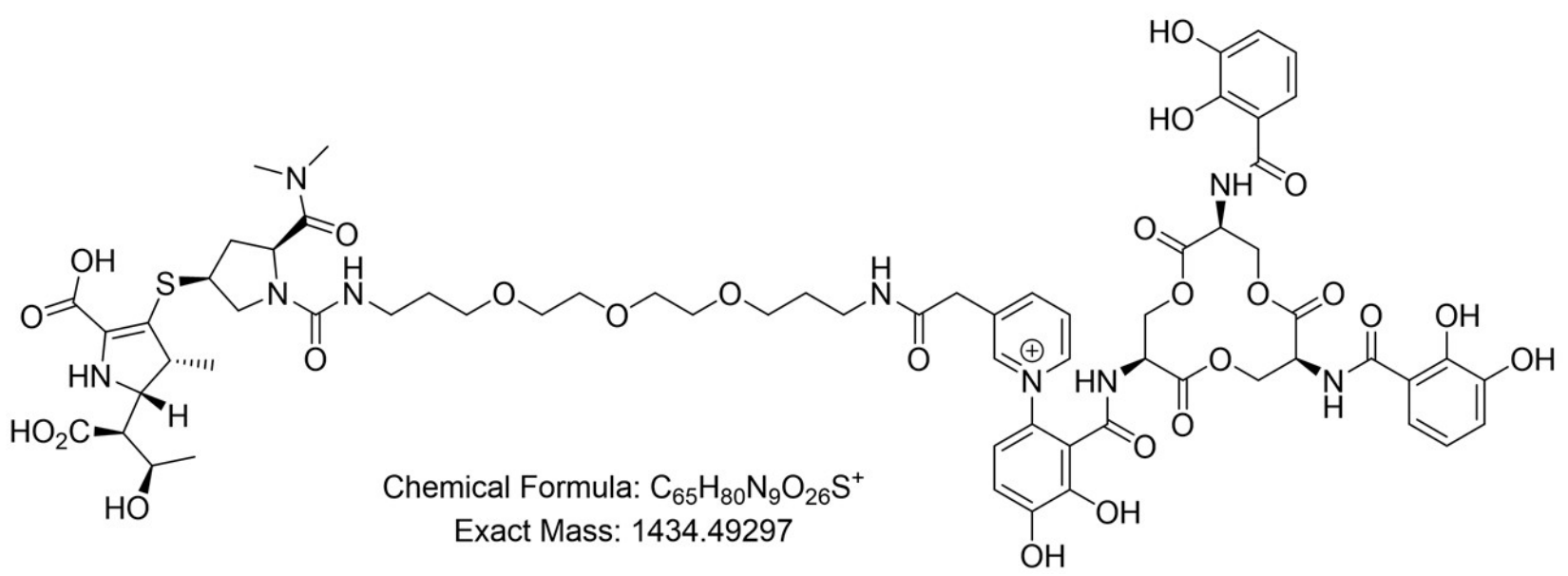

LR HPLC-MS chromatogram of comp. 7b, UV 254 nm, C_{18}, 0–100% H_2O/MeCN+0.1% FA (linear gradient, 0-12 min), +ESI (pur. 99%)

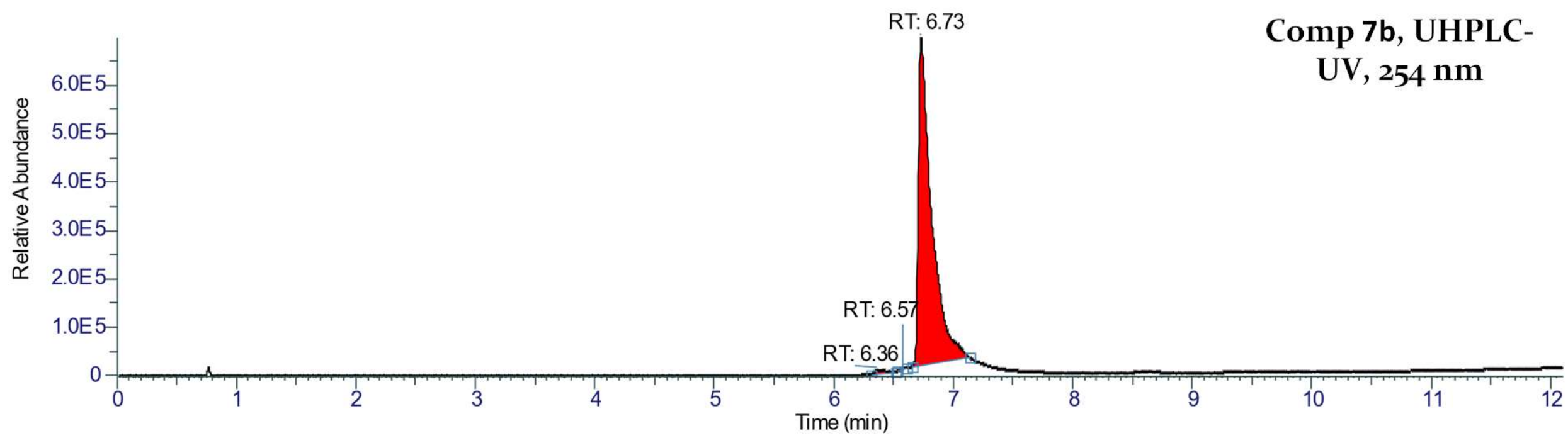

LR HPLC-MS BPC chromatogram of comp. 7b, +ESI, m/z 200–2000

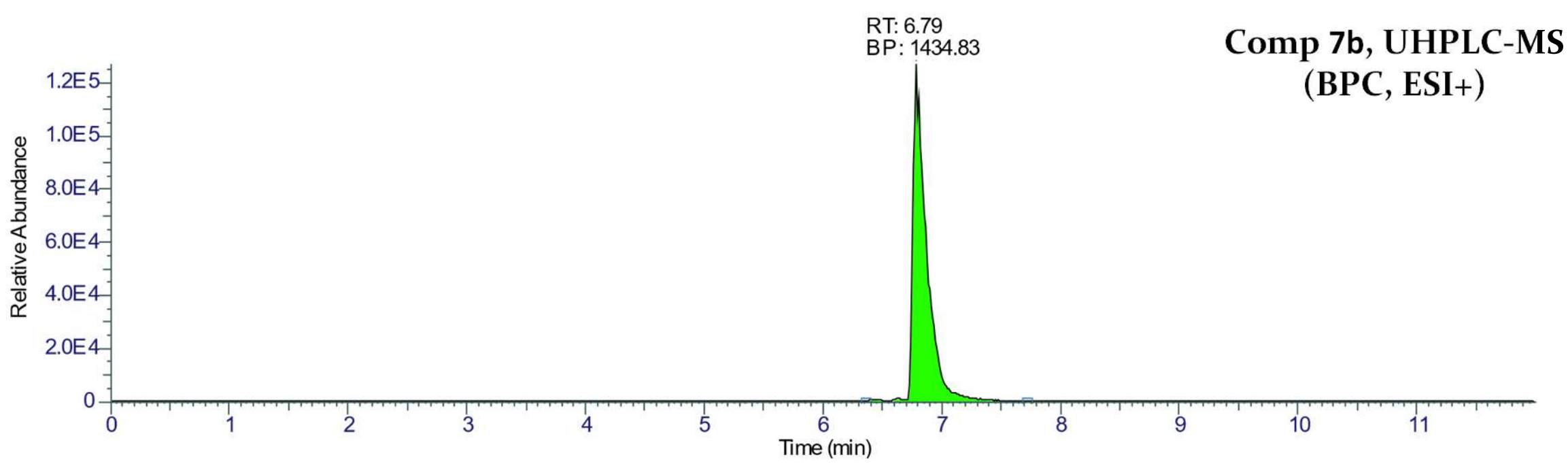

LR HPLC-MS BP spectrum of comp. 7b, +ESI, m/z 200–2000

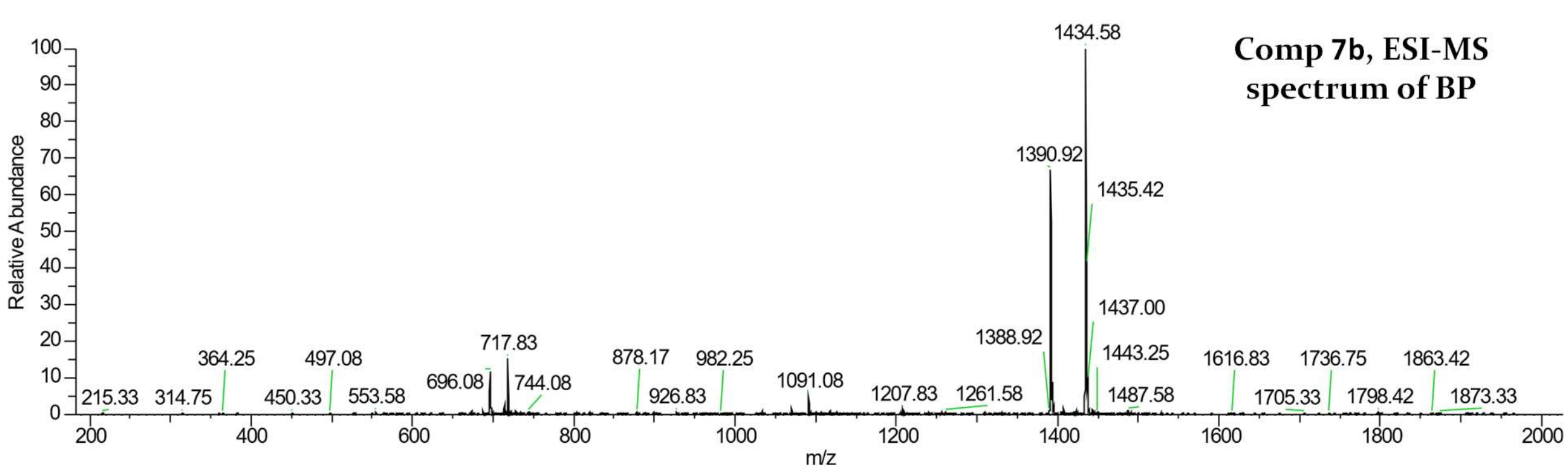

Comp. 8

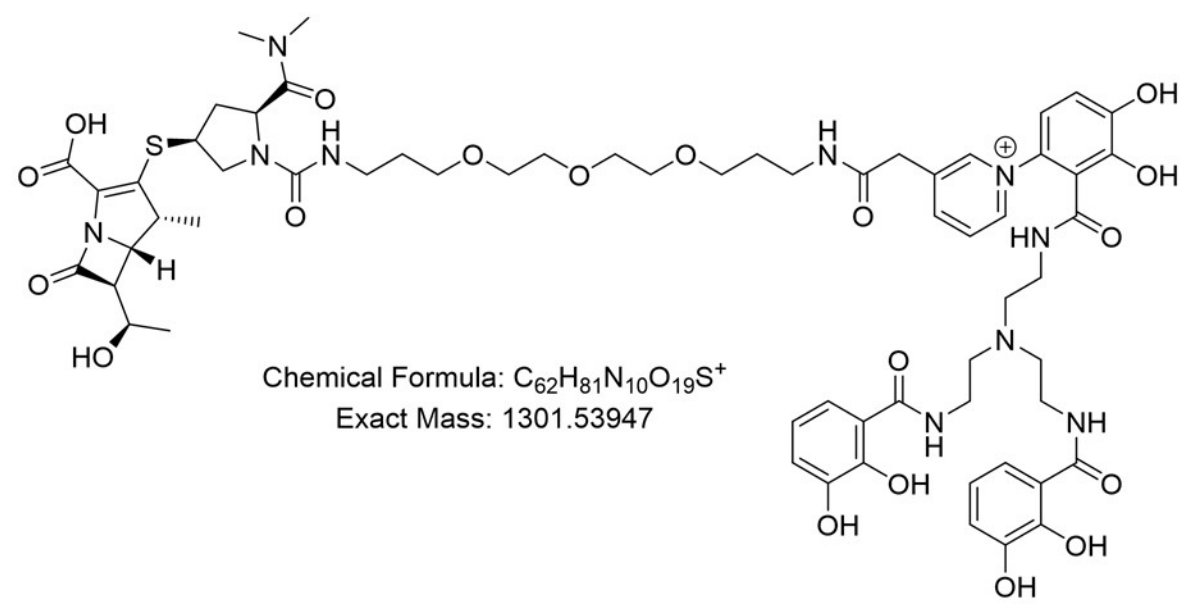

LR HPLC-MS chromatogram of comp. 8, UV 254 nm, C_{18}, 0–100% H_2O/MeCN+0.1% FA (linear gradient, 0-12 min), +ESI (pur. 98%)

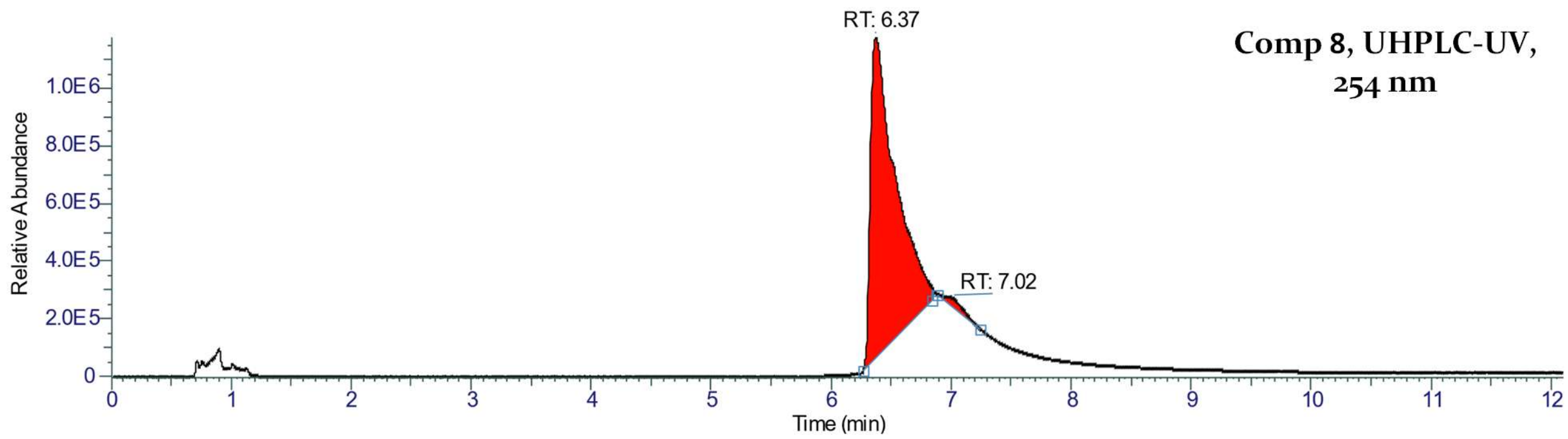

LR HPLC-MS BPC chromatogram of comp. 8, +ESI, m/z 200–2000

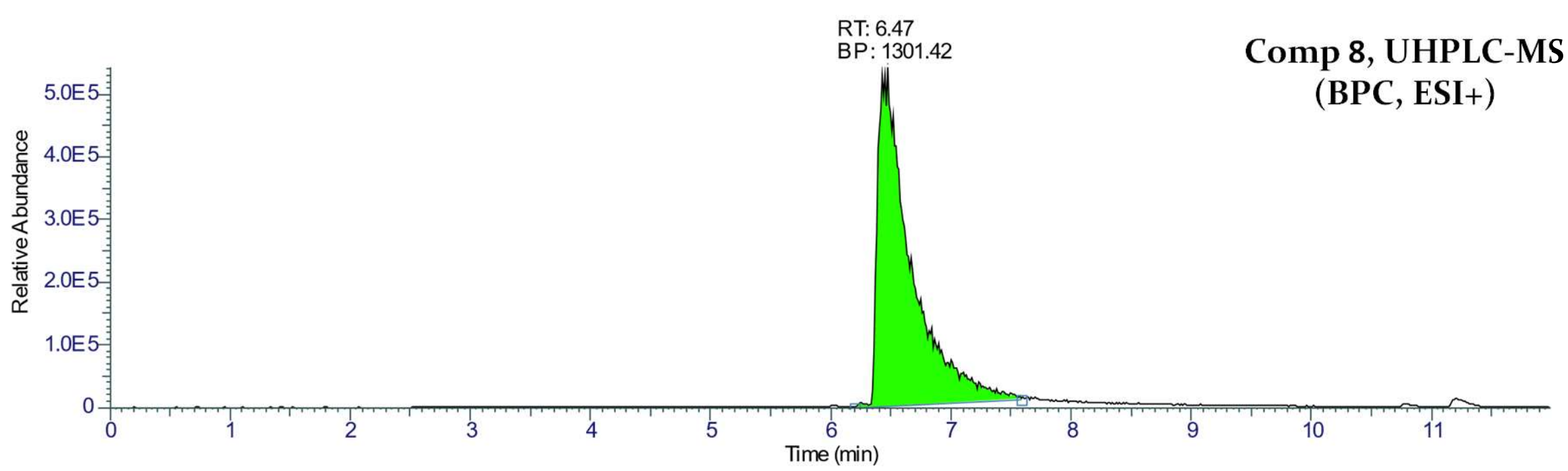

LR HPLC-MS BP spectrum of comp. 8, +ESI, m/z 200–2000

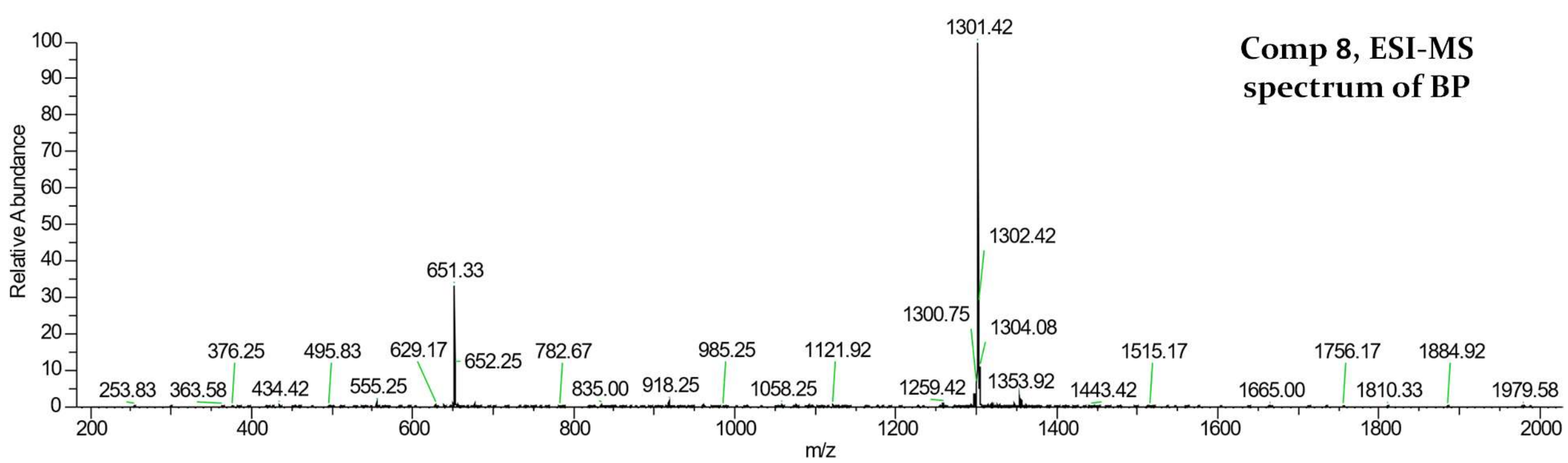

Comp. 8, HRMS (ESI): *m/z* [M]$^+$ calcd for $C_{62}H_{81}N_{10}O_{19}S^+$ calculated 1301.5395, found 1301.5378

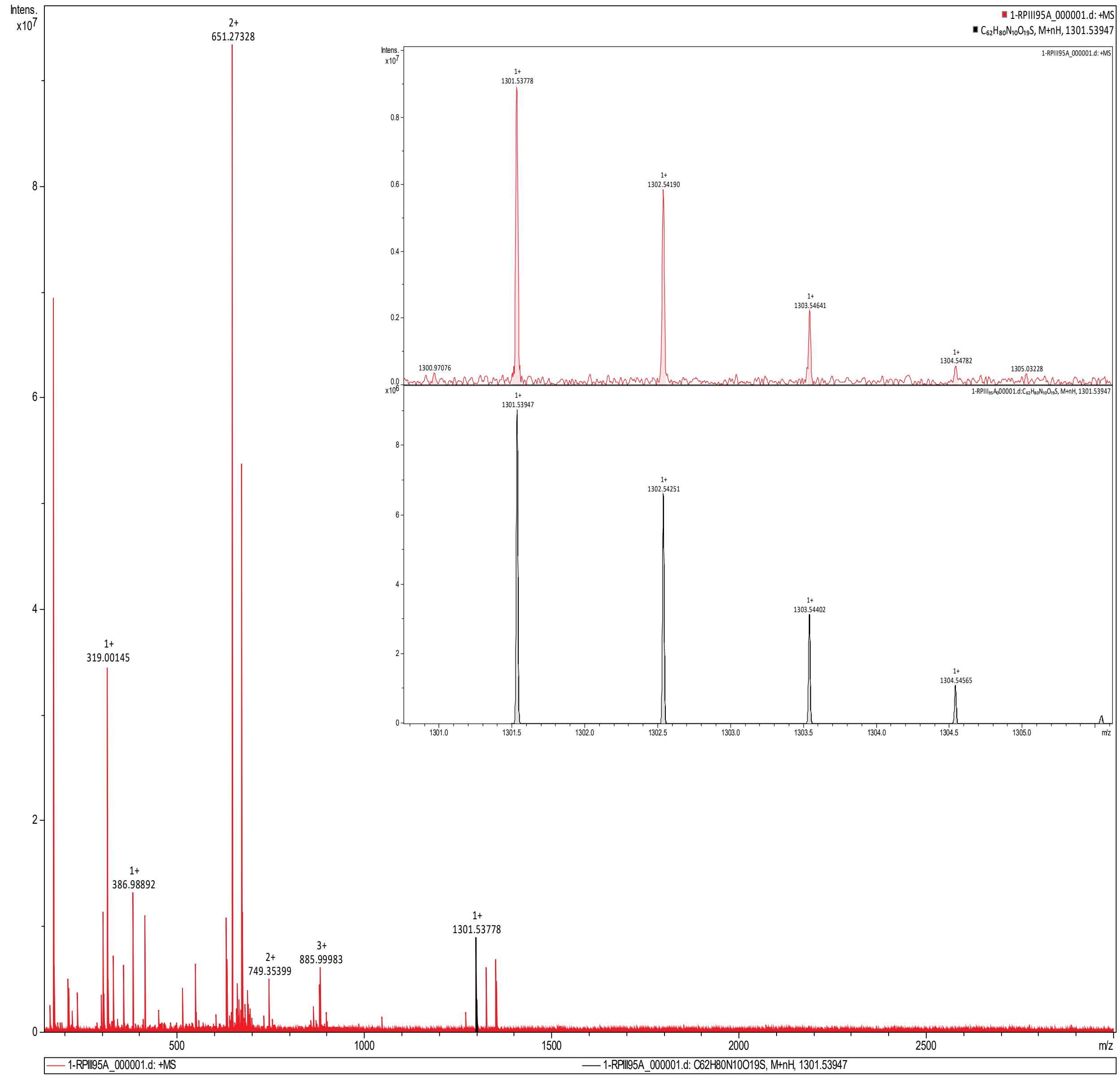

Comp. 9

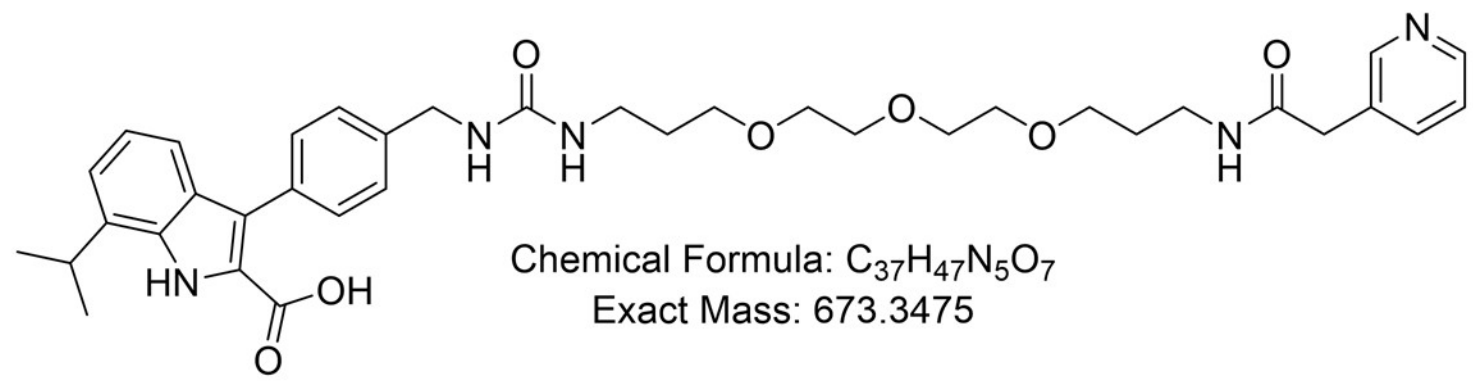

LR HPLC-MS chromatogram of comp. 9, UV 254 nm, C_{18}, 0–100% H_2O/MeCN+0.1% FA (linear gradient, 0-12 min), +ESI (pur. 97%)

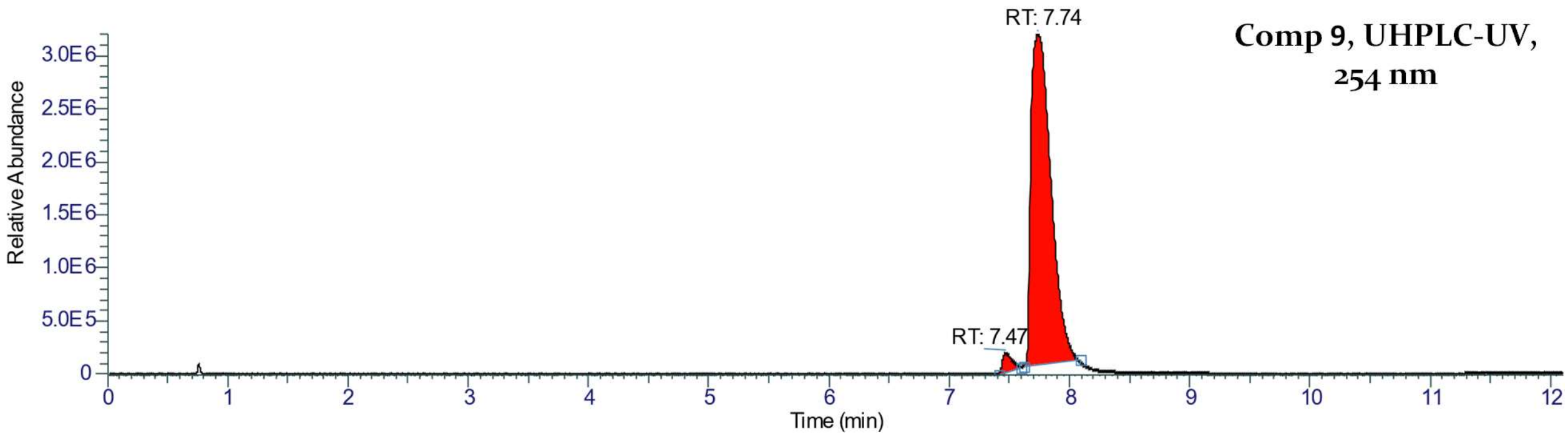

LR HPLC-MS BPC chromatogram of comp. 9, +ESI, m/z 200–2000

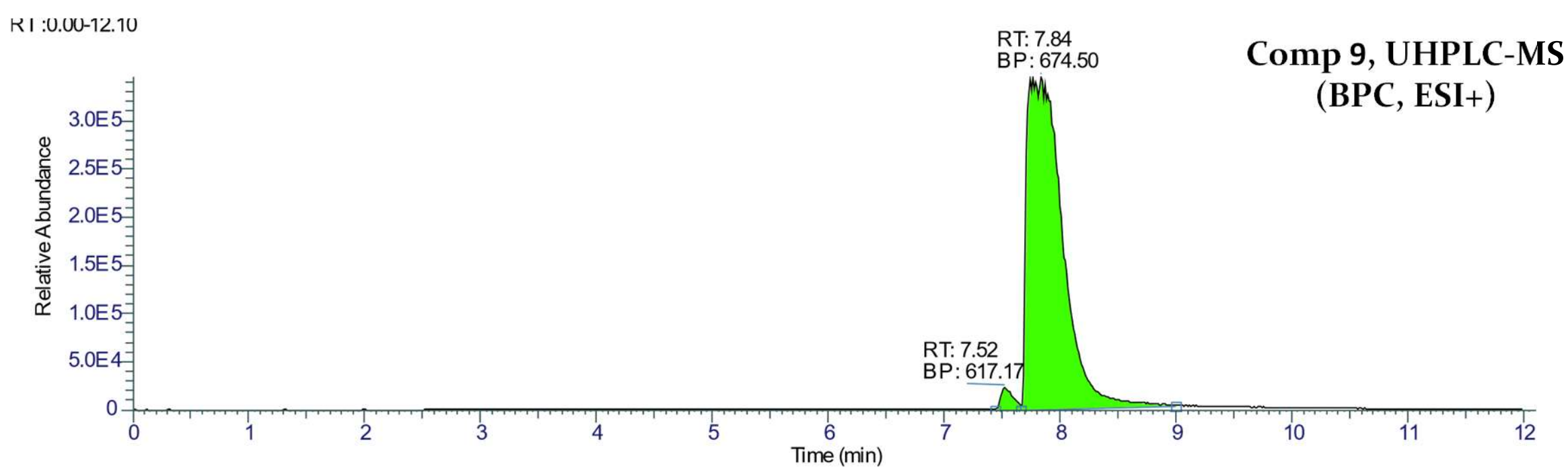

LR HPLC-MS BP spectrum of comp. 9, +ESI, m/z 200–2000

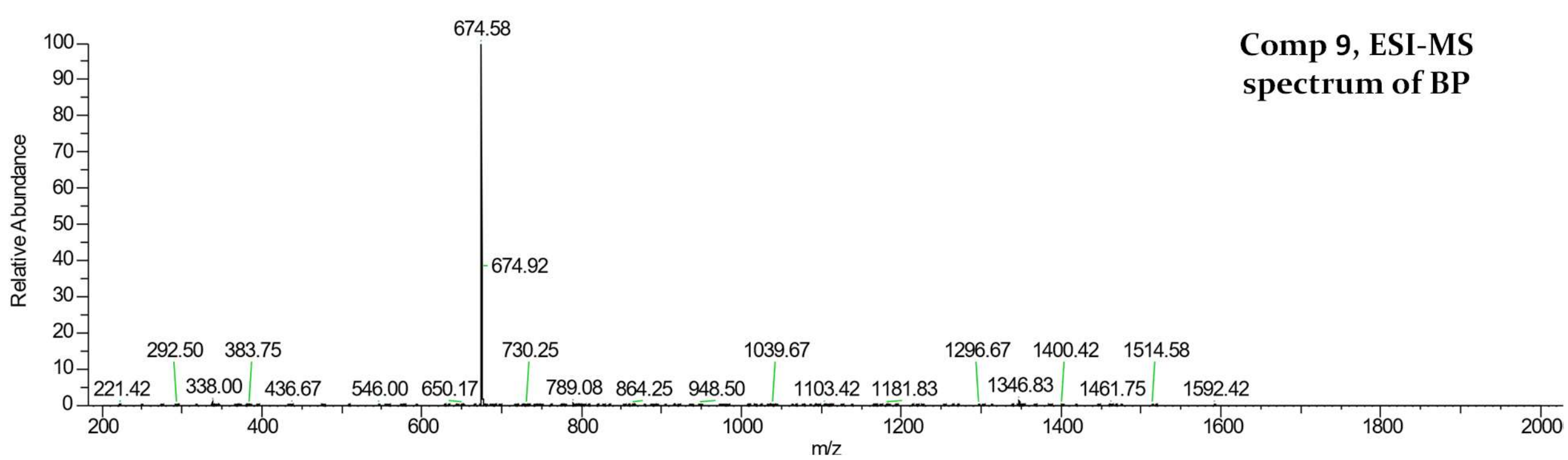

Comp. 10

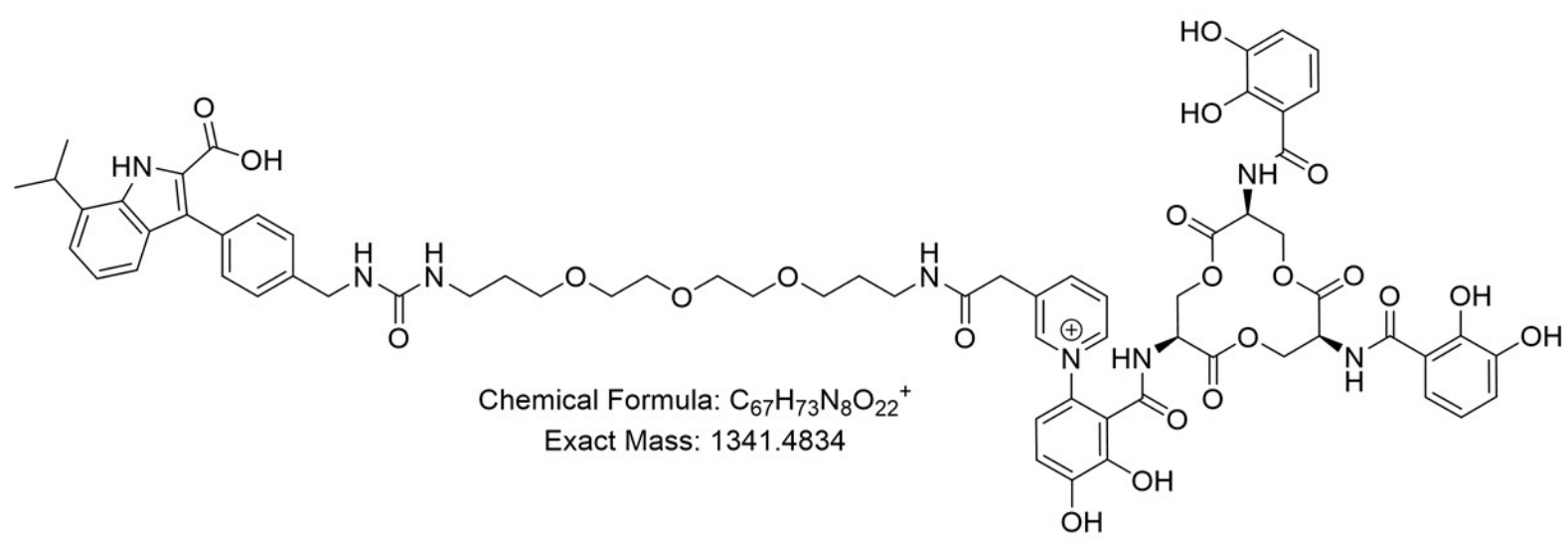

LR HPLC-MS chromatogram of comp. 11, UV 254 nm, C_{18}, 0–100% H_2O/MeCN+0.1% FA (linear gradient, 0-12 min), +ESI (pur. 96%)

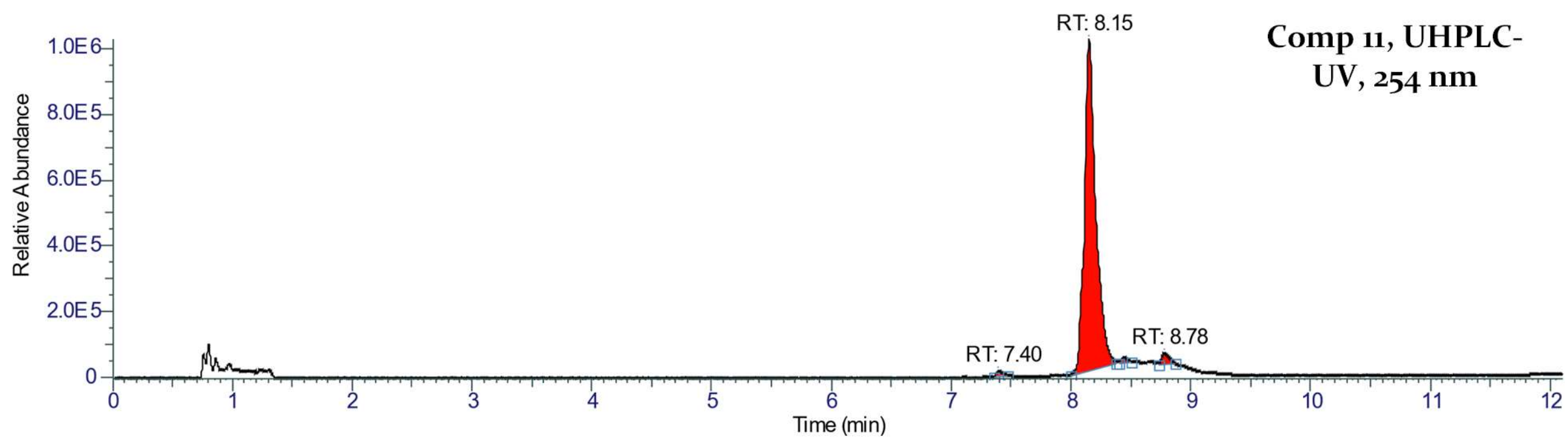

LR HPLC-MS BPC chromatogram of comp. 11, +ESI, m/z 200–2000

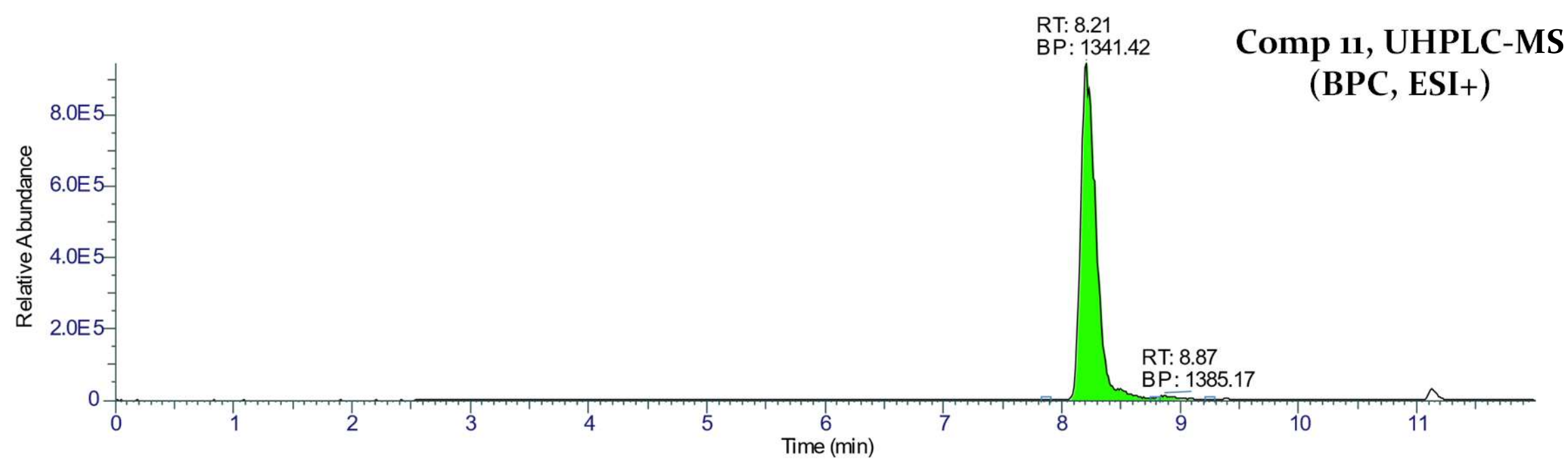

LR HPLC-MS BP spectrum of comp. 11, +ESI, m/z 200–2000

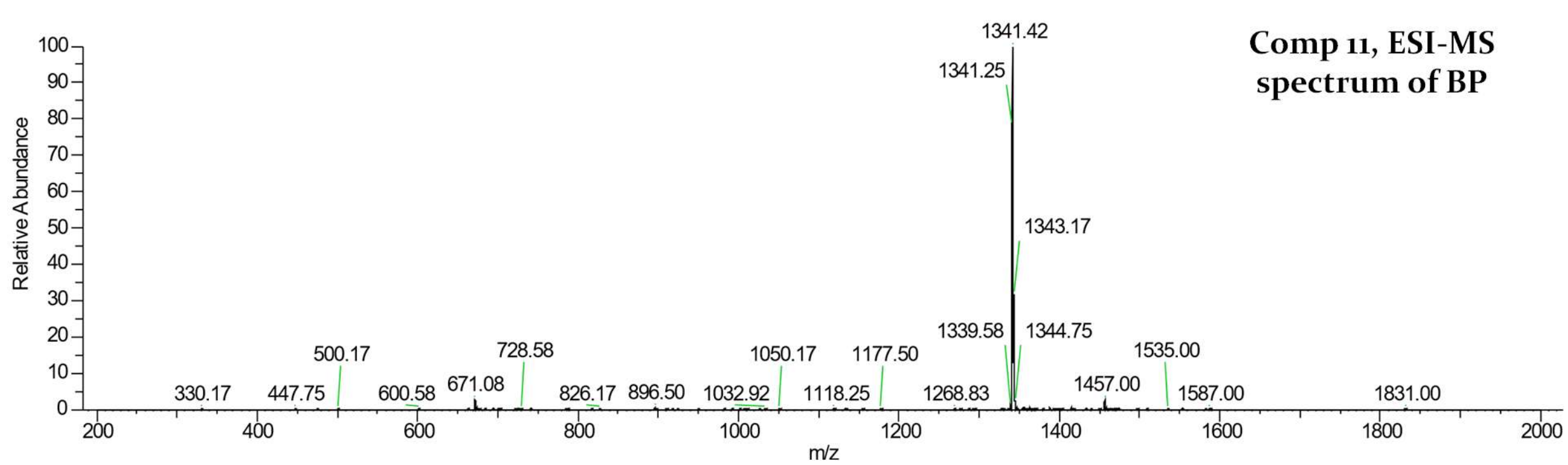

Comp. 11, HRMS (ESI): *m/z* [M]$^+$ calcd for $C_{67}H_{73}N_8O_{22}^+$ 1341.4839, found 1341.4832

Elemental Composition Report

Page 1

Single Mass Analysis

Tolerance = 50.0 mDa / DBE: min = -1.5, max = 50.0
Element prediction: Off
Number of isotope peaks used for i-FIT = 3

Monoisotopic Mass, Even Electron Ions
13 formula(e) evaluated with 6 results within limits (up to 1 closest results for each mass)
Elements Used:
C: 60-70 H: 68-78 N: 7-9 O: 20-25

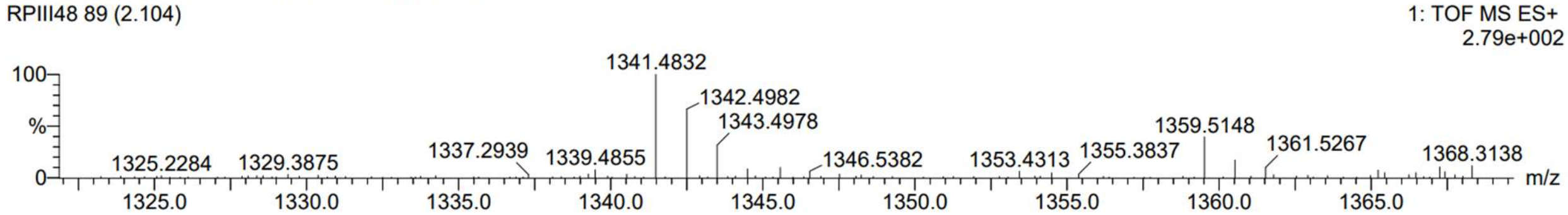

Minimum:				-1.5		
Maximum:		50.0	10.0	50.0		
Mass	Calc. Mass	mDa	PPM	DBE	i-FIT	Formula
1341.4832	1341.4839	-0.7	-0.5	35.5	2.0	C67 H73 N8 O22

Comp. 10

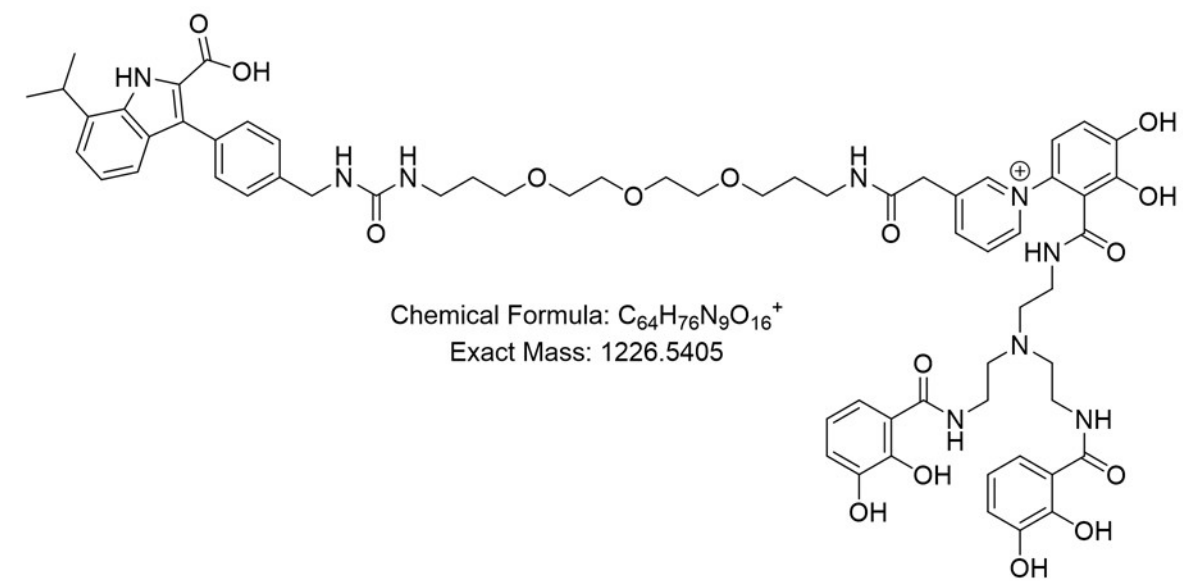

LR HPLC-MS chromatogram of comp. 10, UV 254 nm, C_{18}, 0–100% H_2O/MeCN+0.1% FA (linear gradient, 0-12 min), +ESI (pur. 99%)

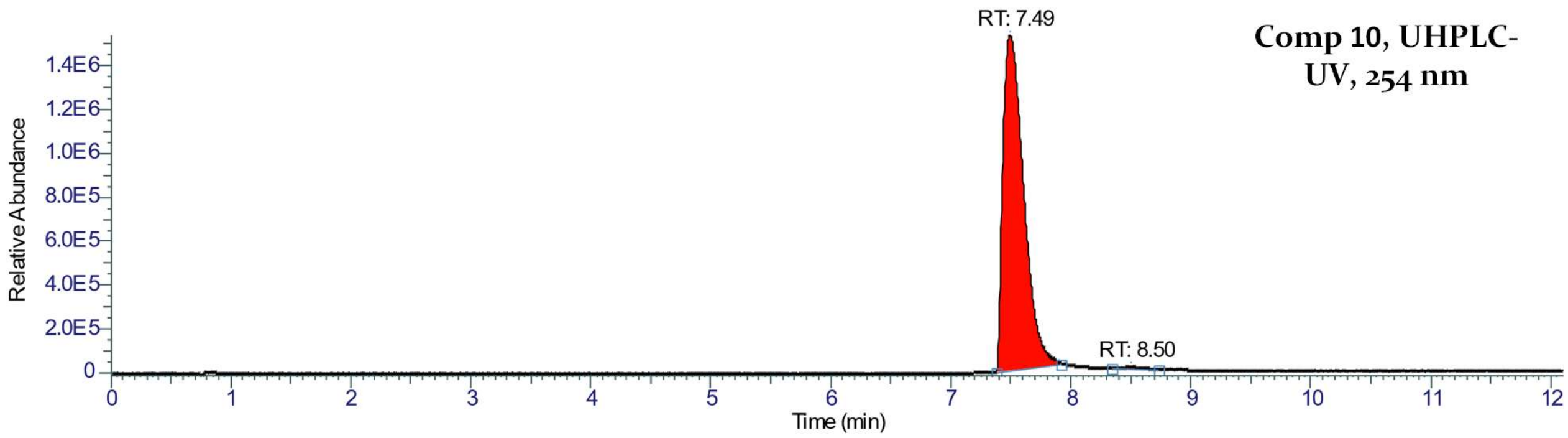

LR HPLC-MS BPC chromatogram of comp. 10, +ESI, m/z 200–2000

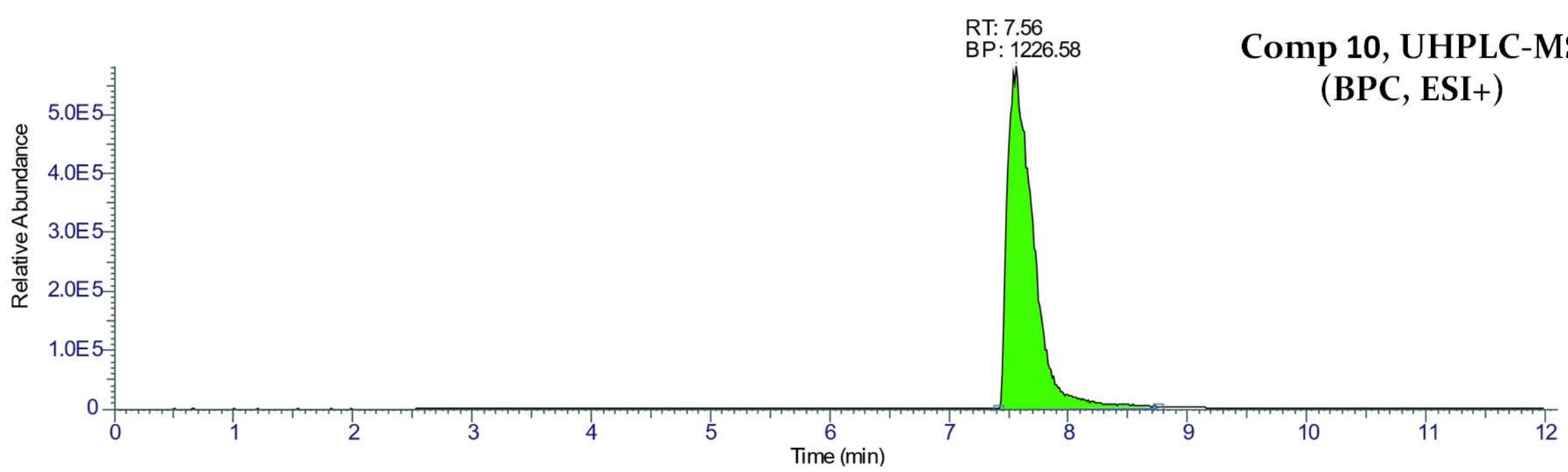

LR HPLC-MS BP spectrum of comp. 10, +ESI, m/z 200–2000

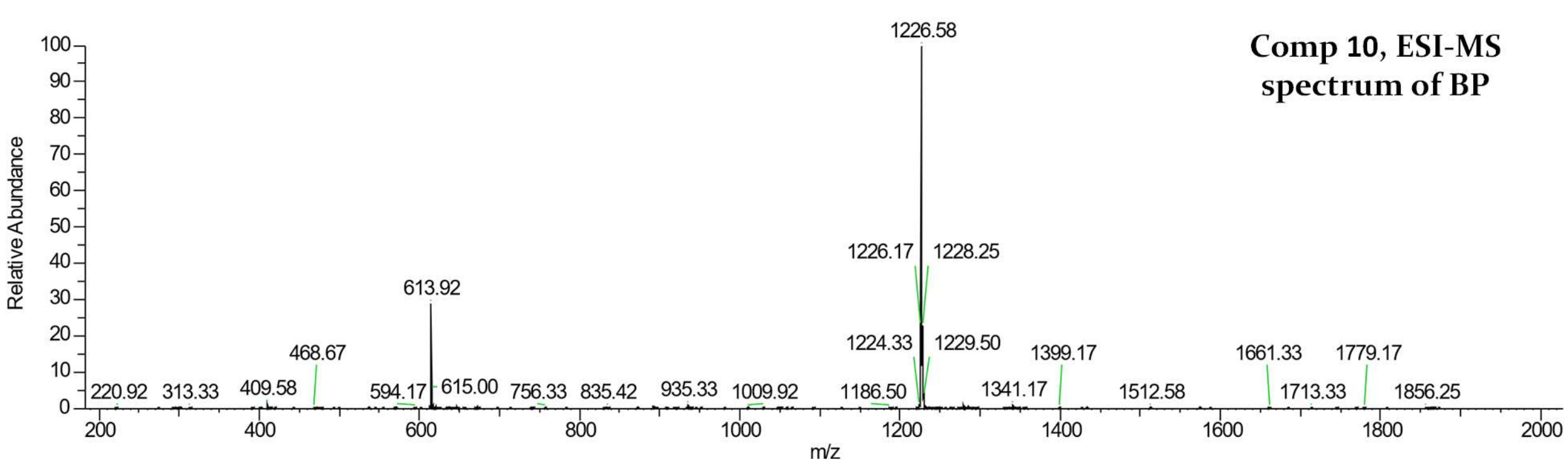

Comp. 10, HRMS (ESI): ***m/z*** **[M]$^+$ calcd for $C_{64}H_{76}N_9O_{16}^+$ 1226.5405, found 1226.5405**

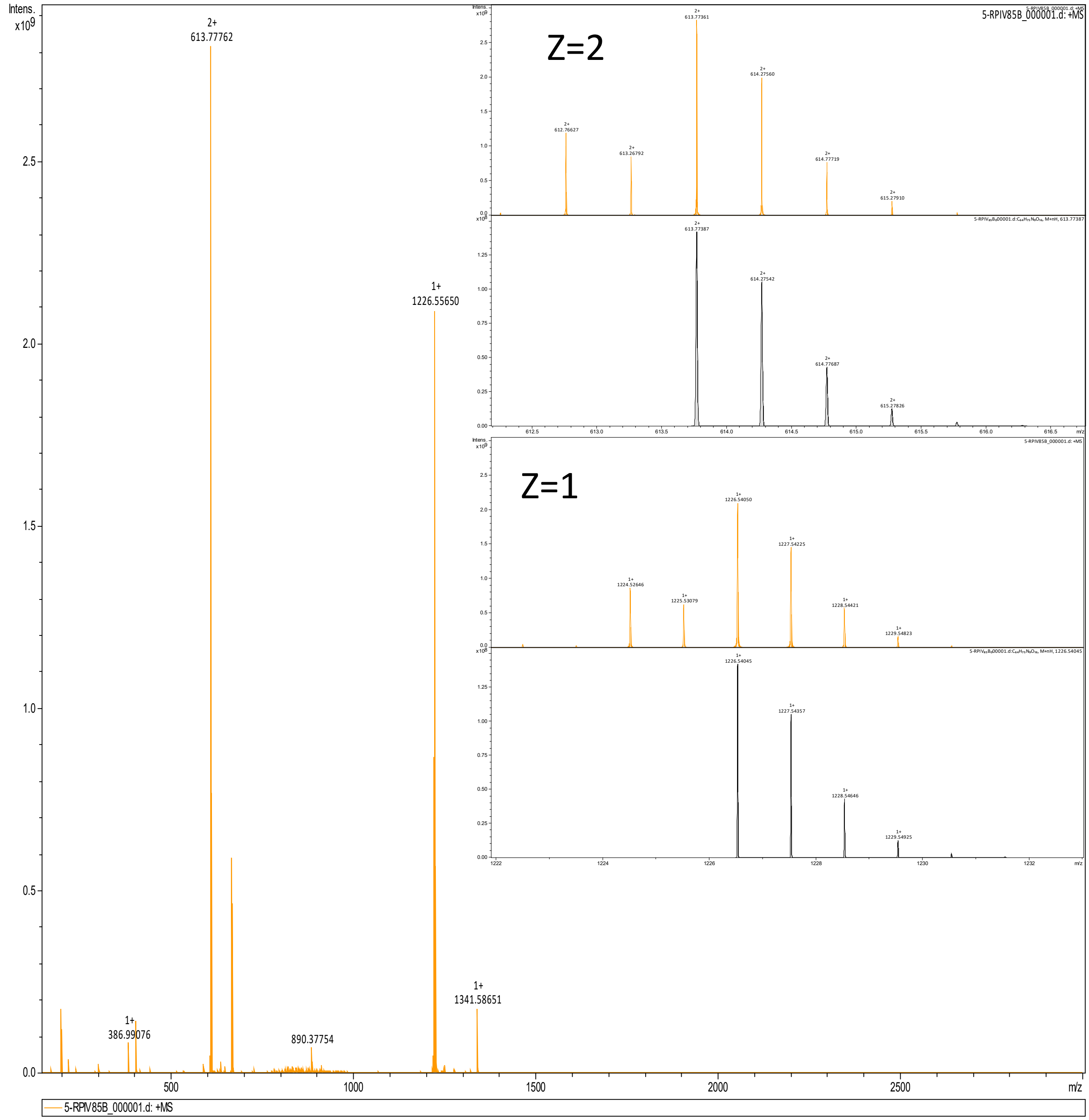

Comp. 21

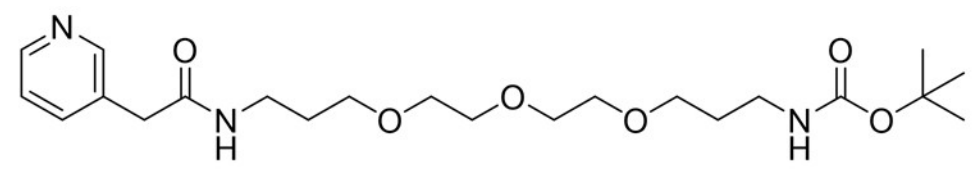

Chemical Formula: $C_{22}H_{37}N_3O_6$
Exact Mass: 439.2682

LR HPLC-MS chromatogram of comp. 21, UV 254 nm, C18, 0–100% H2O/MeCN+0.1% FA (linear gradient, 0-12 min), +ESI (pur. 93%)

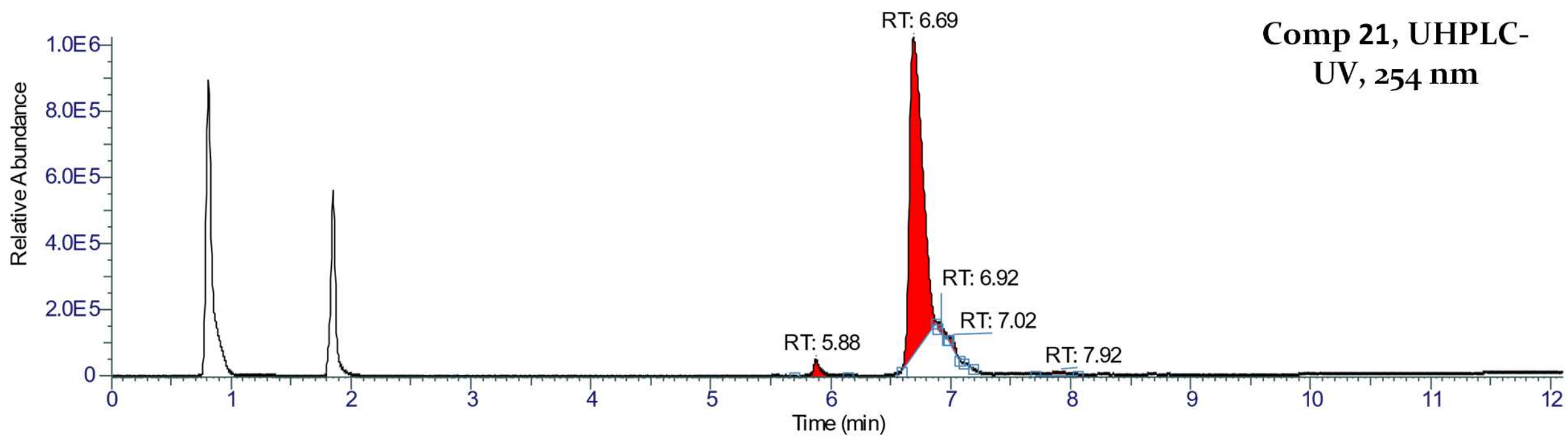

LR HPLC-MS BPC chromatogram of comp. 21, +ESI, m/z 200–2000

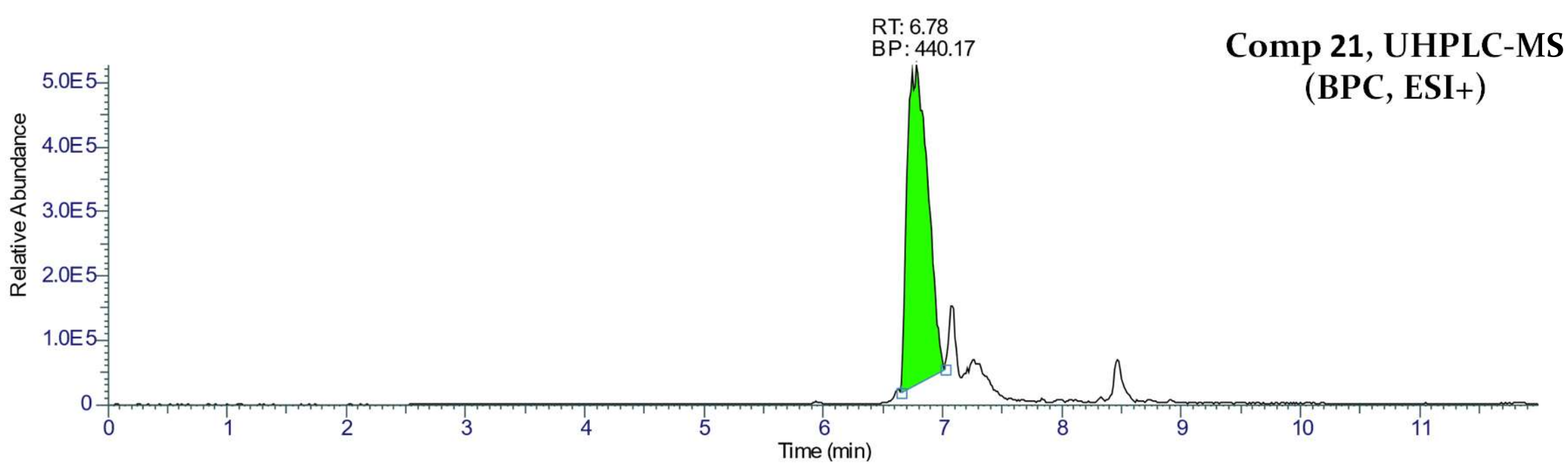

LR HPLC-MS BP spectrum of comp. 21, +ESI, m/z 200–2000

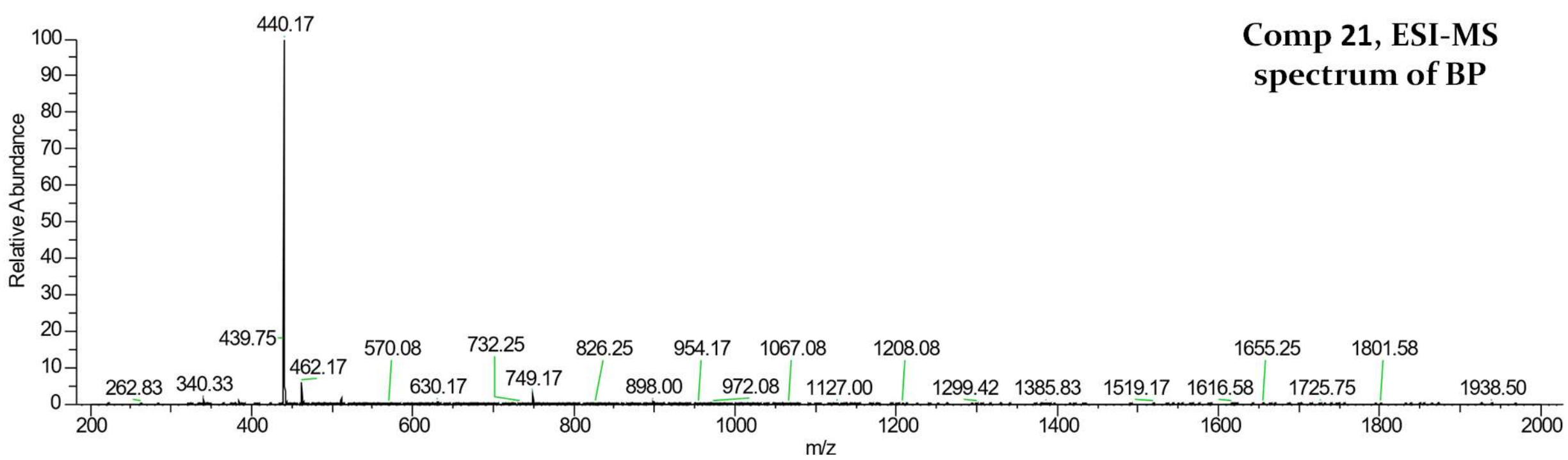

Comp. 22

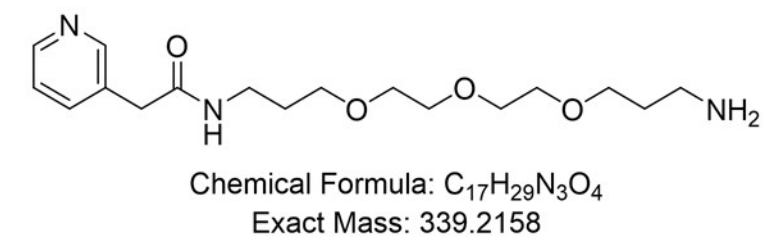

LR HPLC-MS chromatogram of comp. 22, UV 254 nm, C_{18}, 0–100% H_2O/MeCN+0.1% FA (linear gradient, 0-12 min), +ESI (pur. 75%)

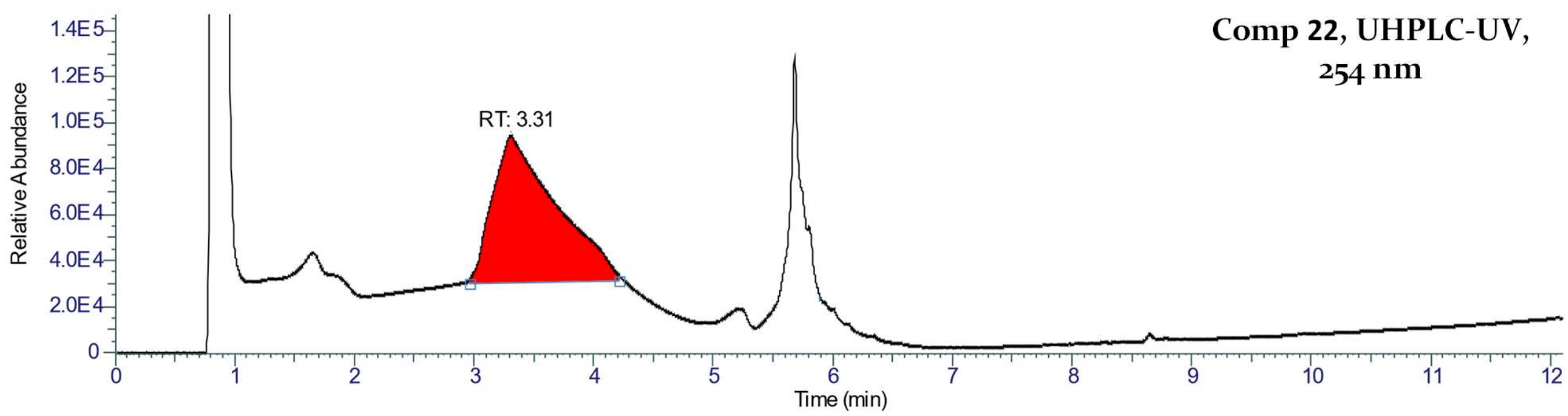

LR HPLC-MS BPC chromatogram of comp. 22, +ESI, m/z 200–2000

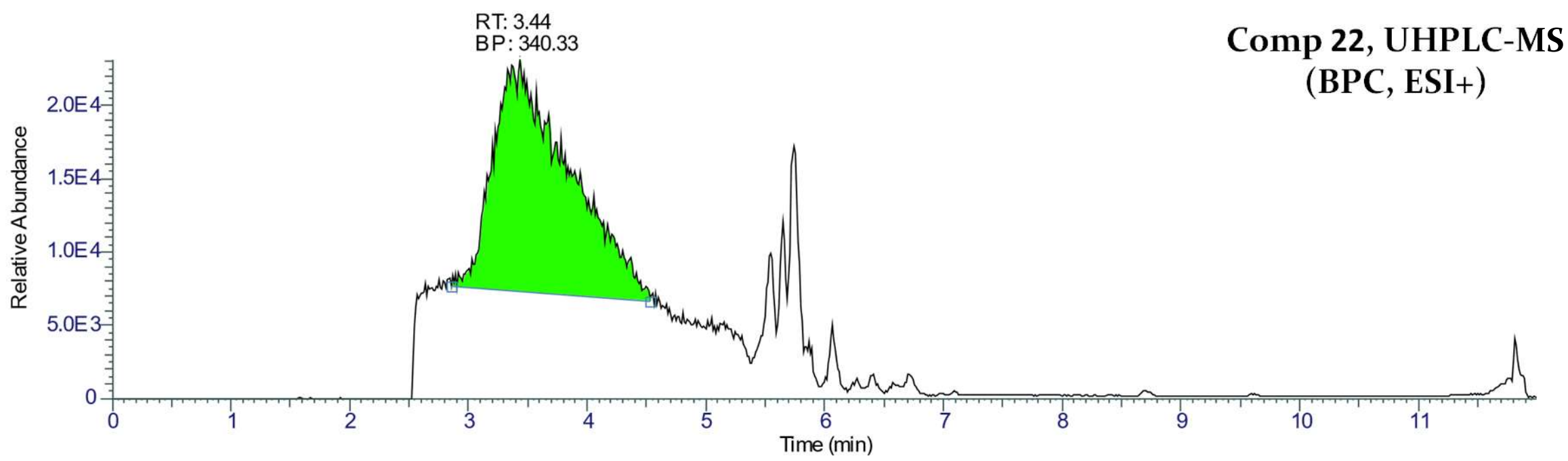

LR HPLC-MS BP spectrum of comp. 22, +ESI, m/z 200–2000

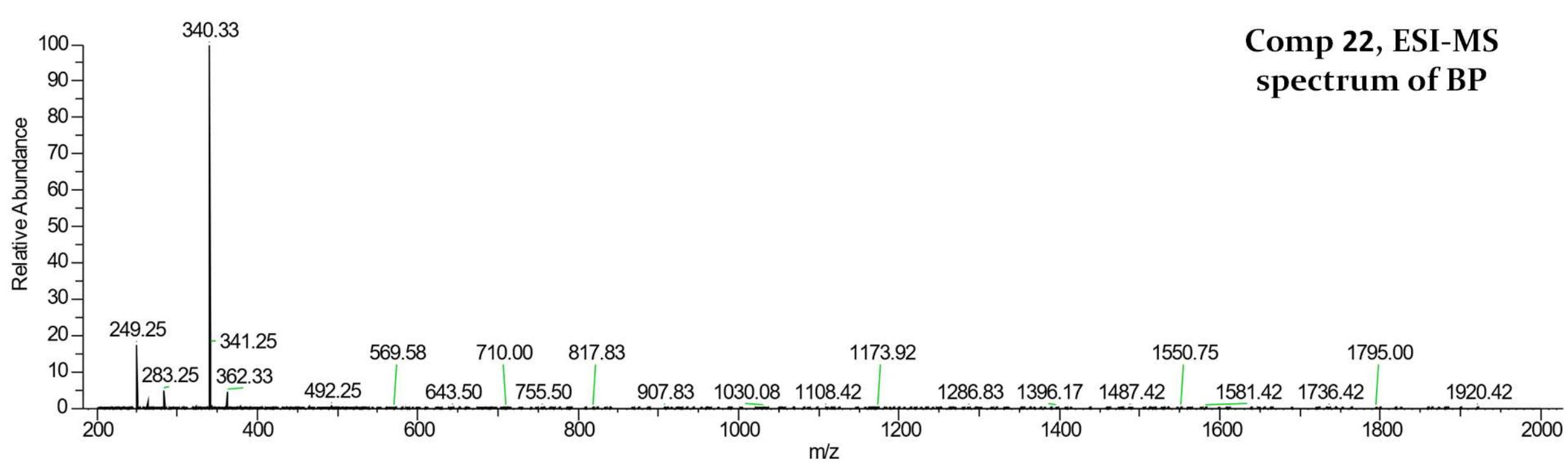

Comp. 25

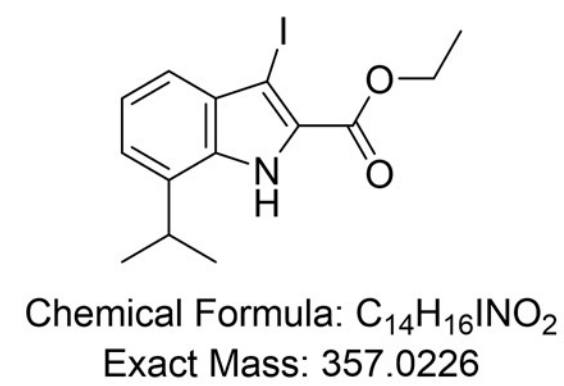

LR HPLC-MS chromatogram of comp. 25, UV 254 nm, C_{18}, 0–100% H_2O/MeCN+0.1% FA (linear gradient, 0-12 min), +ESI (pur. 93%)

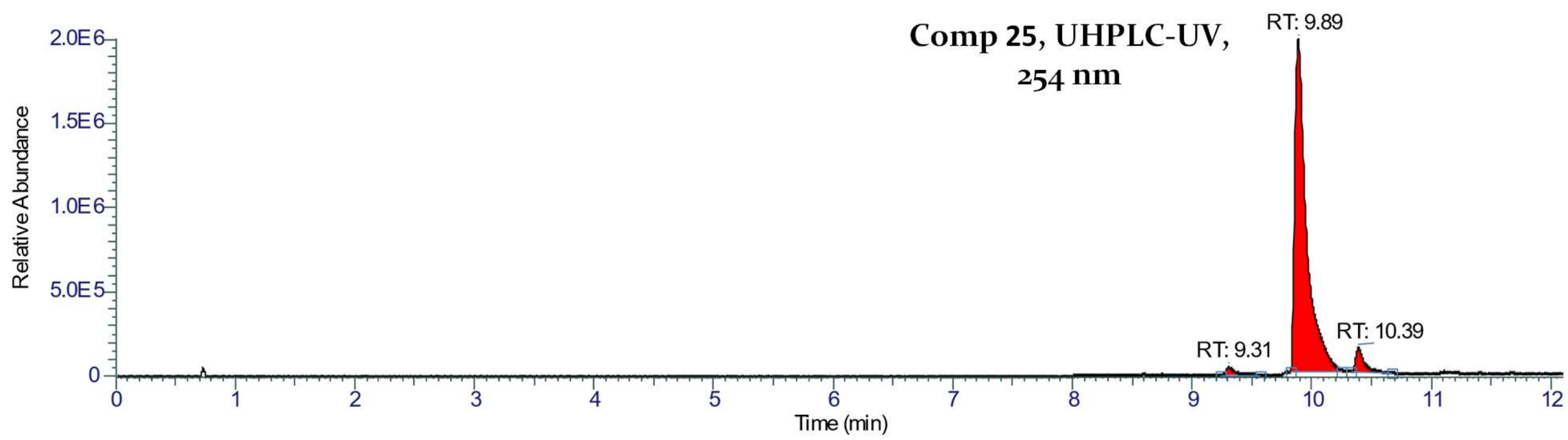

LR HPLC-MS BPC chromatogram of comp. 25, +ESI, m/z 200–2000

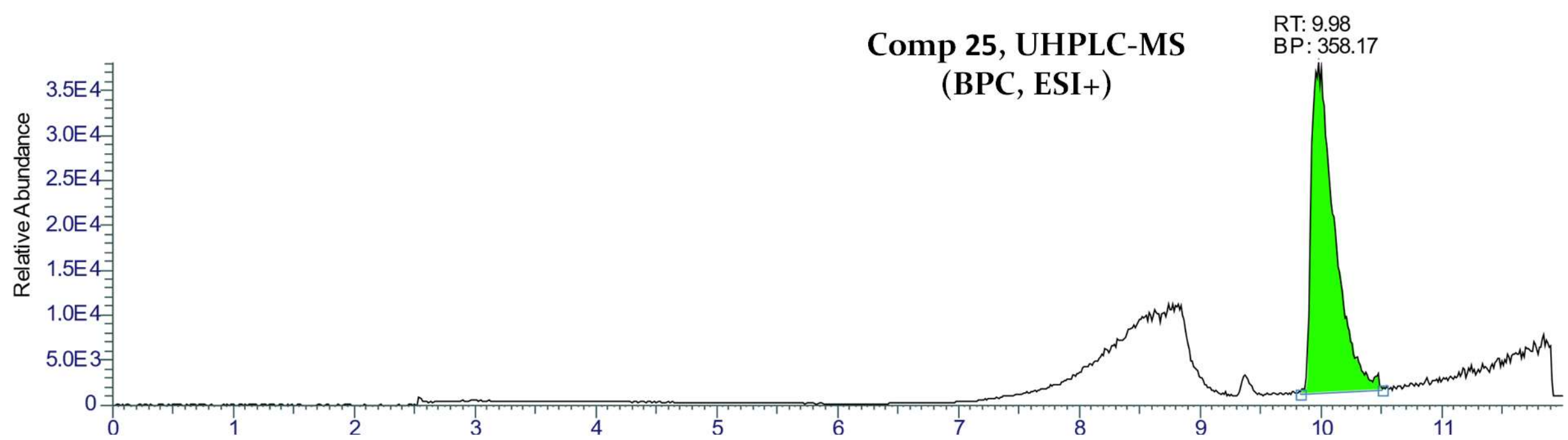

LR HPLC-MS BP spectrum of comp. 25, +ESI, m/z 200–2000

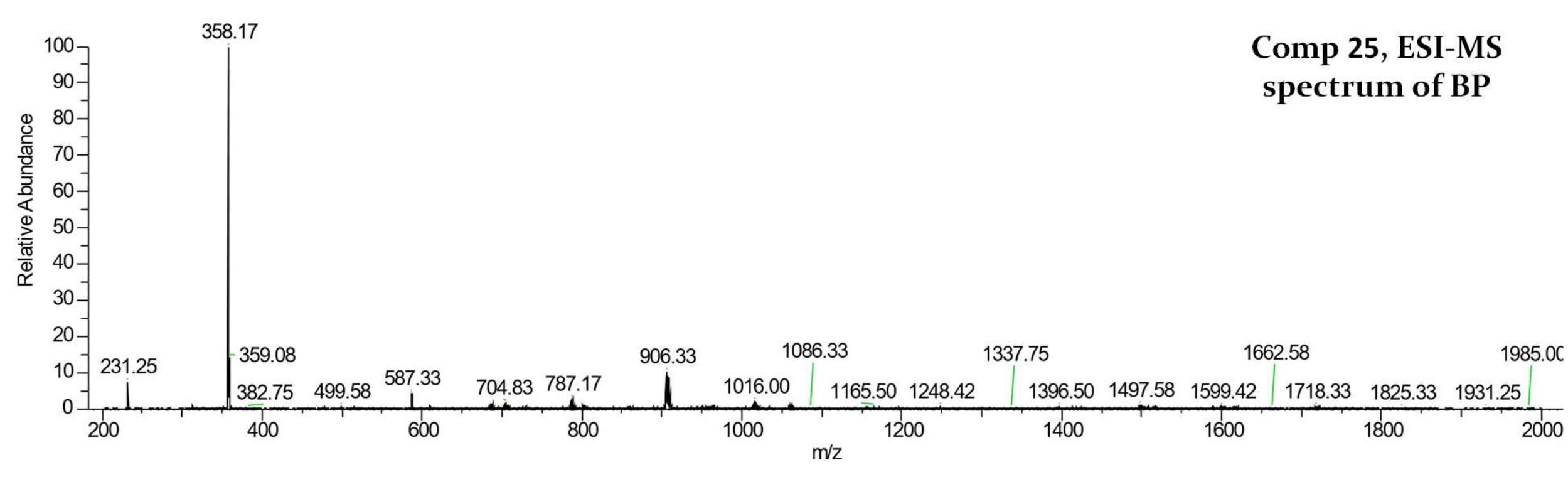

Comp. 26

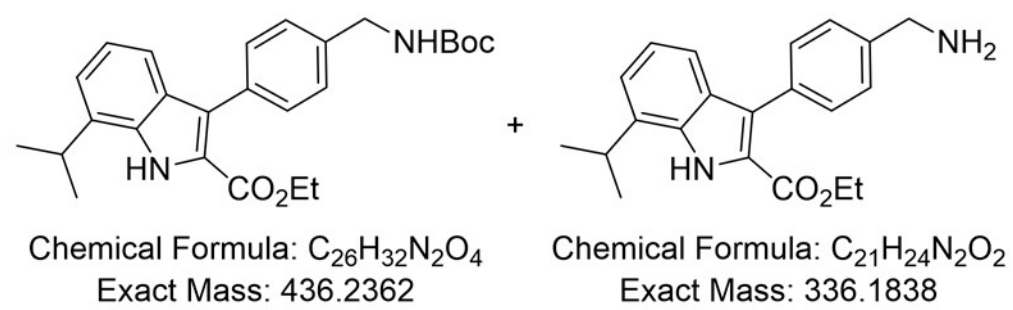

LR HPLC-MS chromatogram of comp. 26, UV 254 nm, C_{18}, 0–100% H_2O/MeCN+0.1% FA (linear gradient, 0-12 min), +ESI (pur. 71%)

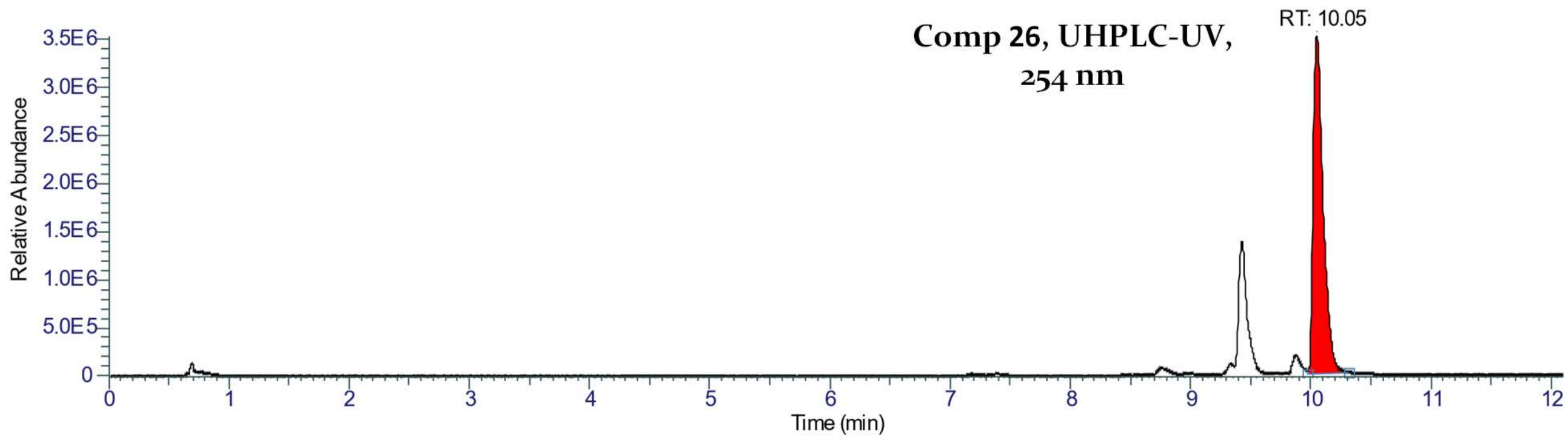

LR HPLC-MS BPC chromatogram of comp. 26, +ESI, m/z 200–2000

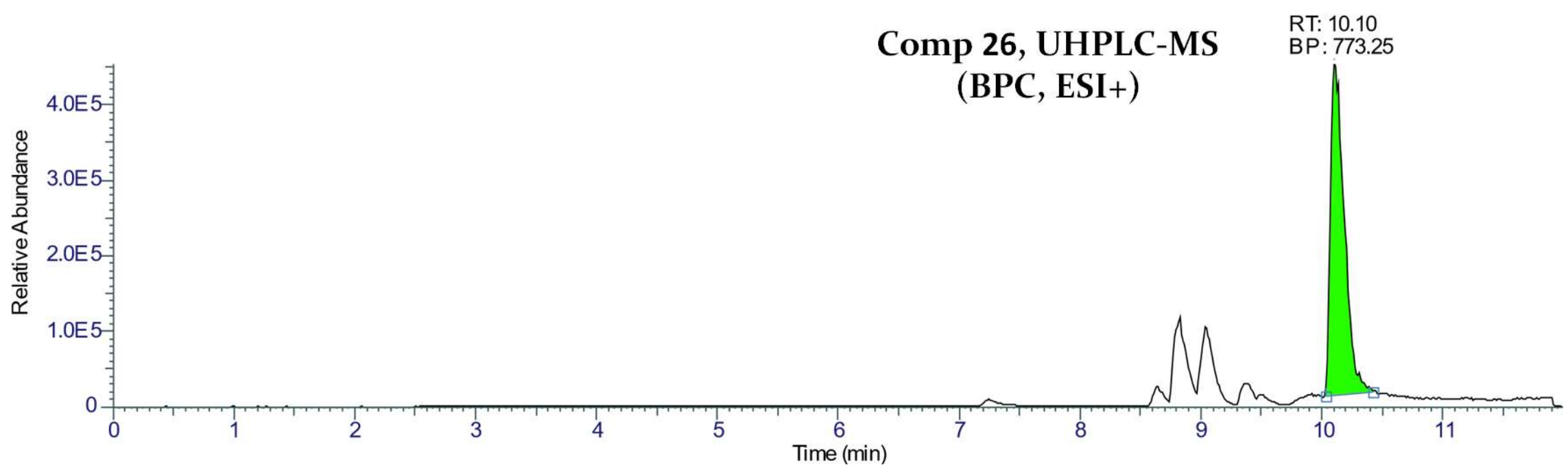

LR HPLC-MS BP spectrum of comp. 26, +ESI, m/z 200–2000

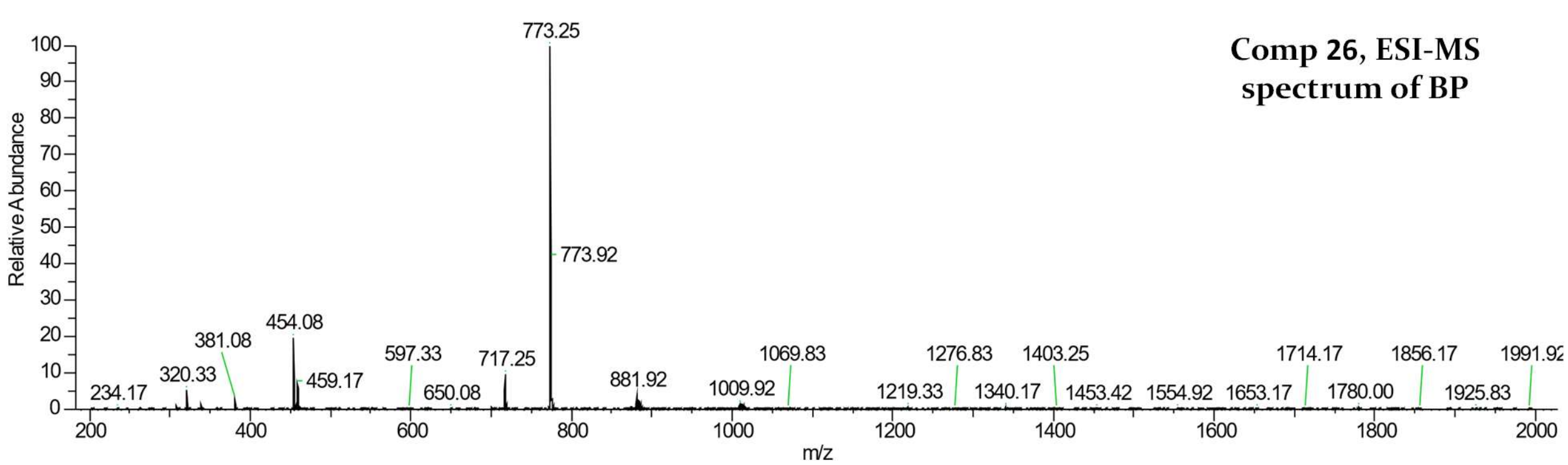

Comp. 27

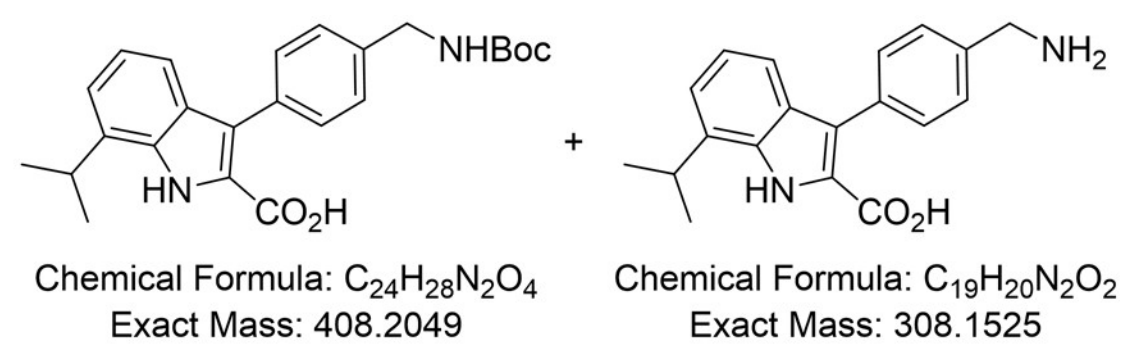

LR HPLC-MS chromatogram of comp. 27, UV 254 nm, C_{18}, 0–100% H_2O/MeCN+0.1% FA (linear gradient, 0-12 min), +ESI (pur. 99%)

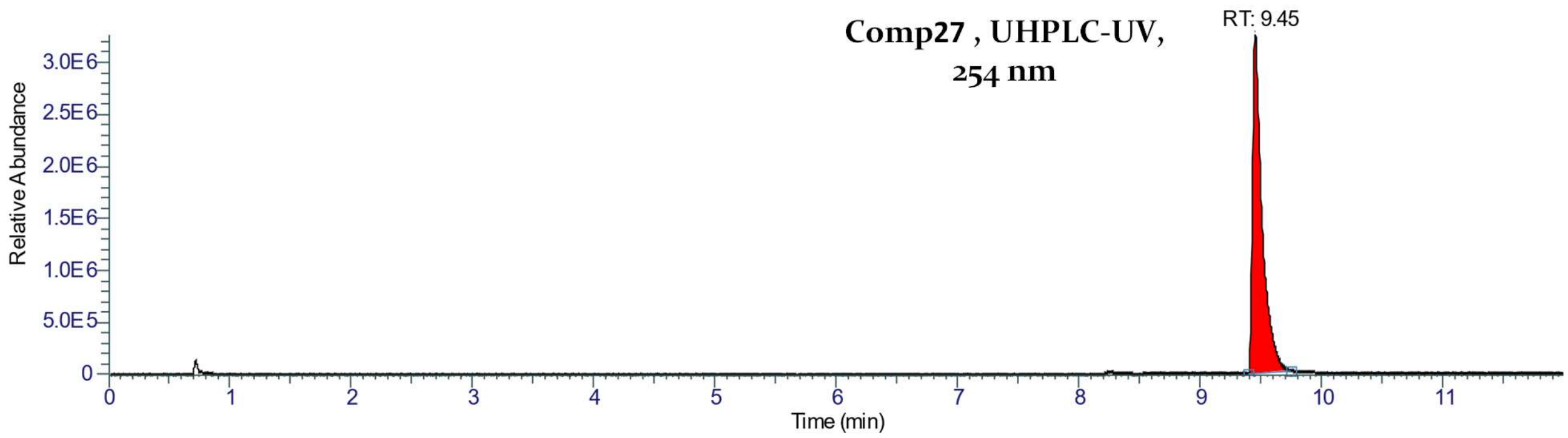

LR HPLC-MS BPC chromatogram of comp. 27, +ESI, m/z 200–2000

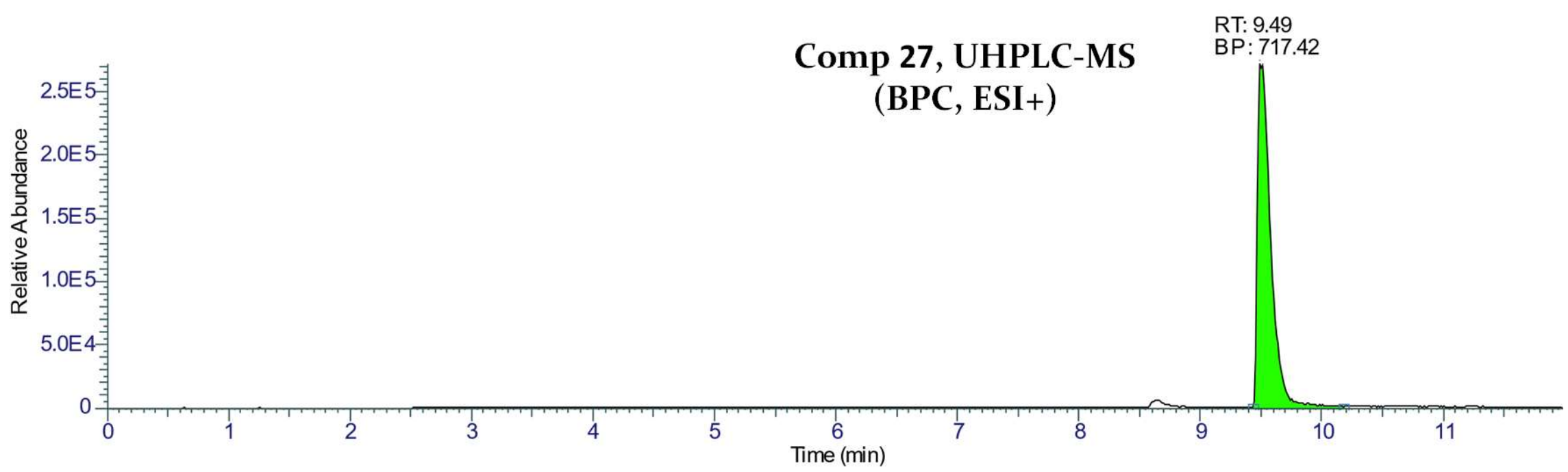

LR HPLC-MS BP spectrum of comp. 27, +ESI, m/z 200–2000

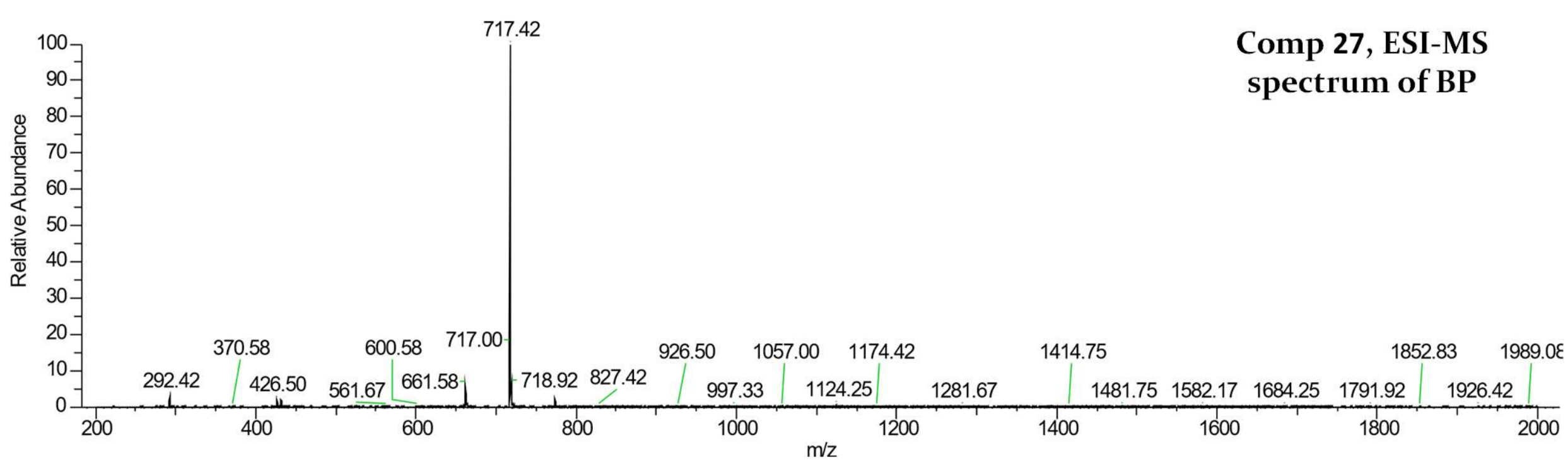

Comp 28.

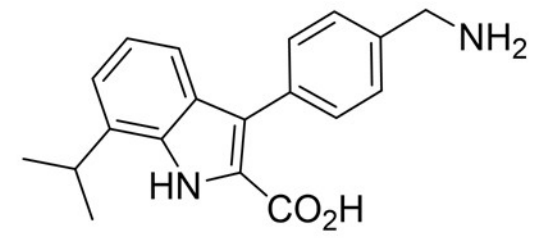

Chemical Formula: $C_{19}H_{20}N_2O_2$
Exact Mass: 308.1525

LR HPLC-MS chromatogram of comp. 28, UV 254 nm, C_{18}, 0–100% H_2O/MeCN+0.1% FA (linear gradient, 0-12 min), +ESI (pur. 98%)

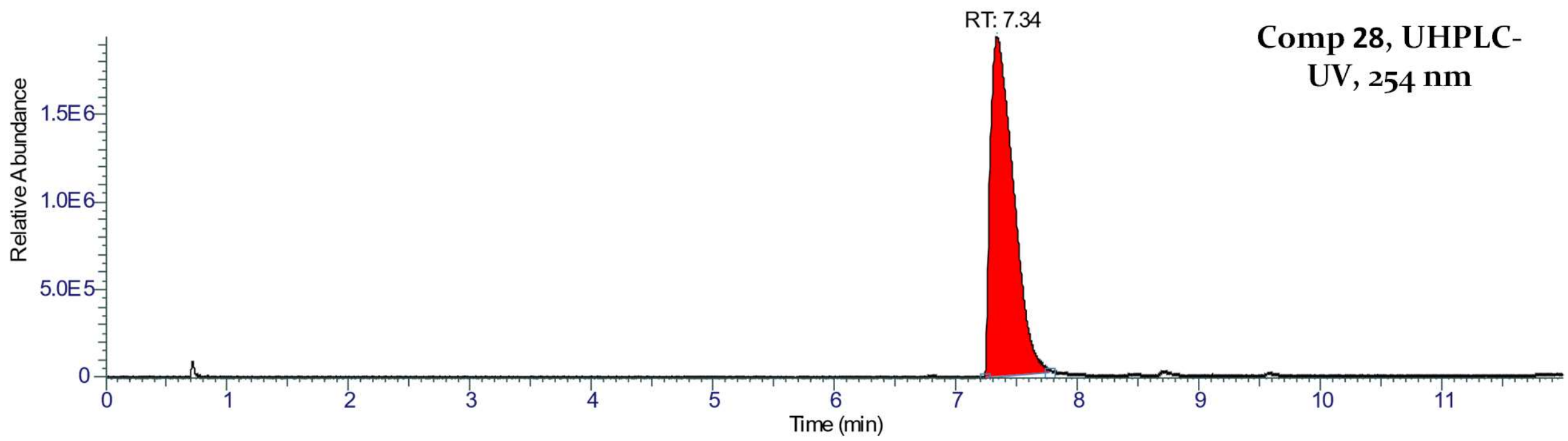

LR HPLC-MS BPC chromatogram of comp. 28, +ESI, m/z 200–2000

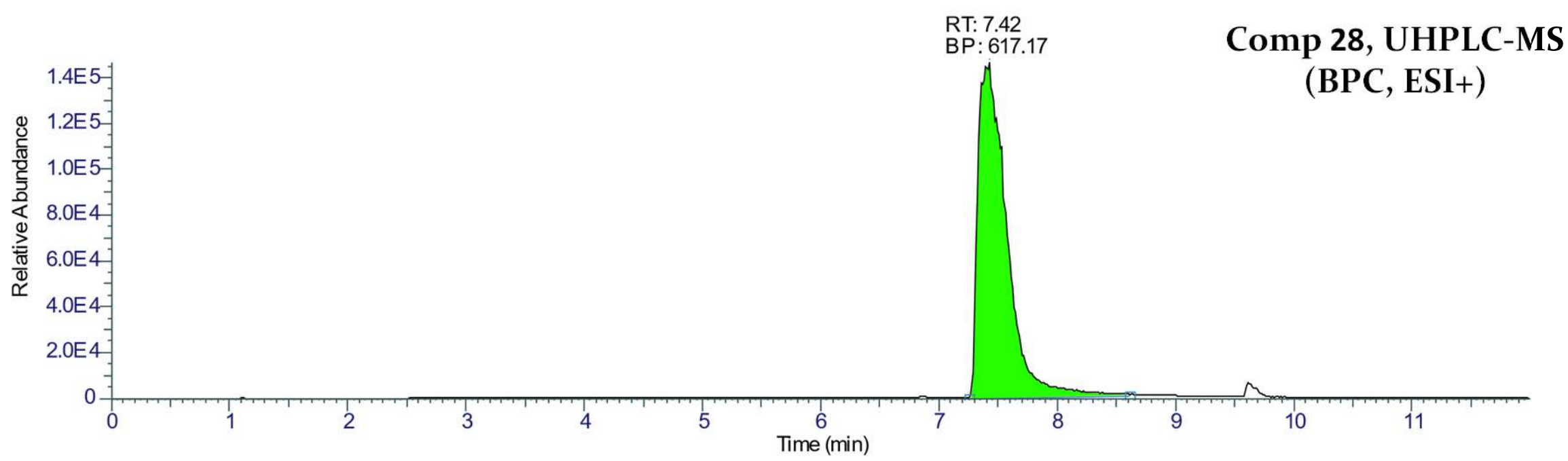

LR HPLC-MS BP spectrum of comp. 28, +ESI, m/z 200–2000

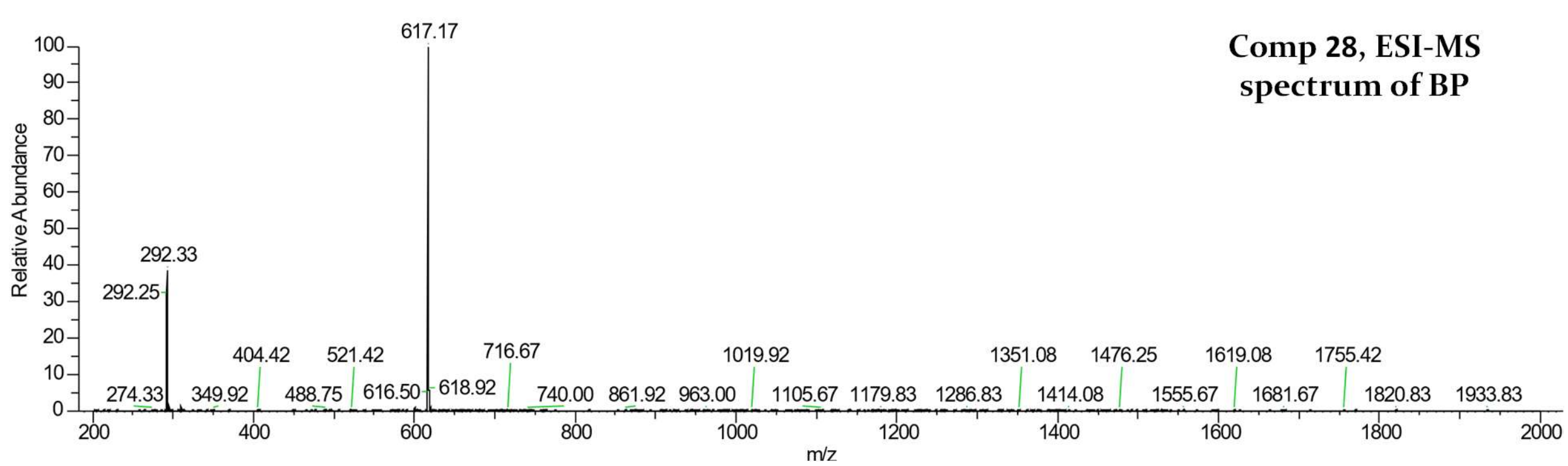

NMR data

***MS1*, ^{1}H NMR in DMSO-d_6, Bruker-300**

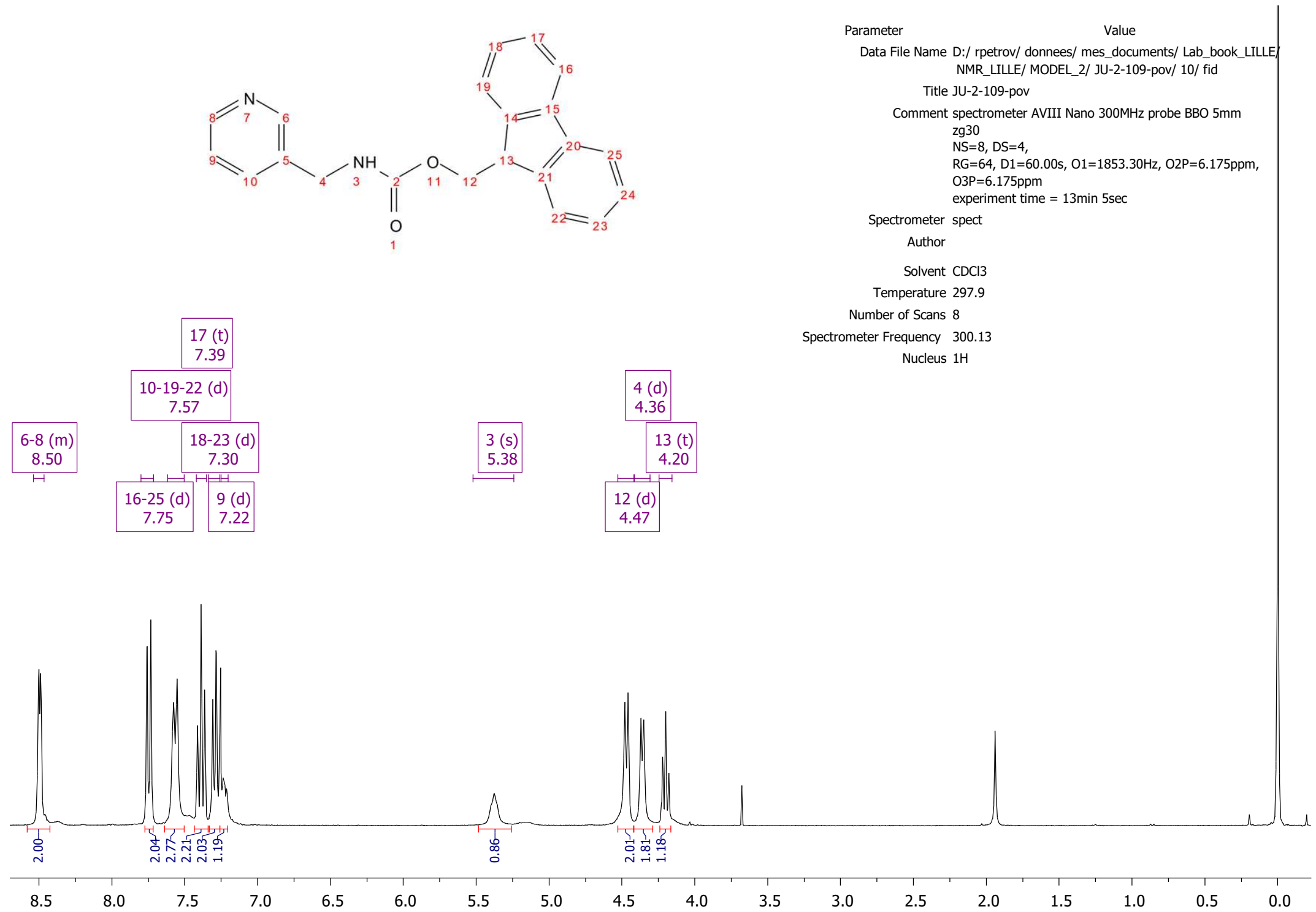

***MS1*, ^{13}C NMR of DMSO-d_6, Bruker-300**

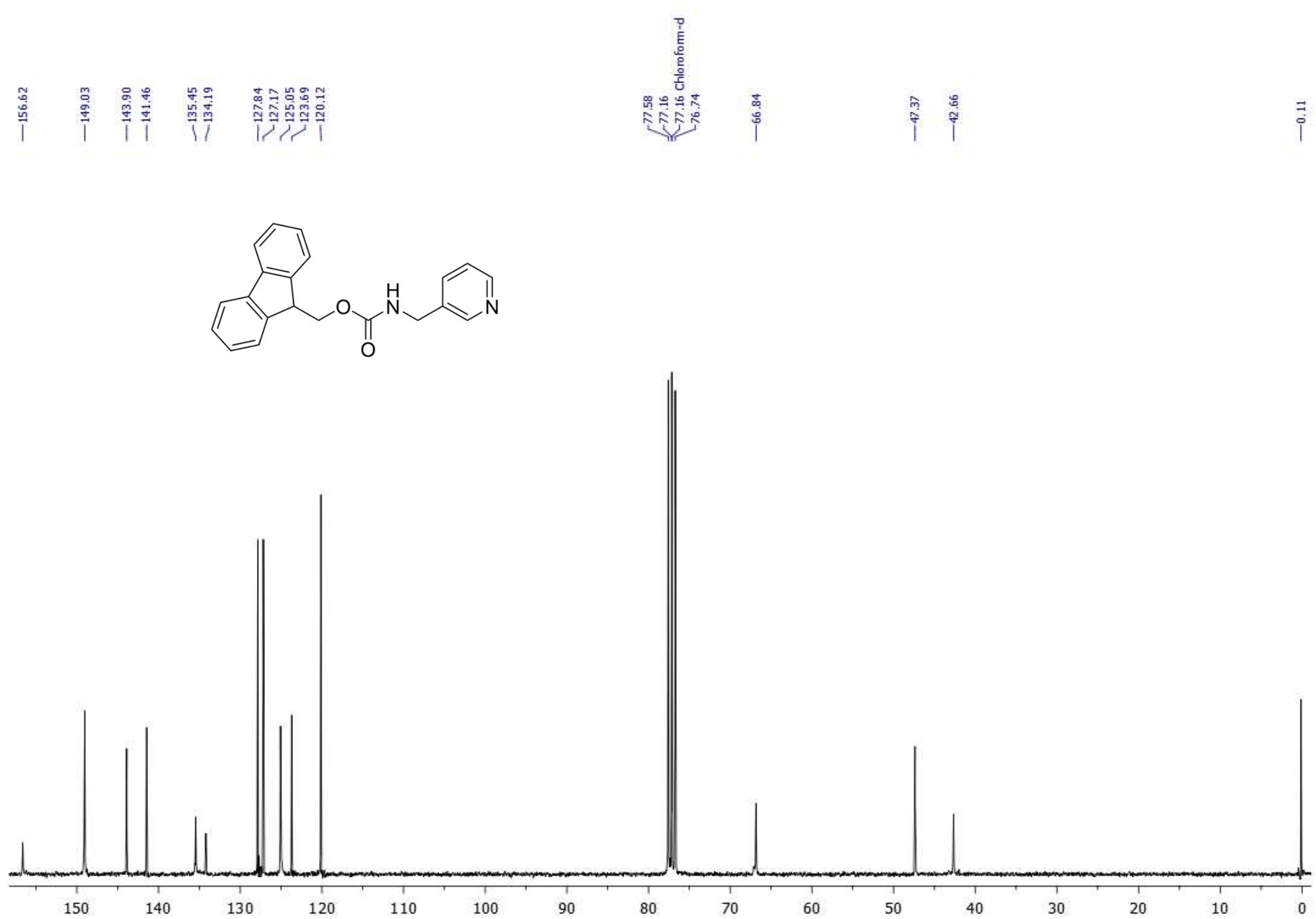

Comp. *MS1* ($^1H/^{13}C/^{15}N$ chemical shifts assignments)

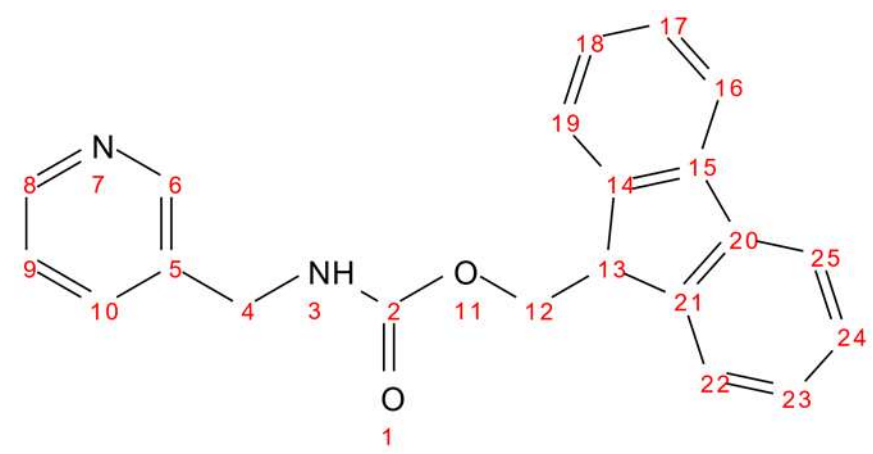

Table 1. $^1H/^{13}C/^{15}N$ chemical shifts assignments in $CDCl_3$ for ***MS1*** at 298K and 7 T referenced against the residual peak of TMS. ovl.: overlap.

Atom No.	1H /ppm	Mult., J, Hz	Integral	13C ppm	15N ppm	HMBC cor. atom no.	TOCSY cor. atom no.	NOE cor. atom no.
	300 MHz			75.47 MHz	30.41 MHz			
2				156.62				
3	5.38	br. s	1H	-			4	10
4	4.36	d, 5.95	2H	42.66		2, 5, 10, 6	3, 6	6
5	-	-	-	134.19				
6	8.5	m	ovl	149.08			4, 9, 10	4
8	8.5	m	ovl	149.03			9, 10	9
9	7.22	dd, 7.22, 7.26	ovl 1H	123.69			6, 8, 10	8
10	7.57	d, 7.26	ovl	135.45		4	6, 9	3
12	4.47	d, 6.64	2H	66.85		2, 13, 20	13	19, 22
13	4.2	t, 6.56	1H	47.38		12, 19, 20, 21, 22	12	19, 22
14	-	-	-	141.46				
15	-	-	-	143.90				
16	7.75	d, 7.51	ovl	120.12				
17	7.39	dd, 7.35	ovl	127.84				
18	7.3	d, 7.16	ovl	127.17				
19	7.57	d, 7.26	ovl	125.05		13		12, 13
20	-	-	-	143.90				
21	-	-	-	141.46				
22	7.57	d, 7.26	ovl	125.05		13		12, 13, 23
23	7.3	d, 7.16	ovl	127.17				22
24	7.39	dd, 7.35	ovl	127.84				25
25	7.75	d, 7.51	ovl	120.12				24

Effect of iodine and iodine/STF mixture on the ^{1}H NMR spectrum of comp MS1

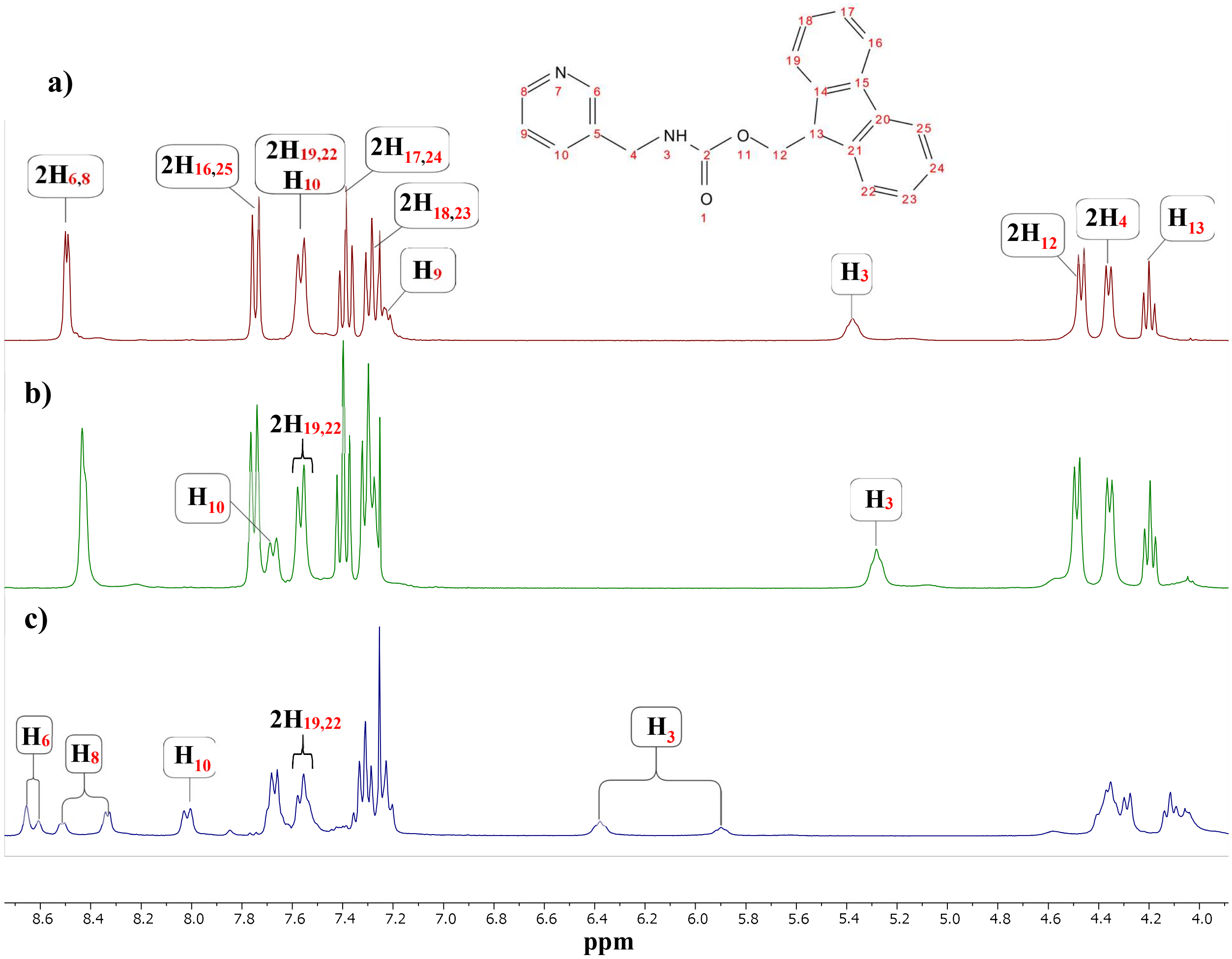

Figure 1. ^{1}H (300 MHz, $CDCl_3$, 298 K) of (a) comp ***MS1***; (b) 1:1 mixture of ***MS1*** and iodine; (c) compound ***MS1*** in the presence of 1 eq. iodine, followed by 1 eq. STF showing a 2:1 ratio for the amide NH proton and downfield shifts of the pyridine aromatic protons.

Comp. *MS3*, ^{1}H NMR in DMSO-d_6

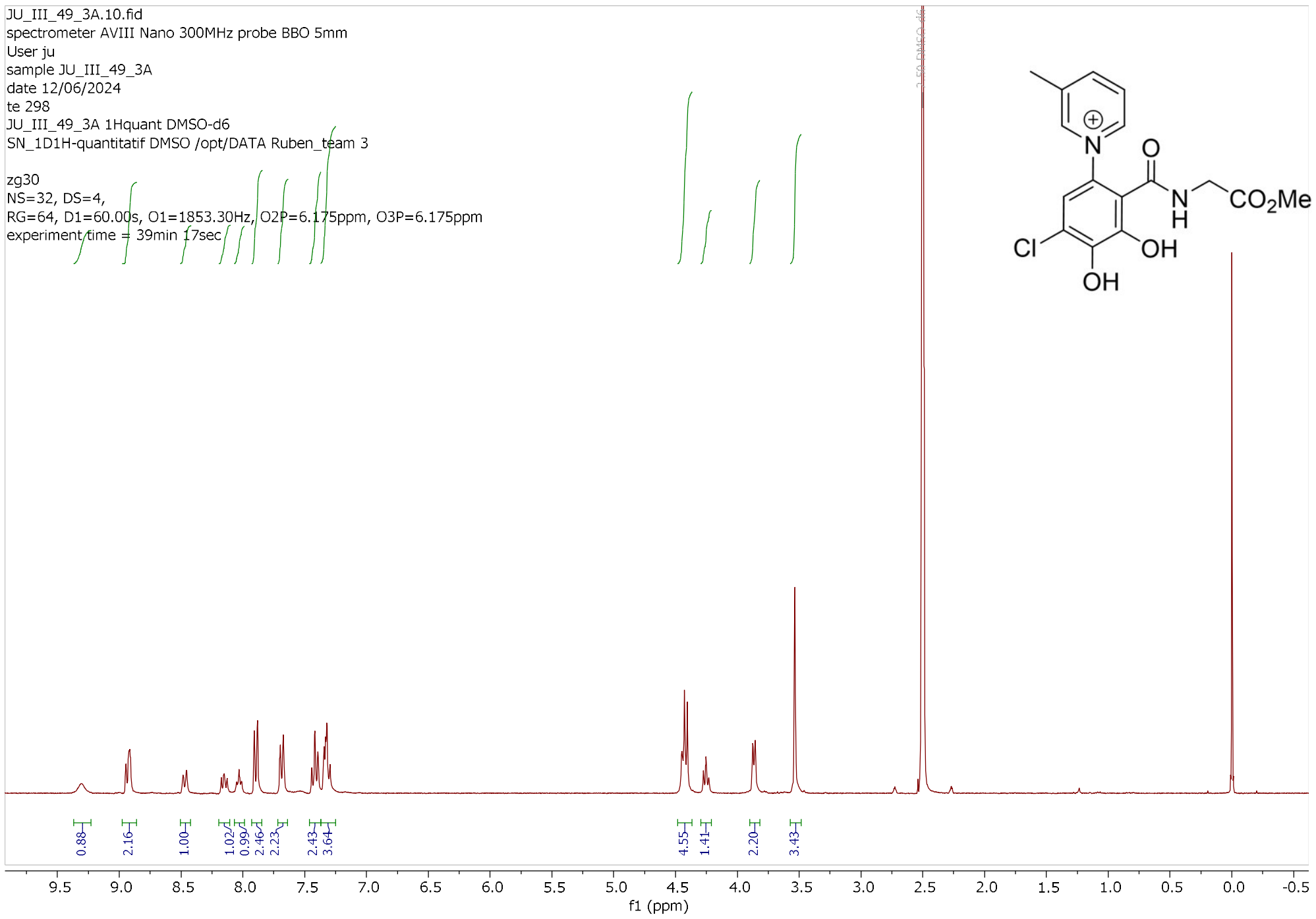

Comp. *MS3*, ^{13}C NMR in DMSO-d_6

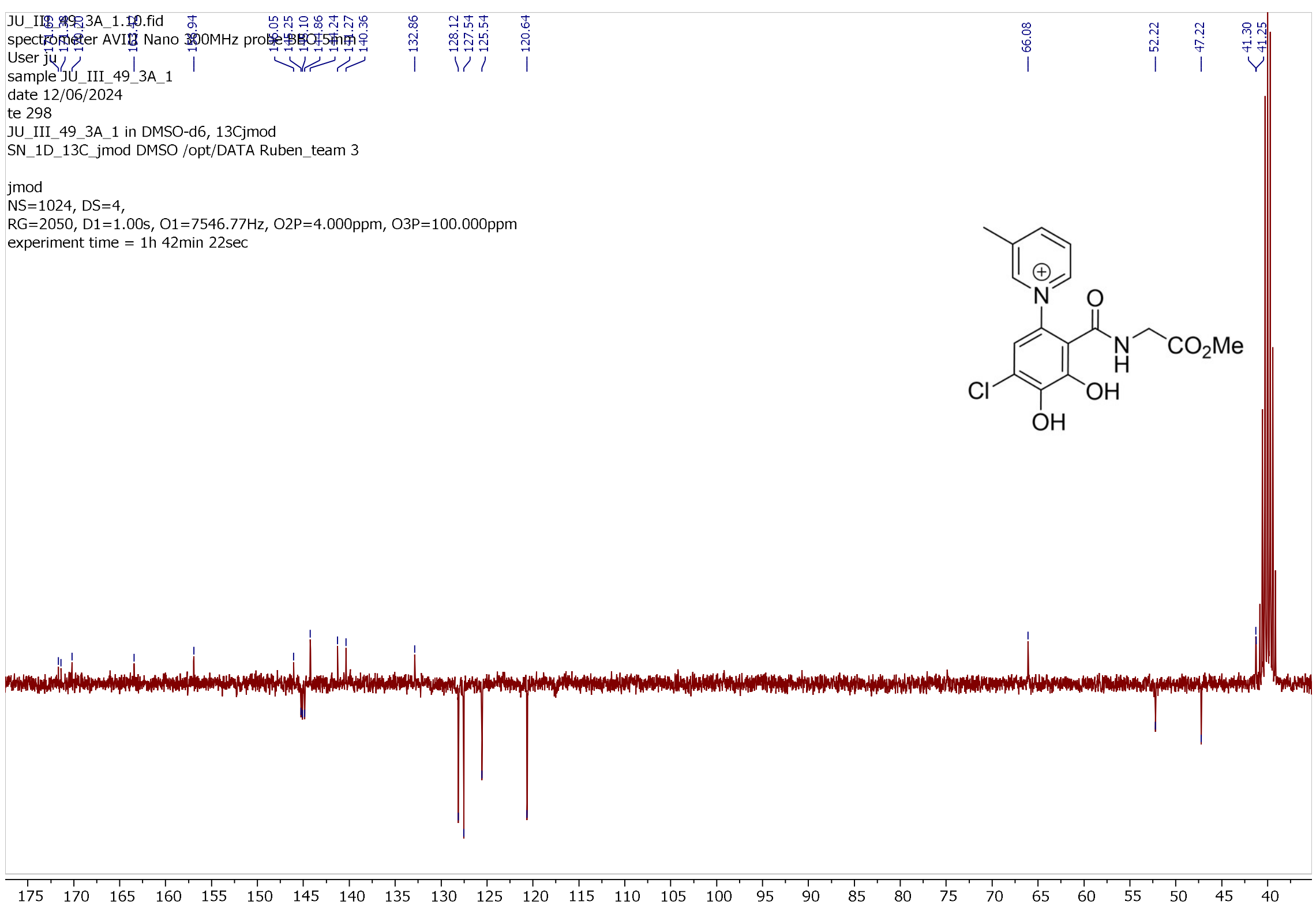

Comp. *MS3* ($^1H/^{13}C/^{15}N$ chemical shifts assignments)

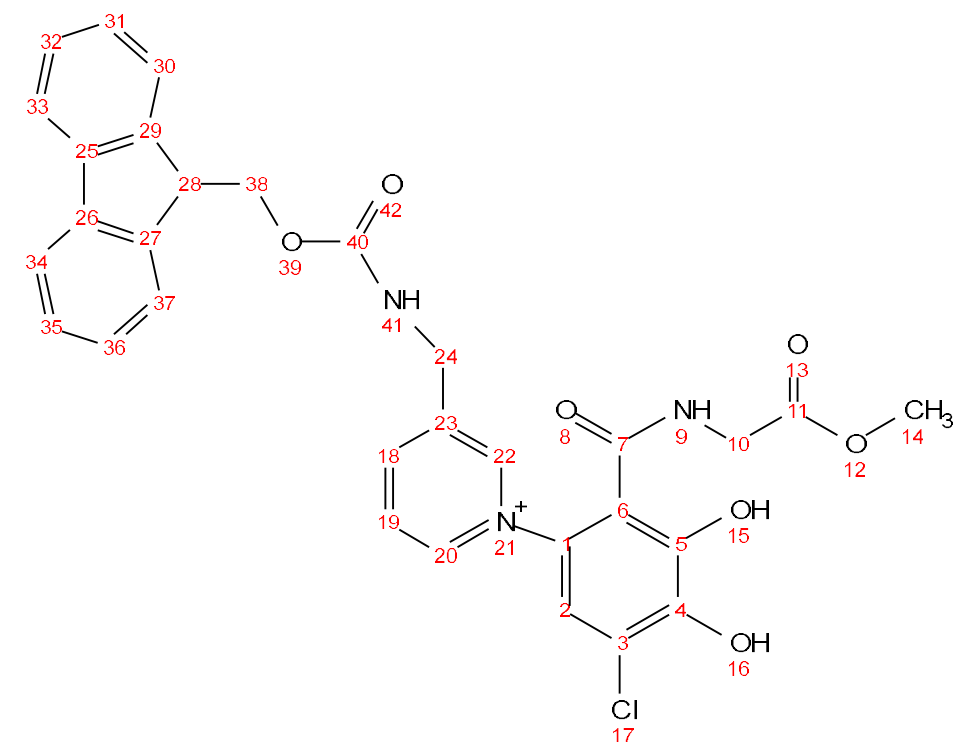

Table 1. $^1H/^{13}C/^{15}N$ chemical shifts assignments in CD_3OD for ***MS3*** at 293K and 14 T referenced against the residual peak of solvent. ov.: overlap.

Atom number	^1H /ppm	Mult., J (Hz)	no H's	Integral	^{13}C /ppm	^{15}N /ppm	HMBC cor. atom no.	COSY cor. atom no.	NOE cor. atom no.
	600 MHz				151 MHz	61 MHz			
1					132.3		2,20,22		
2	7.36	s		1H	117.4				20,22
3					120.5		2		
4					145.5		2		
6					118.2		2		
7					162.9		10		
9	9.25	br		1H		116	10	10	10
10	3.86	d, 5.8		2H	40.9			9	9
11					169.8		10,14		
14	3.53	s		ov.	51.8				
18	8.47	d, 8.2		1H	144.7			19	24
19	8.16	dd, 6.2&8.2		1H	127.2			18,20	
20	8.94	d, 6.2		1H	144.8			19	2
21						211	2,20,22		
22	8.91	s		1H	144.4				2,24,41
23					140.0		19,22,24		
24	4.44	d, 6.1		2H	40.8			41	18,22
25/26					140.8		28,30/37,32/35		
27/29					143.8		28,31/36,33/34, 38		
28	4.25	t, 6.7		1H	46.8			38	30/37
30/37	7.68	d, 7.5		2H	125.1			31/36	28,38
31/36	7.32	td, 7.5&1.0		2H	127.1			30/37,32/ 35	

32/35	7.42	t, 7.5		2H	127.7			31/36,33/34	
33/34	7.90	d, 7.5		2H	120.2			32/35	
38	4.41	d, 6.7		2H	65.6			28	30/37
40					156.5		24,38,41		
41	8.05	t, 6.1		1H		81	41	24	22

Comp. *MS5* ($^{1}H/^{13}C/^{15}N$ chemical shifts assignments)

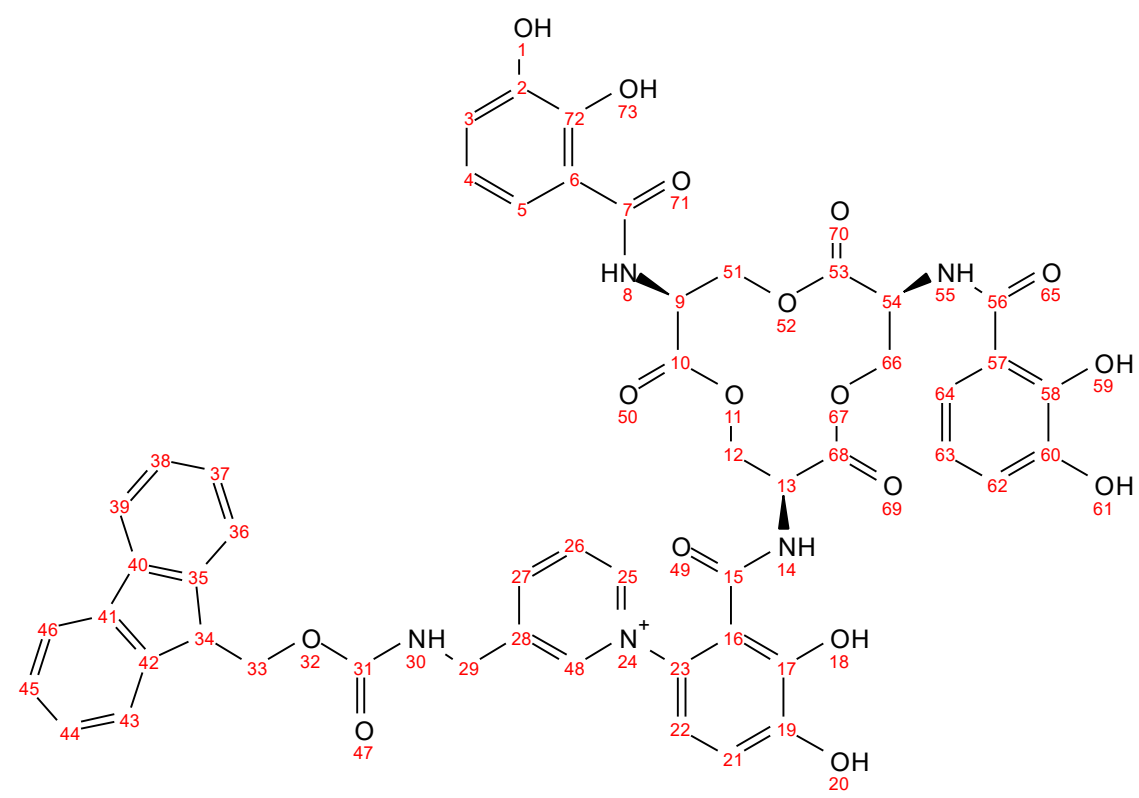

Table 1. $^{1}H/^{13}C/^{15}N$ chemical shifts assignments in DMSO-d_6 for ***MS4*** at 293K and 14 T referenced against the residual peak of DMSO.

Atom number	^{1}H /ppm 600 MHz	J Hz	no H's	Integral	^{13}C /ppm 151 MHz	^{15}N /ppm 61 MHz	HMBC cor. atom no.	HSQC cor. atom no.	COSY cor. atom no.	NOE cor. atom no.
2					145.7		4			
3	7.01	dd, 1.3&7.8			118.7				4/5	
4	6.73	t, 7.9			118.7				3/5	
5	7.35	dd, 1.5&8.0			119.9				3/4	8
6					117.6		4			
7					166.1		5			
8	9.39	br				111			9	5/9/51/51’
9	4.94	td, 3.3&5.8			52.8		10		8/51/51’	8
10					168.1		9/51			
12	4.06	br			65.7		68		12’/13	14
12’	4.75	br			65.7				12/13	
13	5.00	br			51.1				12/12’	14
14	12.41	br			115					12/13
15					165.6					
21	6.79	br			110.0				22	
22	6.33	br			109.1				21	25/48
23					134.8		25/48			
24						219	25/26			
25	8.92	d, 6.0			145.0				26	22
26	8.01	t, 6.9			126.1				25/27	
27	8.35	d, 7.8			143.3				26/48	29

Atom number	^{1}H /ppm	J Hz	no H's	Integral	^{13}C /ppm	^{15}N /ppm	HMBC cor. atom no.	HSQC cor. atom no.	COSY cor. atom no.	NOE cor. atom no.
	600 MHz				151 MHz	61 MHz				
28					138.9		26/29/48			
29	4.40	d, 6.0			40.6				30	27/48
30	8.03					81	29		29	
31					156.5		30/33			
33	4.42	d, 6.5			65.5				34	36/43
34	4.24	t, 6.5			46.8				33/36/43	
35/42					143.8		33/34/39/46			
36/43	7.68	d, 7.5			125.1				34/37/44	33
37/44	7.30	t, 7.5			127.1				36/43/38/45	
38/45	7.40	t, 7.5			127.6				37/44/39/46	
39/46	7.88	d, 7.5			120.2				38/45	
40/41					140.8		34/3643			
48	8.87	s			144.4				27	22/29
51	4.46				65.2		10		9/51’	8
51’	4.62				65.2				9/51	8
53					169.0		54/66/66’			
54	4.88				51.2		53/56		55/66/66’	55
55	9.11	d, 7.8				112			54	54/64/66/66’
56					168.5		54/55/64			
57					115.6		63			
58					148.3		62/64			
60					146.1		63			
62	6.95	dd, 1.4&7.8			119.1				63/64	
63	6.71	t, 7.9			118.5				62/64	
64	7.27	d, 8.0			118.3				62/63	55
66	4.25				63.8		53/68		54/66’	55
66’	4.68	dd, 7.5&11.0			63.8		53/68		54/66	55
68					169.1		12/66/66’			
72					146.6		3/5			

Comp. [bis(3-methylpyridine)I]$^+$BF$_4^-$, ^{1}H NMR in CDCl$_3$

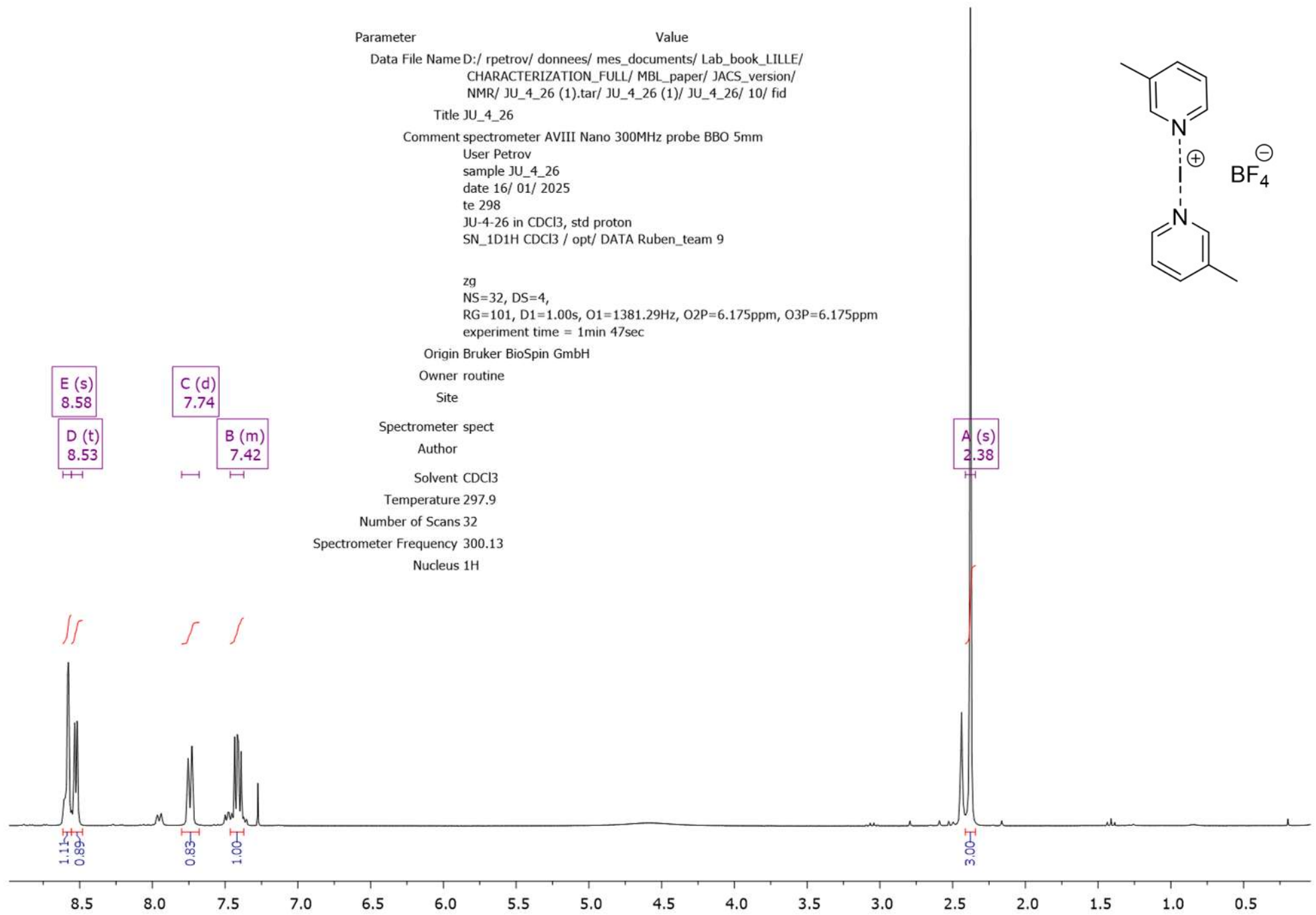

Comp. bis(3-methylpyridine)I$^+$BF$_4^-$, ^{13}C NMR in CDCl$_3$

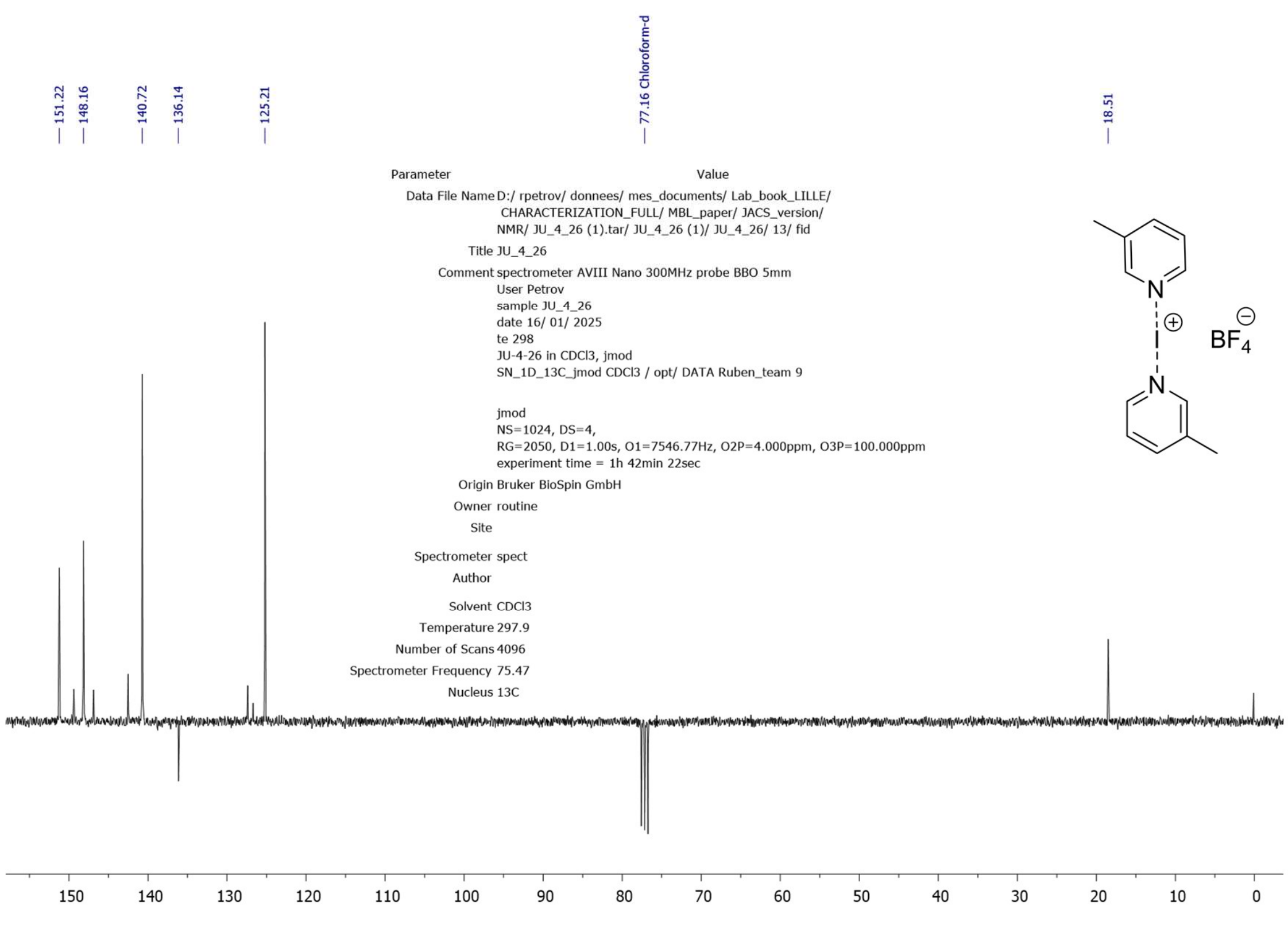

Comp. 1, ^{1}H NMR in DMSO-d_6

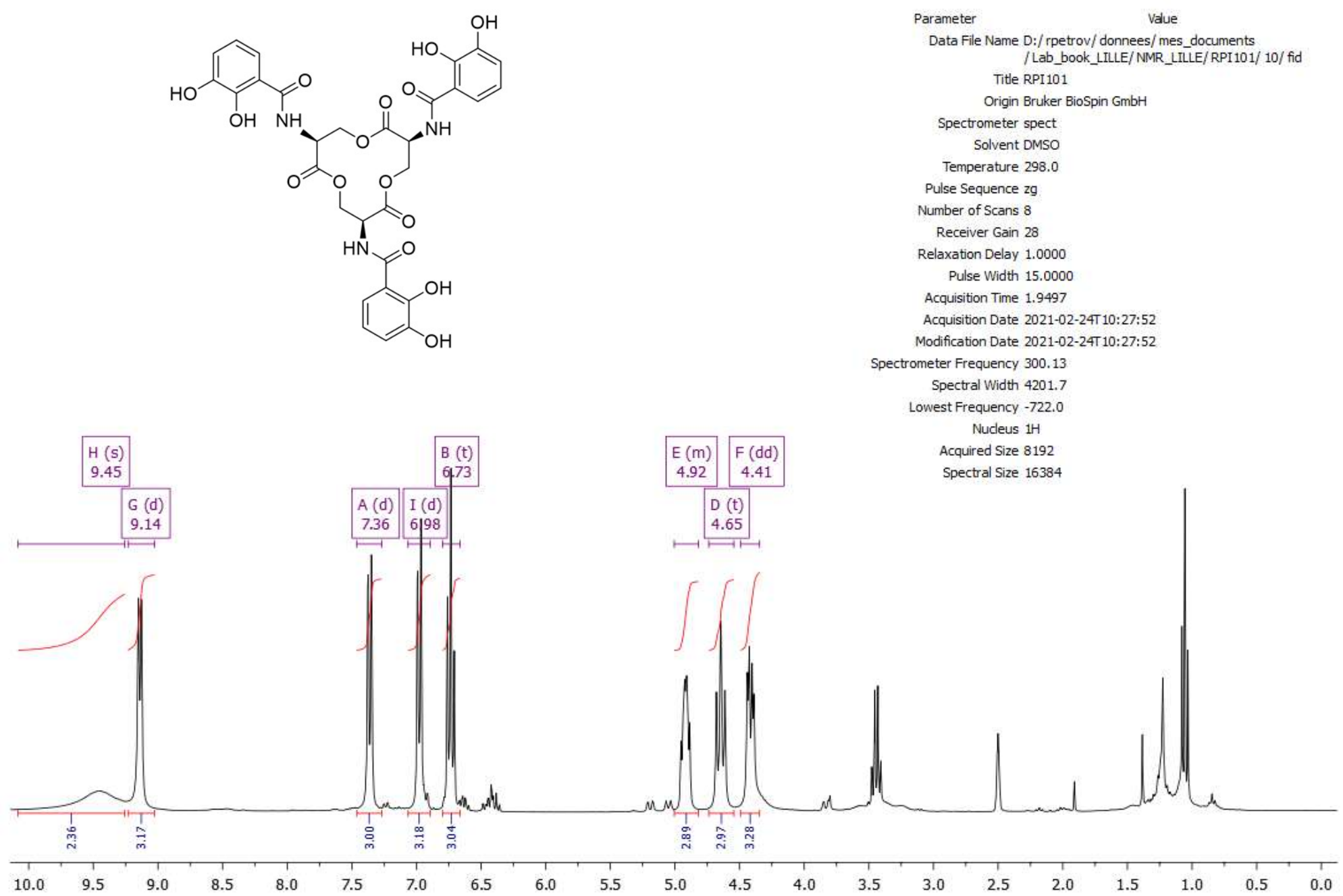

Comp. 1, ^{13}C NMR in DMSO-d_6

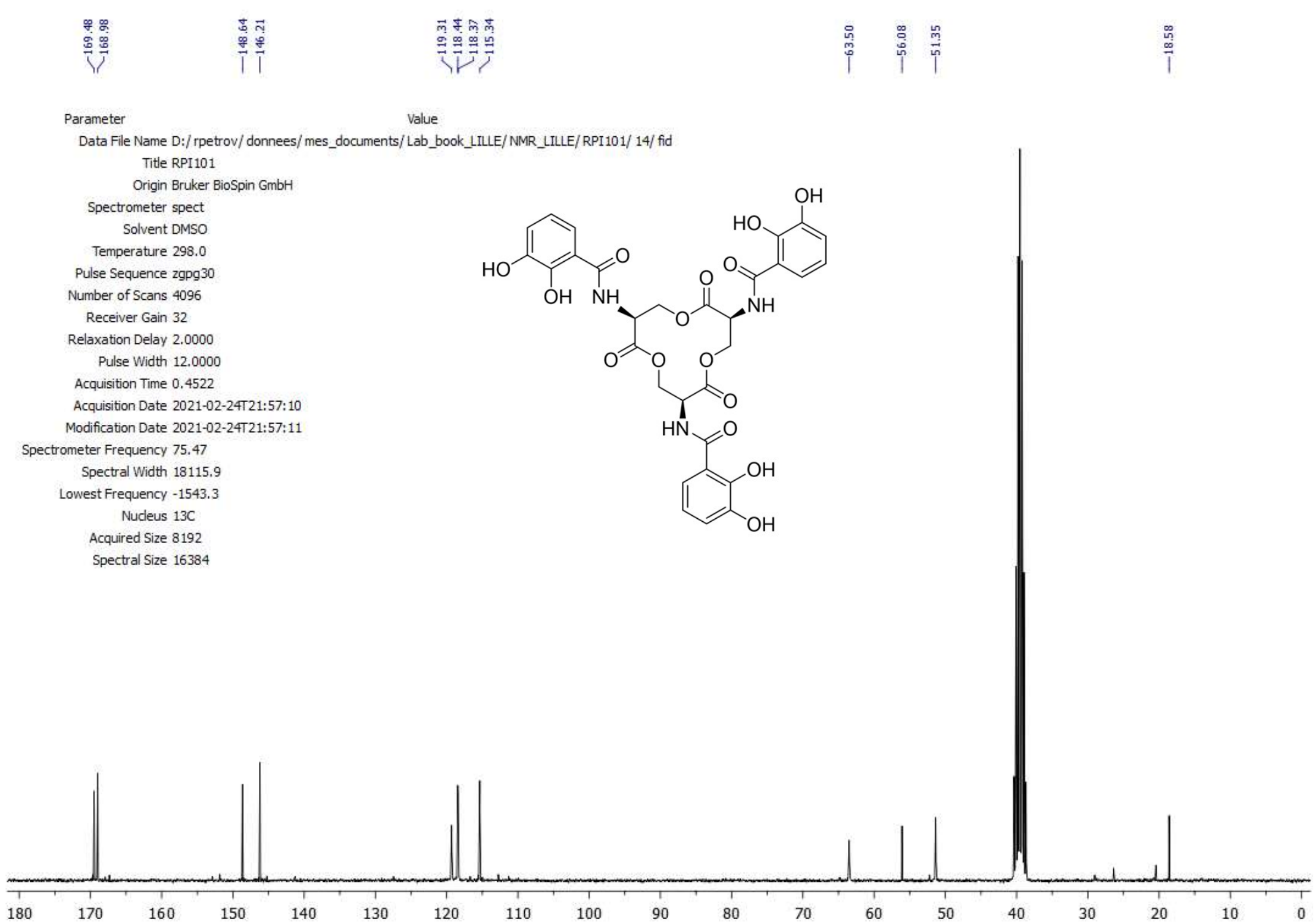

Comp. 2, ^{1}H NMR in DMSO-d_6, Bruker-300

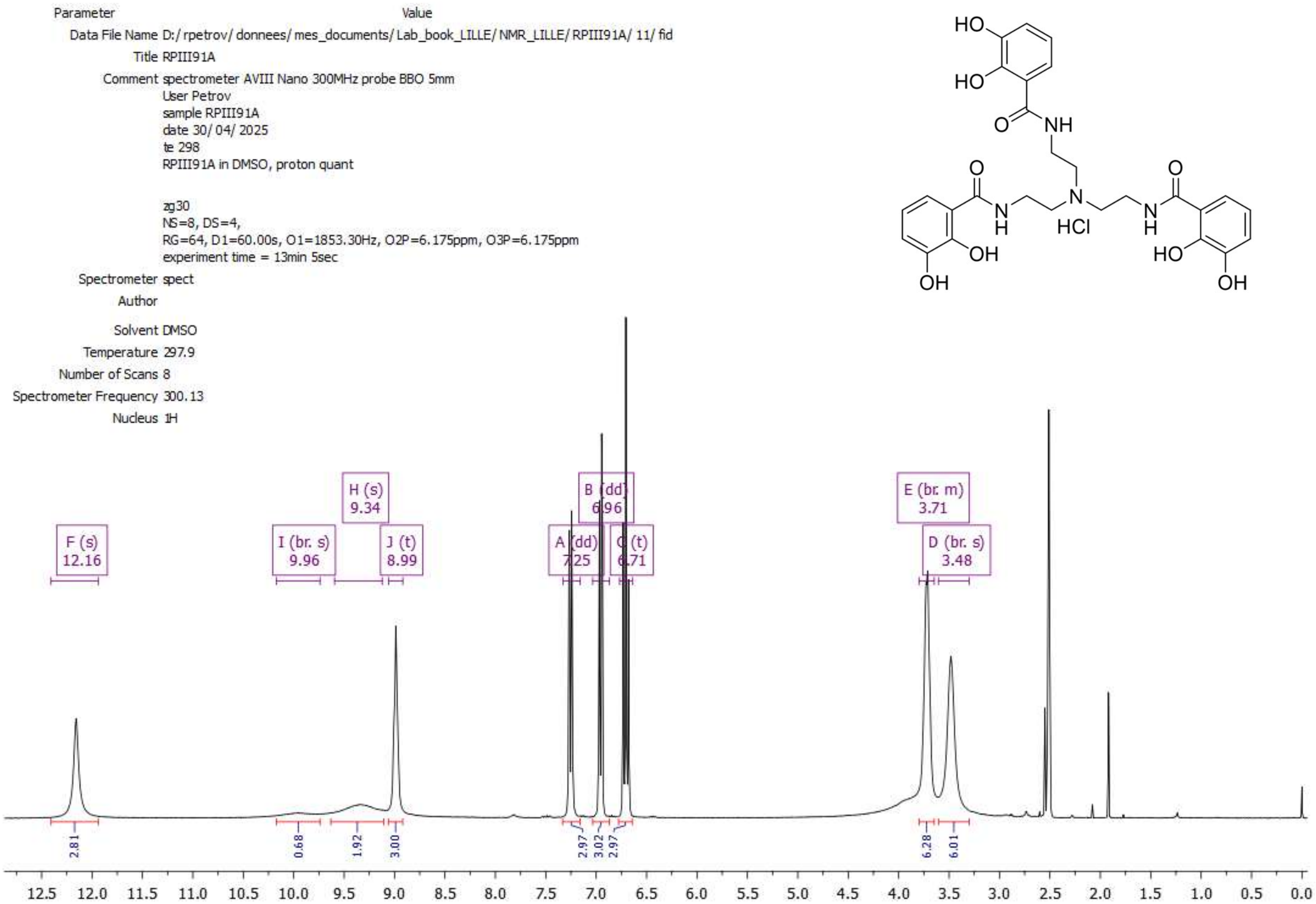

Comp. 2, ^{13}C NMR in DMSO-d_6, Bruker-300

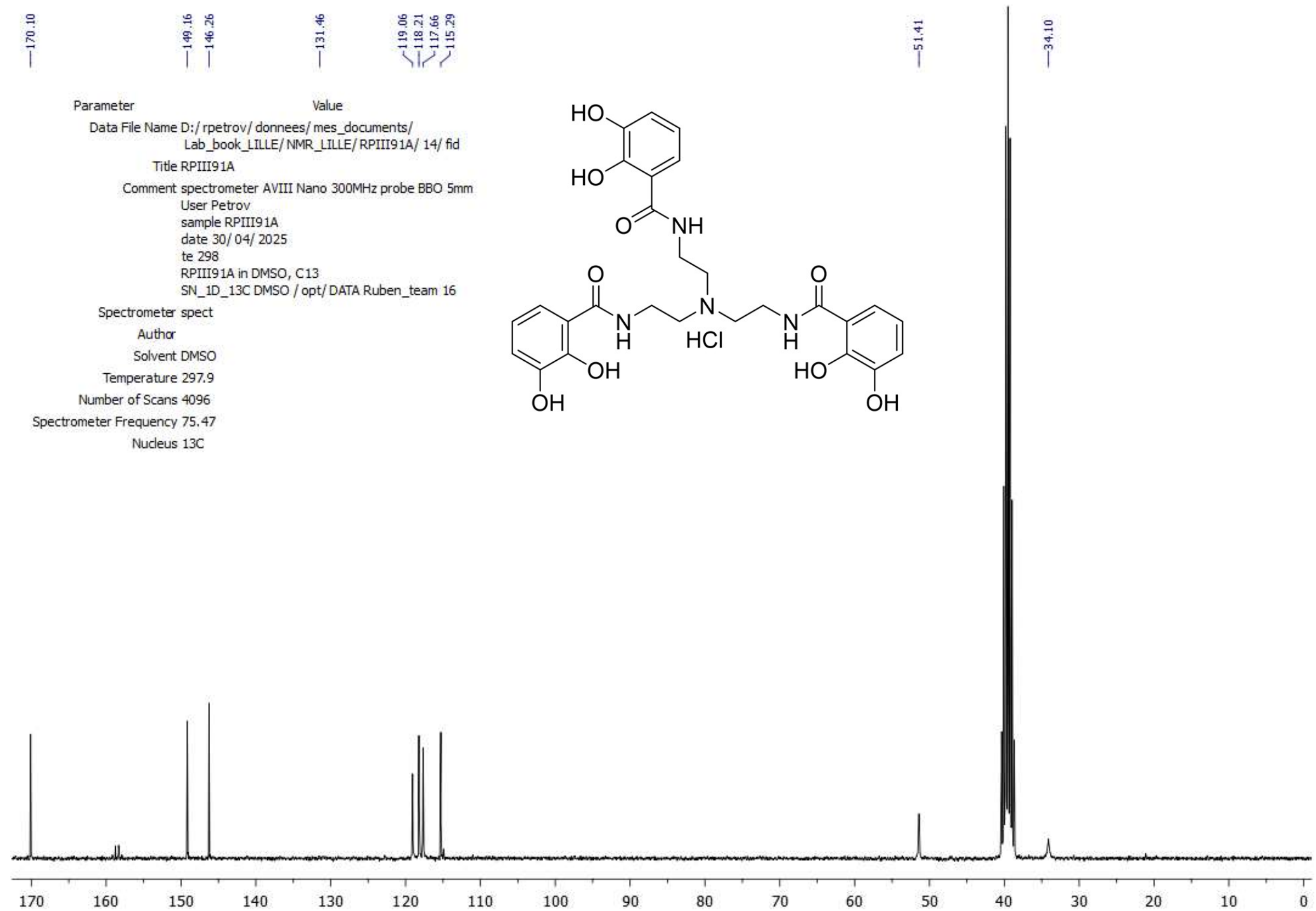

Comp. 4, ^{1}H NMR in DMSO-*d*6, Bruker-300

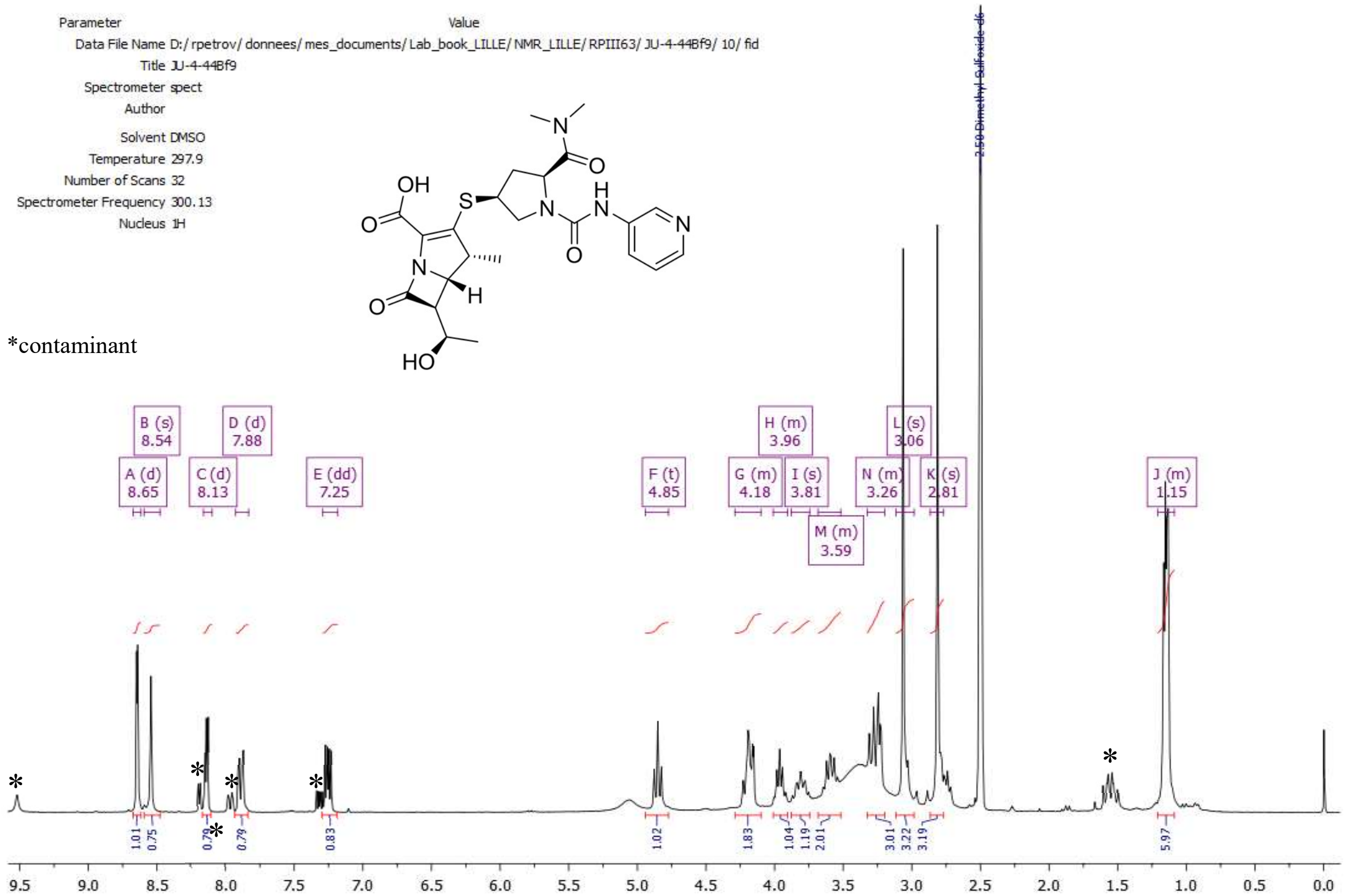

Comp. 4, ^{13}C NMR in DMSO-d_6, Bruker-300

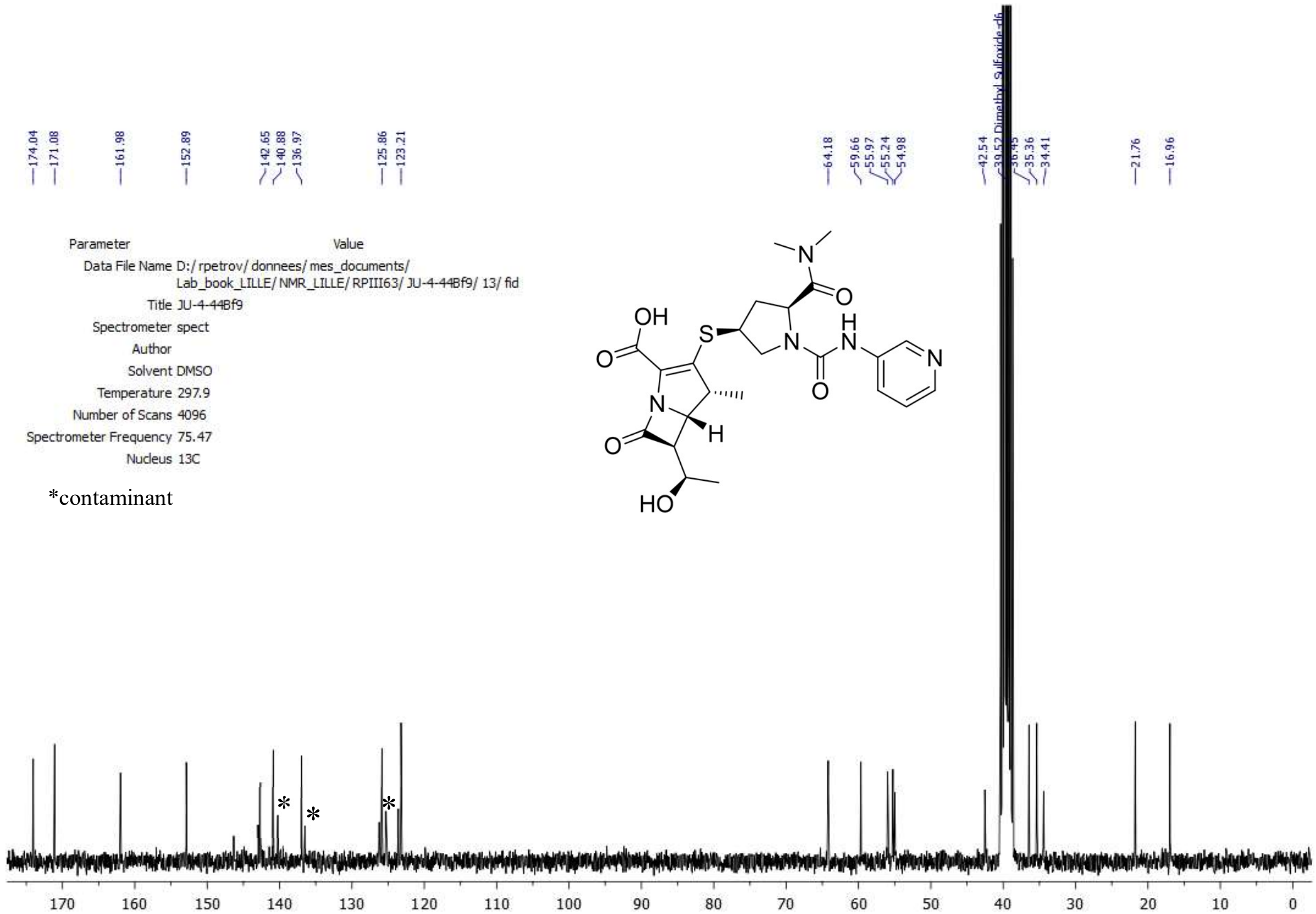

Comp. 5 ^{1}H NMR in DMSO-d_6, Bruker-300

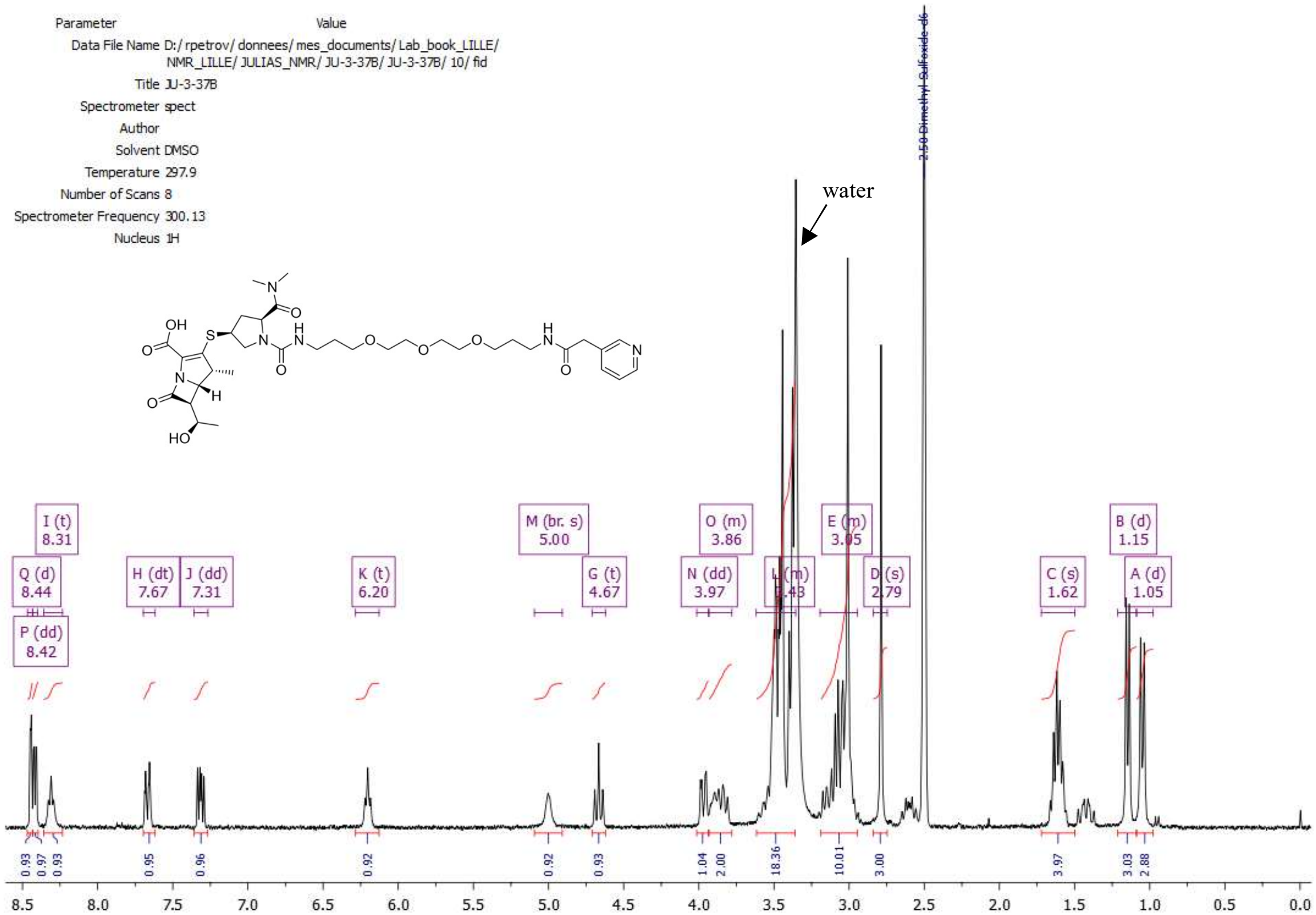

Comp. 5, ^{13}C NMR in DMSO-d_6, Bruker-300

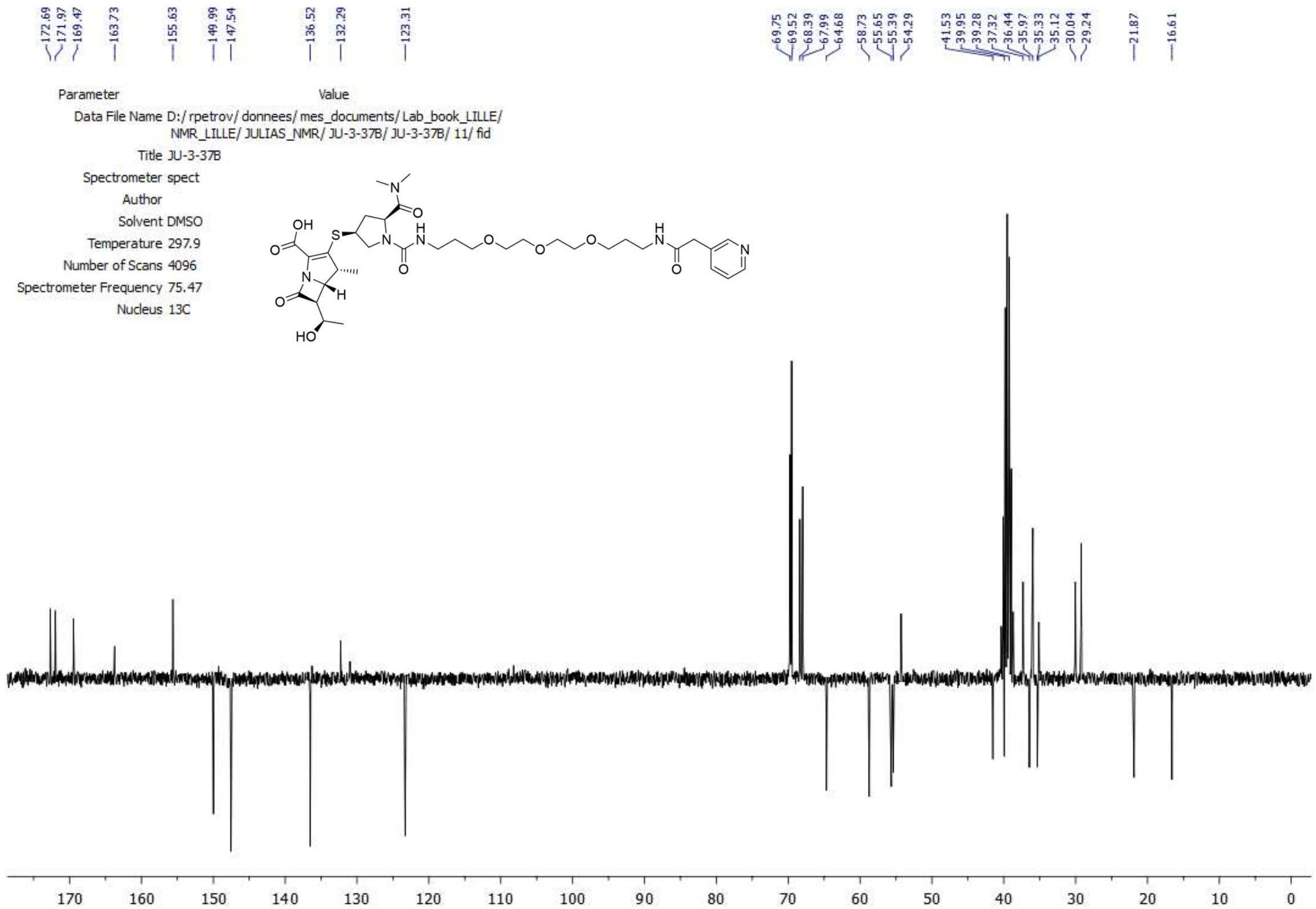

Comp. 6, ¹H/¹³C/¹⁵N chemical shifts in DMSO-d_6

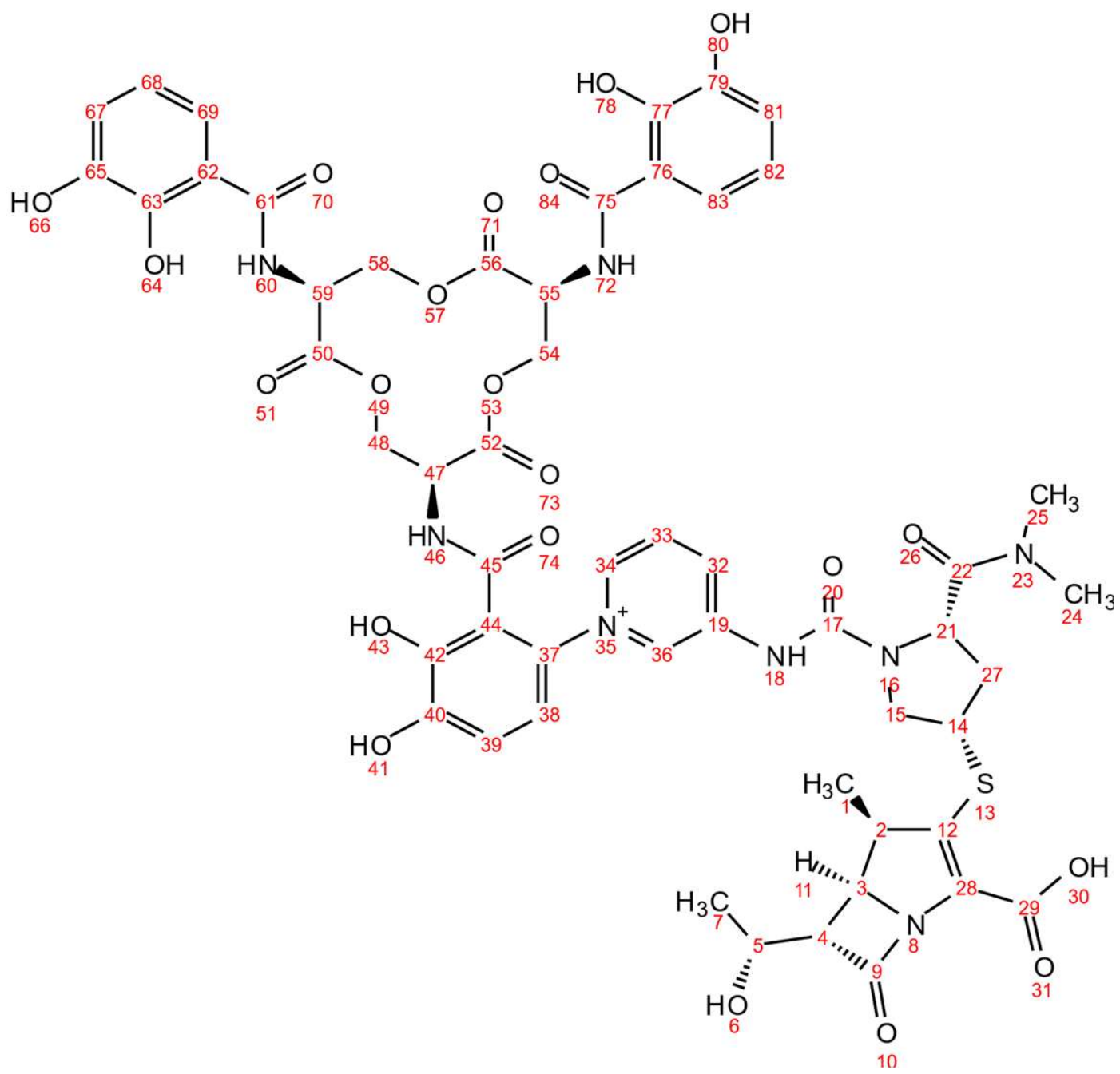

Table 1. ¹H/¹³C/¹⁵N chemical shifts assignments in DMSO-d_6 for comp. 6 at 293K and 14T referenced against the residual peak of solvent, ovl: overlap.

Atom number Comp. **6**	¹H /ppm 600 MHz	J Hz	Integral	¹³C /ppm 151 MHz	¹⁵N /ppm 61 MHz	HMBC cor. atom no.	COSY cor. atom no.	NOE cor. atom no.
1	1.15	m	ovl	17.0			2	14
2	3.60	br	1H	42.5			1/11	11/14/15’
3				55.2				
4	3.24	br	ovl	59.7			5	
5	3.96	br	1H	64.2			4/7	11
7	1.14	m	ovl	21.8			5	
9				174.1		4/11		
11	4.16	br	1H				2	2/5
12				146.1		1		
14	3.81	br	1H	39.9			15/15’/27/27’	1/2
15	3.26	br	ovl	55.1			14/15’	18
15’	4.25	br	ovl	55.1			14/15	2/18
18	9.40	br	ovl		104			15/15’/32/36
19				139.3		33		
21	4.83	br	1H	56.3			27/27’	24/25
22				170.6		24/25		
24	3.03	s	3H	36.4				21/25
25	2.80	s	ovl	35.4				21/24
27	1.55	br	1H	34.2			14/21/27’	
27’	2.78	br	ovl	34.2			14/21/27	
32	8.52	br	ovl	132.7			33	18

Atom number Comp. **6**	^{1}H /ppm	J Hz	Integral	^{13}C /ppm	^{15}N /ppm	HMBC cor. atom no.	COSY cor. atom no.	NOE cor. atom no.
	600 MHz			151 MHz	61 MHz			
33	7.92	br	1H	126.2			32/34	
34	8.58	d, 6.0	ovl	139.1			33	38
36	9.06	br	ovl	135.0				18/38
37				135.3		34/39		
38	6.35	d, 7.9	1H	109.6			39	34/36
39	6.74	d, 7.9	ovl	109.1			38	
40				152.3		38		
42				160.0		39		
46	10.07	br	1H				47	
47	5.01	br	1H	51.0			46/48/48’	
48	4.06	br	1H	65.8			47/48’	
48’	4.77	br	1H	65.8			47/48	
50				168.1		58		
52				169.2		48		
54	4.25	br	ovl	63.8			54’/55	
54’	4.67	br	ovl	63.8			54/55	
55	4.89	m	1H	51.3			54/54’/72	
56				168.9		54		
58	4.43	m	1H	65.3			58’/59	
58’	4.65	br	ovl	65.3			58/59	
59	4.95	dt, 6.4&3.4	1H	52.9			58/58’/60	
60	9.48	br	ovl					69
61				166.0		69		
62				117.6		68		
63				146.7		67/69		
65				145.7		68		
67	7.00	d, 7.6	2H	118.5			68	
68	6.71	m	ovl	118.5			67/69	
69	7.35	d, 8.0	2H	119.9			68	60
72	9.16	br	ovl				55	83
75				168.3		83		
76				115.2		82		
77				148.4		81/83		
79				146.2		82		
81	6.94	d, 7.6	2H	118.0			82	
82	6.69	m	ovl	118.4			81/83	
83	7.25	d, 7.25	2H	118.3			82	72

Comp. 7, $^{1}H/^{13}C/^{15}N$ chemical shifts in DMSO-d_6

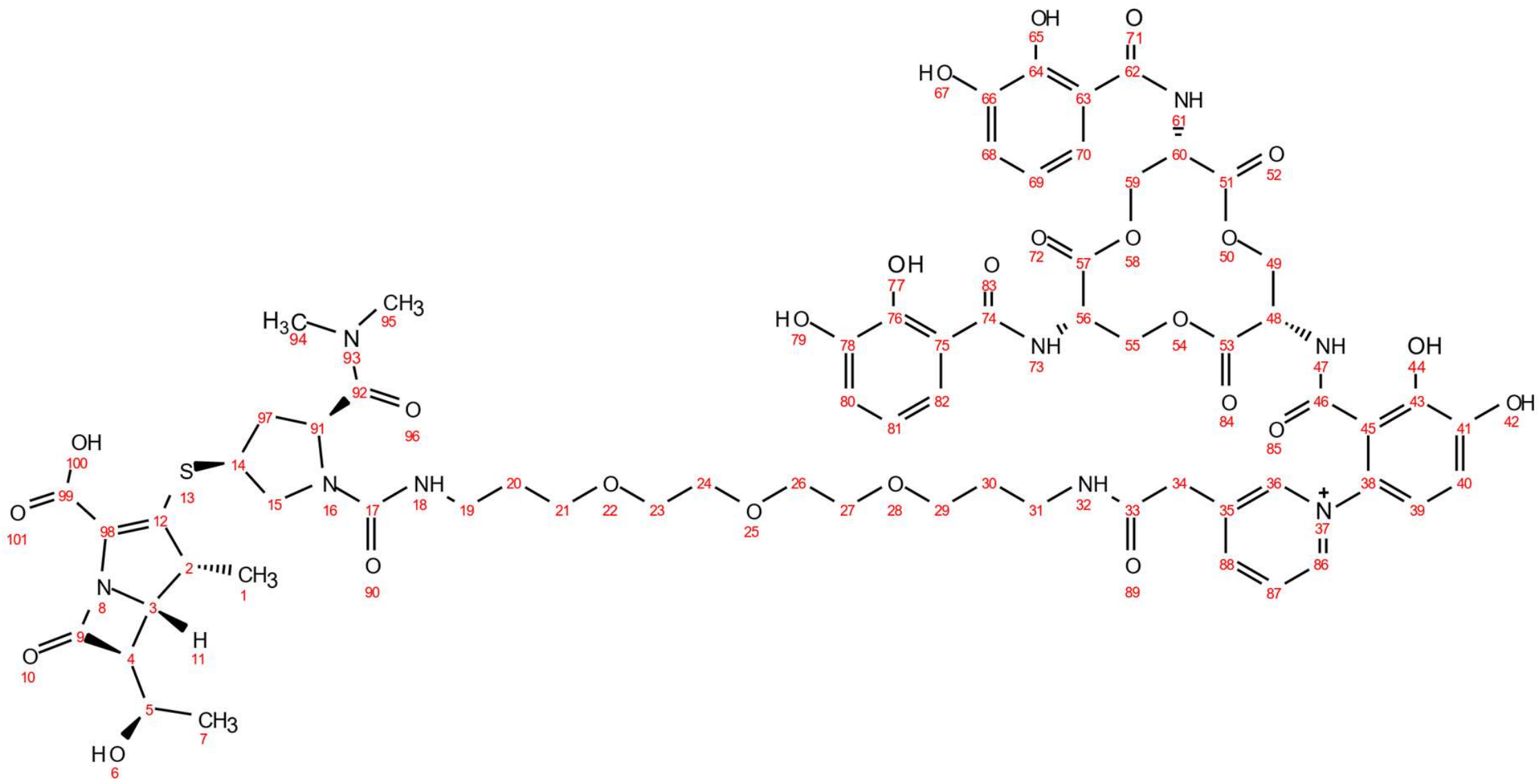

Table 2. $^{1}H/^{13}C/^{15}N$ chemical shifts assignments in DMSO-d_6 for comp. 7 at 293K and 14 T referenced against the residual peak of solvent, ovl: overlap.

Atom number (Comp. 7)	^{1}H /ppm 600 MHz	Mult., J (Hz)	Integral	^{13}C /ppm 151 MHz	^{15}N /ppm 61 MHz	HMBC cor. atom no.	COSY cor. atom no.	NOE cor. atom no.
1	1.13	d, 7.5	ovl	17.0			2	4/91
2	3.49	m	1H	42.7			1/11	11/15’
3/11	4.14	dd, 2.2&9.5	1H	55.3			2/4	2
4	3.22	dd, 2.5&6.2	1H	59.6			5/11	1
5	3.95	m	ovl	64.2			4/7	
7	1.14	d, 6.4	ovl	21.8			5	
8					157	2/4/11		
9				173.9		11/4/5		
12				147.0		1/2/14		
14	3.73	m	ovl	39.9			15/15’97/97’	1/91
15	3.08	t, 9.6	1H	54.3			14	
15’	3.93	m	ovl	54.3			14	2/18
16					94	97		
17				155.6		19/91		
18	6.21	t, 4.9	1H		84	20	19	15’
19	3.00	m	ovl	37.3			18/20	
20	1.59	m	ovl	30.1			19/21	
21	3.37	m	ovl	68.3			20	
23/27	3.45	m	ovl	69.5			24/26	
24/26	3.49	m	ovl	69.8			23/27	
29	3.38	m	ovl	67.9			30	
30	1.63	m	ovl	29.2			29/31	

Atom number (Comp. **7**)	^{1}H /ppm 600 MHz	Mult., J (Hz)	Integral	^{13}C /ppm 151 MHz	^{15}N /ppm 61 MHz	HMBC cor. atom no.	COSY cor. atom no.	NOE cor. atom no.
31	3.11	m	ovl	36.2			30/32	
32	8.17	br	1H		118	30/31	31	34
33				167.8		31/32/34		
34	3.73	s	ovl	37.9				32/36/88
35				135.3		34/87		
36	8.93	s	1H	145.8				34
37					219	36/39/86/87		
38				134.8		36/40/86		
39	6.33	d, 7.9	1H	110.1			40	36/86
40	6.76	d, 7.9	1H	109.1			39	
41				152.4		39		
43				160.0		40		
45				108.7		39		
46				165.6		39		
47	12.4	br	1H		114			
48	5.00	br	1H	51.1			49/49’	
49	4.06	d, 10	1H	65.8			48/49’	
49’	4.77	d, 10	1H	65.8			48/49	
51				168.1		49/49’/59/59’/60		
53				169.1		48/49/49’/55/55’		
55	4.27	d, 10.9	1H	63.8			55’/56	
55’	4.68	m	ovl	63.8			55/56	
56	4.89	dt, 3.4&7.8	1H	51.3			55/55’/73	
57				168.9		55/55’/56/59/59’		
59	4.44	d, 11.5	1H	65.2			59’/60	
59’	4.64	d, 11.5	1H	65.2			59/60	
60	4.95	td, 3.4&6.4	1H	52.9			59/59’/61	
61	9.15	br	1H		112		60	70
62				168.4		70		
63				115.7		69		
64				148.3		68/70		
66				146.1		69		
68	6.95	d, 7.6	1H	119.1			69/70	
69	6.70	t, 7.8	ovl	118.5			68/70	
70	7.26	d, 8.0	1H	118.3			68/69	61
73	9.44	br	1H		111		56	82
74				166.0		82		
75				117.5		81		
76				146.6		80/82		
78				145.7		81		
80	7.01	d, 7.7	1H	118.6			81/82	

Atom number (Comp. **7**)	^{1}H /ppm 600 MHz	Mult., J (Hz)	Integral	^{13}C /ppm 151 MHz	^{15}N /ppm 61 MHz	HMBC cor. atom no.	COSY cor. atom no.	NOE cor. atom no.
81	6.72	t, 7.9	ovl	118.6			80/82	
82	7.35	d, 8.1	1H	119.9			80/81	73
86	8.91	d, 6.0	1H	144.6			87	39
87	7.98	t, 7.0	1H	125.6			86/88	
88	8.47	d, 7.9	1H	145.9			87	34
91	4.69	m	ovl	55.5			97/97’	14/15’/94/97’
92				171.8		91/94/95/97		
93					96	94/95		
94	3.01	s	ovl	36.5				91/95/97’
95	2.78	s	3H	35.3				94
97	1.48	ddd, 7.6&10.5&12.3	1H	34.8			14/91/97’	
97’	2.69	td, 8.0&12.3	1H	34.8			14/91/97	91/94
98				125.9		2		
99				162.0				

Comp. 8, $^{1}H/^{13}C/^{15}N$ chemical shifts in DMSO-d_6

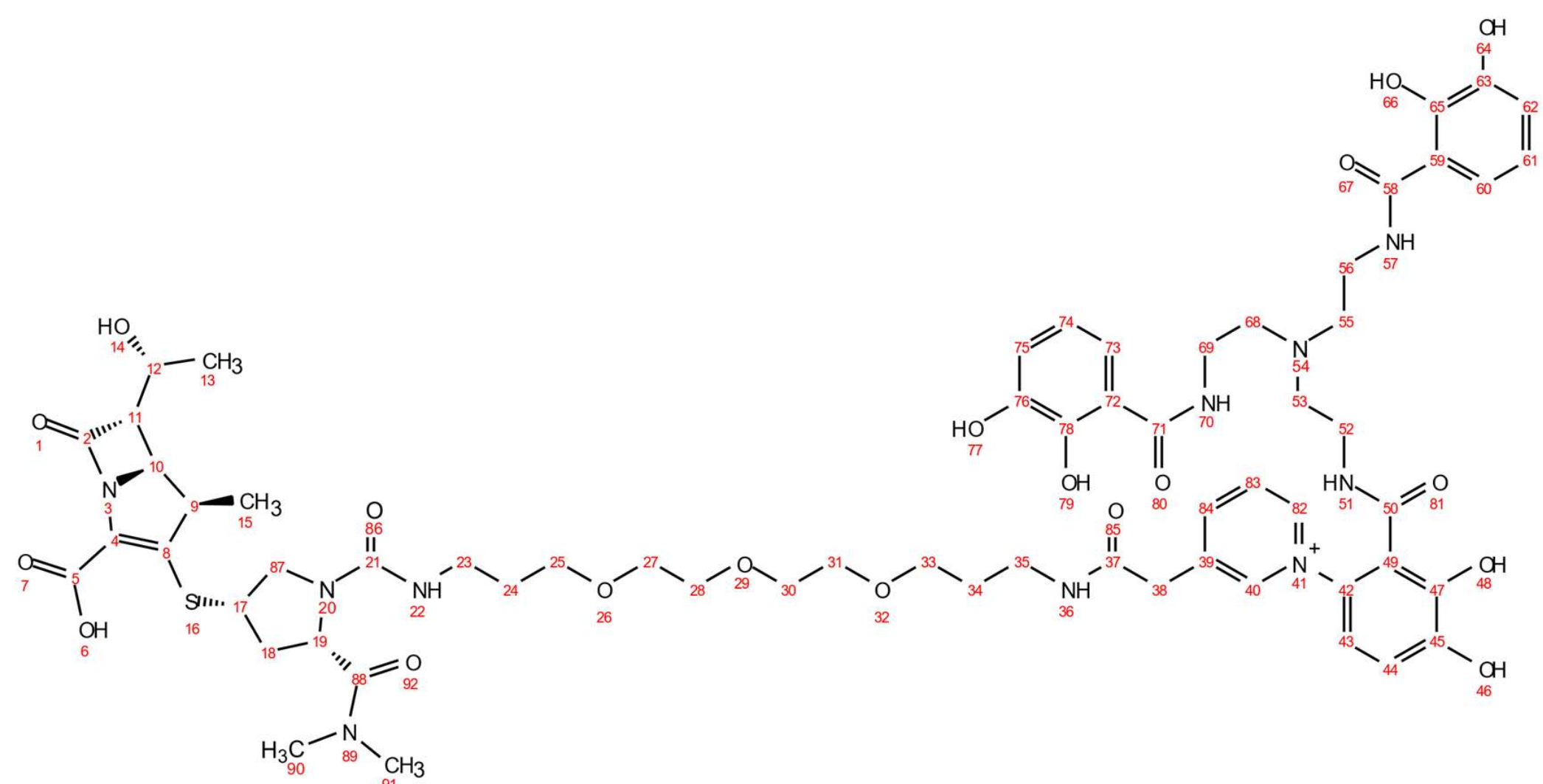

Table 3. $^{1}H/^{13}C/^{15}N$ chemical shifts assignments in DMSO-d_6 for comp. 8 at 293K and 14 T referenced against the residual peak of solvent. ovl: overlap.

Atom number (comp. **8**)	^{1}H /ppm 600 MHz	Mult., J (Hz)	Integral	^{13}C /ppm 151 MHz	^{15}N /ppm 61 MHz	HMBC cor. atom no.	COSY cor. atom no.	NOE cor. atom no.
3					158	10/11		
9	3.48	m	ovl	42.7			10,15	17/87
10	4.14	dd, 2.6&9.5	1H	55.3			9,11	12
11	3.21	m	ovl	59.5			10,12	13/15

Atom number (comp. **8**)	^{1}H /ppm 600 MHz	Mult., J (Hz)	Integral	^{13}C /ppm 151 MHz	^{15}N /ppm 61 MHz	HMBC cor. atom no.	COSY cor. atom no.	NOE cor. atom no.
12	3.95	m, 6.3	ovl	64.2			13,11	10
13	1.14	d,	3H	21.8			12	11
15	1.12	d,	3H	17.0			9	11/17
17	3.72	m	ovl	39.9			18,18’,87,87’	9/15/19
18	1.49	m	1H	34.7			17,18’,19	87
18’	2.68	m	1H	34.7			17,18,19	
19	4.69	t, 7.8	1H	55.5			18,18’	17/22/90/91
22	6.21	t, 5.4	1H		84		23	19/87/87’/90
23	3.00	m	ovl	37.3			22,24	
24	1.59	m	1H	30.1			23,25	
25	3.37	m	ovl	68.3			24	
27/31	3.45	m	ovl	69.5				
28/30	3.49	m	ovl	69.8				
33	3.38	m	ovl	67.9			34	
34	1.63	m	1H	29.2			33,35	
35	3.12	m	ovl	36.2			34,36	
36	8.15	t, 5.3	1H		118		35	38
38	3.73	s	ovl	39.9				36/40/84
40	8.88	s	1H	145.6				38/43
41					220			
43	6.26	d, 7.8	1H	109.3			44	40/82
44	6.70	d, 7.8	1H	108.5			43	
54					31	56/69		
55/68	2.65	m	4H	52.9			56/69	
56/69	3.33	m	ovl	37.5			55/57/69/70	
57/70	8.93	t, 5.3	2H		112		56/69	60/73
60/73	7.29	dd, 1.4&8.2	2H	117.3			61/62/74/75	57/70
61/74	6.52	t, 8.0	2H	117.9			60/62/73/75	
62/75	6.87	dd, 1.4&7.8	2H	118.7			60/61/73/74	
82	8.82	d, 6.0	1H	144.5			83	43
83	7.97	dd, 6.0&8.0	1H	125.4			82/84	
84	8.46	d, 8.0	1H	145.7			83	38
87	3.08	t, 9.6	1H	54.3			17,87’	9/18/22
87’	3.94	m	ovl	54.3			17,87	22
					96	90/91		
90	3.00	s	ovl	36.4				19/22/91
91	2.78	s	3H	35.4				19/90

Comp. 9, ^{1}H NMR in DMSO-d_6, Bruker-300

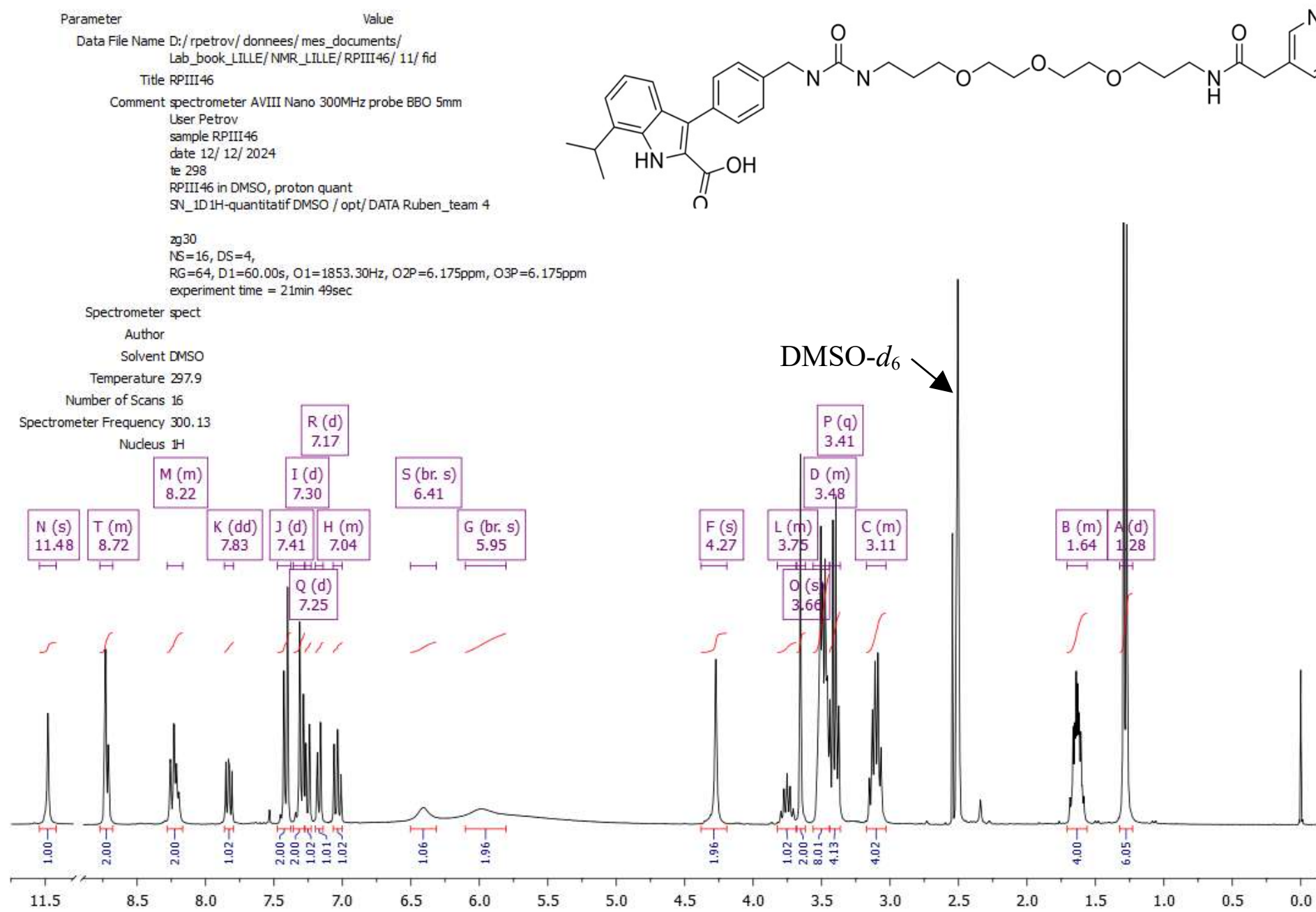

Comp. 9, ^{13}C NMR in DMSO-d_6, Bruker-300

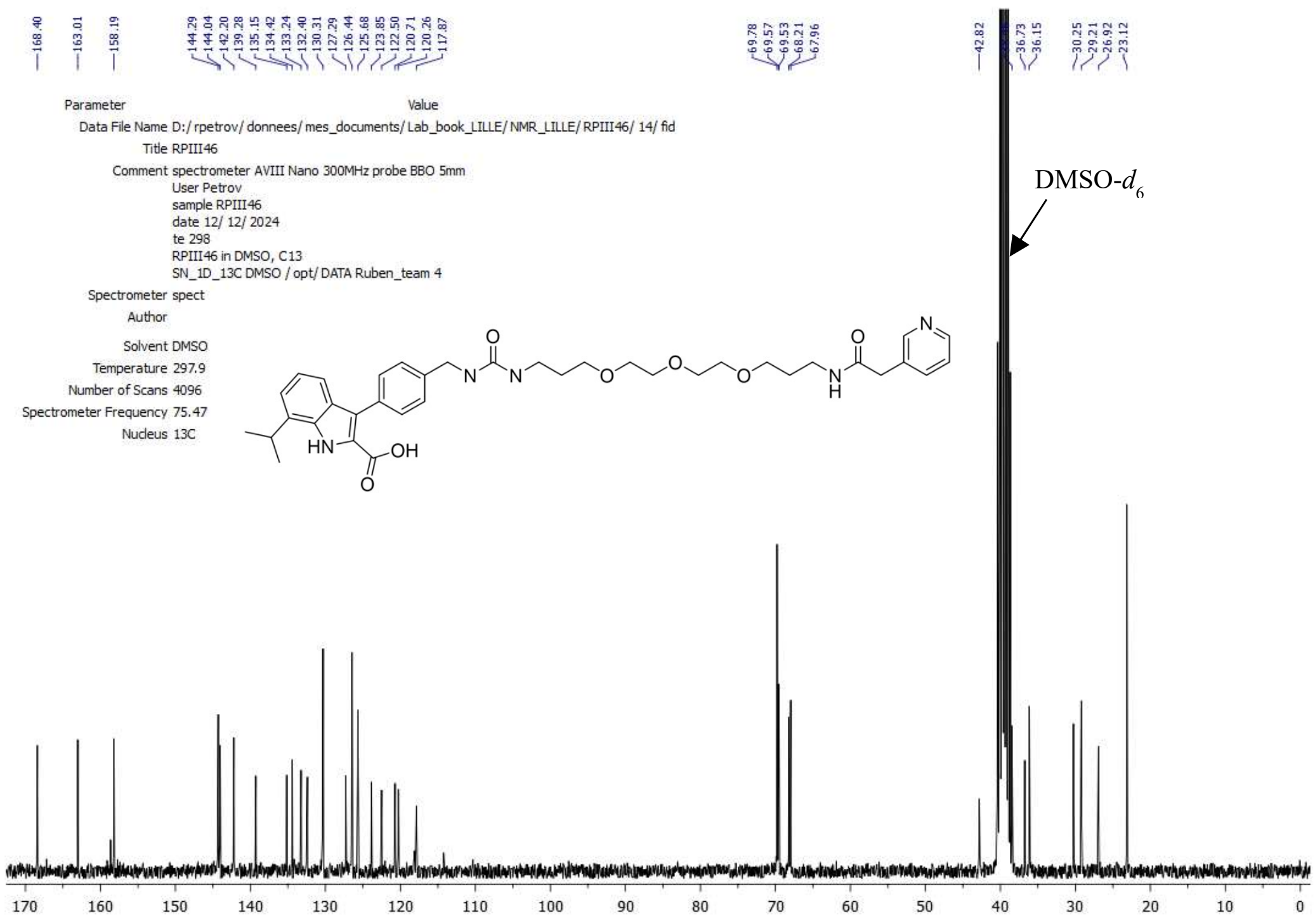

Comp. 11, $^{1}H/^{13}C/^{15}N$ chemical shifts in DMSO-d_6

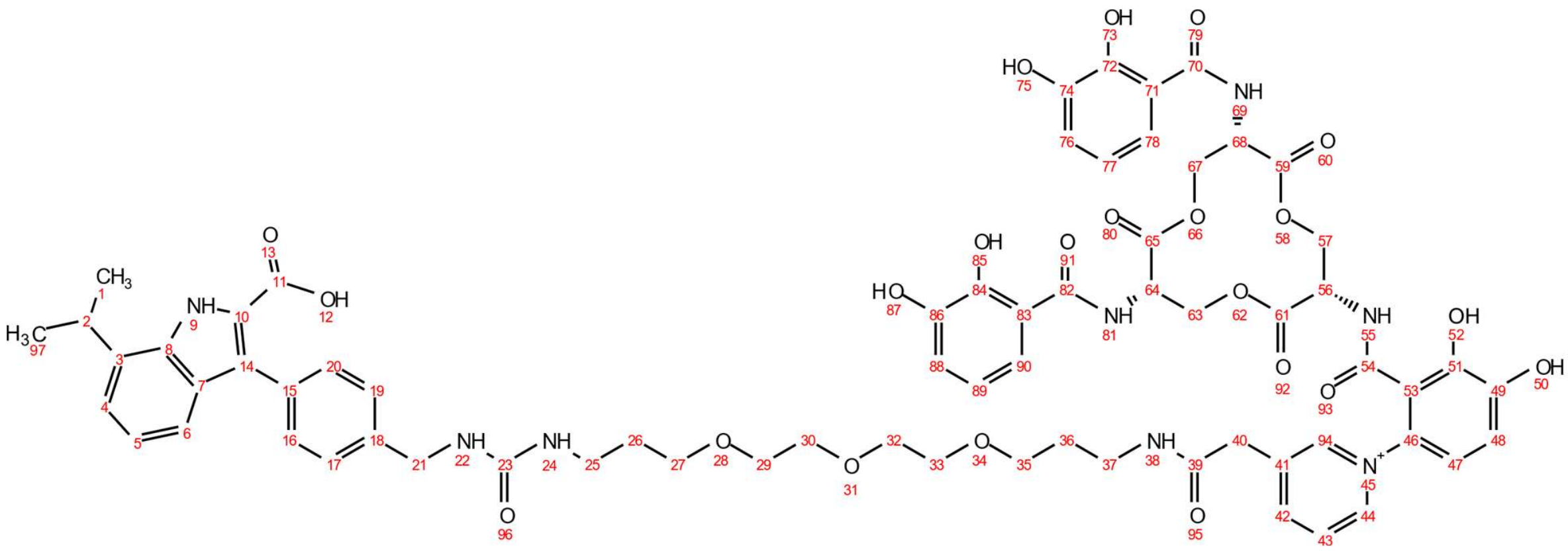

Table 4. $^{1}H/^{13}C/^{15}N$ chemical shifts assignments in DMSO-d_6 for comp. 11 at 293K and 14 T referenced against the residual peak of solvent; ovl: overlap.

Atom number (Comp. 11)	^{1}H /ppm 600 MHz	Mult., J (Hz)	Integral	^{13}C /ppm 151 MHz	^{15}N /ppm 61 MHz	HMBC cor. atom no.	COSY cor. atom no.	NOE cor. atom no.
1/97	1.27	d, 6.8	6H	23.2			2	4
2	3.74	7plet, 6.8	ovl	26.9			1/97	9
3				133.2		1/5/97		
4	7.16	d, 7.1	1H	120.3			5	1/97
5	7.02	t, 7.6	1H	120.7			4/6	
6	7.24	d, 8.1	1H	117.9			5	
7				127.3		5/9		
8				134.4		2/4/6/9		
9	11.49	s	1H		132	4		2
10				123.9		9		
11				163.1		9		
14				122.5		6/9/16/20		
15				132.4		17/19		
16/20	7.40	d, 8.0	2H	130.3			17/19	
17/19	7.29	d, 8.0	2H	126.5			16/20	21
18				139.3		16/20/21		
21	4.26	d, 5.7	ovl	42.8			22	17/19
22	6.37	t, 5.7	1H		83	21	21	24
23				158.2		21/22/24/25		
24	5.96	t, 6.6	1H		81	25/26	25	22
25	3.07	q, 6.3	2H	36.7			24/26	
26	1.62	5plet, 6.7	ovl	30.2			25/27	
27	3.40	t, 6.4	2H	68.2		29	26	
29/33	3.45	m	4H	69.5		27/35	30/32	
30/32	3.49	m	4H	69.8			29/33	
35	3.38	t, 6.2	2H	67.9		33	36	

Atom number (Comp. 11)	^{1}H /ppm 600 MHz	Mult., J (Hz)	Integral	^{13}C /ppm 151 MHz	^{15}N /ppm 61 MHz	HMBC cor. atom no.	COSY cor. atom no.	NOE cor. atom no.
36	1.64	5plet, 6.7	ovl	29.2			35/37	
37	3.11	q, 6.1	2H	36.2			36/38	
38	8.16	t, 4.5	1H		118	37/38	37	40
39				167.8		37/38/40		
40	3.72	s	ovl	37.9				38/42/94
41				135.3		40/42/43/94		
42	8.47	d, 8.1	1H	145.9			43	40
43	7.98	t, 7.0	1H	125.6			42/44	
44	8.90	d, 6.0	1H	144.6			43	47
45					218	42/44/47/94		
46				134.8		44/48/94		
47	6.33	d, 6.9	1H	110 .1			48	44/94
48	6.76	d, 7.9	1H	109.2			47	
49				152.4		47		
51				160.0		48		
53				109.2		47		
54				165.6				
55	12.4	br	1H					
56	5.00	br	1H	51.1			57/57’	
57	4.06	d, 10.6	1H	65.8			56/57’	
57’	4.77	d, 9.3	1H	65.8			56/57	
59				168.1		57/57’/67/67’		
61				169.1		57/57’/63/63’		
63	4.26	m	ovl	63.8			63’/64	
63’	4.69	dd, 8.3&10.8	1H	63.8			63/64	
64	4.90	dt, 3.5&7.6	1H	51.3			63/63’/81	
65				168.9		63/63’/67/67’		
67	4.45	dd, 3.5&11.2	1H	65.2			67’/68	
67’	4.64	d, 10.6	1H	65.2			67/68	
68	4.96	td, 3.5&6.3	1H	52.9			67/67’/69	
69	9.42	d, 4.6	1H		111		68	78
70				166.0		69/78		
71				117.5		77		
72				146.6		76/78		
74				145.7		77		
76	7.01	d, 7.9	1H	119.1			77/78	
77	6.73	t, 7.9	1H	118.6			76/78	
78	7.35	dd, 1.2&8.0	1H	119.9			76/77	69

Atom number (Comp. 11)	^{1}H /ppm 600 MHz	Mult., J (Hz)	Integral	^{13}C /ppm 151 MHz	^{15}N /ppm 61 MHz	HMBC cor. atom no.	COSY cor. atom no.	NOE cor. atom no.
81	9.13	d, 7.1	1H		112		64	90
82				168.4		81/90		
83				115.6		89		
84				148.3		88/90		
86				146.1		89		
88	6.94	d, 7.8	1H	119.1			89/90	
89	6.70	t, 7.9	1H	118.6			88/90	
90	7.26	d, 8.1	1H	7.26			88/89	81
94	8.92	s	1H	145.8				40/47

Comp. 10, ^{1}H/^{13}C/^{15}N chemical shifts in DMSO-d_6

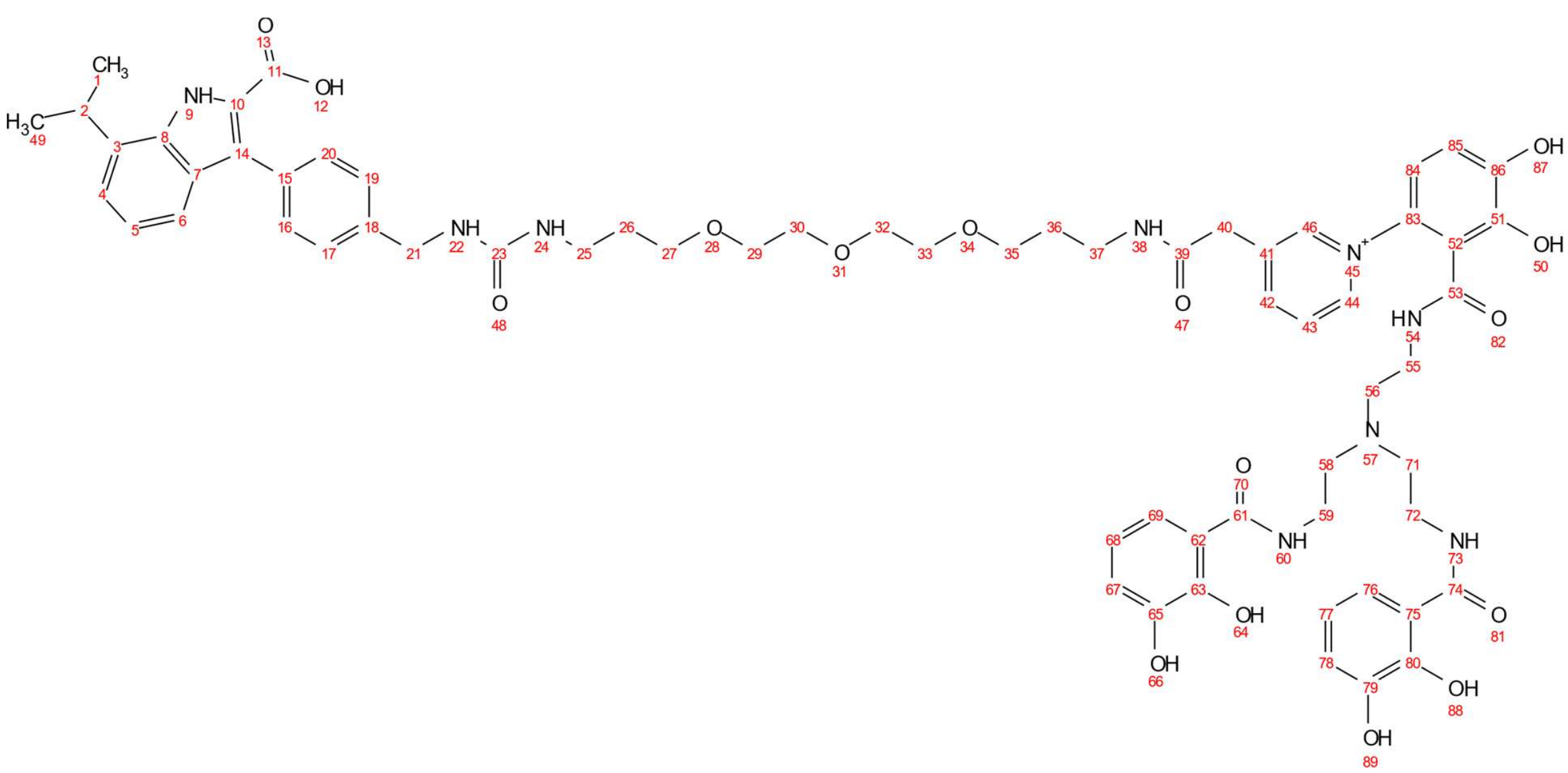

Table 5. ^{1}H/^{13}C/^{15}N chemical shifts assignments in DMSO-d_6 for comp. 10 at 293K and 14T referenced against the residual peak of solvent. ovl: overlap.

Atom number Comp. **10**	^{1}H /ppm 600 MHz	Mult., J (Hz)	Integral	^{13}C /ppm 151 MHz	^{15}N /ppm 61 MHz	HMBC cor. atom no.	COSY cor. atom no.	NOE cor. atom no.
1/49	1.27	d, 6.9	6H	23.2			2	4
2	3.75	7plet, 6.9	1H	26.9			1/49	9
3				133.3		1/5/9/49		
4	7.16	d, 7.1	1H	120.3			5	1/49
5	7.03	t, 7.6	1H	120.7			4/6	
6	7.24	d, 8.1	1H	117.8			5	
7				127.3		5/9		
8				134.5		4/6		

Atom number Comp. **10**	^{1}H /ppm 600 MHz	Mult., J (Hz)	Integral	^{13}C /ppm 151 MHz	^{15}N /ppm 61 MHz	HMBC cor. atom no.	COSY cor. atom no.	NOE cor. atom no.
9	11.51	s	1H		133			2
10				123.9		9		
11				163.1		9		
14				122.5		6/9		
15				132.2		17/19		
16/20	7.40	d, 8.2	2H	130.3			17/19	
17/19	7.29	d, 8.2	2H	126.5			16/20	21
18				139.3		16/20		
21	4.25	d, 4.2	2H	42.8			22	17/19
22	6.42	br	1H		83		21	
23				158.2		21/25		
24	6.00	br	1H		82		25	
25	3.07	t, 6.4	2H	36.7			24/26	
26	1.61	m	ovl	30.3			25/27	
27	3.39	m	ovl	68.2			26	
29/33	3.43	m	ovl	69.6				
30/32	3.48	m	ovl	69.8				
35	3.39	m	ovl	67.9			36	
36	1.62	m	ovl	29.2			35/37	
37	3.11	t, 6.4	ovl	36.3			36/38	
38	8.30	t, 5.3	1H		119		37	40
39				167.7		38/40		
40	3.79	s	2H	37.8				38/42/46
41				136.6		40/43		
42	8.58	d, 8.0	1H	147.5			43	40
43	8.13	dd, 6.1&8.0	1H	126.7			42/44	
44	8.91	d, 8.0	ovl	144.3			43	84/85
45					Xxx	43/84/85		
46	8.96	s	1H	145.9				40/84/85
51				143.9		84/85		
52				120.2		84/85		
52				163.3				
58/71	3.40	br	ovl	51.4			59/72	
59/72	3.62	br	4H	33.9			58/60/71/73	
60/73	8.91	br	ovl		109		59/72	69/76
61/74				170.0		60/69/73/76		
62/75				115.4		68/77		
63/80				149.0		67/69/76/78		
65/79				146.2		68/77		

Atom number Comp. **10**	^{1}H /ppm 600 MHz	Mult., J (Hz)	Integral	^{13}C /ppm 151 MHz	^{15}N /ppm 61 MHz	HMBC cor. atom no.	COSY cor. atom no.	NOE cor. atom no.
67/78	6.94	dd, 1.4&7.8	2H	119.0			68/69/76/77	
68/77	6.69	t, 8.0	2H	118.2			67/69/76/78	
69/76	7.21	dd, 1.4&8.2	2H	117.7			67/68/77/78	60/73
83				132.2		44/46/84/85		
84	7.07	m	ovl	117.4				
85	7.07	m	ovl	115.3				
86				148.5		84/85		

Comp. 16, ¹H NMR in DMSO-d_6, Bruker-300

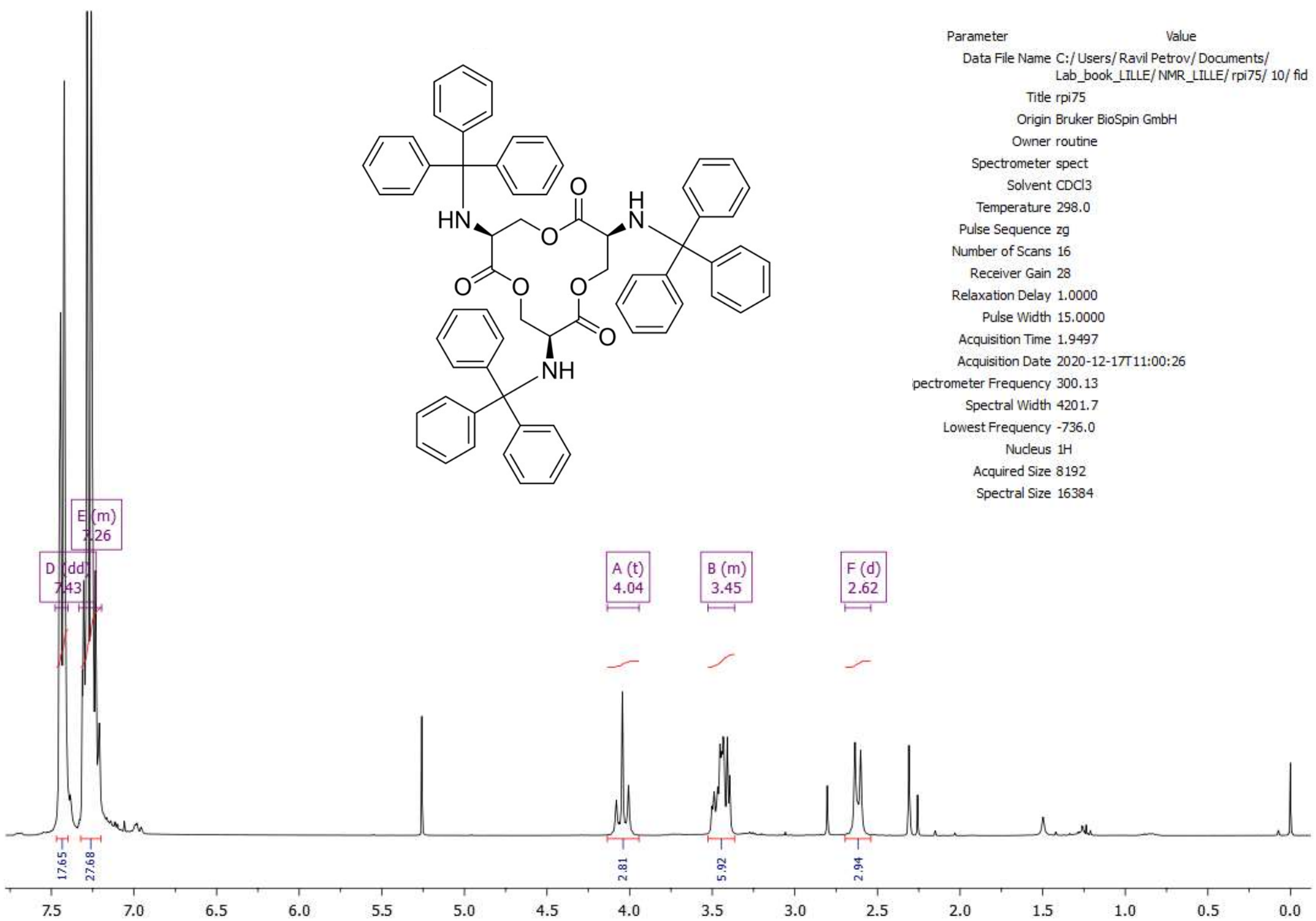

Comp. 16, ^{13}C NMR in DMSO-d_6, Bruker-300

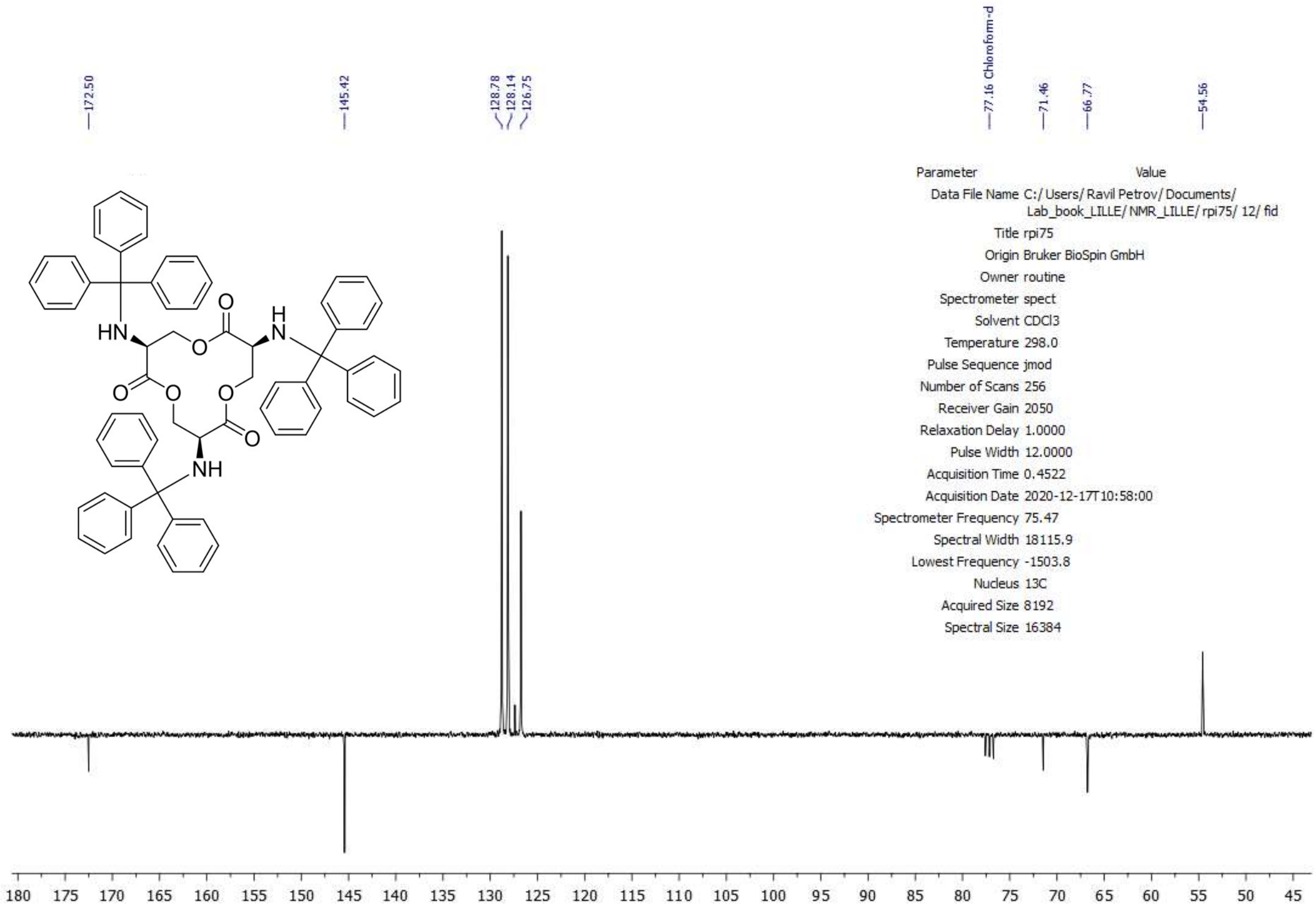

Comp. 18, ^{1}H NMR in $CDCl_3$, Bruker-300

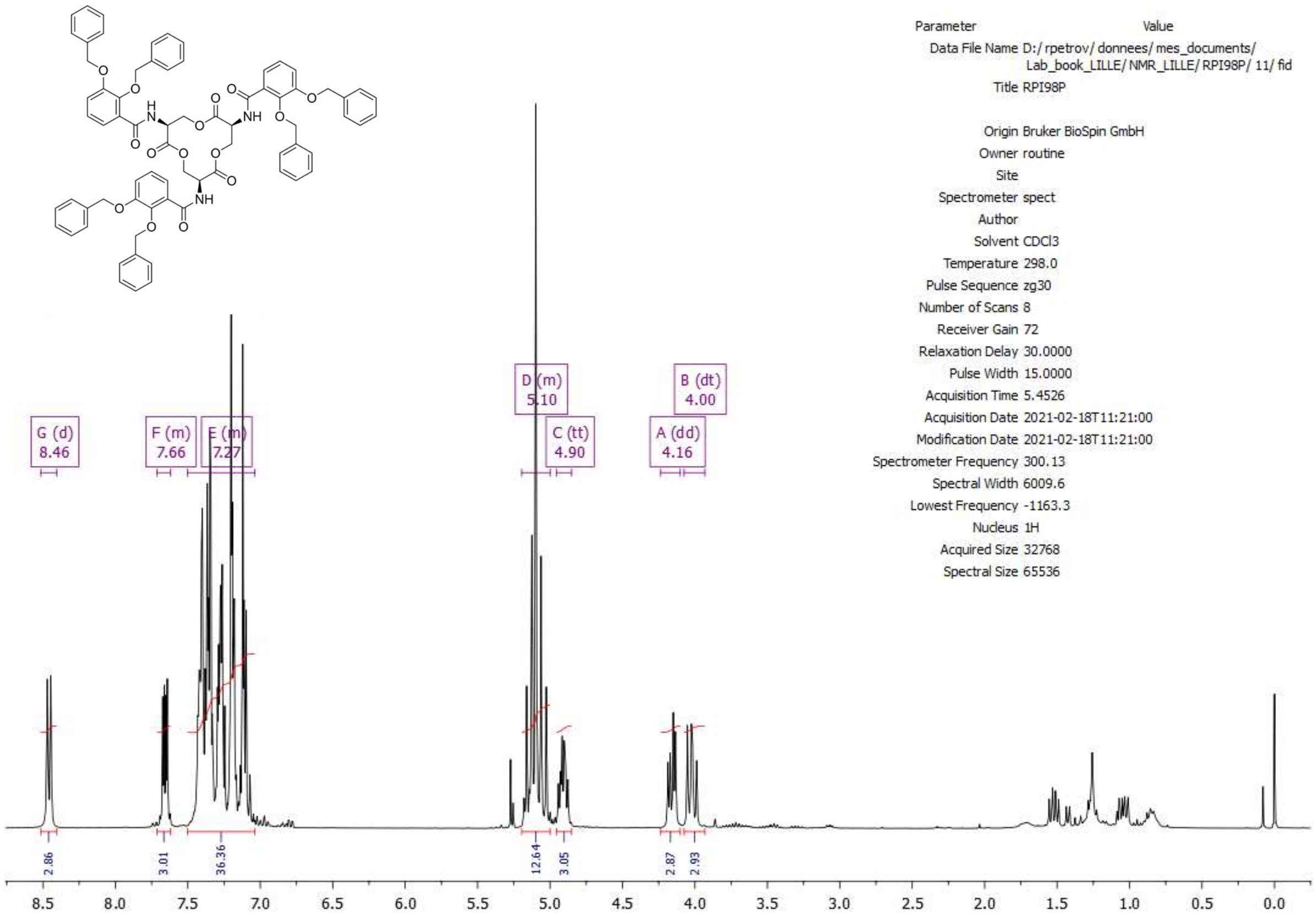

Comp. 18, ^{13}C NMR in $CDCl_3$, Bruker-300

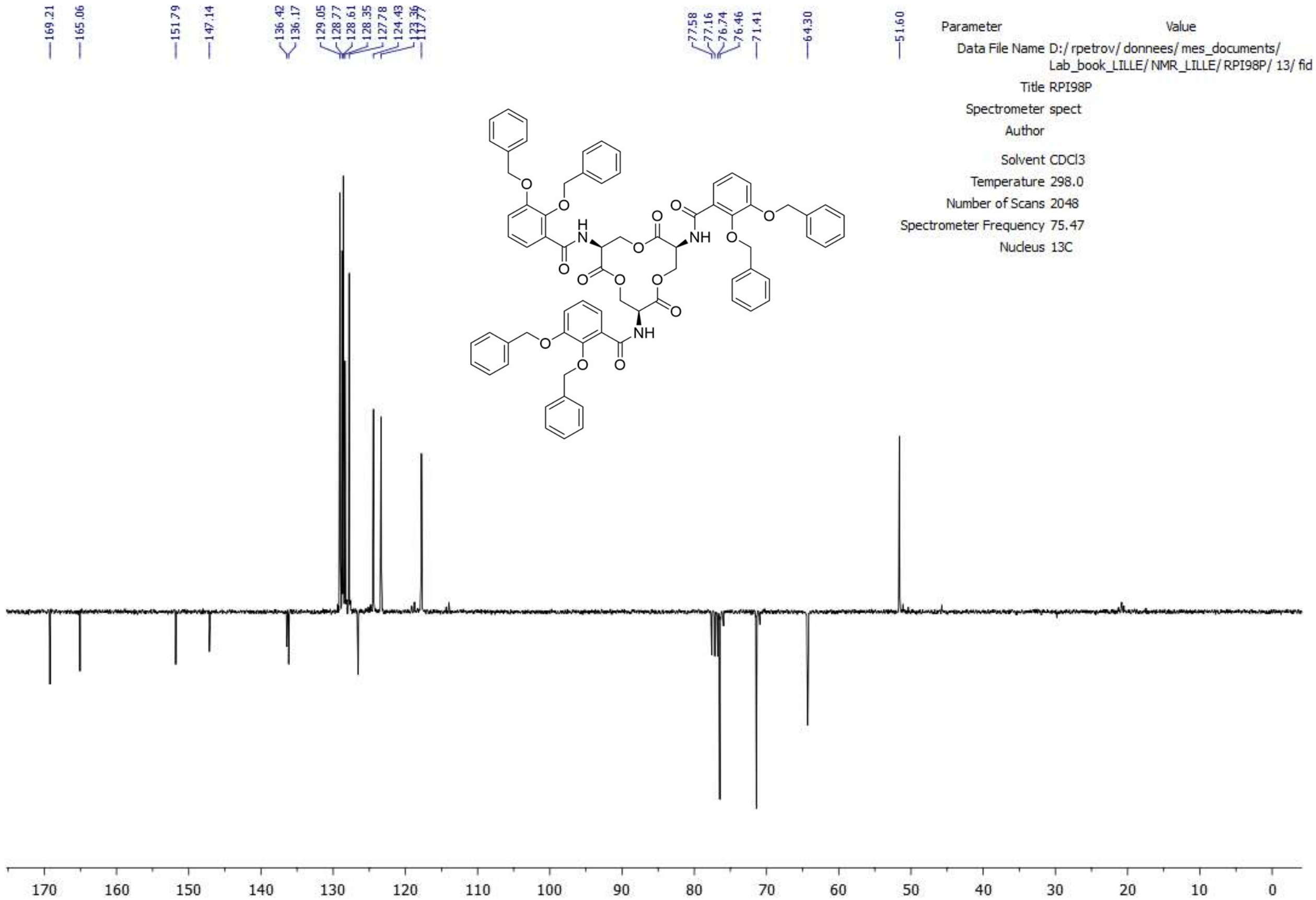

Comp. 20, ^{1}H NMR in $CDCl_3$, Bruker-300

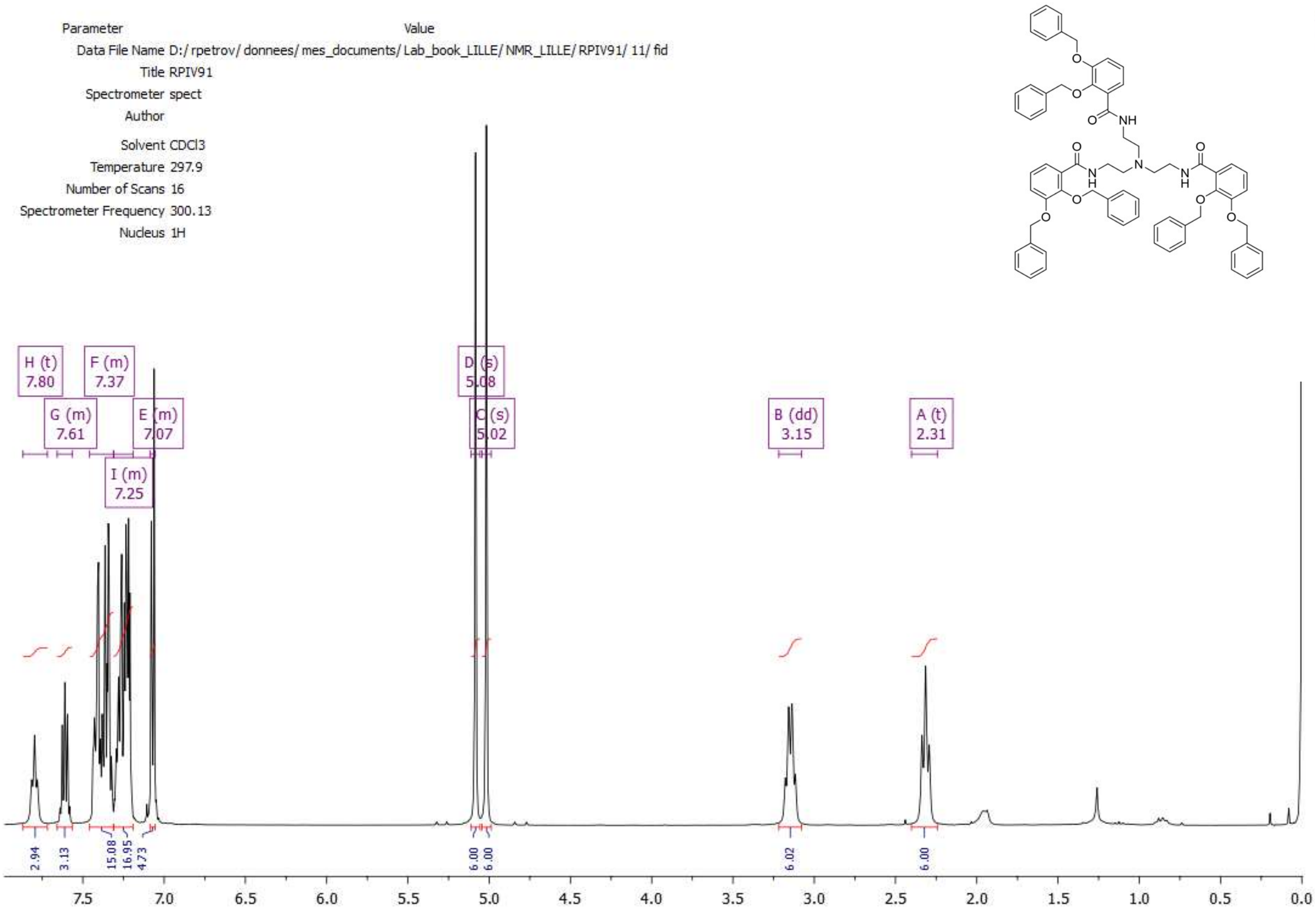

Comp. 20, ^{13}C NMR in $CDCl_3$, Bruker-300

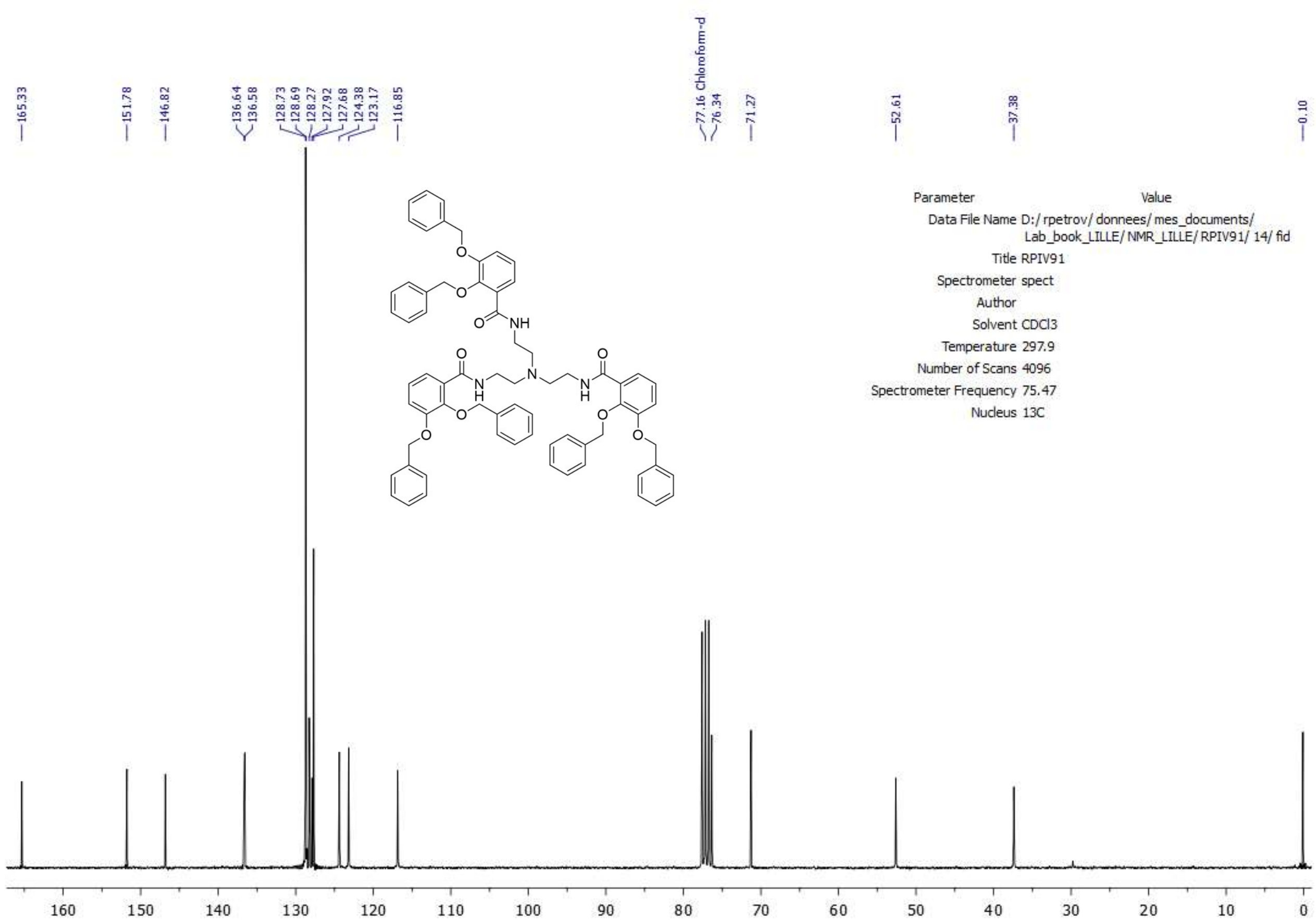

Comp. 21, ^{1}H NMR in DMSO-d_6, Bruker-300

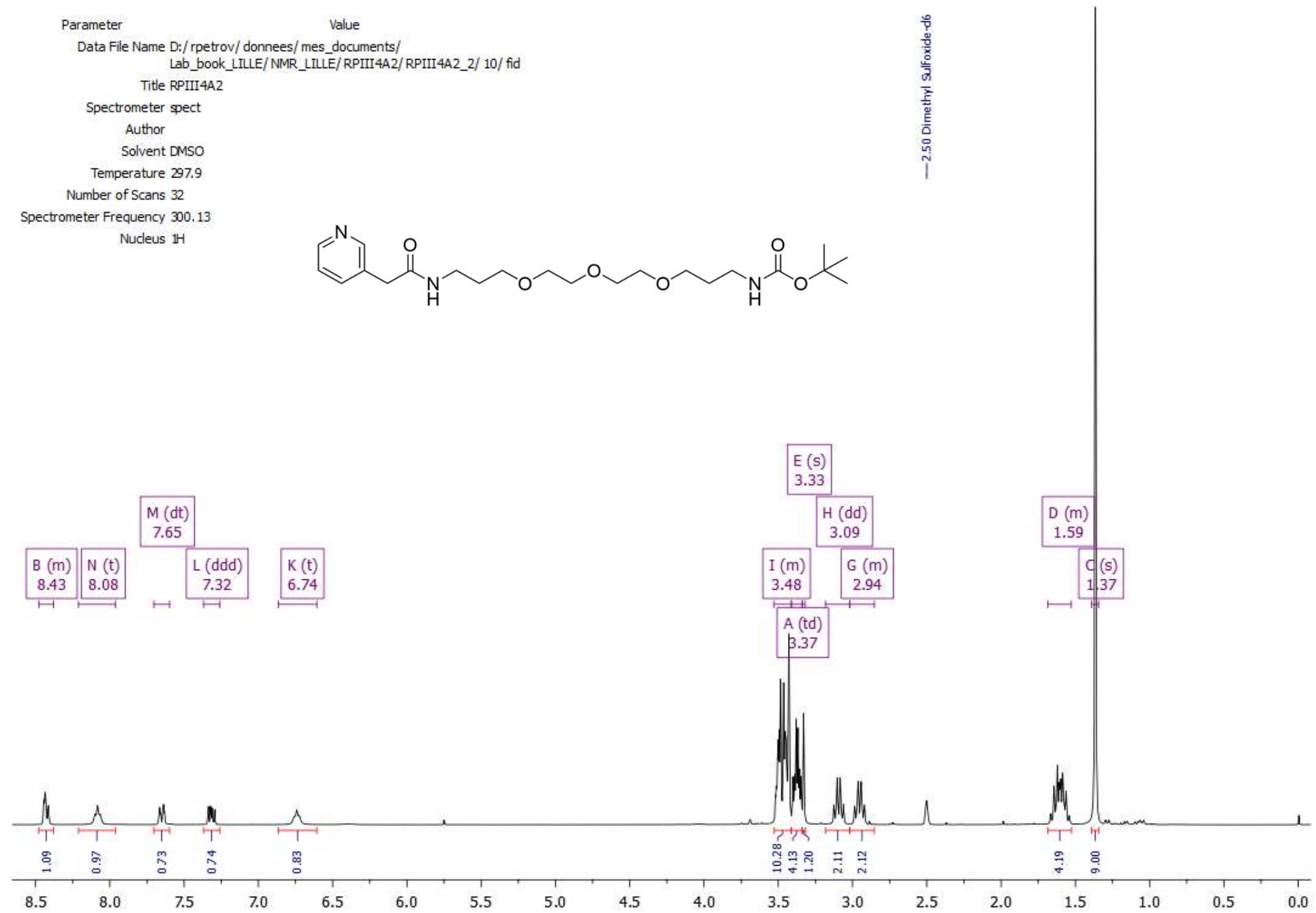

Comp. 21, ^{1}H NMR in $CDCl_3$, Bruker-300

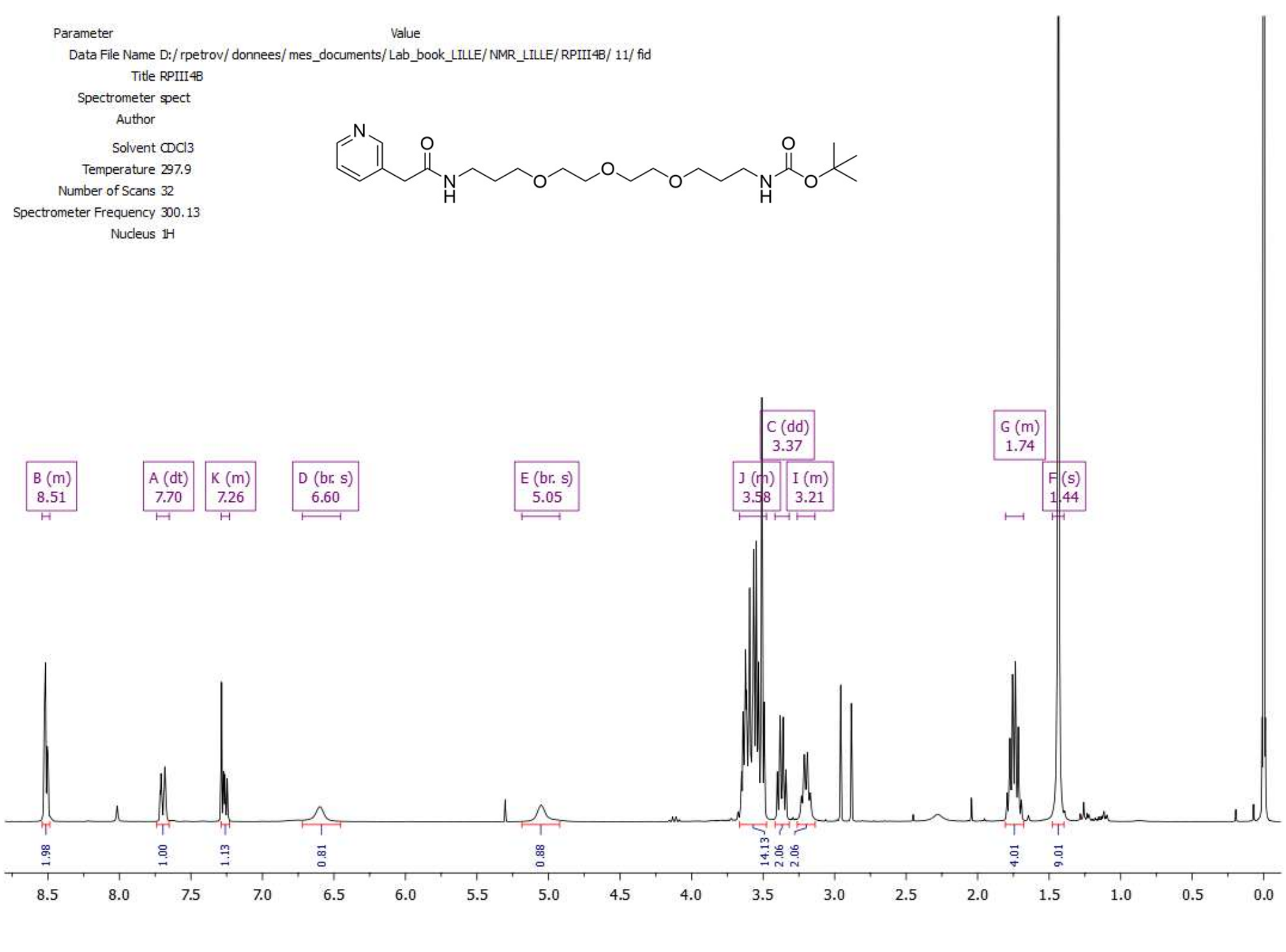

Comp. 21, ^{13}C NMR in $CDCl_3$, Bruker-300

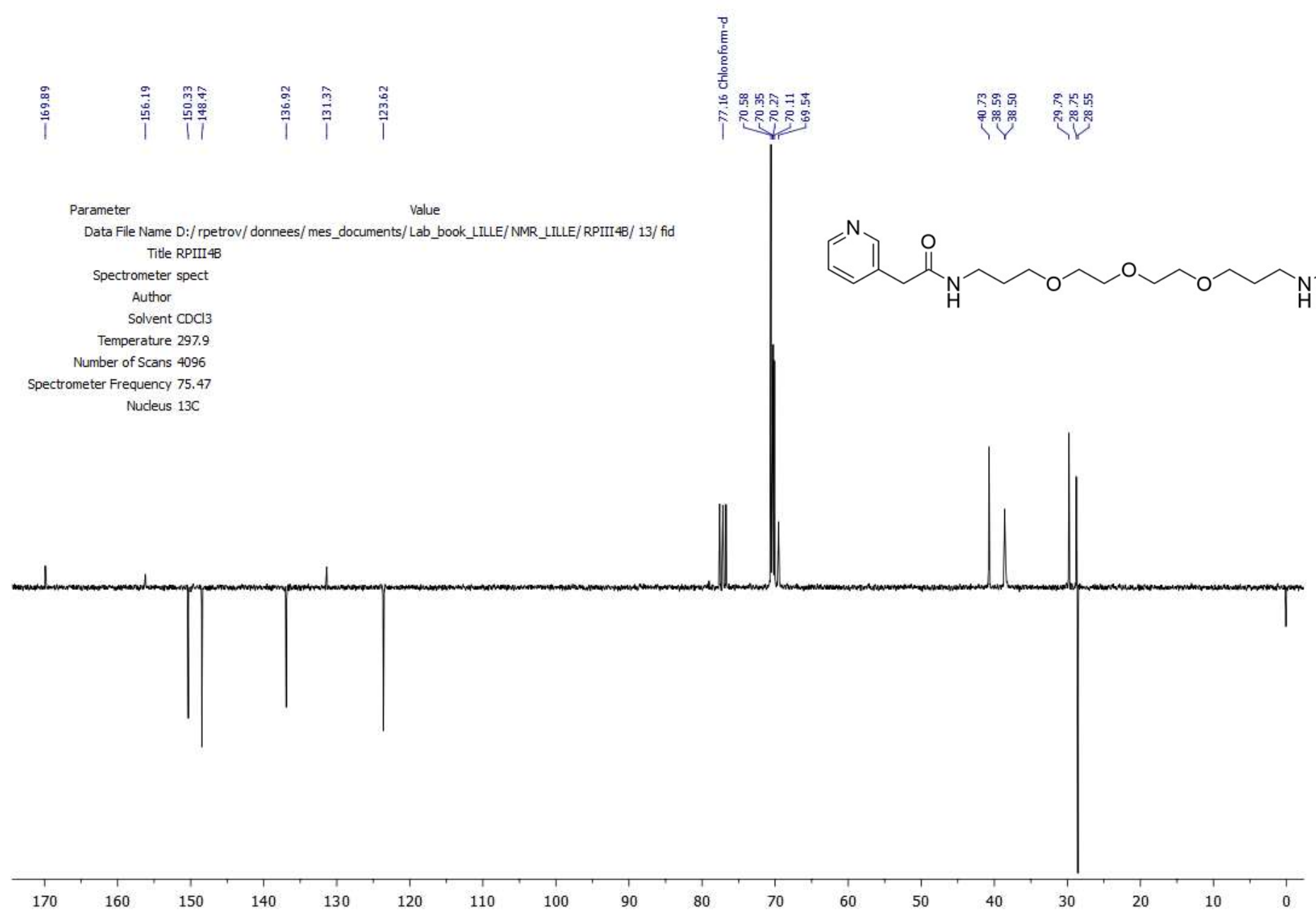

Comp. 22, ^{1}H NMR in $CDCl_3$, Bruker-300

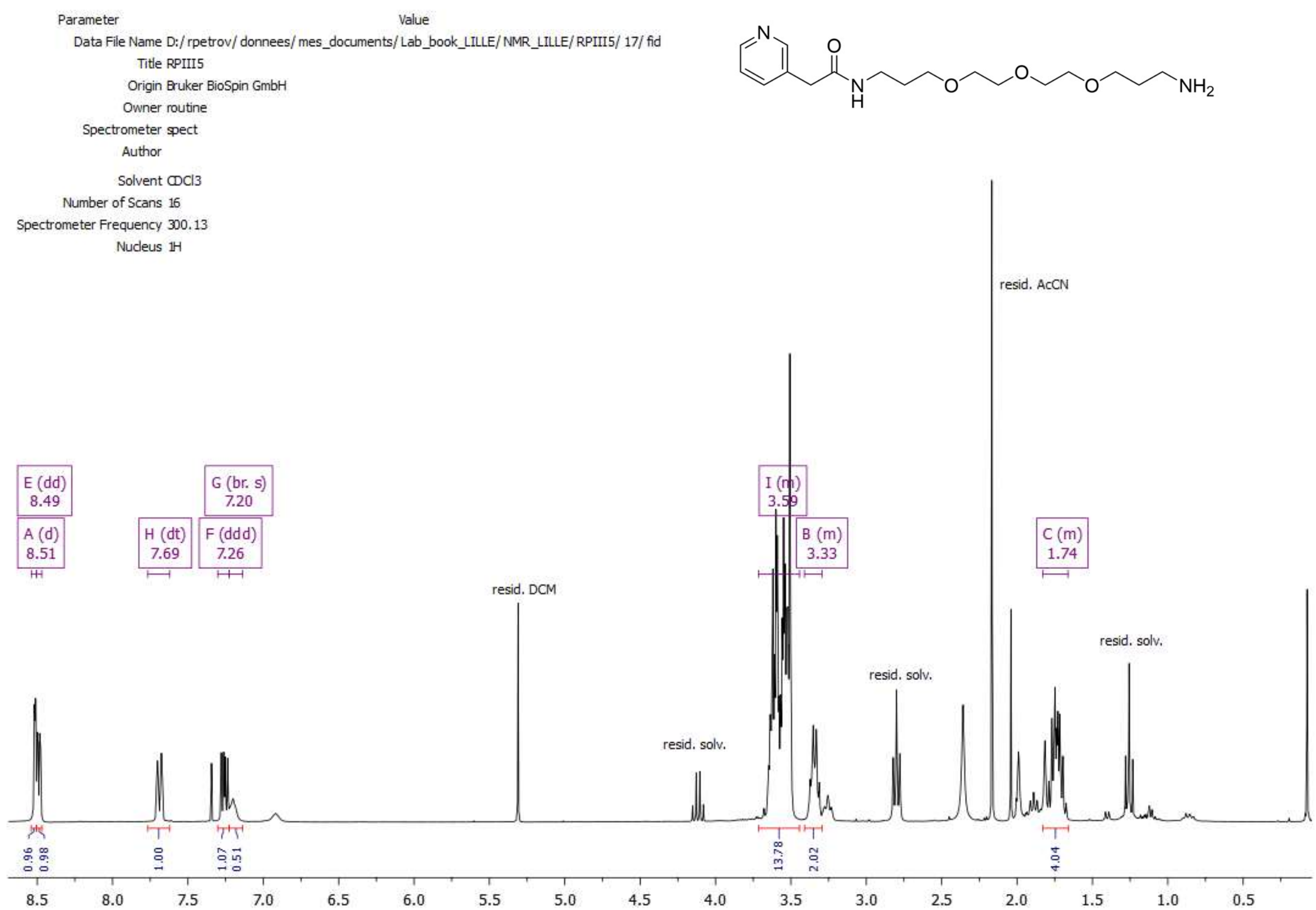

Comp. 22, ^{13}C NMR of in $CDCl_3$, Bruker-300

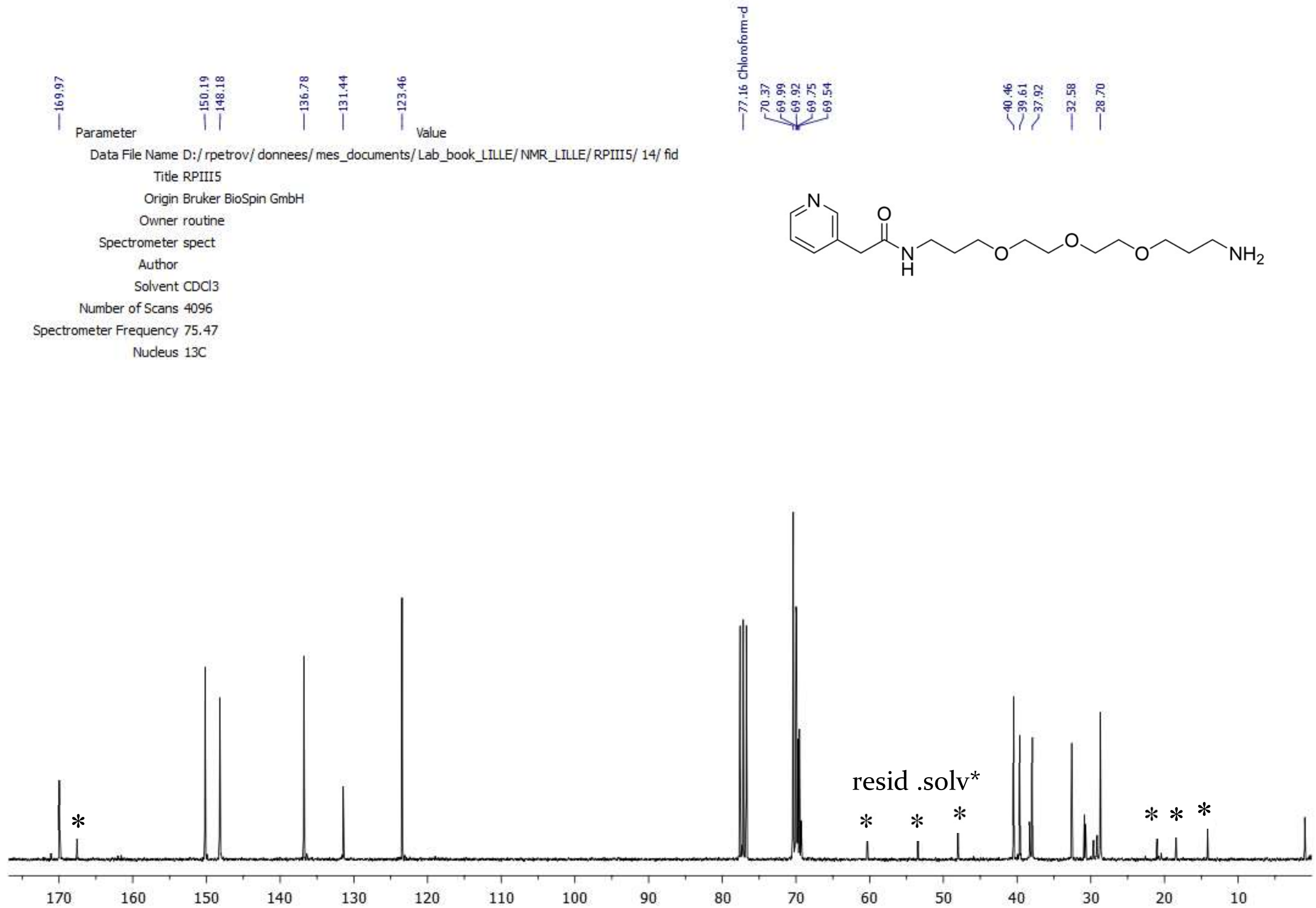

Comp. 25, ^{1}H NMR in DMSO-d_6, Bruker-300

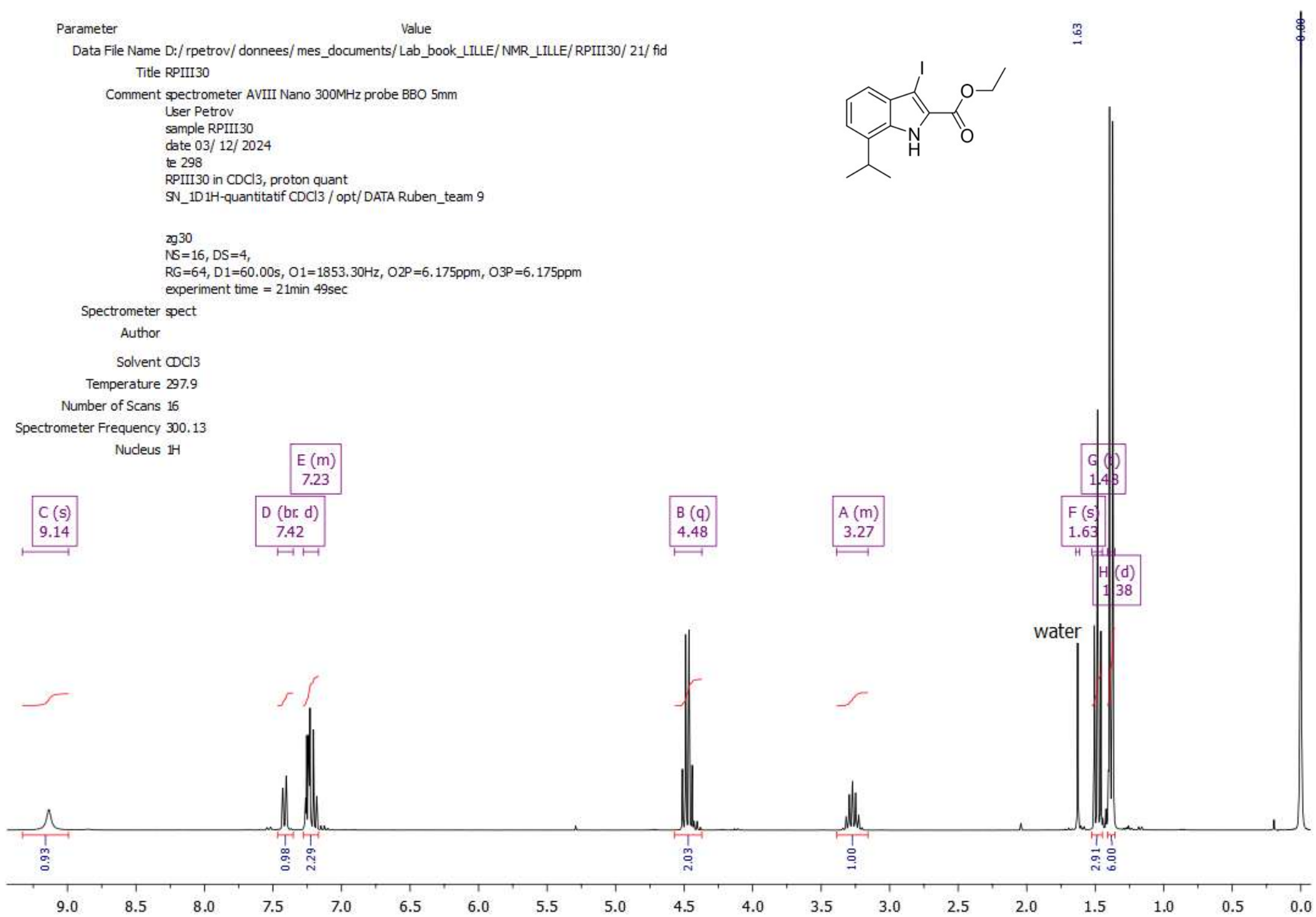

Comp. 25, ^{13}C NMR in DMSO-d_6, Bruker-300

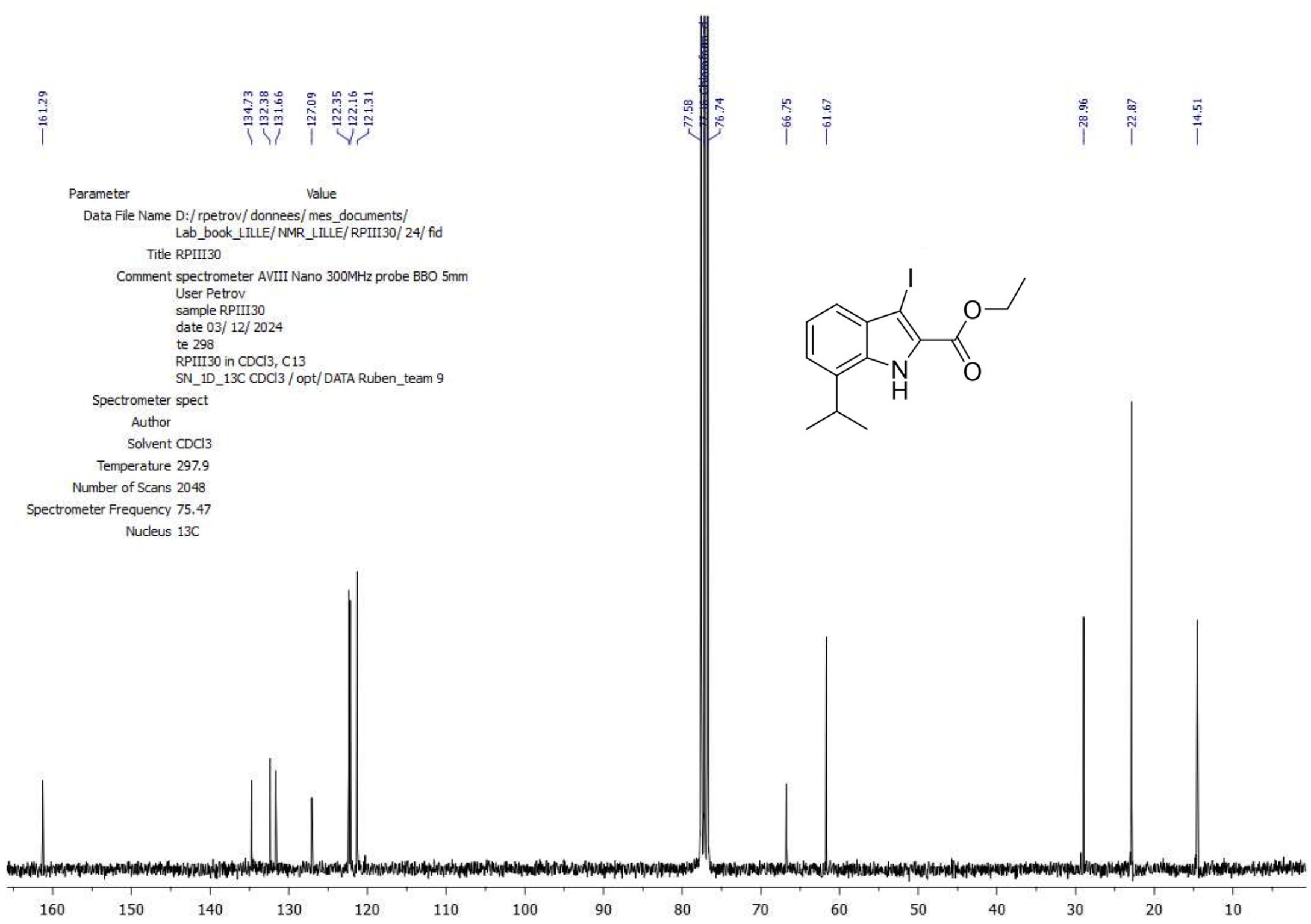

Comp. 27, ^{1}H NMR in DMSO-d_6, Bruker-300

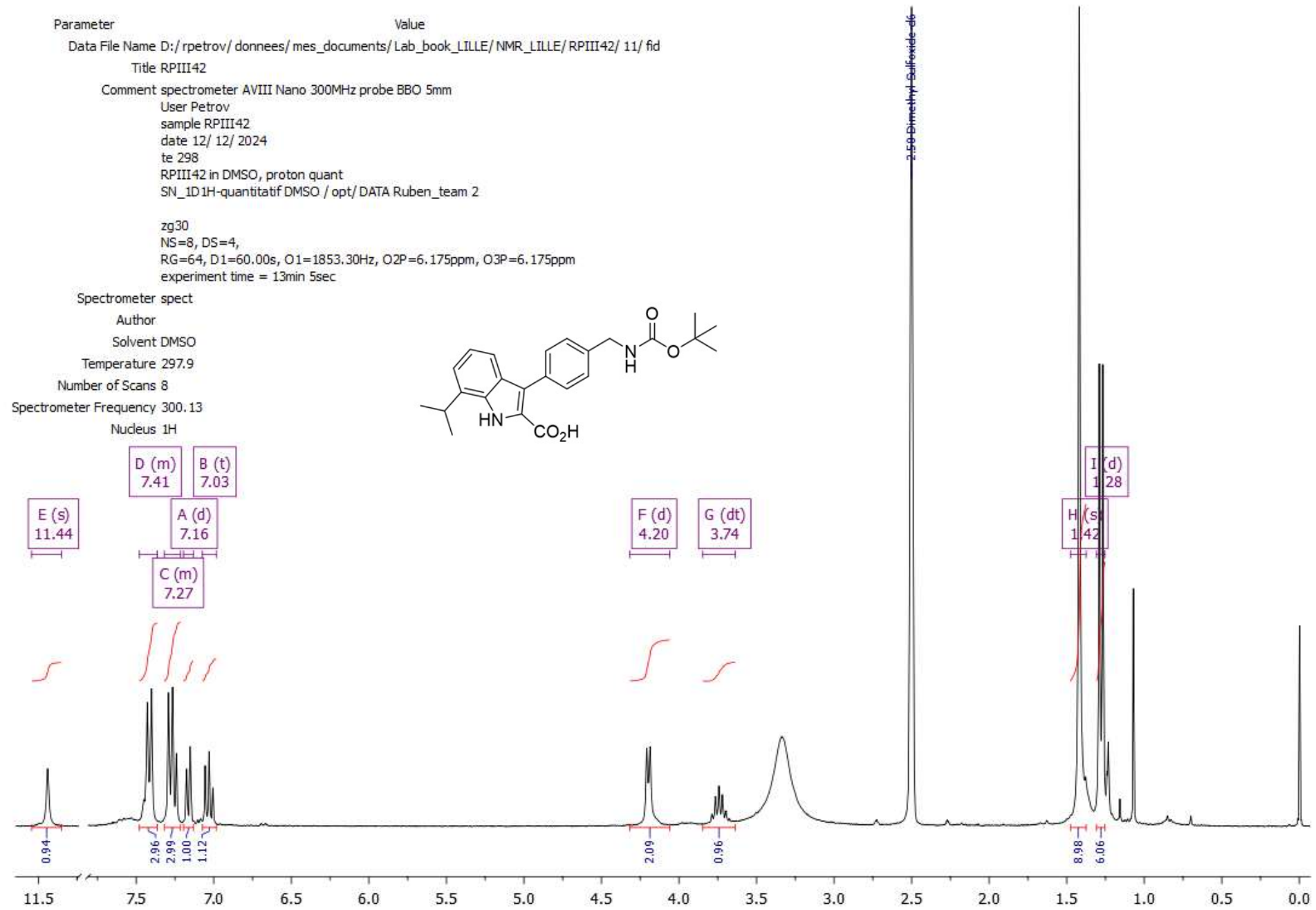

Comp. 27, ^{13}C NMR in DMSO-d_6, Bruker-300

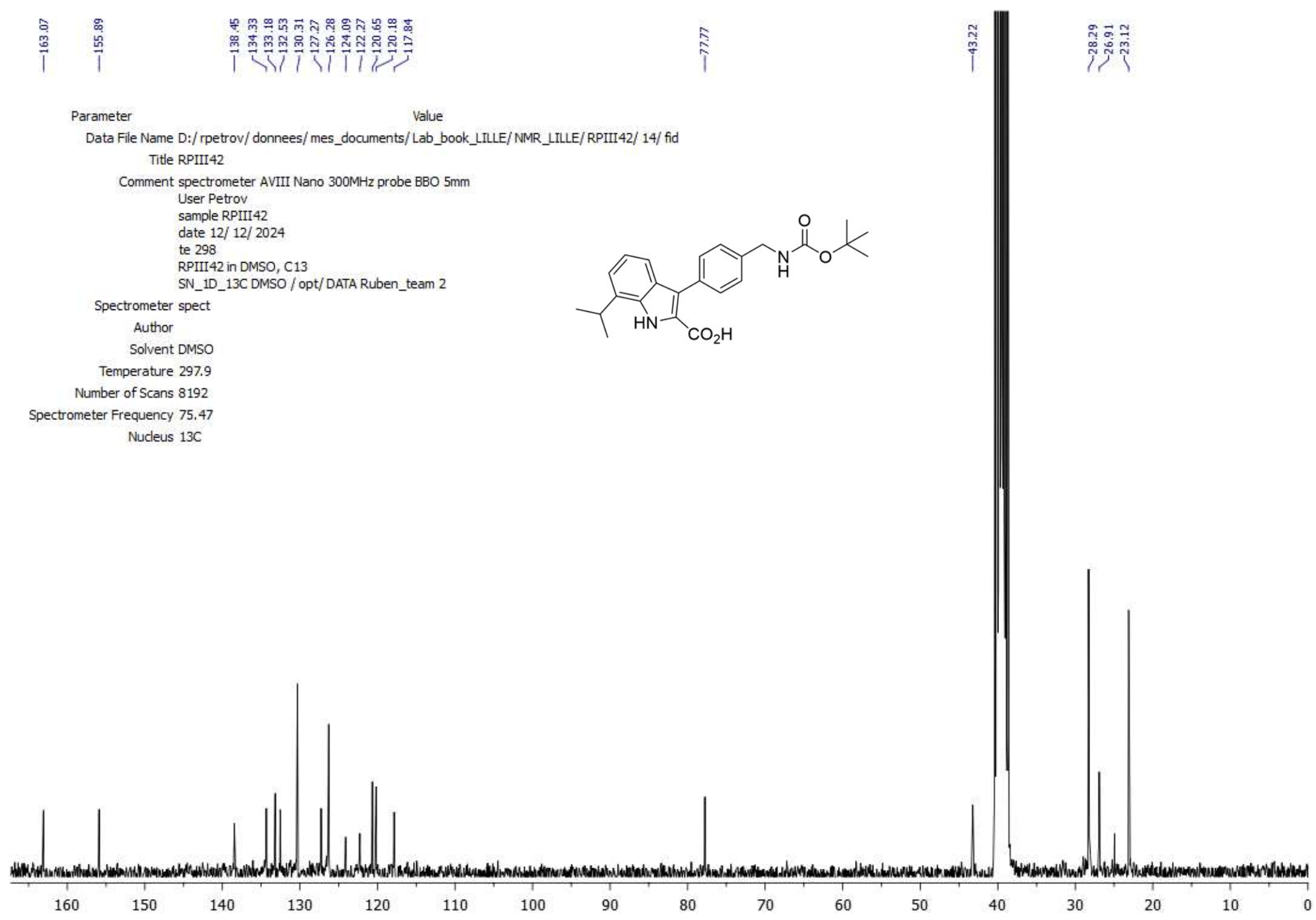

Comp. 28, ^{1}H NMR in DMSO-d_6, Bruker-300

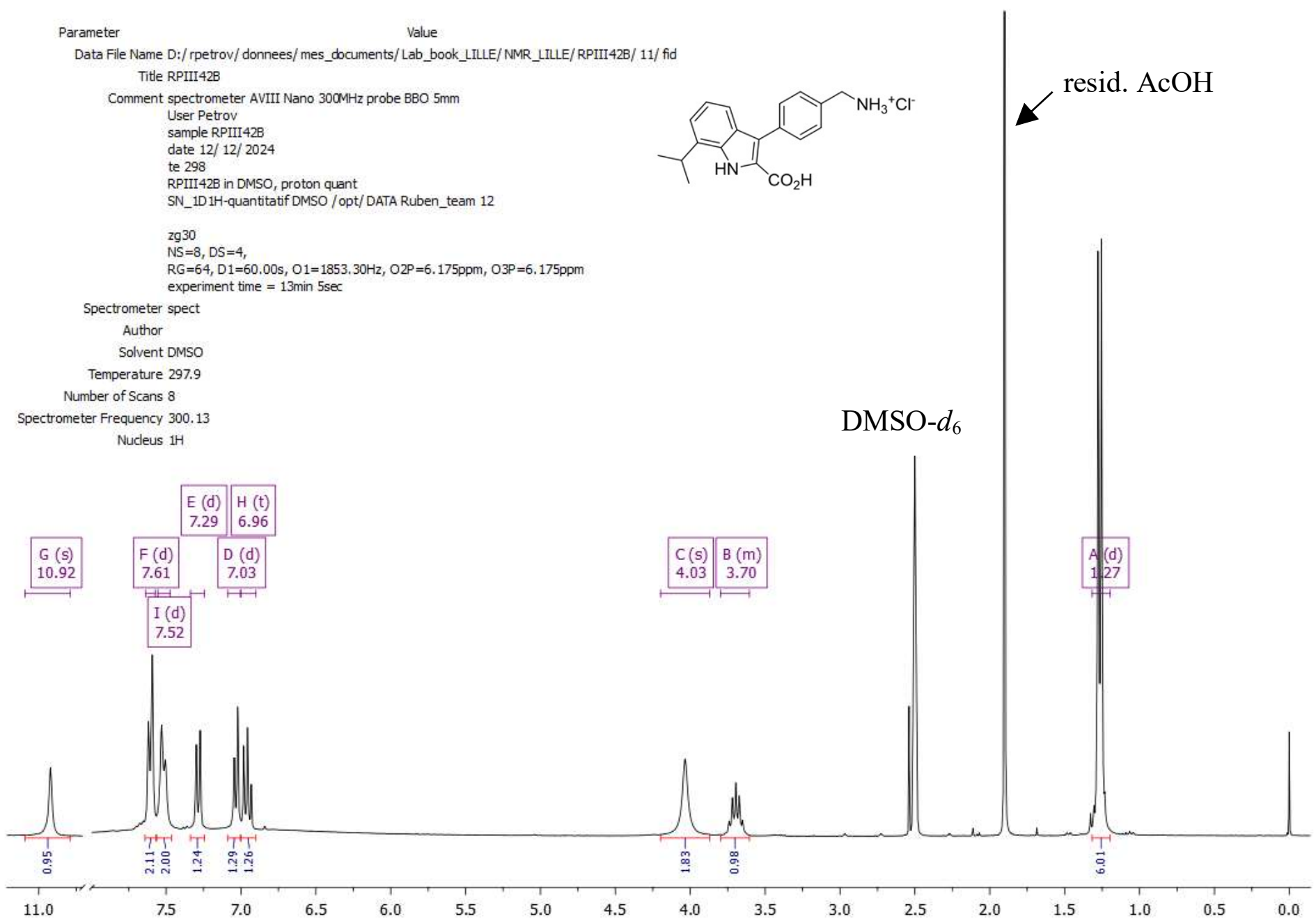

Comp. 28, ^{13}C NMR in DMSO-d_6, Bruker-300

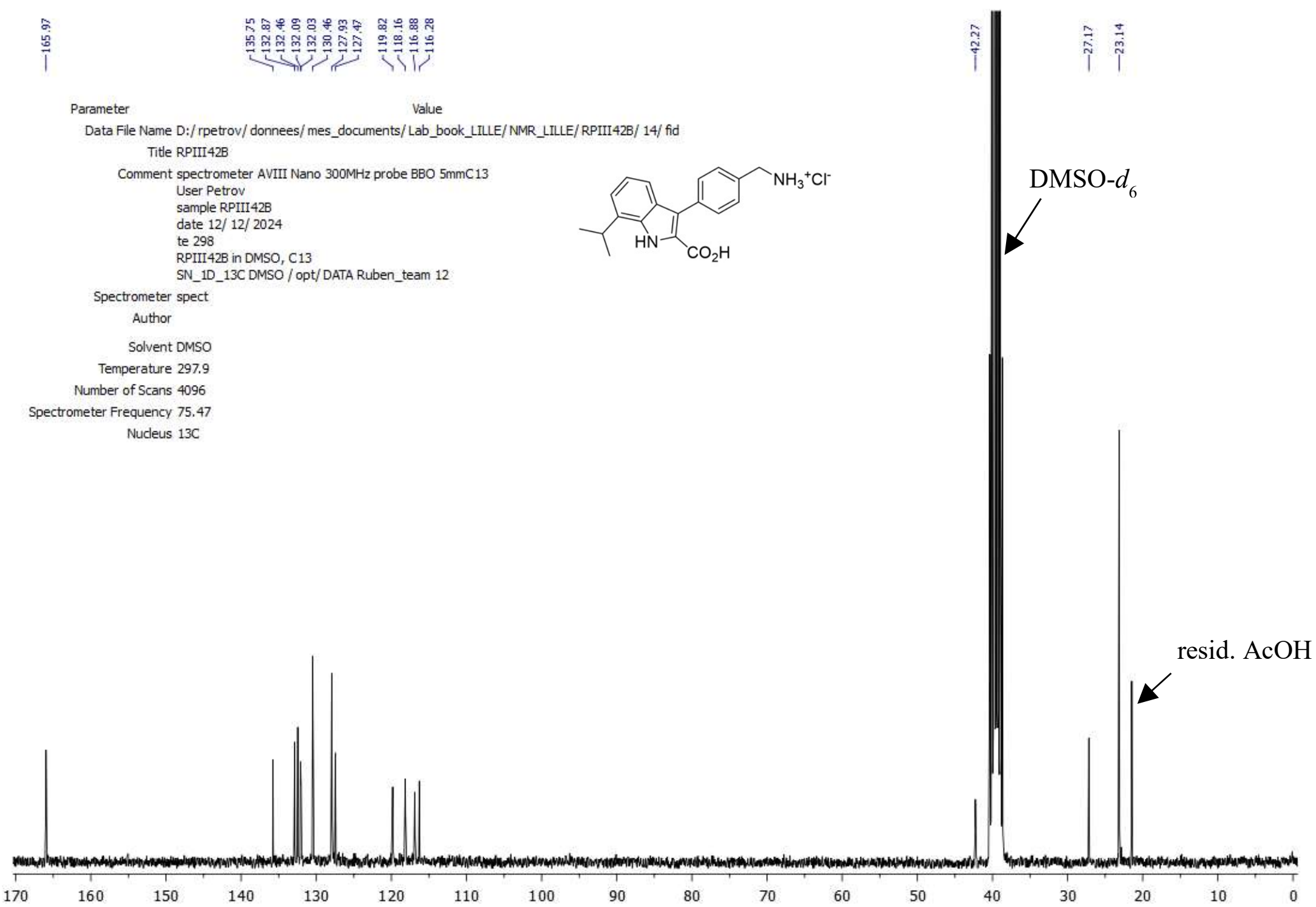

Supplement: Supplementary file 1 [file ja6c08350_si_001.pdf]
