# Supplementary material for: Siderophore-Enabled Delivery of Carbapenem and Metallo-β-Lactamase Inhibitor: A Promising Strategy against Multidrug-Resistant Pseudomonas aeruginosa
Source: J Am Chem Soc. 2026 Sep 27;148(39):41959–77. doi: 10.1021/jacs.6c08350 (PMC13651510; doi:10.1021/jacs.6c08350)
Supplement: Supplementary file 2 [file ja6c08350_si_002.pdf]

Supplementary Information

Supplemental Figures S1-8

and Tables S1-9

Siderophore-Enabled Delivery of Carbapenem and Metallo-β-Lactamase Inhibitor: A Promising Strategy Against Multidrug-Resistant *Pseudomonas aeruginosa*

AUTHOR NAMES: Shahriar SAFARI[1§], Ravil PETROV[1§], Yulia P. USTIMENKO[1], Katy JEANNOT[2], Xavier TRIVELLI[3], Ernesto ANOZ-CARBONELL[1], Ruben C. HARTKOORN[1]*

[1] Univ. Lille, CNRS, Inserm, CHU Lille, Institut Pasteur Lille, U1019 - UMR 9017 - CIIL - Center for Infection and Immunity of Lille, F-59000 Lille, France.

[2] Bacteriology Unit, University Hospital of Besançon, F-25000 Besançon, France; Associated French National Reference Center for Antibiotic Resistance in *Pseudomonas* spp and *Acinetobacter* spp, F-25000 Besançon, France.

[3] Univ. Lille, CNRS, Centrale Lille, UAR2638 - IMEC - Institut Michel-Eugène Chevreul, F-59000 Lille, France.

[§] *S.S.* and *R.P.* contributed equally to this work

[*] ruben.hartkoorn@inserm.fr

Supplemental Figures

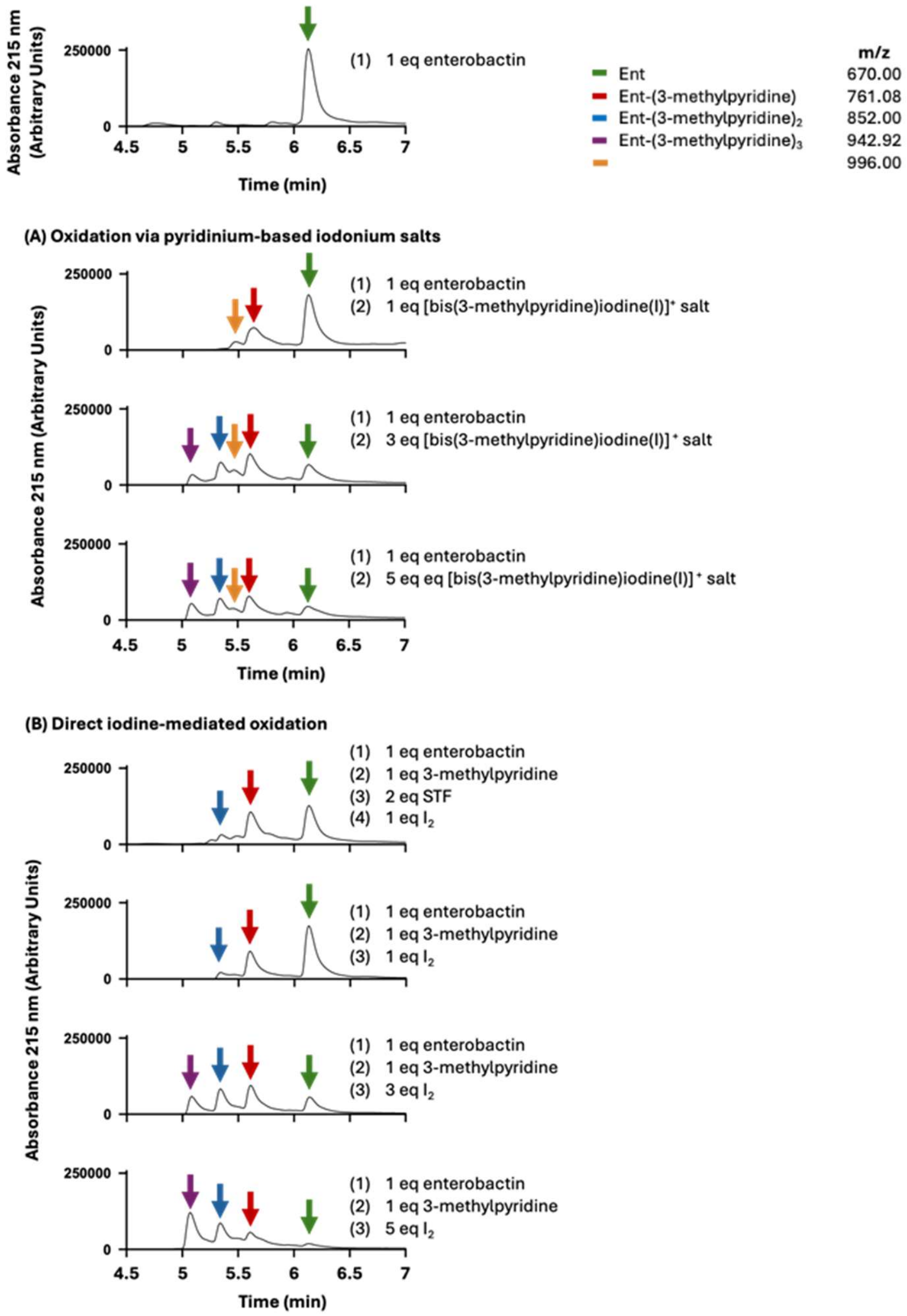

Figure S1. Coupling of Enterobactin [1] with 3-methylpyridine using direct iodine-mediated oxidation (A) and iodonium salt-mediated oxidation (B) strategies. Reactions were performed using different equivalents of the oxidizing reagent (iodine or bis(3-methylpyridine)iodine(I)]$^+$ salt) to evaluate the influence of stoichiometry on the formation of mono-, bis-, and tris-functionalized products. Reactions were performed following the general procedure for model reactions described in the *Synthetic and Analytical Chemistry* section of the Supporting Information. The order of addition of each reaction component is indicated next to each chromatographic trace, with the oxidizing reagent added as the final component to initiate the reaction. Reaction mixtures were analyzed by UHPLC-MS using an Ultimate 3000 UHPLC system coupled to an LCQ Fleet Ion Trap Mass Spectrometer (Thermo Scientific). Chromatographic profiles correspond to UV absorbance at 215 nm, and peak assignments were established based on the corresponding *m/z* values determined by mass spectrometry analysis. Chromatograms are representative of three independent experiments.

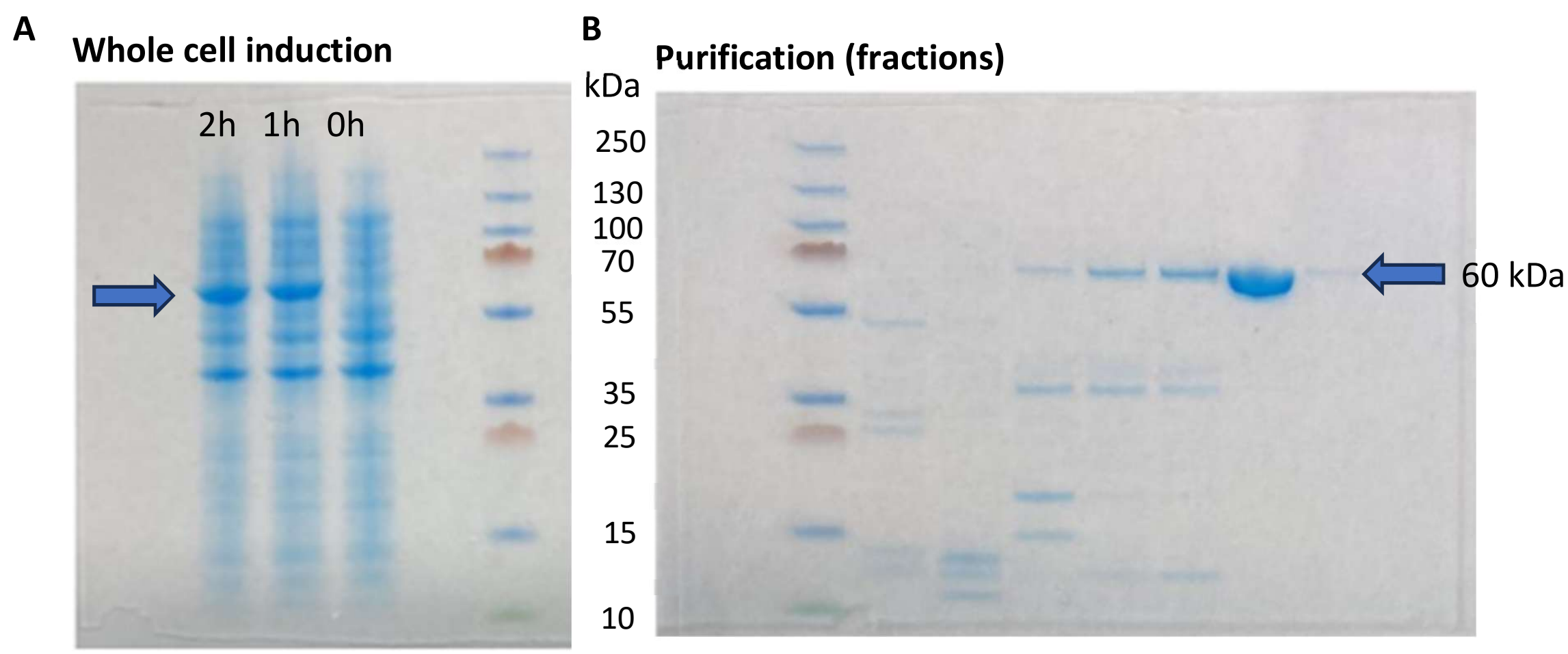

Figure S2. Pa-PBP3 expression and purification. SDS–PAGE analysis of recombinant Pa-PBP3. **(A)** Expression of Pa-PBP3 in *E. coli* NiCo21(DE3) after induction with 1 mM IPTG at 0, 1, and 2 hours. **(B)** Purification fractions of Pa-PBP3 obtained using a HisTrap HP 5 mL column (Cytiva). Pa-PBP3 (~60 kDa) is indicated by blue arrows. Molecular weight markers correspond to the PageRuler™ Plus Prestained Protein Ladder.

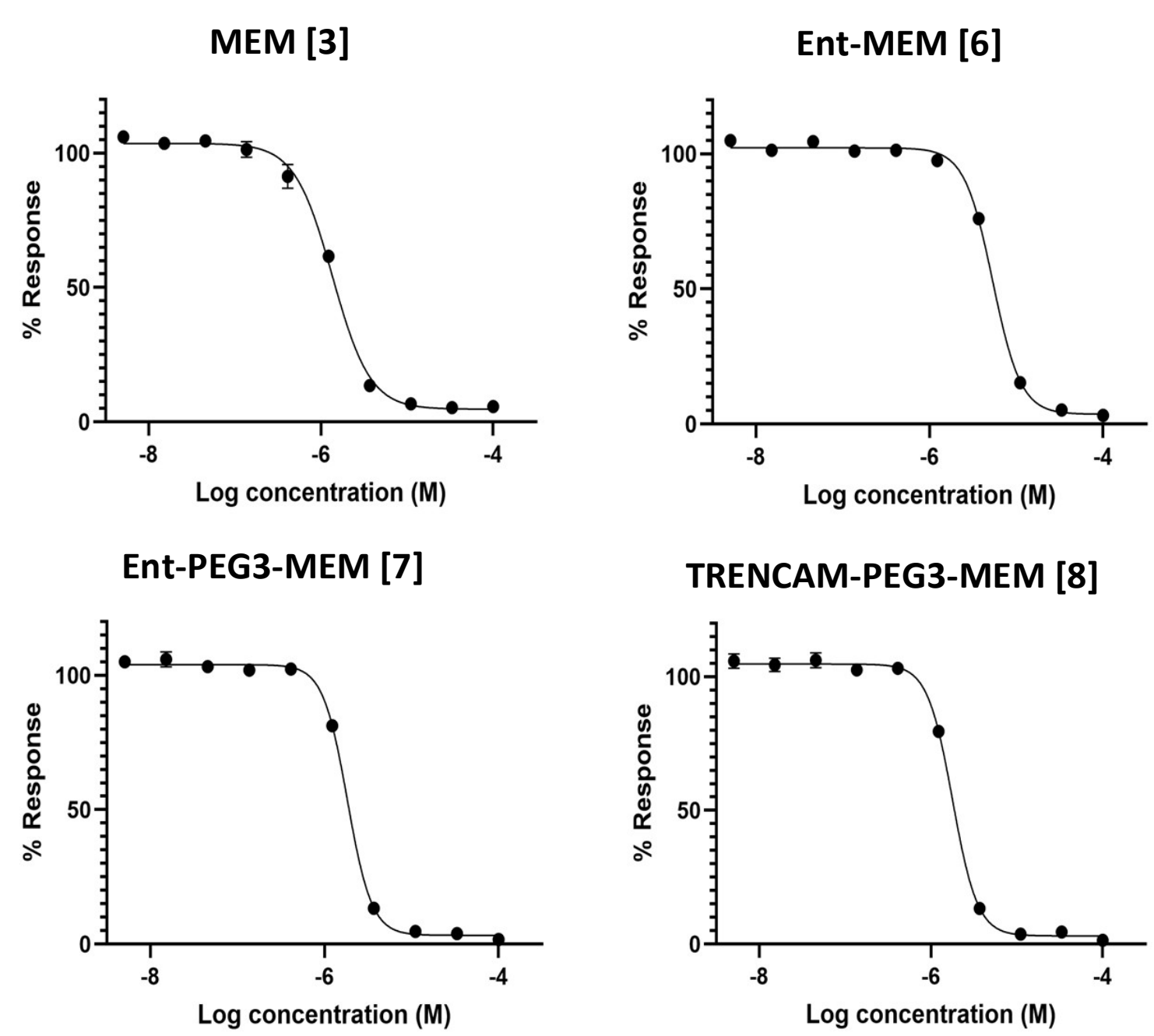

Figure S3. Dose response curves of meropenem conjugates with Pa-PBP3. Assays were performed using a Bocillin-FL-based fluorescence polarization (FP) assay with 1 µM purified recombinant Pa-PBP3. Dose-response curves were generated using GraphPad Prism (v10.4.2). Data are representative of three independent experiments.

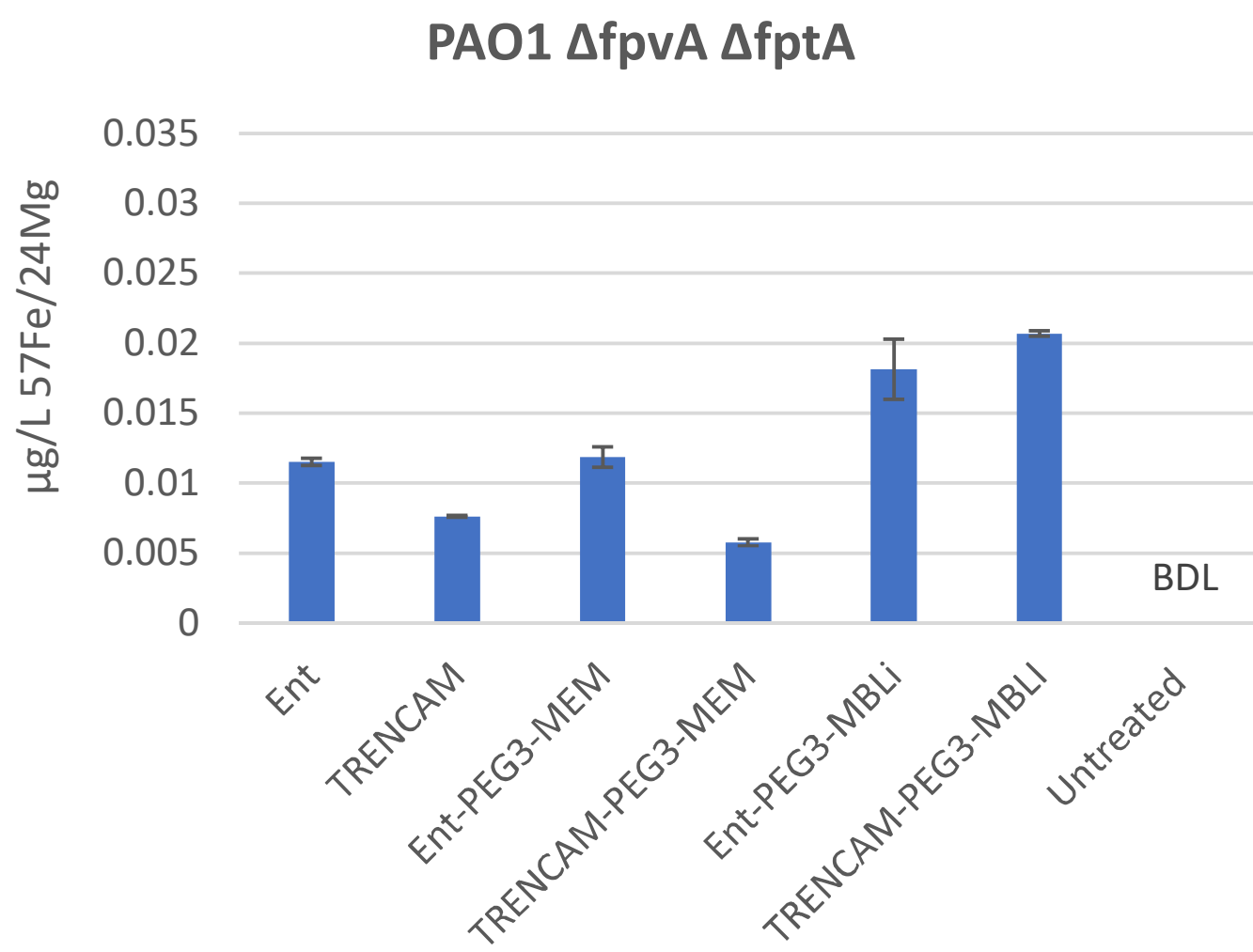

Figure S4. ^{57}Fe uptake by the *P. aeruginosa* FpvA/FptA double knockout strain following incubation with ^{57}Fe-preloaded siderophore conjugates [**7**], [**8**], [**10**], and [**11**]. Intracellular ^{57}Fe was quantified by ICP-MS after 1 h incubation. Results are expressed as $^{57}Fe/^{24}Mg$ (µg/L) and represent two independent experiments. BDL: Below Detection Limit

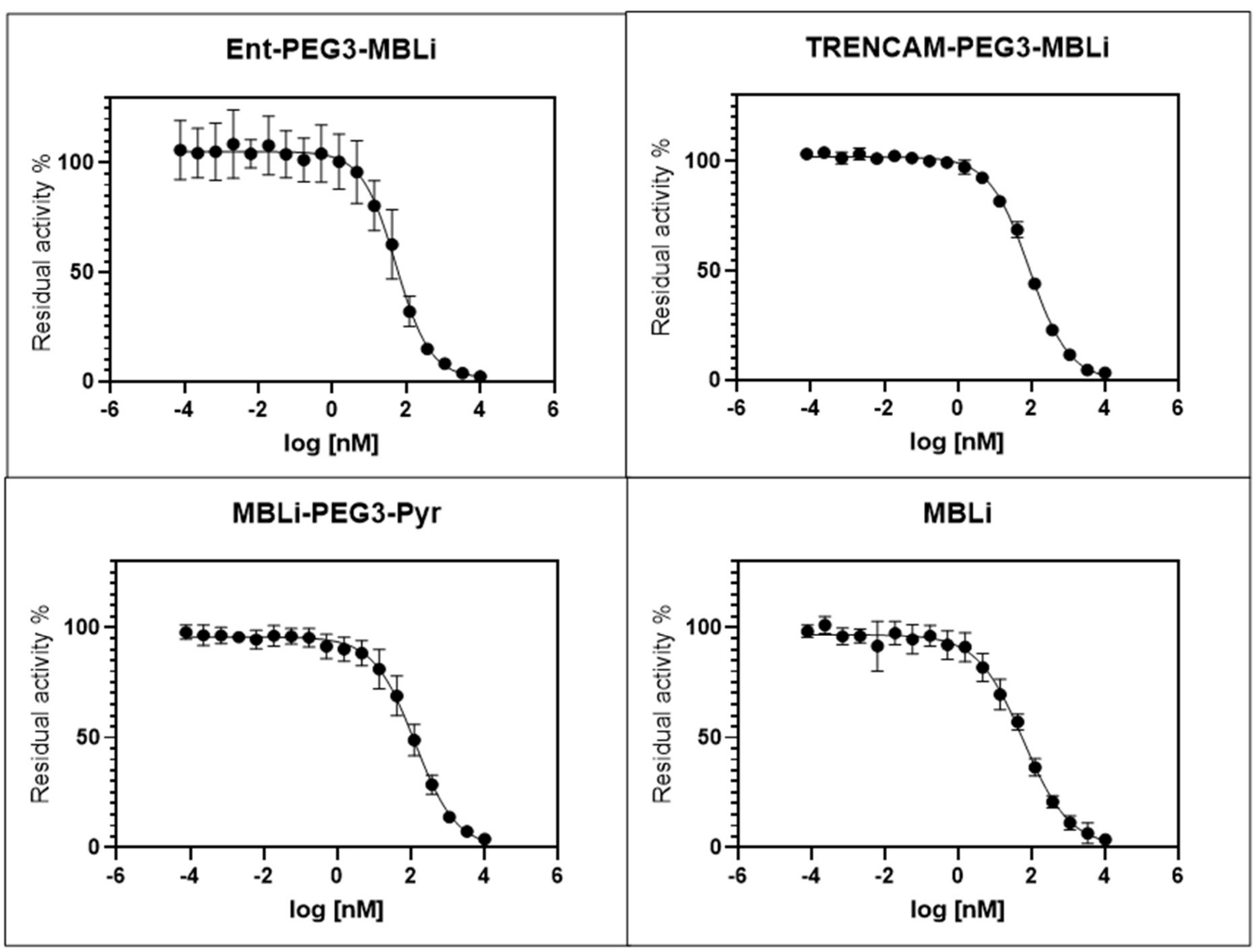

Figure S5. Dose–response curves of MBL inhibitor conjugates against recombinant NDM-1. Inhibition of recombinant NDM-1 was measured using a nitrocefin hydrolysis assay in the presence of increasing concentrations of each inhibitor. Dose–response curves were generated by fitting normalized initial velocity data from three independent experiments to a four-parameter logistic model using GraphPad Prism version 10.4.1.

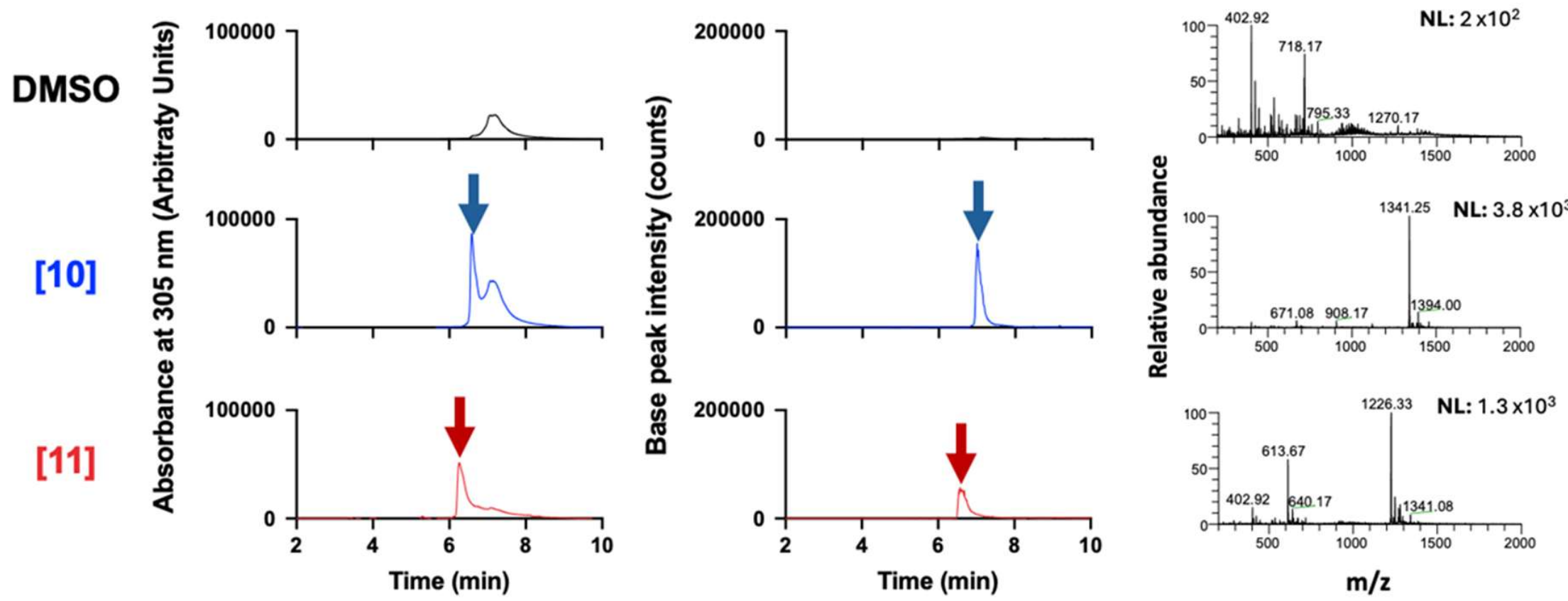

Figure. S6. UHPLC-MS analysis of crude extracts of *P. aeruginosa* incubated with siderophore conjugates [10] and [11]. Representative chromatographic profiles of crude bacterial extracts obtained after 4 h incubation of P. *aeruginosa* PAO1 WT with DMSO (control), [10], or [11]. UV chromatograms recorded at 305 nm are shown on the right, and their corresponding MS base peak chromatograms (positive mode, m/z 200-2000) are shown in the central panel, and the normalized mass spectra acquired between 2 and 10 min are shown on the right, with the corresponding NL value indicated for each spectrum. Arrows indicate the chromatographic peaks corresponding to intact conjugates [10] and [11], assigned by their MS signals (m/z 1226.54 for [10] and m/z 1341.48 for [11]. No additional peaks corresponding to cleavage or degradation products were observed under these conditions. Samples were analyzed by UHPLC-MS using an Ultimate 3000 UHPLC system coupled to an LCQ Fleet Ion Trap Mass Spectrometer (Thermo Scientific). Chromatograms are representative of three independent experiments.

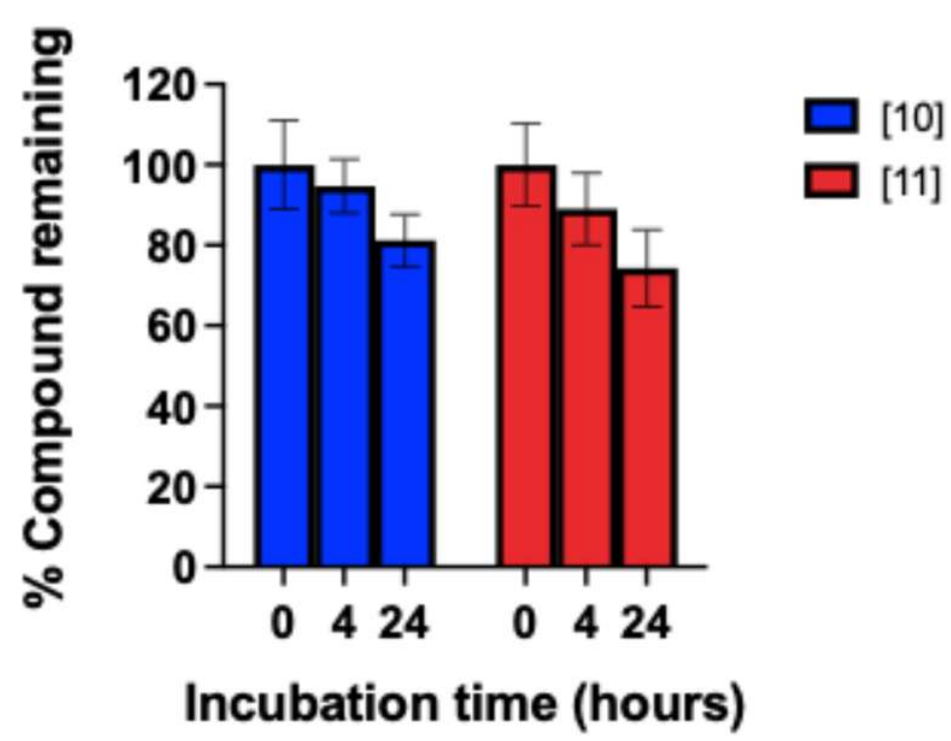

Figure S7. Stability of [10] and [11] in bacteria-free, "spent" media. [10] and [11] was spiked into "spent" media (culture filtrate obtained from an overnight culture of *P. aeruginosa* PAO1 in iron-depleted cation-adjusted Mueller-Hinton) and incubated at 37°C. To determine conjugate stability, samples were taken at different time points (0, 4 and 24 hours), and analyzed by analytical UHPLC-MS, as described in the methods section. The amount of [10] and [11] was quantified using the area under de peak, and presented as a percentage area compared to t=0 (paired). Data shows the mean ± SD of 3 biological replicate data.

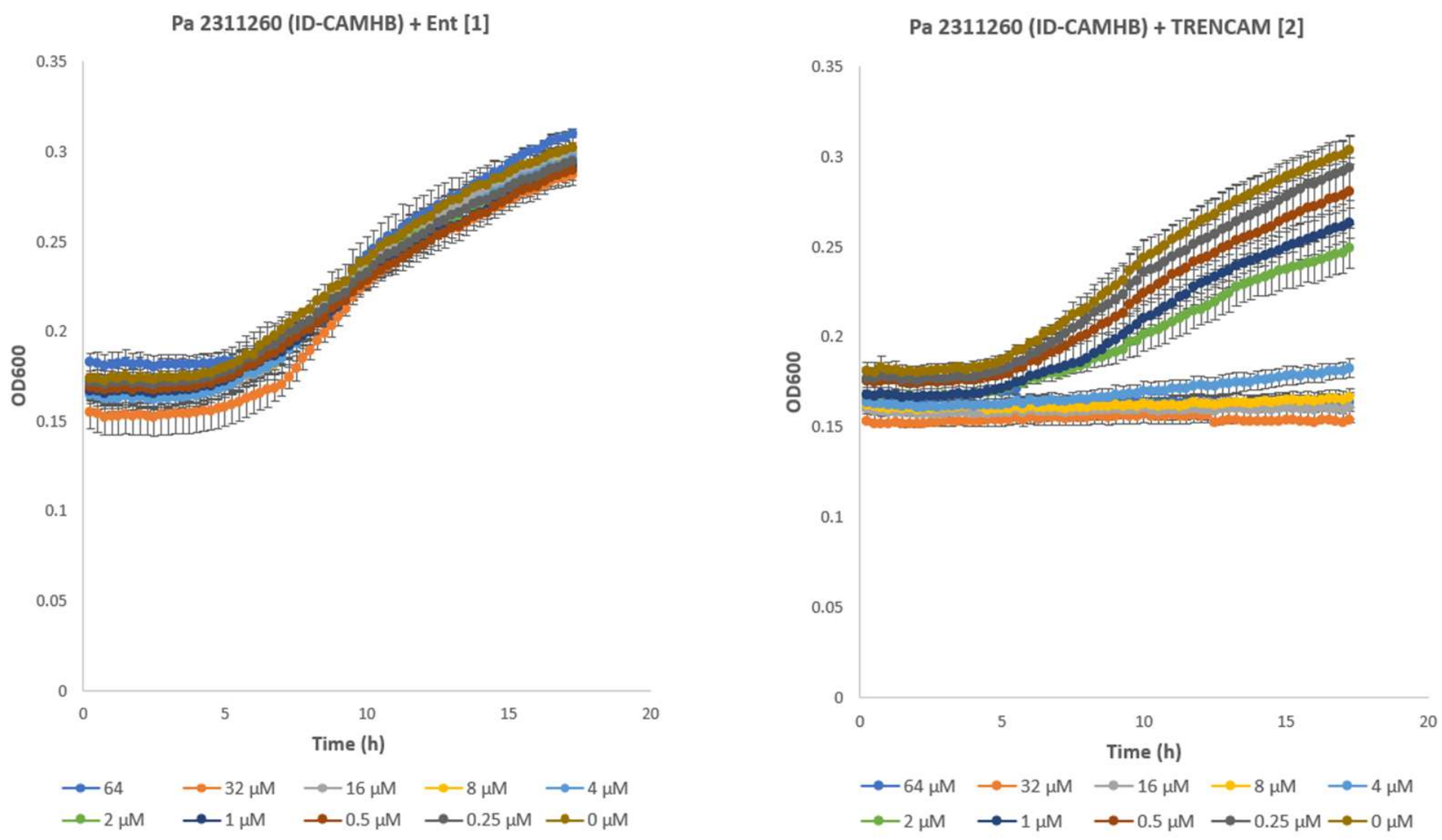

Figure S8. Growth curves of Pa 2311260 grown in ID-CAMHB medium with increasing concentrations of compounds Ent [**1**] and TRENACAM [**2**] (0-64 µM, two-fold dilution). OD_{600} was measured every 15 min. Experiments were performed in duplicate and mean values are shown. Ent [**1**] showed no detectable effect on bacterial growth up to 64 µM, whereas TRENACAM [**2**] inhibited growth in a concentration-dependent manner, with close to no growth observed at 4 µM.

Supplemental Tables

Table S1. Description and antibiotic resistance genoptype of the clinical *P. aeruginosa* strains used in this study. Trunc:Truncated

Strain	source	ST type	Serotype	T3SS	acquired β-lactamase	PDC variant (AmpC)	Intrinsic oxacillinase	aminoglycoside modifying enzymes (AME)	Tetracycline	Chloramphenicol	Trimethoprim	Sulfamide	Macrolide	Quinolone	*GyrA*	*GyrB*	*ParC*	*ParS*	*AmpD*	*PonA*	*mexR*	*MexX*	*MexY*	*MexZ*	*NalD*	*OprD*	*PiuA*	*PfeA*	*FiuA*	*FoxA*	*MutL*	*MutS*
**2311260**	Urines	ST1047	O:7	ExoU	NDM-1	PDC-12	OXA-488	*aphA7, strA, strB*				*sul1*	*msrE, mphE*		T83I		S87L														A391V	
**2512892**	Rectal swab	ST773	O:11	ExoU	NDM-1	PDC-16	OXA-395	*RmtB4*	*tet(G)*			*sul1*		*qnrVC1*	T83I		S87L							VPAL79-82V								
**2412091**	Pus	ST773	O:11	ExoU	NDM-1	PDC-16	OXA-395	*RmtB4, aadA6*	*tet(G)*	*floR2*		*sul1*		*qnrVC1*	T83I		S87L							VPAL79-82V								
**2512537**	Rectal swab	ST773	O:11	ExoU	NDM-1	PDC-16	OXA-395	*rmtB4, aadA6*	*tet(G)*	*floR2*		*sul1*		*qnrVC1*	T83I		S87L							VPAL79-82V								
**2412170**	Ear	ST773	O:11	ExoU	NDM-1	PDC-16	OXA-395	*RmtB4, aadA6*	*tet(G)*	*floR2*		*sul1*		*qnrVC1*	T83I		S87L				L75Q			VPAL79-82V								
**2513161**	Blood culture	ST773	O:11	ExoU	NDM-1	PDC-16	OXA-395	*RmtB4*	*tet(G)*	*floR2*		*sul1*		*qnrVC1*	T83I		S87L							VPAL79-82V								
**196883**	Rectal swab	ST316	O:11	exoU	IMP-1	PDC-36	OXA-395	*rmtB4, strA, strB*	*tet(G)*	*floR2*		*sul1*			T83I					D507N		Trunc.		Trunc.	S32N				T152P			
**2412280**	Urines	ST235	O:11	ExoU	IMP-19	PDC-35	OXA-488	*aac(6')-Ib3, aac(6')-Ib4, aacA4', aadA11, aadB*			*dfrB1*	*sul1*			T83I,D87Y		S87L		V10G					A38V			Trunc.					
**2412054**	Urines	ST267	O:2	ExoS	VIM-2	PDC-3	OXA-1026	*aac(6')-II, strA, strB*																								Q6L
**2411595**	Other	ST233	O:6	ExoS	VIM-2, PER-1, OXA-4	PDC-3	OXA-486	*aacC1, aac(6')-II, aadA2, aphA6, strA, strB*	*tet(G)*	*floR2*	*dfrB5*	*sul1*			T83I	P749S		L145M M250V									Trunc.					
**2512749**	Rectal swab	ST2068	O:5	ExoS	VIM-4	PDC-1	OXA-904	*aacA7, strA, strB*				*sul1*	*mphE msrE*									R282C						V703I		P171L		
**2411694**	Urines	ST308	O:11	ExoU	NDM-1	PDC-19a	OXA-488	*rmtD3, aacC1, aacA4, aadA6, strA, strB*	*tet(G)*	*floR2*	*dfrB5*	*sul1*		*qnrVC1*	T83I		S87L						D237X	R45C	G81X	Q402X					A391V	
**2411726**	Bone	ST773	O:11	ExoU	NDM-1	PDC-16	OXA-395	*rmtB4, aadA6*	*tet(G)*	*floR2*		*sul1*		*qnrVC1*	T83I		S87L							VPAL79-82V		Trunc.						
**2311286**	Other	ST357	O:11	ExoU	NDM-1, LCR-1	PDC-11	OXA-846	*rmtB4, aacA4, aadB, aphA7, strA, strB*	*tet(G)*	*floR2*		*sul1*			T83I		S87L	A13T							F51I	M372V	Trunc.				S436G	

Table S2. Pyridine-modified MBL inhibitor [**9**] was unable to boost meropenem activity in two different NDM-1 producing clinical isolates of *P. aeruginosa* (both with a wild-type OprD porin). Data are derived from checkerboard assays conducted between Pyridine-modified MBL inhibitor [**9**] and meropenem [**3**] in ID-CAMHB.

			**MIC (µM) of MEM [3] in ID-CAMHB**	
		**Strain**	**Pa 2311286**	**Pa 2412170**
		**Metallo-Beta-lactamases**	**NDM-1**	**NDM-1**
		**Other carbapenemase**		
		**TBDT**		
		**OprD**	WT	WT
**pyridine-modified MBL inhibitor [9] (µM)**	16	→	>64	>64
	8	→	>64	>64
	4	→	>64	>64
	2	→	>64	>64
	1	→	>64	>64
	0.5	→	>64	>64
	0.25	→	>64	>64
	0.12	→	>64	>64
	0.06	→	>64	>64
	0.03	→	>64	>64
	0	→	>64	>64

Table S3. The boosting of MEM [**3**] by enterobactin- and TRENCAM- MBL inhibitor conjugates ([**10**] and [**11**] respectively) in a panel of MBL-producing clinical strains of *P. aeruginosa*, and a clinical strain of NDM-1 expressing *A. baumannii*. Data are the results from two independent checkerboard assays.

			**MIC (µM) of MEM [3]** in ID-CAMHB														
		**Strain**	**Pa 2311260**	**Pa 2512892**	**Pa 2412091**	**Pa 2512537**	**Pa 2412170**	**Pa 2513161**	**Pa 196883**	**Pa 2412280**	**Pa 2412054**	**Pa 2411595**	**Pa 2512749**	**Pa 2411694**	**Pa 2411726**	**Pa 2311286**	**Ab CIP7010**
		**Metallo-Beta-lactamases**	**NDM-1**	**NDM-1**	**NDM-1**	**NDM-1**	**NDM-1**	**NDM-1**	**IMP-1**	**IMP-19**	**VIM-2**	**VIM-2**	**VIM4**	**NDM-1**	**NDM-1**	**NDM-1**	**NDM-1**
		**Beta-lactamases**	-			-			-	-	-	PER-1 OXA-4	-	-	-	-	-
		**TBDT**							*fiuA* (T152P)	*piuA* (truncated)		*piuA* (truncated)	*pfeA* (V703I)			*piuA* (truncated)	
		**OprD**	WT	WT	WT	WT	WT	WT	WT	WT	WT	WT	WT	mut (Q402X)	mut (truncated)	mut (M372V)	
**Ent-PEG3-MBLi [10] (µM)**	16	→	0.25-0.5	4-8	8	8	8	32	4	8	16	8-16	32-64	64->64	64	32	0.5-2
	8	→	0.5	4	8	8	8	32	4-8	8	16-32	8-16	>64	64	64	32	4
	4	→	1-2	4	8	8	8	32	4-8	8	32	8-16	>64	64	64	32	8
	2	→	2	8	8	16-32	16	32	8	16	64	16	>64	>64	64	64	16-32
	1	→	4	16	16	32	16	32	8-16	32	64	32	>64	>64	>64	64	32-64
	0.5	→	8	32	32-64	64->64	32	64	16-32	64	>64	32	>64	>64	>64	64	64
	0.25	→	8-16	32	32-64	>64	64	64	16	64	>64	32 -64	>64	>64	>64	64	64->64
	0.12	→	32	>64	>64	>64	64	>64	32	64	>64	64	>64	>64	>64	>64	>64
	0.06	→	>64	>64	>64	>64	>64	>64	32	64	>64	64->64	>64	>64	>64	>64	>64
	0.03	→	>64	>64	>64	>64	>64	>64	64	>64	>64	>64	>64	>64	>64	>64	>64
	0	→	>64	>64	>64	>64	>64	>64	64->64	>64	>64	>64	>64	>64	>64	>64	>64
**TRENCAM-PEG3-MBLi [11] (µM)**	1	→	16-32	>64	>64	64	64	>64	32	-	64	64->64	>64	>64	>64	>64	16-32
	0.5	→	32-64	>64	>64	>64	>64	>64	64	32	>64	>64	>64	>64	>64	>64	32-64
	0.25	→	32-64	>64	>64	>64	>64	>64	64	64	>64	>64	>64	>64	>64	>64	64
	0.12	→	>64	>64	>64	>64	>64	>64	64	64	>64	>64	>64	>64	>64	>64	64
	0.06	→	>64	>64	>64	>64	>64	>64	64	64	>64	>64	>64	>64	>64	>64	64
	0.03	→	>64	>64	>64	>64	>64	>64	64	64->64	>64	>64	>64	>64	>64	>64	64
	0	→	>64	>64	>64	>64	>64	>64	>64	>64	>64	>64	>64	>64	>64	>64	64

Table S4. The antibiotic susceptibility of selected NDM-1 expressing clinical isolates with loss of function mutations in the porin OprD, to Ent-PEG3-MEM [**7**] in the presence and absence of Ent-PEG3-MBLi [**10**] (µM). In these strains Ent-PEG3-MBLi [**10**] was unable to potentiate the activity of MEM [**3**], but could potentiate the activity of the siderophore conjugate Ent-PEG3-MEM [**7**]. Data are the results from two independent checkerboard assays.

			**MIC (µM) of Ent-PEG3-MEM [7]** in ID-CAMHB		
		**Strain**	**Pa 2311286**	**Pa 2411694**	**Pa 2411726**
		**Metallo-Beta-lactamases**	**NDM-1**	**NDM-1**	**NDM-1**
		**Other carbapenemase**	-	-	-
		**TBDT**	*piuA* (truncated)		
		**OprD**	mut (M372V)	mut (Q402X)	mut (truncated)
**Ent-PEG3-MBLi [10] (µM)**	8	→	4	2	2 - 4
	4	→	2	1 - 2	2 - 4
	2	→	1	1 - 2	1 - 2
	1	→	1 - 2	1	1 - 2
	0.5	→	2	4	2 - 4
	0.25	→	>16	16 - >16	8
	0.12	→	>16	>16	>16
	0.06	→	>16	>16	>16
	0.03	→	>16	>16	>16
	0	→	>16	>16	>16

Table S5. The boosting of cefiderocol by enterobactin- and TRENCAM- MBL inhibitor conjugates ([**10**] and [**11**] respectively) in a of panel clinical *P. aeruginosa* strains expressing NDM-1. At concentrations above 1 μM, [10] began to antagonize the activity of CFD, which is thought to be due to competition for available iron**.** Data are the results from two independent checkerboard assays.

			**MIC (μM) of CFD** in ID-CAMHB								
	**Strain**		**Pa 2311260**	**Pa 2512892**	**Pa 2412091**	**Pa 2512537**	**Pa 2412170**	**Pa 2513161**	**Pa 2411694**	**Pa 2411726**	**Pa 2311286**
	**Metallo-β-lactamases**		**NDM-1**	**NDM-1**	**NDM-1**	**NDM-1**	**NDM-1**	**NDM-1**	**NDM-1**	**NDM-1**	**NDM-1**
	**β-lactamases**		-			-			-	-	-
	**TBDT**										*piuA* (truncated)
	**OprD**		WT	WT	WT	WT	WT	WT	mut (Q402X)	mut (truncated)	mut (M372V)
**Ent-PEG3-MBLi [10] (μM)**	8	→	4	4	4	2-4	4	4	8	4	>8
	4	→	2-4	2	2	2-4	2	2	2-4	1-2	8->8
	2	→	1-2	1	1	2	1	1	0.25-0.5	0.25	4-8
	1	→	1-2	0.06	0.06	0.06-0.12	0.06-0.12	0.12	0.12-0.25	0.12-0.25	4
	0.5	→	2	0.12	<0.03	0.06	<0.03	0.06	0.12-0.25	0.12-0.25	2-4
	0.25	→	2	<0.03	<0.03	0.06	<0.03	0.06-0.12	0.12-0.25	0.06-0.12	4
	0.12	→	2-4	<0.03	<0.03-0.06	0.12-0.25	0.06	0.06-0.12	0.25-0.5	0.12	4-8
	0.06	→	2-4	0.06-0.12	0.06	0.25-0.5	0.06	0.12	0.5	0.12-0.25	4-8
	0.03	→	4	0.12	0.12	0.5	0.12	0.12-0.25	1	0.25-0.5	4-8
	0	→	8	0.5	0.5 - 1	1	0.25	0.5	2-4	2-4	8
**TRENCAM-PEG3-MBLi [11] (μM)**	1	→	4	0.12	0.12 -0.25	-	<0.03-0.06	0.12	0.5	0.25	1-2
	0.5	→	4	0.06	0.25 - 0.5	0.125	0.06	0.25	1	0.5 - 1	2
	0.25	→	2-4	0.12 - 0.25	0.25 - 0.5	0.25	0.12	0.25	1	1	2
	0.12	→	2-4	0.25 - 0.5	0.5	0.25 - 0.5	0.25	0.5 - 1	1-2	1-2	2-4
	0.06	→	2-4	0.25 - 0.5	0.5 - 1	0.5	0.25 - 0.5	0.5 - 1	2	2	2-4
	0.03	→	2-4	0.5	1	0.5	0.5 - 1	1	2	2	4
	0	→	4-8	0.5	1 - 2	1	1	1	4-8	4	8

Table S6- HepG2 cytotoxicity quantified s the percentage (%) of propidium iodide positive HepG2 cells following 72 h exposure to the indicated compounds at the indicated concetrations. Data are a mean of 2 biological replicates, each a technical triplicates.

	**HepG2 cytotoxicity** Percentage (%) propidium iodide positive cells at 72 h					
Concentration (µM)	Ent [1]	TRENCAM [2]	Ent-PEG3-MEM [7]	TRENCAM-PEG3-MEM [8]	Ent-PEG3-MBLI [10]	TRENCAM-PEG3-MBLi [11]
100 µM	-1	7	1	1	0	14
50 µM	0	3	1	1	0	0
25 µM	4	2	1	1	0	-1
12.5 µM	1	2	0	0	0	0
6.4 µM	1	1	0	0	-1	0
3.2 µM	0	0	0	0	0	0

Table S7. Evaluation of the pathogenicity of different NDM-producing clinical CRPA strains to *Galleria mellonella* larvae. Data shows the larval mortality 24 h after infection with 20 CFU of different NDM-producing clinical CRPA strains. Five larvae were used per strain. Overall data shows strains Pa 2311260, Pa 2512892 and Pa 2412091 to be more pathogenic than the others.

Strain	Larval deaths (out of 5)	Mortality (%)
**Pa 2311260**	5	100
**Pa 2512892**	5	100
**Pa 2412091**	5	100
**Pa 2512537**	3	60
**Pa 2412170**	3	60
**Pa 2513161**	2	40

Table S8. *p* values for comparisons between the antibiotic (MEM [3] or CFD) and antibiotic + MBL inhibitor ([10] or [11]) treatment groups in *G. mellonella* infection model. Statistical differences between groups were assessed using the Log-rank test and the Gehan-Breslow-Wilcoxon test, as implemented in GraphPad Prism. The Log-rank (Mantel-Cox) test evaluates differences across the entire follow-up period, whereas the Gehan-Breslow-Wilcoxon test gives greater weight to events occurring at earlier time points. The corresponding Kaplan–Meier survival curves are presented in the main text.

Strain	treatment groups		Logrank (Mantel-Cox) test	Gehan-Breslow-Wilcoxon test
Pa 2311260	Infected (PBS treated)	MEM-20 [3]	>0.9999	>0.9999
	MEM-20 [3] + Ent-MBLi [10]	MEM-20 [3]	0.0004	0.0004
	MEM-20 [3] + Tre-MBLi [11]	MEM-20 [3]	0.0004	0.0004
	Infected (PBS treated)	CFD	>0.9999	>0.9999
	CFD + Ent-MBLi [10]	CFD	0.0209	0.0209
	CFD + Tre-MBLi [11]	CFD	0.0293	0.0293
Pa 2412091	Infected (PBS treated)	MEM-20 [3]	0.0074	0.0066
	MEM-20 [3] + Ent-MBLi [10]	MEM-20 [3]	0.0017	0.0004
	MEM-20 [3] + Tre-MBLi [11]	MEM-20 [3]	0.0631	0.0127
	Infected (PBS treated)	CFD	0.0571	0.0571
	CFD + Ent-MBLi [10]	CFD	<0.0001	<0.0001
	CFD + Tre-MBLi [11]	CFD	0.0094	0.0095
Pa 2512892	Infected (PBS treated)	MEM-20 [3]	0.0671	0.0671
	MEM-20 [3] + Ent-MBLi [10]	MEM-20 [3]	0.0018	0.0008
	MEM-20 [3] + Tre-MBLi [11]	MEM-20 [3]	0.0449	0.0654
	Infected (PBS treated)	CFD	0.1462	0.1462
	CFD + Ent-MBLi [10]	CFD	0.0533	0.01
	CFD + Tre-MBLi [11]	CFD	0.6154	0.169

Table S9. List of primer used in this study

No.	Primer sequence (5'→3')	Purpose
1	tatcgataagcttgatatcgaattcCAGCTTAGCCCCTCCCTC	Forward primer for amplification of upstream fragment of *oprD*
2	ggatcgacagACTTCATCACTTTCATTGTGATTGC	Reverse primer for amplification of upstream fragment of *oprD*
3	gtgatgaagtCTGTCGATCCTGTAATCGACCG	Forward primer for amplification of downstream fragment of *oprD*
4	cggccgctctagaactagtggatccGGCCAGGCGACGGATCAT	Reverse primer for amplification of downstream fragment of *oprD*
5	tatcgataagcttgatatcgaattcGCCTGCGCATCGCCCACG	Forward primer for amplification of upstream fragment of *pfeA*
6	acgacgcggtGGGCGCGTGAGGACATCG	Reverse primer for amplification of upstream fragment of *pfeA*
7	tcacgcgcccACCGCGTCGTTCTGAGCCATAAC	Forward primer for amplification of downstream fragment of *pfeA*
8	cggccgctctagaactagtggatccGCCCCGCTCGTTCGCCGA	Reverse primer for amplification of downstream fragment of *pfeA*
9	tatcgataagcttgatatcgaattcGCGCTGGAGGACATTTCC	Forward primer for amplification of upstream fragment of *pirA*
10	acgaggtggtGGAATTGGGGGTACATGC	Reverse primer for amplification of upstream fragment of *pirA*
11	ccccaattccACCACCTCGTTCTGATCG	Forward primer for amplification of downstream fragment of *pirA*
12	cggccgctctagaactagtggatccGCGAACTGGTCGAGGACC	Reverse primer for amplification of downstream fragment of *pirA*
13	tatcgataagcttgatatcgaattcaattcGGCGTCGGCGTACTGCTGC	Forward primer for amplification of upstream fragment of *mexR*
14	tatcctcaagTCACGGGGTAGTTCATTGGTTTGGC	Reverse primer for amplification of upstream fragment of *mexR*
15	taccccgtgaCTTGAGGATATTTAAGAACATTCTTTTCG	Forward primer for amplification of downstream fragment of *mexR*
16	cggccgctctagaactagtggatccTCGCTCGTCGAATGATCAAC	Reverse primer for amplification of downstream fragment of *mexR*
17	tatcgataagcttgatatcgaattcGGGCCCTATCAATCTGCG	Forward primer for amplification of upstream fragment of *fpvA*
18	agtcccagcgCGTGTGGTGCTGGCATGG	Reverse primer for amplification of upstream fragment of *fpvA*
19	gcaccacacgCGCTGGGACTTCTGAGCC	Forward primer for amplification of downstream fragment of *fpvA*
20	cggccgctctagaactagtggatccGGTCGAAGCGTTCTGGAAC	Reverse primer for amplification of downstream fragment of *fpvA*
21	tatcgataagcttgatatcgaattcTACGACGTCGATGCCGGG	Forward primer for amplification of upstream fragment of *fptA*
22	acgcgccccgTCGTCTCCGTTTTCATCGTTCG	Reverse primer for amplification of upstream fragment of *fptA*
23	acggagacgaCGGGGCGCGTTCTGATGC	Forward primer for amplification of downstream fragment of *fptA*
24	cggccgctctagaactagtggatccCCGAACAGGCTGACCAGCAC	Reverse primer for amplification of downstream fragment of *fptA*
29	gccgacgaactggaaagcactc	Forward primer for PCR verification of *pfeA* deletion
30	tcatctaccagccgcgccatc	Reverse primer for PCR verification of *pfeA* deletion
31	caactgctccgcgatctctc	Forward primer for PCR verification of *pirA* deletion
32	ccgggttctgctccttgag	Reverse primer for PCR verification of *pirA* deletion
33	ctctcccgaaccccatcttc	Forward primer for PCR verification of *fpvA* deletion
34	tcaacctggcgggatggtc	Reverse primer for PCR verification of *fpvA* deletion
35	cgtggaattgcgcctggaag	Forward primer for PCR verification of *fptA* deletion
36	aggccatgcaggtcgaacag	Reverse primer for PCR verification of *fptA* deletion
37	cacggatagcgggcatcgttc	Forward primer for PCR verification of *oprD* deletion
38	cagctcagggccgtattgctc	Reverse primer for PCR verification of *oprD* deletion
39	tcgggtcgagctgttgcac	Forward primer for PCR verification of *mexR* deletion
40	cagacgtcgcgggtgaaatc	Reverse primer for PCR verification of *mexR* deletion
41	ATCCTACCATGGACCTGCACGTGATCGACCATGACTTC	Forward primer for amplification of truncated *ftsI* gene
42	ACTCTACTCGAGGCCACGCCCTCCTTTTGCG	Reverse primer for amplification of truncated *ftsI* gene
